# Effects of prenatal small-quantity lipid-based nutrient supplements on pregnancy, birth, and infant outcomes: a systematic review and meta-analysis of individual participant data from randomized controlled trials in low- and middle-income countries

**DOI:** 10.1016/j.ajcnut.2024.08.008

**Published:** 2024-08-16

**Authors:** Kathryn G Dewey, K Ryan Wessells, Charles D Arnold, Seth Adu-Afarwuah, Benjamin F Arnold, Per Ashorn, Ulla Ashorn, Ana Garcés, Lieven Huybregts, Nancy F Krebs, Anna Lartey, Jef L Leroy, Kenneth Maleta, Susana L Matias, Sophie E Moore, Malay K Mridha, Harriet Okronipa, Christine P Stewart

**Affiliations:** 1 Institute for Global Nutrition and Department of Nutrition, University of California, Davis, Davis, CA, United States; 2 Department of Nutrition and Food Science, University of Ghana, Legon, Accra, Ghana; 3 Francis I. Proctor Foundation, University of California, San Francisco, San Francisco, CA, United States; 4 Center for Child, Adolescent and Maternal Health Research, Faculty of Medicine and Health Technology, Tampere University, Tampere, Finland; 5 Department of Paediatrics, Tampere University Hospital, Tampere, Finland; 6 Proyecto Salud y Nutrición, Jhpiego, Guatemala City, Guatemala; 7 Poverty, Health, and Nutrition Division, International Food Policy Research Institute, Washington, DC, United States; 8 Section of Nutrition, Department of Pediatrics, University of Colorado School of Medicine, Denver, CO, United States; 9 Department of Nutrition and Dietetics, School of Global and Public Health, Kamuzu University of Health Sciences, Blantyre, Malawi; 10 Department of Nutritional Sciences and Toxicology, University of California, Berkeley, Berkeley, CA, United States; 11 Department of Women & Children’s Health, King’s College London, London, UK and MRC Unit The Gambia at the London School of Hygiene and Tropical Medicine, Fajara, The Gambia; 12 Center for Non-communicable Diseases and Nutrition, BRAC James P Grant School of Public Health, Bangladesh; 13 Department of Nutritional Sciences, Oklahoma State University, Stillwater, OK, United States

**Keywords:** maternal nutrition, low birth weight, preterm birth, fetal growth restriction, balanced energy protein supplementation, antenatal interventions, infant wasting, infant stunting

## Abstract

**Background:**

Undernutrition during pregnancy increases the risk of giving birth to a small vulnerable newborn. Small-quantity lipid-based nutrient supplements (SQ-LNSs) contain both macro- and micronutrients and can help prevent multiple nutritional deficiencies.

**Objectives:**

We examined the effects of SQ-LNSs provided during pregnancy compared with *1*) iron and folic acid or standard of care (IFA/SOC) or *2*) multiple micronutrient supplements (MMSs) and identified characteristics that modified the estimates of effects of SQ-LNSs on birth outcomes.

**Methods:**

We conducted a 2-stage meta-analysis of individual participant data from 4 randomized controlled trials of SQ-LNSs provided during pregnancy (*n* = 5273). We generated study-specific and subgroup estimates of SQ-LNS compared with IFA/SOC or MMS and pooled the estimates. In sensitivity analyses, we examined whether the results differed depending on methods for gestational age dating, birth anthropometry, or study design.

**Results:**

SQ-LNSs (compared with IFA/SOC) increased birth weight [mean difference: +49 g; 95% confidence interval (CI): 26, 71 g] and all birth anthropometric *z*-scores (+0.10–0.13 standard deviation); they reduced risk of low birth weight by 11%, newborn stunting by 17%, newborn wasting by 11%, and small head size by 15%. Only 2 trials compared SQ-LNSs and MMSs; *P* values for birth outcomes were >0.10 except for head circumference (e.g., *z*-score for gestational age: +0.11; 95% CI: −0.01, 0.23). Effect estimates for SQ-LNSs compared with IFA/SOC were greater among female infants and, for certain outcomes, among mothers with body mass index <20 kg/m^2^, inflammation, malaria, or household food insecurity. Effect estimates for SQ-LNSs compared with MMSs were greater for certain outcomes among female infants, first-born infants, and mothers <25 y.

**Conclusions:**

SQ-LNSs had positive impacts on multiple outcomes compared to IFA/SOC, but further research directly comparing SQ-LNSs and MMSs is needed. Targeting SQ-LNSs to vulnerable subgroups may be worth considering.

**Clinical Trial Registry:**

This study was registered at PROSPERO as CRD42021283391.

## Introduction

Undernutrition is prevalent among females of reproductive age globally, with an estimated 1.2 billion deficient in ≥1 micronutrients [1], 571 million (30%) with anemia [[2], [3], [4]], and 170 million (∼10%) being underweight [3,4]. As a result, many of them enter pregnancy with nutritional deficits. The risk of undernutrition during pregnancy is exacerbated by the elevated nutrient needs to support gestation, particularly in low- and middle-income countries where diets are often inadequate in multiple nutrients [5]. This situation contributes to poor maternal health and the risk of giving birth to a small vulnerable newborn (SVN), an umbrella term that encompasses small-for-gestational age (SGA), preterm birth, and low birth weight (LBW) [6]. In 2020, 26.2% of all live births globally were SVNs, with 16.3% SGA, 8.8% preterm, and 1.1% both SGA and preterm [7]. Although poor nutrition is not the only cause of these outcomes [8], interventions to improve maternal nutrition, such as multiple micronutrient supplements (MMSs) and balanced energy protein (BEP) supplementation for undernourished mothers, should be considered critical elements of antenatal care packages aimed at reducing SVN births [9].

Lipid-based nutrient supplements (LNSs) provide multiple micronutrients embedded in a food base that also provides energy, protein, and fat. LNSs are a type of BEP, as they meet the criterion that protein contributes <25% of the energy content [10]. The intended daily ration of LNSs can be small-, medium-, or large-quantity (SQ-, MQ- or LQ-LNSs) [11]. Maternal SQ-LNSs were designed to fill nutrient gaps during pregnancy and the first 6 mo postpartum with a daily ration of only 20 g (118 kcal/d) [11], which minimizes cost and potential displacement of home-prepared foods. SQ-LNSs are not primarily designed to fill energy gaps, as such gaps can be filled by more affordable local foods [11]. SQ-LNSs provide several key nutrients not provided by iron and folic acid (IFA) supplements or MMSs, including essential fatty acids (EFAs) as well as calcium, magnesium, potassium, and phosphorus [11]. This combination of macro- and micronutrients addresses multiple potential nutritional deficiencies and thus can reduce maternal undernutrition. Maternal SQ-LNSs are currently being distributed by the United States Agency for International Development in selected food aid programs [12], but their use is not yet widespread.

A previous meta-analysis of maternal LNSs [13] demonstrated that LNSs given during pregnancy, compared to IFA, had positive effects on birth weight, length, duration of gestation, SGA, and newborn stunting. That meta-analysis included 3 trials, all of which used SQ-LNSs. In a separate comparison of LNS and MMS that also included 3 trials (2 SQ-LNS, 1 MQ-LNS), there were no significant differences in birth outcomes [13]. More recently, Hunter et al. [14] conducted a meta-analysis of maternal LNSs compared with MMSs that included 4 trials (2 SQ-LNS, 1 MQ-LNS, 1 LQ-LNS); they found a significant reduction in LBW but not preterm birth or SGA. The authors of these previous meta-analyses did not have individual participant data (IPD) and thus were not able to examine individual-level effect-measure modification.

Effect-measure modification analysis can provide important insights regarding the potential for participants to benefit from an intervention as well as their potential to respond [15]. These 2 concepts reflect different attributes, with potential to benefit most likely related to greater deficits at baseline, and potential to respond related to lack of constraints on exhibiting an improvement in the outcome due to factors such as infection or inflammation. In some cases, individuals with greater potential to benefit may also have lower potential to respond, which can limit the beneficial impact of an intervention. Identification of subgroups of pregnant females with the greatest potential to benefit from or respond to LNSs can help inform decisions regarding targeting. We conducted an IPD meta-analysis of randomized controlled trials (RCTs) of SQ-LNSs provided during pregnancy that had 2 objectives: *1*) to compare overall effects of SQ-LNS with provision of i) IFA or standard of care (IFA/SOC) or ii) MMS; and *2*) examine potential modifiers of the estimates of the effects of SQ-LNS (as compared to either IFA/SOC or MMS) on birth outcomes.

## Methods

The protocol for this systematic review and IPD meta-analysis was prospectively registered on PROSPERO (CRD42021283391) [16]. The statistical analysis plan (SAP) is available on Open Science Framework (https://osf.io/nj5f9/) [17] and was posted prior to analysis. The protocol was reviewed by the institutional review board (IRB) of the University of California, Davis and determined to be exempt from IRB approval given that protocols for each individual trial had been previously approved by their respective ethical committees and that all individual trials included participants’ written informed consent.

### Inclusion and exclusion criteria for this IPD meta-analysis

We included prospective RCTs of maternal SQ-LNSs that met the following study-level inclusion criteria: *1*) trial was conducted in a low- or middle-income country [18]; *2*) maternal SQ-LNSs (∼125 kcal/d) were provided for at least part of pregnancy to intervention group participants; *3*) comparison group(s) received IFA, MMSs (defined below), or SOC; *4*) trial reported ≥1 outcome of interest (defined below); and *5*) trial used an individual or cluster-randomized design in which the same participants were measured at baseline and endline (longitudinal follow-up) or different participants were measured at baseline and endline (repeated cross-sectional data collection). Trials were excluded if: *1*) severe or moderate malnutrition was an inclusion criterion for pregnant females to be eligible to participate; *2*) study was conducted with sick or hospitalized populations; *3*) the only available comparison group received other types of non-LNS maternal food supplementation; or *4*) SQ-LNS provision was combined with an additional nutrition-specific intervention within a single arm (e.g., SQ-LNSs + food rations compared with control), and there was no appropriate comparison group that would allow isolation of the SQ-LNS effect (e.g., food rations alone).

For comparisons of SQ-LNSs with MMSs, MMS was defined as including ≥3 micronutrients [19] and similar in form (e.g., tablet or capsule) to globally used MMS formulations [20]. We used the same exclusion criterion as Smith et al. [19], i.e., excluding micronutrient powders because they provide additional nutrients compared to MMS tablets that might have independent effects on the outcomes of interest.

### Search methods and identification of studies

We began the search by considering the studies identified by and included in the 2018 Cochrane systematic review and meta-analysis of the provision of preventive LNSs during pregnancy [13]. We then repeated the database search methods employed in that review to capture any studies published or registered as a randomized trial between January 2018 and September 2021. There were no language restrictions. We searched the following international electronic bibliographic databases: the Cochrane Library (Cochrane Central Register of Controlled Trials, Cochrane Database of Systematic Reviews), MEDLINE (Ovid, In-Process and Other Non-Indexed Citations Ovid, Epub ahead of print Ovid), EMBASE (Ovid), CINAHL Complete (EBSCOhost), Web of Science (Social Sciences Citation Index, Science Citation Index, Conference Proceedings Citation Index-Science, Conference Proceedings Citation Index-Social Sciences), Epistemonikos (current issue), ClinicalTrials.gov, and WHO International Clinical Trials Registry Platform. In addition, we searched 9 regional databases: IBECS, SciELO (Scientific Electronic Library Online), AIM (Africa Index Medicus), IMEMR (Index Medicus for the Eastern Mediterranean Region), LILACS (Latin American and Caribbean Health Sciences Literature), IRIS (PAHO/WHO Institutional Repository for Information Sharing), WPRIM (Western Pacific Index Medicus), IMSEAR (Index Medicus for the South-East Asian Region), and Native Health Research Database.

After reviewing the titles and abstracts of all studies included in the previous review, as well as the additional studies identified by the database searches, we selected all potentially relevant studies for full text review and screened them based on the inclusion and exclusion criteria. After completing the search, we communicated with investigators of all potentially eligible studies in progress, regardless of whether the results had been published.

### Data collection and harmonization

We invited the principal investigators of eligible studies published or in progress to participate in this IPD meta-analysis, and we provided a data dictionary listing definitions of variables requested. Each contacted investigator provided deidentified individual participant datasets with those variables to the IPD analyst (CDA), who communicated with investigators to request any missing variables or other clarifications, as needed.

### IPD integrity

We conducted a complete-case intention-to-treat analysis [21]. We evaluated whether the study sample sizes in our pooled data set were the same as in the study protocols and publications. To address missing outcome data, we tabulated the percentage of participants lost to follow-up between enrollment and the assessment of the outcomes for each study. We also assessed whether missing data were differential with respect to intervention group by comparing rates of missingness across randomized arms.

We flagged biologically implausible values. For anthropometric outcomes, we calculated *z*-scores using the 2006 WHO child growth standards [22,23] and the INTERGROWTH standards [24], checked the values for acceptable SDs, and flagged implausible values if they were outside of published WHO acceptable ranges [22,23]. Implausible values were inspected for errors and either winsorized [25] if within 2 SD of the WHO acceptable ranges or removed from analysis if clearly impossible on an outcome-by-outcome basis. Such cleaning was necessary for <0.2% of participants. There was a consistently low rate of implausibility across outcomes and studies. We also checked summary statistics from the harmonized dataset (e.g., means and SDs) against each trial’s published values.

### Assessment of risk of bias in each study and quality of evidence across studies

Two independent reviewers (KRW and CDA) assessed risk of bias in each trial against the following criteria: random sequence generation and allocation concealment (selection bias), blinding of participants and personnel (performance bias), blinding of outcome assessment (detection bias), incomplete outcome data (attrition bias), selective reporting (reporting bias), and other sources of bias [26]. Any discrepancies were resolved by discussion or consultation with the core working group, as needed. KRW, CDA, and CPS assessed the quality of evidence for anthropometric outcomes across all trials based on the 5 Grading of Recommendations Assessment, Development and Evaluation (GRADE) criteria: risk of bias, inconsistency of effect, imprecision, indirectness, and publication bias [27].

### Specification of outcomes and effect measures

We prespecified all outcomes in the SAP [17]. As shown in Box 1 [[22], [23], [24],^28^], there were 3 categories of outcomes: *1*) birth size and duration of gestation; *2*) anthropometric outcomes at 6 mo of age; and *3*) adverse outcomes (miscarriage, stillbirth, Cesarean section, early neonatal mortality, neonatal mortality, and 0–6 mo mortality). Continuous birth size outcomes included values in units of measurement [e.g., birth weight in grams; birth length, head circumference, and mid-upper arm circumference (MUAC) in centimeters], as *z*-scores [length-for-age *z*-score (LAZ), weight-for-age *z*-score (WAZ), weight-for-length *z*-score (WLZ), BMI-for-age *z*-score (BMIZ), and head circumference-for-age *z*-score (HCZ)] based on WHO Child Growth Standards at birth [22,23], and as *z*-scores for gestational age [weight-for-gestational age *z*-score (WGAZ), length-for-gestational age *z*-score (LGAZ) and head circumference-for-gestational age *z*-score (HCGAZ) based on INTERGROWTH-21st standards [24]. At birth, BMIZ was used instead of WLZ because the latter cannot be calculated for children with lengths <45 cm and exclusion of those infants would create bias; when WLZ can be calculated, these 2 variables are highly correlated (*r* > 0.90). For continuous outcomes based on INTERGROWTH-21st standards, we excluded participants without ultrasound data for calculation of gestational age. Binary birth size outcomes included low birth weight (LBW, <2500 g), birth weight <2000 g, small-for gestational age (SGA, <10th percentile based on INTERGROWTH-21st), large-for-gestational age (LGA, >90th percentile based on INTERGROWTH-21st), newborn stunting (LAZ <−2 SD), low LGAZ (<−2 SD), low BMIZ (<−2 SD), low HCZ (<−2 SD), and low HCGAZ (<−2 SD). Duration of gestation was expressed in weeks, and preterm birth was defined as delivery <37 wk. For binary outcomes based on gestational age, we retained participants without ultrasound dating but conducted a sensitivity analysis in which they were excluded.

BOX 1 **Outcome variables**

**Table:** 

Continuous outcomes	Categorical outcomes
Birth size and duration of gestation	
Birth weight (g), weight-for-age *z*-score (WAZ)	Low birth weight (LBW) <2500 g [28], Birth weight <2000 g
Weight-for-gestational age *z*-score (WGAZ)	Small-for-gestational age (SGA) <10^th^ percentile [24]
	Large-for-gestational age (LGA) >90^th^ percentile [24]
Birth length (cm), length-for-age *z*-score (LAZ)	Newborn stunting, LAZ <−2 SD [22]
Length-for-gestational age *z*-score (LGAZ)	Low LGAZ (LGAZ <−2 SD) [24]
BMI-for-age *z*-score (BMIZ)^1^	Newborn wasting, BMIZ <−2 SD [22]
Birth head circumference (cm), head circumference-for-age *z*-score (HCZ)	Small head circumference, HCZ <−2 SD [23]
Head circumference-for-gestational age *z*-score (HCGAZ)	Low HCGAZ, HCGAZ <−2 SD [24]
Mid-upper arm circumference (MUAC, cm)	
Duration of gestation (wk)	Preterm birth (duration of gestation <37 wk)
Anthropometric outcomes at 6 mo of age	
Weight-for-age *z*-score (WAZ)	Underweight, WAZ <−2 SD [22]
Length-for-age *z*-score (LAZ)	Stunting, LAZ <−2 SD [22]
Weight-for-length *z*-score (WLZ)	Wasting, WLZ <−2 SD [22]
Head circumference-for-age *z*-score (HCZ)	Small head circumference, HCZ <−2 SD [23]
MUAC-for-age *z*-score (MUACZ)	Low MUAC, MUACZ <−2 SD or MUAC <125 mm [23]
	Acute malnutrition, WLZ <−2 SD or MUAC <125 mm [22]
Adverse outcomes	
	Cesarean section
	Miscarriage, embryo, or fetal death <28 wk [28]
	Stillbirth, fetal death >28 wk of gestation [28]
	Early neonatal mortality ≤7 d [28]
	Neonatal mortality *≤*28 d [28]
	Infant mortality <6 mo

Abbreviations: BMIZ, BMI-for-age *z*-score; HCGAZ, head circumference-for-gestational age *z*-score; HCZ, head circumference-for-age *z*-score; LAZ, length-for-age *z*-score; LBW, low birth weight; LGA, large-for-gestational age; LGAZ, length-for-gestational age *z*-score; MUAC, mid-upper arm circumference; MUACZ, mid-upper arm circumference-for-age *z*-score; SD, standard deviation; SGA, small-for-gestational age; WAZ, weight-for-age *z*-score; WGAZ, weight-for-gestational age *z*-score; WLZ, weight-for-length *z*-score.

1 BMIZ is used as a proxy for weight-for-length *z*-score because the latter is not calculated for children with lengths <45 cm [22].

Alt-text: BOX 1

For continuous outcomes, the principal measure of effect was the mean difference (MD) between intervention and comparison groups. For binary outcomes, it was the relative risk (RR) for birth outcomes and adverse outcomes, for which incidence was known, and the prevalence ratio (PR; relative difference in proportions between groups) for anthropometric outcomes measured cross-sectionally at 6 mo of age. We also estimated the effect for binary outcomes as the absolute differences between intervention groups in events per 1000 individuals. The absolute differences are useful for assessing public health impact, but we considered them secondary outcomes because they can be less consistent than RRs or PRs across studies [26].

The comparisons of interest were *1*) SQ-LNS compared with IFA/SOC; and *2*) SQ-LNS compared with MMS. Study arms in which SQ-LNSs were administered prior to conception were excluded from the main analyses because the objectives were to evaluate the effects of SQ-LNSs when given during pregnancy; however, the preconception study arms were included in sensitivity analyses.

### Synthesis methods and exploration of variation in effects

We followed the same procedures for analysis as described previously [29], which included evaluation of full sample main effects of the intervention as well as effect-measure modification (hereafter generally referred to as effect modification, for simplicity) by individual-level characteristics. We had also planned to evaluate effect modification by study-level characteristics [17], but only 4 eligible trials were identified, so there was inadequate statistical power for those analyses and thus they are not described herein.

Briefly, we followed a 2-stage meta-analysis approach [30]. In the first stage, intervention effect estimates (or effect modification interaction term estimates) were generated within each individual study according to its study design. In the second stage, the first-stage estimates were pooled using both inverse-variance fixed effects and random effects.

The individual-level characteristics considered as potential modifiers of the estimates of effects of SQ-LNSs (hereafter referred to as potential effect modifiers) were similar to those included in our previous IPD meta-analysis of trials using child SQ-LNSs [29]. The potential effect modifiers for this analysis are shown in Box 2 [31,32].

BOX 2 **Potential effect modifiers1**

**Table:** 

Individual-level child, maternal and household effect modifiers
Child • Sex (female vs. male) • Birth order (first-born vs. later-born) Maternal • Maternal height (<150.1 cm vs. ≥150.1 cm)^2^ • Maternal BMI (<20 kg/m^2^ vs. ≥20 kg/m^2^)^3^^,^^4^ • Maternal age (<25 y vs. ≥25 y)^4^ • Maternal education (no formal or incomplete primary vs. complete primary or greater) • Baseline anemia status (Hb ≥110 g/L vs. <110 g/L) • Baseline inflammation status (CRP ≤5 mg/L and AGP ≤1 g/L vs. not) • Baseline malaria status (positive rapid test for malaria vs. negative test) • Gestational age at start of supplementation (<14 wk vs. ≥14 wk)^4^ • Compliance with supplementation (≥4 d/wk vs. <4d/wk)^5^ Household • Household socioeconomic status (< study median vs. ≥ study median)^6^ • Household food security (moderate-to-severe food insecurity vs. mild to secure) • Sanitation (unimproved vs. improved)^7^

Abbreviations: AGP, α-1-acid glycoprotein; CRP, C-reactive protein; Hb, hemoglobin; SD, standard deviation.

1 Comparisons follow the format nonreference compared with reference category. Some potential effect modifiers that were listed in the statistical analysis plan were not included: maternal marital status was dropped because of insufficient variation; maternal depression was dropped because it was only evaluated at 6 mo postpartum; source water quality was not included because there was little variation in 2 of the eligible trials; season at time of conception was not included because the seasonal factors that may influence pregnancy outcomes (rainy/dry, harvest/hungry, heat stress) do not always coincide and a 9-mo pregnancy involves exposure to several seasons at different stages of pregnancy.

2 Cutoff is −2 SD for height at 19 y of age: https://www.who.int/growthref/hfa_girls_5_19years_z.pdf?ua=1.

3 When a prepregnancy weight measurement was not available, we estimated weight back to the ninth week of gestation using a restricted cubic spline model regressing baseline weight on gestational age at enrollment with 4 knots based on quintiles in the study’s full data set.

4 Maternal BMI, age, and gestational age at the start of supplementation had different distributions across the different studies. To avoid introducing ecological bias and difficulty disentangling the effect attributable to study-level factors (design, context) rather than individual-level factors, we chose cutoffs that were both biologically relevant and resulted in each study having reasonable sample size in the different strata.

5 Cutoff of >4 d/wk chosen to accommodate the categorical variable for compliance used in the Bangladesh trial [31], which had only these choices: “did not take at all,” “used to take sometimes (1–3 d/wk),” “used to take almost every day (4–6 d/wk),” “used to take regularly every day,” and “other.”

6 Based on a study-defined, study-specific assets index.

7 Improved sanitation includes flush/pour flush to piped sewer system, septic tanks, or pit latrines; ventilated improved pit latrines, composting toilets, or pit latrines with slabs [32]; see Supplemental Table 2, based on baseline data.

Alt-text: BOX 2

We assessed heterogeneity of effect estimates using *I*^2^ and τ^2^ statistics, within strata when relevant [33]. We used a *P* value <0.05 for main effects and a *P*-for-interaction <0.10 for effect modification by individual-level characteristics. Given that the birth outcomes are highly correlated and the effect modification analyses are inherently exploratory, we did not adjust for multiple hypothesis testing because doing so may be unnecessary and counterproductive [34].

### Additional sensitivity analyses

We prespecified several sensitivity analyses:

• Exclusion of trials with a high level of missingness (>20%) of outcome data relative to enrollment for pregnancy outcomes and relative to live births for later outcomes.
• For outcomes that relied on gestational age, exclusion of individuals for whom there was no ultrasound dating of the gestational age of the fetus.
• For birth outcomes (e.g., infant anthropometry), exclusion of individuals for whom data were collected >72 h after birth.
• Exclusion of studies in which additional LNSs were provided to certain mothers (e.g., those with low BMI or gestational weight gain).
• Inclusion of trials/arms that provided SQ-LNSs prior to conception (pooled main effects only).

## Results

### Literature search and study characteristics

**FIGURE 1:**
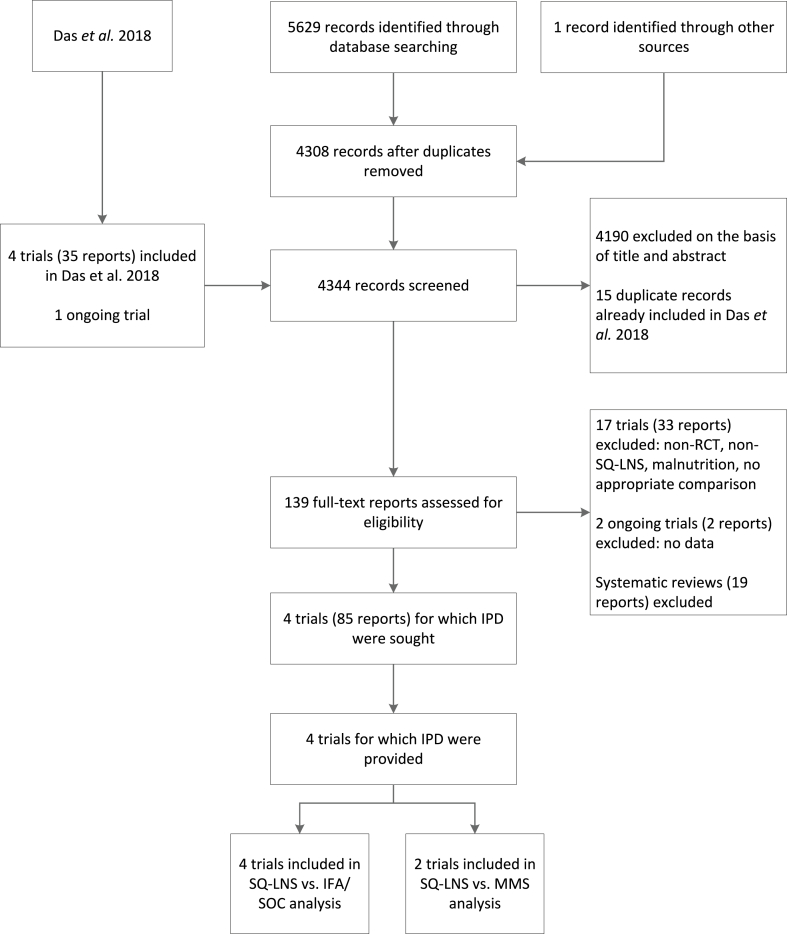
Study flow diagram. IFA, iron and folic acid supplement; IPD, individual participant data; MMS, multiple micronutrient supplement; RCT, randomized controlled trial; SOC, standard of care; SQ-LNS, small-quantity lipid-based nutrient supplement.

We identified 4 trials that met our selection criteria and were completed in time to be included in our analyses, all of which provided IPD (Figure 1, Table 1) [31,[35], [36], [37]]. The trials were conducted in Bangladesh [31], Ghana [35], Malawi [36], and Guatemala [37] between 2009 and 2017. The Guatemala study was 1 of 4 sites in the Women First trial [37]. In that trial, an unfortified LNS (in addition to SQ-LNS) supplying 300 kcal/d was provided to intervention group participants who were either underweight or who had inadequate gestational weight gain. In 3 of the 4 study sites (Democratic Republic of the Congo, India, and Pakistan), >90% of enrolled participants in the intervention groups received this supplement in addition to SQ-LNS, so the total amount of LNS provided to them was ∼418 kcal/d, which would be categorized as MQ-LNS rather than SQ-LNS. Therefore, only the Guatemala site is included in these analyses (where <10% of enrolled participants received the unfortified LNS in addition to SQ-LNS).

**TABLE 1:** Characteristics of trials included in the individual participant data analysis^1^

Country, years of study, study name, *N*, trial design, (author date)	Maternal intervention groups	Comparisons		Maternal characteristics at enrollment				
		SQ-LNS vs. control	SQ-LNS vs. MMS	Gestational age (wk)	Maternal age (y)	Maternal prepregnancy BMI (kg/m^2^)	Nulliparous (%)	Moderately or severely food insecure (%)
Bangladesh, 2011–2015,RDNS, *N* = 4011, cluster RCT, longitudinal follow-up (Mridha et al. [31], 2016)	SQ-LNS from ≤20 wk of gestation to 6 mo postpartum	✓	—	13.1 (3.8)	22.0 (5.0)	20.1 (2.7)	39.7	38.2
	IFA from ≤20 wk of gestation to 3 mo postpartum^2^	✓	—					
Ghana, 2009–2014, iLiNS-DYAD-G, *N* = 1320, RCT, longitudinal follow-up (Adu-Afarwuah et al. [35], 2015)	SQ-LNS from ≤20 wk of gestation to 6 mo postpartum	✓	✓	16.2 (3.3)	26.5 (5.3)	23.9 (4.3)	36.8	29.7
	IFA from ≤20 wk of gestation to delivery, low-dose Ca from delivery to 6 mo postpartum	✓	—					
	MMS from ≤20 wk of gestation to 6 mo postpartum	—	✓					
Malawi, 2011–2014, iLiNS-DYAD-M, *N* = 1391^3^, RCT, longitudinal follow-up (Ashorn et al. [36] 2015)	SQ-LNS from ≤20 wk of gestation to 6 mo postpartum^4^	✓	✓	16.8 (2.1)	25.1 (6.2)	20.5 (2.7)	20.6	66.2
	IFA from ≤20 wk of gestation to delivery, low-dose Ca from delivery to 6 mo postpartum^4^	✓	—					
	MMS from ≤20 wk of gestation to 6 mo postpartum^4^	—	✓					
Guatemala, 2013 - 2017 Women First^5^, N = 1808^6^ RCT, longitudinal follow-up (Hambidge et al. [37], 2019)	SQ-LNS from ≥3 mo preconception to delivery	(✓)^7^	—	11.9 (2.0)	24.4 (4.3)	25.4 (4.1)	6.3	-
	SQ-LNS from start of second trimester (12–14 wk) to delivery	✓	—					
	SOC, which included access to IFA^8^	✓	—					

Abbreviations: IFA, iron and folic acid supplement; iLiNS-DYAD, International Lipid-Based Nutrient Supplements (-G, Ghana; -M, Malawi); MMS, multiple micronutrient supplement; RCT, randomized controlled trial; SOC, standard of care; RDNS, Rang-Din Nutrition Study; SQ-LNS, small-quantity lipid-based nutrient supplement; UNIMMAP, United Nations International Multiple Micronutrient Antenatal Preparation.

1 Detailed nutrient composition of supplements is provided in Supplemental Table 1. IFA in Bangladesh, Ghana, and Malawi contained 60 mg iron + 400 μg folic acid. MMS in Ghana and Malawi was designed to match the SQ-LNS composition for the nutrients in common and thus provided a higher dose of several micronutrients in comparison to the standard UNIMMAP MMS.

2 In 3 study arms of the RDNS trial, participants received IFA during pregnancy and for 3 mo postpartum (these study arms differed in that index children received 1 of 3 different child supplements from 6–24 mo); for the purpose of these analyses, these study arms were combined and compared with the maternal SQ-LNS arm.

3 Total number enrolled in Malawi was 1391. Of the participants, 869 were assigned to the complete intervention and 18-mo follow-up, and 522 participants were assigned to pregnancy supplementation only, with simplified follow-up.

4 Interventions noted in the table were provided to participants assigned to the complete intervention cohort. Participants in the simplified follow-up received supplements (SQ-LNS, IFA, or MMS) from ≤20 wk of gestation to delivery only.

5 The Women First trial provided a protein-energy supplement (in addition to SQ-LNS) to enrolled participants who were either underweight or had inadequate gestational weight gain. In 3 of the 4 study sites (Democratic Republic of the Congo, India, and Pakistan), >90% of enrolled participants received this supplement in addition to SQ-LNS. Therefore, only the Guatemala site is included in these analyses (where <10% of enrolled participants received a protein-energy supplement in addition to SQ-LNS).

6 Total number enrolled (prior to conception) was 1808 in Guatemala; the number of live births for data analysis was 651 (193 in the preconception arm, 229 in the pregnancy arm, 229 in the SOC arm).

7 The study arm that provided preconception SQ-LNS was excluded from the primary comparisons; results including this arm are presented in supplemental materials.

8 At 12 wk of gestation, 51.4% reported consuming iron supplements and 56% reported consuming folic acid supplements; by 32 wk of gestation, the corresponding percentages were 83.3% and 85.1%, respectively.

All 4 trials provided data for the comparison of SQ-LNSs with IFA/SOC; in 3 trials, IFA was provided to this comparison group [31,35,36], and in 1 trial (Guatemala), the comparison group received SOC (biweekly visits to monitor pregnancy status; access to IFA supplements at each clinic visit) [37]. Two of the 4 trials (Ghana and Malawi) also included an arm that received MMSs and thus provided data for the comparison of SQ-LNSs and MMSs [35,36]. In Ghana and in the “complete follow-up” cohort in Malawi (who received supplementation beyond pregnancy, see Table 1), SQ-LNSs and MMSs were provided until 6 mo postpartum, and IFA was provided until delivery followed by placebo (low-dose calcium) until 6 mo postpartum; in the “simplified follow-up” cohort in Malawi (supplementation only during pregnancy), SQ-LNSs, MMSs, or IFA were provided only until delivery [35,36]. In Bangladesh, SQ-LNSs were provided until 6 mo postpartum and IFA until 3 mo postpartum [31], and in Guatemala, SQ-LNSs were provided only until delivery [37]. The nutrient composition of the SQ-LNSs was nearly identical across trials (Supplemental Table 1), except for more vitamin D and less zinc in the version used in Guatemala. The MMS used in Ghana and Malawi was a formulation designed to match the micronutrient content of the SQ-LNS [11], which was based in part on the results of a trial in Guinea-Bissau [38] that demonstrated a greater impact on birth weight when the MMS contained 2× (compared with 1×) the recommended dietary allowance of most of the nutrients (except for iron, folic acid, and vitamin A). Thus, the MMS used in Ghana and Malawi had higher levels of most of the micronutrients compared to the United Nations International Multiple Micronutrient Antenatal Preparation (UNIMMAP) [39].Mean gestational age when prenatal supplementation began was ∼12 wk in Guatemala, ∼13 wk in Bangladesh, and 16–17 wk in Ghana and Malawi (Table 1). Mean maternal age was lowest in Bangladesh (∼22 y) and was 24–27 y in the other sites. Mean maternal BMI was lowest in Bangladesh (20.1 kg/m^2^) and Malawi (20.5 kg/m^2^) and was considerably higher in Ghana and Guatemala (∼24–25 kg/m^2^). The proportion of participants who were nulliparous at enrollment was highest in Bangladesh and lowest in Guatemala. In the 3 sites with compliance data (Bangladesh, Ghana, and Malawi), the percentage reportedly consuming supplements (IFA or SQ-LNSs) >4 d/wk was 81.0%–92.5%. In the same 3 sites, in which household food insecurity was also assessed, the percentage reporting moderate or severe food insecurity ranged from 30% in Ghana to 66% in Malawi. Additional information on maternal, child, and household characteristics is available in Supplemental Table 2.

The study populations differed with regard to risk of adverse birth outcomes, with Bangladesh having the highest incidence (in the IFA/SOC group) of LBW (37%), SGA (59%), newborn stunting (23%), low BMIZ (34%), and preterm birth (14%) (Supplemental Table 3). The incidence of these outcomes in the IFA/SOC groups at the other 3 sites was 13%–14% for LBW, 22%–27% for SGA, 10%–17% for newborn stunting, 6%–11% for low BMIZ, and 8%–12% for preterm birth. Study-level data on anthropometric outcomes at 6 mo of age and adverse outcomes in the IFA/SOC groups are available in Supplemental Tables 4 and 5.

All trials were judged to have low risk of bias for 6 of the 7 categories: random sequence generation, allocation concealment, outcome assessment (except for 1 trial labeled “unclear”), incomplete outcome, selective reporting, and “other” (Supplemental Table 6 and Supplemental Figure 1). All trials had a high risk of bias for blinding of participants, as blinding was not possible given the physical difference in the supplements provided.

### SQ-LNS compared with IFA/SOC

#### Main effects

For most birth outcomes, all 4 trials contributed to the pooled effect estimates, and the total sample size was 4922–5348 (Table 2). For continuous birth outcomes based on INTERGROWTH-21st standards, we excluded participants without ultrasound data, as mentioned above, and as a result, only 3 trials contributed to the estimates for WGAZ, LGAZ, and HCGAZ (total n ∼ 1460–1717).

**TABLE 2:** Main effects of maternal SQ-LNS compared with IFA/SOC on birth outcomes.

Birth outcome	Number of participants (trials)	MD (95% CI)					*P* value^1^	Quality of the evidence (GRADE)
Continuous outcomes^2^								
Birth weight (g)	5273 (4)	48.7 (26.1, 71.2)					<0.001	Moderate
Weight-for-age *z*-score (WAZ)	5273 (4)	0.12 (0.06, 0.17)					<0.001	Moderate
Weight-for-gestational age *z*-score (WGAZ)^3^	1717 (3)	0.13 (0.05, 0.21)					0.001	Moderate
Birth length (cm)	5014 (4)	0.20 (0.09, 0.31)					<0.001	Moderate
Length-for-age *z*-score (LAZ)	5014 (4)	0.11 (0.06, 0.17)					<0.001	Moderate
Length-for-gestational age *z*-score (LGAZ)^3^	1460 (3)	0.13 (0.05, 0.21)					0.002	Moderate
BMI-for-age *z*-score (BMIZ)	5002 (4)	0.10 (0.04, 0.16)					0.002	Moderate
Head circumference (cm)	5016 (4)	0.11 (0.04, 0.18)					0.002	Moderate
Head circumference-for-age *z*-score (HCZ)	5016 (4)	0.10 (0.04, 0.16)					0.001	Moderate
Head circumference-for-gestational age *z*-score (HCGAZ)^3^	1461 (3)	0.11 (0.02, 0.20)					0.019	Moderate
Mid-upper arm circumference (MUAC) (cm)	4581 (3)	0.08 (0.02, 0.14)					0.009	Moderate
Duration of gestation (wk)^3^	5348 (4)	0.12 (0.01, 0.24)					0.040	Moderate

	Number of participants (trials)	SQ-LNS events per 1000	IFA/SOC events per 1000	RR (95% CI)	*P* value^1^	Difference in events per 1000 (95% CI)	*P* value^1^	Quality of the evidence (GRADE)
Binary outcomes^4^								
Low birth weight (LBW)	5273 (4)	168	198	0.89 (0.80, 0.99)	0.033	−28.6 (−51.3, −5.9)	0.013	Moderate
Birth weight <2 kg	5273 (4)	25	35	0.78 (0.60, 1.01)	0.062	−9.1 (−18.5, 0.2)	0.054	Moderate
Small-for-gestational age (SGA)^3^	5181 (4)	303	335	0.96 (0.92, 1.01)	0.133	−23.0 (−46.7, 0.7)	0.057	Moderate
Large-for-gestational age (LGA)^3^	4834 (3)	20	23	1.00 (0.61, 1.65)	0.984	1.1 (−3.1, 5.3)	0.611	Moderate
Newborn stunting	5014 (4)	138	164	0.83 (0.74, 0.93)	0.001	−32.2 (−51.5, −12.9)	0.001	Moderate
Low LGAZ^3^	4922 (4)	118	127	0.90 (0.80, 1.01)	0.082	−12.0 (−29.6, 5.6)	0.182	Moderate
Low BMIZ	5002 (4)	123	145	0.89 (0.81, 0.98)	0.022	−23.6 (−42.7, −4.5)	0.015	Moderate
Low HCZ	5016 (4)	100	118	0.85 (0.75, 0.96)	0.009	−22.1 (−40.5, −3.7)	0.019	Moderate
Low HCGAZ^3^	4924 (4)	54	64	0.88 (0.76, 1.01)	0.072	−6.4 (−18.4, 5.5)	0.291	Moderate
Preterm birth^3^	5348 (4)	106	111	0.94 (0.80, 1.10)	0.445	−7.1 (−25.8, 11.5)	0.454	Moderate

Abbreviations: BMIZ, BMI-for-age *z*-score; CI, confidence interval; GRADE, Grading of Recommendations Assessment, Development and Evaluation; HCGAZ, head circumference-for-gestational age *z*-score; HCZ, head circumference-for-age *z*-score; IFA, iron and folic acid supplement; LAZ, length-for-age *z*-score; LBW, low birth weight; LGA, large-for-gestational age; LGAZ, length-for-gestational age *z*-score; LNS, lipid-based nutrient supplement; MD, mean difference; MUAC, mid-upper arm circumference; RR, relative risk; SGA, small-for-gestational age; SOC, standard of care; SQ-LNS, small-quantity lipid-based nutrient supplement; WAZ, weight-for-age *z*-score; WGAZ, weight-for-gestational age *z*-score.

1 The P value column corresponds to the pooled main effect 2-sided superiority testing of the intervention effect estimate and 95% CI presented in the preceding column.

2 For continuous outcomes, values are MDs: LNS – IFA/SOC (95% CIs). For almost all outcomes, the *I*^2^ value (heterogeneity) was 0, except for WGAZ (*I*^2^ = 0.31). *I*^2^ describes the percentage of variability in effect estimates that may be due to heterogeneity rather than chance. Approximately 0.3–0.6 is considered moderate heterogeneity.

3 For continuous outcomes based on INTERGROWTH-21st standards, we excluded participants without ultrasound data for calculation of gestational age. For the corresponding binary outcomes (SGA, LGA, Low LGAZ, Low HCGAZ) as well as for duration of gestation and preterm birth, we retained participants without ultrasound dating but conducted a sensitivity analysis in which they were excluded (see Supplemental Table 7).

4 For binary outcomes, values are RRs: LNS compared with IFA/SOC (95% CIs). Difference in events per 1000 was calculated using the prevalence difference (95% CI) and multiplying by 1000. For almost all outcomes, the *I*^2^ value (heterogeneity) was 0, except for low BMIZ (*I*^2^ = 0.26 for RR and 0.20 for difference in events per 1000) and Low HCGAZ (*I*^2^ = 0.32 for difference in events per 1000). *I*^2^ describes the percentage of variability in effect estimates that may be due to heterogeneity rather than chance. Approximately 0.3–0.6 is considered moderate heterogeneity.

Maternal SQ-LNSs had a significant positive effect on all of the continuous birth outcomes, with an MD compared to IFA/SOC of +49 g for birth weight; +0.2 cm for birth length; +0.10–0.13 *z*-scores for WAZ, WGAZ, LAZ, LGAZ, BMIZ, HCZ, and HCGAZ; +0.08 cm for MUAC; and +0.12 wk for duration of gestation. Maternal SQ-LNSs reduced risk of LBW by 11%, newborn stunting by 17%, low BMIZ by 11%, and low HCZ by 15%. Effect estimates for the other binary birth outcomes were not statistically significant but were in the same direction [except for LGA; RR: 1.00; 95% confidence interval (CI): 0.61, 1.65]; *P* values were between 0.05 and 0.10 for birth weight <2 kg (RR: 0.78; 95% CI: 0.60, 1.01), low LGAZ (RR: 0.90; 95% CI: 0.80, 1.01), and low HCGAZ (RR: 0.88; 95% CI: 0.76, 1.01).

We rated the quality of the evidence for all birth outcomes as moderate based on the GRADE criteria listed in the Methods: 3–4 RCTs were available for all outcomes, risk of bias was low except for blinding of participants, heterogeneity was low, precision was rated as high because all trials had sample sizes >400, all trials were directly aimed at evaluating SQ-LNSs, and funnel plots revealed no indication of publication bias.

**FIGURE 2:**
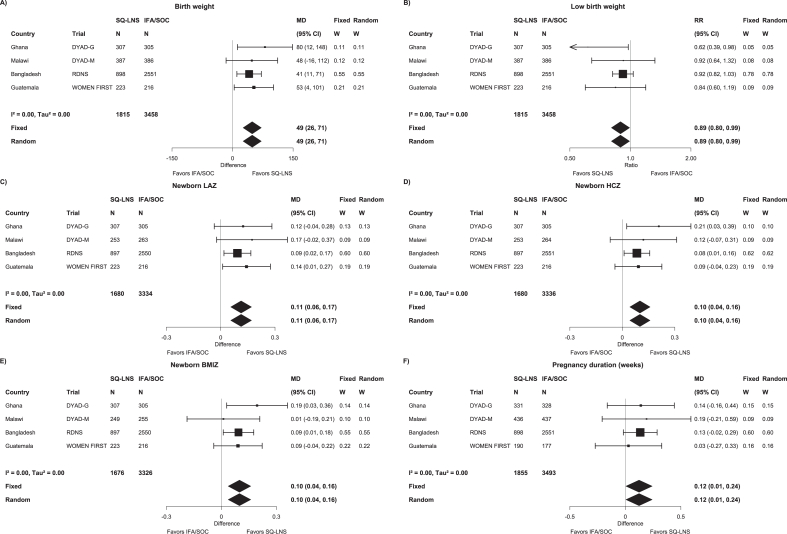
Forest plots of the effect of SQ-LNS compared with that of IFA/SOC on (A) birth weight (g), (B) LBW, (C) newborn LAZ, (D) newborn HCZ, (E) newborn BMIZ, and (F) duration of gestation. Individual study estimates were generated from log-binomial regression for binary outcomes and linear regression for continuous outcomes controlling for baseline measure when available and using robust standard errors for cluster-randomized trials. Pooled estimates were generated using inverse-variance weighting with both fixed and random effects. BMIZ, body mass index *z*-score; CI, confidence interval; HCZ, head circumference-for-age *z*-score; IFA, iron and folic acid; LAZ, length-for-age *z*-score; LNS, lipid-based nutrient supplement; LBW, low birth weight; MD, mean difference; RR, relative risk; SOC, standard of care; SQ-LNS, small-quantity lipid-based nutrient supplement.

In general, there was consistency across studies in the direction of effects, with very low heterogeneity based on *I*^2^ values (Figure 2A–F for birth weight, LBW, newborn LAZ, newborn HCZ, newborn BMIZ, and duration of gestation; Supplemental Figure 2A–BM for all outcomes). Low *I*^2^ values may be attributable to relatively wide CIs for some of the point estimates rather than low variability in the RRs. For nearly all outcomes, fixed-effects and random-effects models generated identical estimates.

With regard to the sensitivity analyses, no trials had a high level of missingness of outcome data, so for that reason, no sensitivity analyses were needed. When including only ultrasound dating for outcomes dependent on gestational age (which required exclusion of the largest trial, in Bangladesh), the magnitude of the effect estimates increased but the 95% CIs were wider and did not meet criteria for significance: RR: 0.86; 95% CI: 0.73, 1.01 for SGA (*n* = 1717), MD: 0.15; 95% CI: −0.03, 0.33 for duration of gestation (*n* = 1880), and RR: 0.90; 95% CI: 0.68, 1.18 for preterm birth (*n* = 1880) (Supplemental Table 7A). For the other sensitivity analyses, restricting birth size outcomes to data collected within 72 h of birth, excluding the Guatemala trial (in which some participants received additional LNS), or including the preconception arm of the Guatemala trial, there was very little change in the findings.

For most of the effects of SQ-LNS compared with IFA/SOC on anthropometric outcomes at 6 mo of age, all 4 trials contributed to the pooled estimates, and the total sample size was ∼5030; outcomes based on MUAC *z*-score were not available for Guatemala (Table 3). Maternal SQ-LNSs reduced underweight at 6 mo (PR: 0.85; 95% CI: 0.73, 0.99), but effects on the other outcomes were not statistically significant; *P* values were between 0.05 and 0.10 for LAZ (MD: +0.06; 95% CI: −0.01, 0.13) and low HCZ (PR, 0.90; 95% CI: 0.80, 1.02). In sensitivity analyses, the PR for underweight became nonsignificant when Guatemala was excluded (PR: 0.96; 95% CI: 0.80, 1.16) (with or without exclusion of the “simplified follow-up” cohort in Malawi; in this cohort and in Guatemala, maternal supplementation did not continue after delivery). The PR for underweight was not strengthened when the preconception arm was included for Guatemala (0.90; 95% CI: 0.78, 1.13). For other outcomes, these sensitivity analyses did not substantively alter the findings (Supplemental Table 7B).

**TABLE 3:** Main effects of maternal SQ-LNS compared with IFA/SOC on infant anthropometric outcomes at 6 mo of age^1^

Infant anthropometric outcomes at 6 mo	Number of participants (trials)	MD (95% CI)					*P* value^2^	Quality of the evidence (GRADE)
Continuous outcomes^3^								
Weight-for-age *z*-score (WAZ)	5029 (4)	0.05 (−0.02, 0.13)					0.173	Moderate
Length-for-age *z*-score (LAZ)	5032 (4)	0.06 (−0.01, 0.13)					0.080	Moderate
Weight-for-length *z*-score (WLZ)	5028 (4)	0.00 (−0.07, 0.07)					0.969	Moderate
Head circumference-for-age *z*-score (HCZ)	5028 (4)	0.01 (−0.05, 0.07)					0.679	Moderate
MUAC-for-age *z*-score (MUACZ)	4600 (3)	0.00 (−0.07, 0.08)					0.949	Moderate

	Number of participants (trials)	SQ-LNS events per 1000	IFA/SOC events per 1000	PR (95% CI)	*P* value^2^	Difference in events per 1000 (95% CI)	*P* value^2^	Quality of the evidence (GRADE)
Binary outcomes^4^								
Underweight	5029 (4)	114	132	0.85 (0.73, 0.99)	0.041	−22.1 (−41.8, −2.5)	0.027	Moderate
Stunted	5032 (4)	198	219	0.90 (0.80, 1.02)	0.102	−17.4 (−43.3, 8.5)	0.187	Moderate
Wasted	5028 (4)	24	32	0.91 (0.66, 1.25)	0.558	−6.3 (−15.8, 3.1)	0.191	Moderate
Low HCZ	5028 (4)	110	125	0.90 (0.80, 1.02)	0.098	−13.2 (−31.9, 5.4)	0.164	Moderate
Low MUAC (MUACZ <−2 SD or MUAC <125 mm)	4600 (3)	68	64	1.00 (0.78, 1.29)	0.970	−0.1 (−17.9, 17.7)	0.993	Moderate
Acute malnutrition (WLZ <−2 SD or MUAC <125 mm)	4595 (3)	80	82	1.00 (0.79, 1.25)	0.970	−0.7 (−19.4, 17.9)	0.938	Moderate

Abbreviations: CI, confidence interval; GRADE, Grading of Recommendations Assessment, Development and Evaluation; HCZ, head circumference-for-age *z*-score; IFA, iron and folic acid supplement; LAZ, length-for-age *z*-score; LNS, lipid-based nutrient supplement; MD, mean difference; MUAC, mid-upper arm circumference; MUACZ, mid-upper arm circumference-for-age *z*-score; PR, prevalence ratio; SD, standard deviation; SOC, standard of care; SQ-LNS, small-quantity lipid-based nutrient supplement; WAZ, weight-for-age *z*-score; WLZ, weight-for-length *z*-score.

1 For continuous outcomes, values are MDs: LNS – IFA/SOC (95% CIs). For binary outcomes, values are PRs: LNS compared with IFA/SOC (95% CIs). Difference in events per 1000 was calculated using the prevalence difference (95% CI) and multiplying by 1000.

2 The *P* value column corresponds to the pooled main effect 2-sided superiority testing of the intervention effect estimate and 95% CI presented in the preceding column.

3 For continuous outcomes, values are MDs: LNS – IFA/SOC (95% CIs). For almost all outcomes, the *I*^2^ value (heterogeneity) was 0, except for LAZ (*I*^2^ = 0.02). *I*^2^ describes the percentage of variability in effect estimates that may be due to heterogeneity rather than chance. Approximately 0.3–0.6 is considered moderate heterogeneity.

4 For binary outcomes, values are PRs: LNS compared with IFA/SOC (95% CIs). Difference in events per 1000 was calculated using the prevalence difference (95% CI) and multiplying by 1000. For almost all outcomes, the *I*^2^ value (heterogeneity) was 0, except for underweight (*I*^2^ = 0.41 for RR and 0.37 for difference in events per 1000) and wasted (*I*^2^ = 0.39 for PR and 0.36 for difference in events per 1000). *I*^2^ describes the percentage of variability in effect estimates that may be due to heterogeneity rather than chance. Approximately 0.3–0.6 is considered moderate heterogeneity.

There were no significant differences in any of the adverse outcomes in the comparison between SQ-LNSs and IFA/SOC, and the RR was generally ≤1 except for incidence of stillbirths (Table 4). Because of uncertainty in gestational age dating, it is sometimes difficult to distinguish between miscarriage and stillbirths (i.e., before compared with after 28 wk of gestation), and in low-resource settings, it can also be difficult to differentiate between stillbirth and early neonatal death [36]. For these reasons, Table 4 includes comparisons for 2 composite variables for these outcomes (miscarriage or stillbirth; miscarriage or stillbirth or early neonatal mortality). The RRs for those composite adverse outcomes were both <1.0. Adverse outcome findings were similar in the sensitivity analyses (Supplemental Table 7C).

**TABLE 4:** Main effects of maternal SQ-LNS compared with IFA/SOC on adverse outcomes^1^.

Adverse outcomes	Number of participants (trials)	SQ-LNS events per 1000	IFA/SOC events per 1000	RR (95% CI)^2^	*P* value^3^	Heterogeneity *(I*^2^*)*^4^	Difference in events per 1000 (95% CI)^2^	P value^3^	Heterogeneity *(I*^*2*^*)*^4^
Cesarean section	5693 (4)	167	156	1.04 (0.90, 1.19)	0.595	0.42	18.6 (−3.6, 40.9)	0.101	0.01
Miscarriage	6102 (4)	49	59	0.87 (0.70, 1.08)	0.213	0.00	0.7 (−7.1, 8.6)	0.852	0.27
Stillbirth	6102 (4)	24	18	1.31 (0.90, 1.91)	0.153	0.49	7.1 (−1.7, 15.9)	0.116	0.53
Miscarriage or stillbirth	6102 (4)	75	77	0.98 (0.81, 1.19)	0.870	0.39	1.4 (−12.3, 15.1)	0.844	0.40
Early neonatal mortality	5506 (4)	14	20	0.74 (0.46, 1.19)	0.208	0.04	−4.8 (−13.2, 3.7)	0.272	0.22
Miscarriage or stillbirth or early neonatal mortality	6102 (4)	89	97	0.93 (0.77, 1.12)	0.452	0.00	−6.6 (−23.4, 10.3)	0.444	0.00
Neonatal mortality	5528 (4)	21	25	0.83 (0.56, 1.24)	0.360	0.00	−2.9 (−12.3, 6.6)	0.551	0.10
Mortality 0–6 mo	5566 (4)	30	32	0.95 (0.69, 1.30)	0.739	0.00	−0.6 (−11.0, 9.8)	0.913	0.00

Abbreviations: CI, confidence interval; GRADE, Grading of Recommendations Assessment, Development and Evaluation; IFA, iron and folic acid supplement; LNS, lipid-based nutrient supplement; RR, relative risk; SOC, standard of care; SQ-LNS, small-quantity lipid-based nutrient supplement.

1 The quality of the evidence based on GRADE criteria was rated as moderate for all adverse outcomes.

2 Values are RRs: LNS compared with IFA/SOC (95% CIs). Difference in events per 1000 was calculated using the prevalence difference (95% CI) and multiplying by 1000.

3 The *P* value column corresponds to the pooled main effect 2-sided superiority testing of the intervention effect estimate and 95% CI presented in the preceding column.

4 *I*^2^ describes the percentage of variability in effect estimates that may be due to heterogeneity rather than chance. Approximately 0.3–0.6 is considered moderate heterogeneity.

#### Effect-measure modification by individual-level characteristics

**FIGURE 3:**
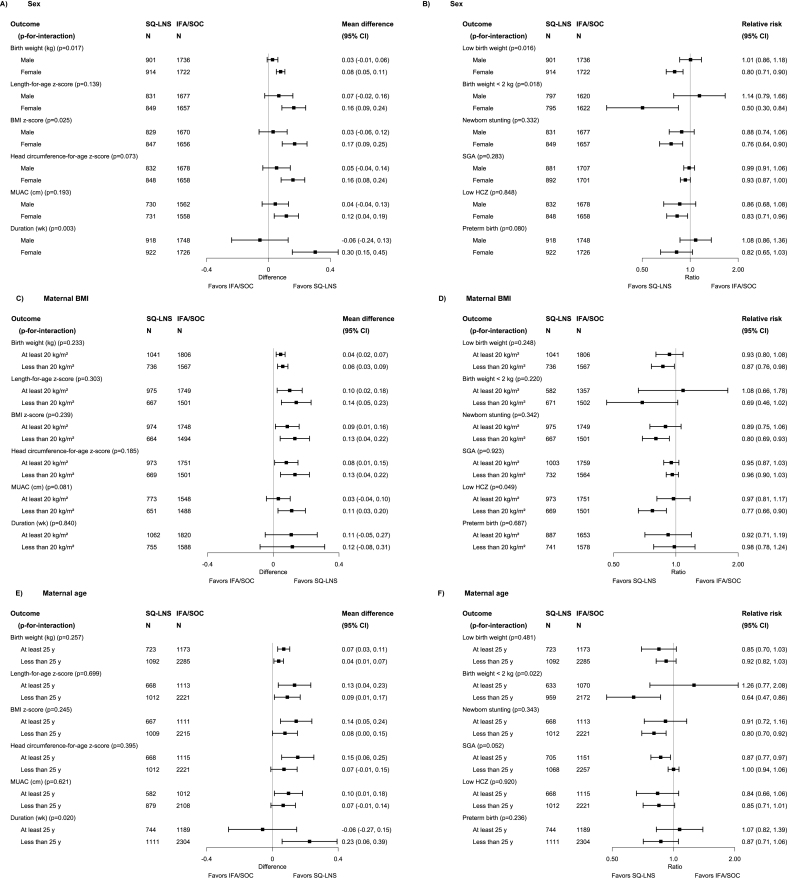

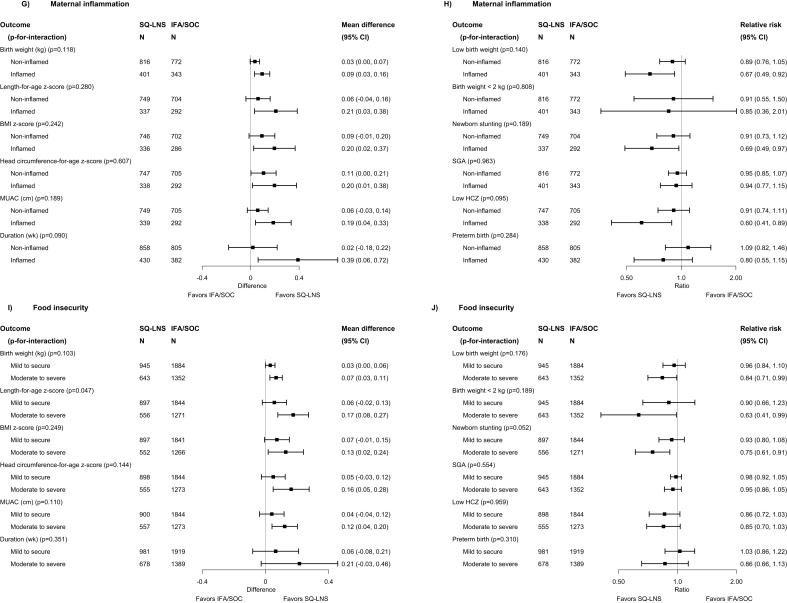
Pooled effects of SQ-LNS compared with those of IFA/SOC on birth outcomes, stratified by selected effect modifiers. (A, B) Sex. (C, D) Material BMI. (E, F) Maternal age. (G, H) Maternal inflammation. (I, J) Food insecurity. Individual study estimates (not shown) were generated from log-binomial regression for binary outcomes and linear regression for continuous outcomes controlling for baseline measures when available and using robust standard errors for cluster-randomized trials. Pooled estimates (shown here) were generated using inverse-variance weighting with fixed effects. CI, confidence interval; HCZ, head circumference-for-age z-score; IFA, iron and folic acid supplement; LNS, lipid-based nutrient supplement; MUAC, mid-upper arm circumference; *P*-for-interaction, *P* value for the interaction indicating the difference in effects of SQ-LNS between the 2 levels of the effect modifier; SGA, small-for-gestational age; SOC, standard of care; SQ-LNS, small-quantity lipid-based nutrient supplement.

For several characteristics, the *P*-for-interaction was >0.10 for all or almost all infant birth outcomes, i.e., effect modification was generally not evident for child birth order; maternal height, education, or anemia at baseline; gestational age at start of supplementation; compliance with supplementation; household socioeconomic status; or sanitation (see Supplemental Figure 3A–N for results stratified by all characteristics). For 5 characteristics, the *P*-for-interaction was <0.10 for ≥2 birth outcomes, and data from ≥3 trials were available (Figure 3A–J shows 12 selected birth outcomes for each of these characteristics, though the number of outcomes for which *P*-for-interaction was <0.10 varied). The estimated effects of maternal SQ-LNSs on birth outcomes were greater among *1*) female (compared with male) infants, for birth weight, WAZ, LBW, birth weight <2 kg, BMIZ, low BMIZ, HCZ, duration of gestation, and preterm birth; *2*) participants with lower BMI (<20 compared with >20 kg/m^2^), for MUAC and low infant HCZ; *3*) younger participants (<25 compared with >25 y), for duration of gestation and birth weight <2 kg (although a greater effect on SGA was seen among those >25 y); *4*) participants with inflammation at baseline (compared with no inflammation), for WAZ (Supplemental Figure 3H1), duration of gestation, and low infant HCZ; and *5*) participants with greater household food insecurity (moderate to severe compared with mild or secure), for birth length (Supplemental Figure 3M1), LAZ, head circumference (Supplemental Figure 3M1) and newborn stunting. Only 2 trials had information on maternal malaria at baseline, but in those trials, the effect estimates for maternal SQ-LNSs were greater among participants with a positive rapid test for malaria at baseline (compared with those with a negative test) for birth weight, WAZ, LBW, and SGA (Supplemental Figures 3I1-2). In sensitivity analyses including only ultrasound dating for outcomes dependent on gestational age (Supplemental Table 8), the interaction with child sex was still evident for duration of gestation but not preterm birth; the interaction with maternal age was still significant (and stronger) for duration of gestation but not for SGA; the interaction with maternal inflammation became weaker for duration of gestation; and there was no change in the interaction with maternal malaria for SGA. In sensitivity analyses restricted to anthropometric outcomes assessed within 72 h of birth, the results were generally stronger for the interactions with child sex and maternal inflammation; similar for maternal BMI, age, and household food insecurity; and weaker for maternal malaria (for birth weight and LBW, but not SGA). The other sensitivity analyses (e.g., exclusion of the Guatemala study) did not substantively alter the results of the effect modification analyses (available at https://osf.io/nj5f9/ [17]).

We explored whether the greater effect of SQ-LNSs on birth size outcomes among female infants (compared with males) could be attributed mainly to the mediating effects on duration of gestation: when including duration of gestation in the models, the *P*-for-interaction was >0.10 for birth weight, WAZ, BMIZ, and HCZ but remained <0.10 for LBW, birth weight <2 kg, and low BMIZ (available at https://osf.io/nj5f9/ [17]).

**FIGURE 4:**
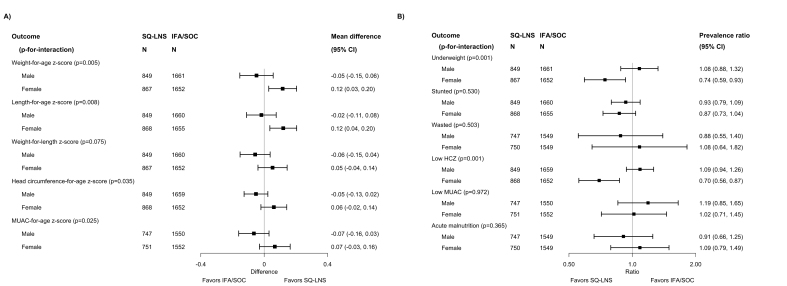
(A, B) Pooled effects of SQ-LNS compared with those of IFA/SOC on anthropometric outcomes at 6 mo of age, stratified by infant sex. Individual study estimates (not shown) were generated from log-binomial regression for binary outcomes and linear regression for continuous outcomes controlling for baseline measures when available and using robust standard errors for cluster-randomized trials. Pooled estimates (shown here) were generated using inverse-variance weighting with fixed effects. CI, confidence interval; HCZ, head circumference-for-age z-score; IFA, iron and folic acid supplement; LNS, lipid-based nutrient supplement; MUAC, mid-upper arm circumference; *P*-for-interaction, *P* value for the interaction indicating the difference in effects of SQ-LNS between the 2 levels of the effect modifier; SOC, standard of care; SQ-LNS, small-quantity lipid-based nutrient supplement.

Some of the above characteristics also modified the effect estimates for the comparison between maternal SQ-LNSs and IFA/SOC with respect to infant anthropometric outcomes at 6 mo, particularly infant sex. Effect estimates were larger for females than for males, as was the case for the birth outcomes (Figure 4). Among female infants, maternal SQ-LNSs were associated with a 26% (95% CI: 7, 41%) reduction in underweight and a 30% (95% CI: 13, 44%) reduction in low HCZ, whereas there was no reduction among males. These findings were very similar in the sensitivity analyses excluding Guatemala (with or without exclusion of the “simplified follow-up” cohort in Malawi) (available at https://osf.io/nj5f9/ [17]). For 3 other characteristics, the *P*-for-interaction was <0.10 for ≥2 distinct infant outcomes at 6 mo. Effect estimates for maternal SQ-LNSs were greater among *1*) participants with greater food insecurity, for LAZ and underweight at 6 mo (Supplemental Figure 3M3, 4); *2*) participants with improved sanitation, for WAZ and WLZ at 6 mo (Supplemental Figure 3N3); and *3*) later-born children, for underweight and stunting at 6 mo (Supplemental Figure 3B4).

Supplemental Figure 4A–N presents pooled estimates for adverse outcomes, stratified by potential individual-level effect modifiers. For many of the potential effect modifiers, the *P*-for-interaction was >0.10 for all of the adverse outcomes; for several other potential effect modifiers, the *P*-for-interaction was <0.10 for only 1 adverse outcome, and for that outcome the difference between the SQ-LNS and IFA/SOC groups was not significant in either subgroup. For birth order, the *P*-for-interaction was <0.10 for 2 outcomes: neonatal mortality and mortality 0–6 mo. In both cases, the stratum-specific estimates were not significant: neonatal and 0–6 mo mortality RRs were respectively 0.54 (95% CI: 0.27, 1.06) and 0.60 (95% CI: 0.32, 1.15) among first-born infants, and 1.08 (95% CI: 0.69, 1.70) and 1.22 (95% CI: 0.86, 1.73) among later-born infants. These results were similar in the sensitivity analyses (available at https://osf.io/nj5f9/ [17]).

### SQ-LNS compared with MMS

#### Main effects

**FIGURE 5:**
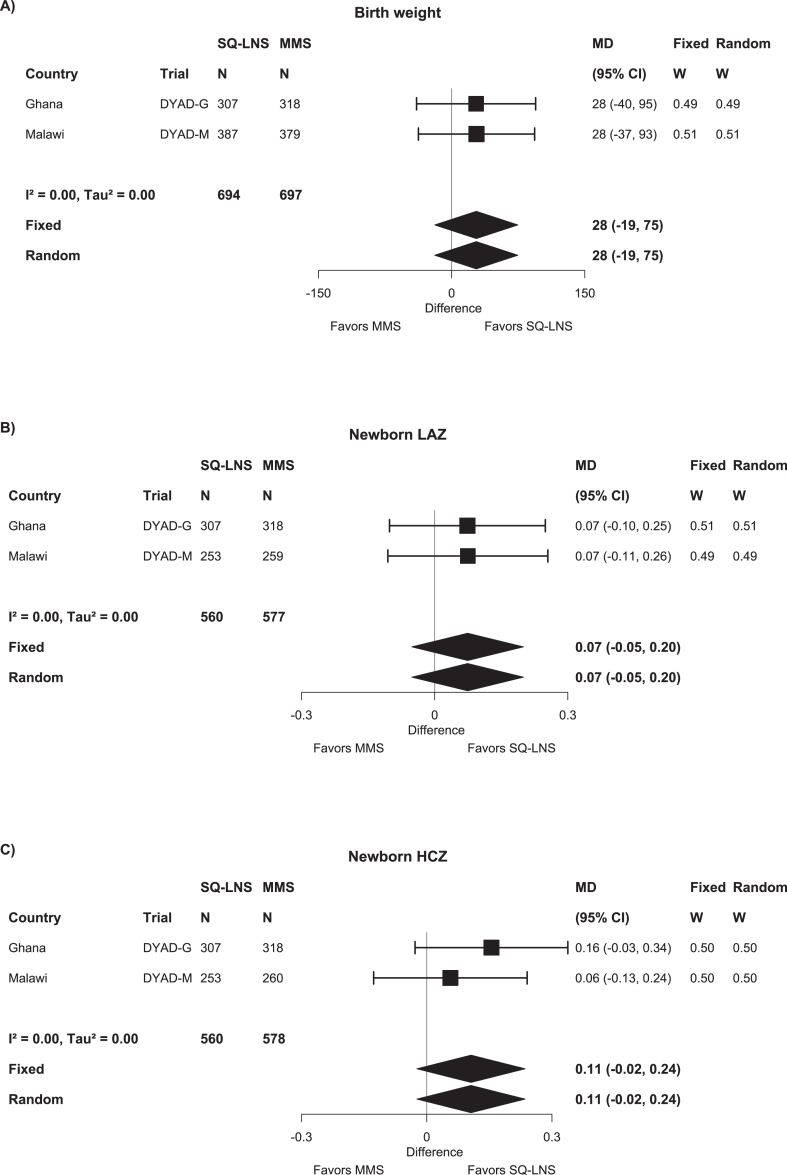
Forest plots of the effect of SQ-LNS compared with that of MMS on (A) birth weight (g), (B) newborn LAZ, and (C) newborn HCZ. Individual study estimates were generated from log-binomial regression for binary outcomes and linear regression for continuous outcomes controlling for baseline measures when available and using robust standard errors for cluster-randomized trials. Pooled estimates were generated using inverse-variance weighting with both fixed and random effects. CI, confidence interval; HCZ, head circumference-for-age *z*-score; LAZ, length-for-age *z*-score; LNS, lipid-based nutrient supplement; MD, mean difference; MMS, multiple micronutrient supplement; SQ-LNS, small-quantity lipid-based nutrient supplement.

Comparison of the effects of SQ-LNSs and MMSs on birth outcomes is based on 2 trials and a total sample size of 1121–1539 (Table 5). *P* values were generally >0.10 except for 2 outcomes with *P* values between 0.05 and 0.10: head circumference (+0.14 cm; 95% CI: −0.02, 0.31 cm) and HCGAZ (+0.11; 95% CI: −0.01, 0.23). There was low heterogeneity, and fixed-effects and random-effects models generated nearly identical estimates (Figure 5A–C and Supplemental Figure 5A–AF). We rated the quality of the evidence for all outcomes as low because data were available for only 2 trials, both conducted in Africa.

**TABLE 5:** Main effects of maternal SQ-LNS compared with MMS on birth outcomes.

Birth outcome	Number of participants (trials)	MD (95% CI)					*P* value^1^	Quality of the evidence (GRADE)
Continuous outcomes^2^								
Birth weight (g)	1391 (2)	27.8 (−19.1, 74.6)					0.245	Low
Weight-for-age *z*-score (WAZ)	1391 (2)	0.06 (−0.05, 0.17)					0.263	Low
Weight-for-gestational age *z*-score (WGAZ)^3^	1377 (2)	0.06 (−0.05, 0.16)					0.294	Low
Birth length (cm)	1137 (2)	0.16 (−0.09, 0.40)					0.209	Low
Length-for-age *z*-score (LAZ)	1137 (2)	0.07 (−0.05, 0.20)					0.247	Low
Length-for-gestational age *z*-score (LGAZ)^3^	1123 (2)	0.08 (−0.04, 0.20)					0.214	Low
BMI-for-age *z*-score (BMIZ)	1121 (2)	0.01 (−0.12, 0.14)					0.830	Low
Head circumference (cm)	1138 (2)	0.14 (−0.02, 0.31)					0.088	Low
Head circumference-for-age *z*-score (HCZ)	1138 (2)	0.11 (−0.02, 0.24)					0.109	Low
Head circumference-for-gestational age *z*-score (HCGAZ)^3^	1124 (2)	0.11 (−0.01, 0.23)					0.066	Low
Mid-upper arm circumference (MUAC) (cm)	1142 (2)	0.08 (−0.02, 0.18)					0.127	Low
Duration of gestation (wk)	1539 (2)	−0.04 (−0.27, 0.20)					0.771	Low

	Number of participants (trials)	SQ-LNS events per 1000	MMS events per 1000	RR (95% CI)^4^	*P* value^2^	Difference in events per 1000 (95% CI)^4^	*P* value^1^	Quality of the evidence (GRADE)
Binary outcomes								
Low birth weight (LBW)	1391 (2)	105	119	0.88 (0.66, 1.18)	0.397	−14.2 (−47.3, 18.8)	0.399	Low
Birth weight <2 kg	1391 (2)	19	19	1.07 (0.52, 2.23)	0.850	−1.1 (−15.1, 12.9)	0.877	Low
Small-for-gestational age (SGA)^3^	1391 (2)	214	224	0.96 (0.79, 1.17)	0.717	−9.3 (−52.7, 34.2)	0.677	Low
Large-for-gestational age (LGA)^3^	1391 (2)	32	28	1.16 (0.64, 2.10)	0.622	5.2 (−12.6, 23.1)	0.564	Low
Newborn stunting	1137 (2)	118	110	1.06 (0.77, 1.47)	0.710	5.5 (−31.3, 42.3)	0.770	Low
Low LGAZ^3^	1137 (2)	109	88	1.22 (0.85, 1.74)	0.276	11.8 (−19.6, 43.2)	0.463	Low
Low BMIZ	1121 (2)	67	68	0.99 (0.64, 1.53)	0.964	−1.2 (−30.8, 28.5)	0.939	Low
Low HCZ	1138 (2)	56	43	1.27 (0.77, 2.10)	0.352	12.3 (−13.0, 37.6)	0.341	Low
Low HCGAZ^3^	1138 (2)	16	17	0.85 (0.35, 2.05)	0.711	−1.6 (−16.6, 13.4)	0.831	Low
Preterm birth	1539 (2)	99	79	1.21 (0.88, 1.66)	0.244	18.4 (−10.2, 47.0)	0.206	Low

Abbreviations: BMIZ, BMI-for-age *z*-score; CI, confidence interval; GRADE, Grading of Recommendations Assessment, Development and Evaluation; HCGAZ, head circumference-for-gestational age *z*-score; HCZ, head circumference-for-age *z*-score; IFA, iron and folic acid supplement; LAZ, length-for-age *z*-score; LBW, low birth weight; LGA, large-for-gestational age; LGAZ, length-for-gestational age *z*-score; LNS, lipid-based nutrient supplement; MD, mean difference; MMS, multiple micronutrient supplement; MUAC, mid-upper arm circumference; RR, relative risk; SGA, small-for-gestational age; SQ-LNS, small-quantity lipid-based nutrient supplement; WAZ, weight-for-age *z*-score; WGAZ, weight-for-gestational age *z*-score.

1 The *P* value column corresponds to the pooled main effect 2-sided superiority testing of the intervention effect estimate and 95% CI presented in the preceding column.

2 For continuous outcomes, values are MDs: LNS – IFA/SOC (95% CIs). For nearly all outcomes, the *I*^2^ value (heterogeneity) was 0, except for head circumference (*I*^2^ = 0.08). *I*^2^ describes the percentage of variability in effect estimates that may be due to heterogeneity rather than chance. Approximately 0.3–0.6 is considered moderate heterogeneity.

3 For continuous outcomes based on INTERGROWTH-21st standards, we excluded participants without ultrasound data for calculation of gestational age. For the corresponding binary outcomes (SGA, LGA, Low LGAZ, Low HCGAZ), we retained participants without ultrasound dating but conducted a sensitivity analysis in which they were excluded.

4 For binary outcomes, values are RRs: LNS compared with IFA/SOC (95% CIs). Difference in events per 1000 was calculated using the prevalence difference (95% CI) and multiplying by 1000. For nearly all outcomes, the *I*^2^ value (heterogeneity) was 0, except for low LGAZ (*I*^2^ = 0.01 difference in events per 1000) and BMIZ (*I*^2^ = 0.30 for RR and difference in events per 1000). *I*^2^ describes the percentage of variability in effect estimates that may be due to heterogeneity rather than chance. Approximately 0.3–0.6 is considered moderate heterogeneity.

Most of the preplanned sensitivity analyses were not applicable to these 2 trials, except for the one restricting birth size outcomes to data collected within 72 h of birth. In that sensitivity analysis, there was little change in the results except that the MDs for head circumference (+0.21 cm; 95% CI: −0.03, 0.45 cm) and HCGAZ (+0.16; 95% CI: −0.01, 0.34) became larger.

Table 6 shows that there were no significant effects in the comparison between SQ-LNSs and MMSs on anthropometric outcomes at 6 mo of age. The findings were not altered when excluding the “simplified cohort follow-up” in Malawi. Table 7 shows no significant differences in adverse outcomes for the comparison between SQ-LNSs and MMSs.

**TABLE 6:** Main effects of maternal SQ-LNS compared with MMS on infant anthropometric outcomes at 6 mo of age.

Infant anthropometric outcomes at 6 mo	Number of participants (trials)	MD (95% CI)					*P* value^1^	Quality of the evidence (GRADE)
Continuous outcomes^2^								
Weight-for-age *z*-score (WAZ)	1292 (2)	−0.01 (−0.14, 0.11)					0.827	Low
Length-for-age *z*-score (LAZ)	1289 (2)	+0.02 (−0.10, 0.13)					0.792	Low
Weight-for-length *z*-score (WLZ)	1288 (2)	−0.04 (−0.16, 0.09)					0.551	Low
Head circumference-for-age *z*-score (HCZ)	1293 (2)	−0.03 (−0.13, 0.08)					0.636	Low
MUAC-for-age *z*-score (MUACZ)	1293 (2)	−0.02 (−0.14, 0.10)					0.727	Low

	Number of participants (trials)	SQ-LNS events per 1000	MMS events per 1000	PR (95% CI)	*P* value^1^	Difference in events per 1000 (95% CI)	*P* value^1^	Quality of the evidence (GRADE)
Binary outcomes^3^								
Underweight	1292 (2)	85	75	1.13 (0.78, 1.64)	0.510	9.5 (−20.2, 39.2)	0.531	Low
Stunted	1289 (2)	166	155	1.05 (0.83, 1.34)	0.673	13.7 (−25.0, 52.5)	0.487	Low
Wasted	1288 (2)	24	23	0.97 (0.48, 1.97)	0.943	0.2 (−16.4, 16.9)	0.977	Low
Low HCZ	1293 (2)	63	81	0.77 (0.52, 1.15)	0.203	−18.0 (−46.2, 10.2)	0.211	Low
Low MUACZ (MUACZ <−2 SD or MUAC <125 mm)	1293 (2)	66	74	0.92 (0.62, 1.36)	0.672	−6.8 (−34.6, 21.1)	0.633	Low
Acute malnutrition (WLZ <−2 SD or MUAC <125 mm)	1288 (2)	75	77	0.98 (0.67, 1.43)	0.918	−1.9 (−31.0, 27.2)	0.899	Low

Abbreviations: CI, confidence interval; GRADE, Grading of Recommendations Assessment, Development and Evaluation; HCZ, head circumference-for-age *z*-score; IFA, iron and folic acid supplement; LAZ, length-for-age *z*-score; LNS, lipid-based nutrient supplement; MD, mean difference; MUAC, mid-upper arm circumference; MMS, multiple micronutrient supplements; MUAC, mid-upper arm circumference; MUACZ, mid-upper arm circumference-for-age *z*-score; PR, prevalence ratio; SD, standard deviation; SQ-LNS, small-quantity lipid-based nutrient supplement; WAZ, weight-for-age *z*-score; WLZ, weight-for-length *z*-score.

1 The *P* value column corresponds to the pooled main effect 2-sided superiority testing of the intervention effect estimate and 95% CI presented in the preceding column.

2 For continuous outcomes, values are MDs: LNS – IFA/SOC (95% CIs). The *I*^2^ value (heterogeneity) was 0 for all outcomes. *I*^2^ describes the percentage of variability in effect estimates that may be due to heterogeneity rather than chance. Approximately 0.3–0.6 is considered moderate heterogeneity.

3 For binary outcomes, values are PRs: LNS compared with IFA/SOC (95% CIs). Difference in events per 1000 was calculated using the prevalence difference (95% CI) and multiplying by 1000. The *I*^2^ value (heterogeneity) was 0 for all outcomes. *I*^2^ describes the percentage of variability in effect estimates that may be due to heterogeneity rather than chance. Approximately 0.3–0.6 is considered moderate heterogeneity.

**TABLE 7:** Main effects of maternal SQ-LNS compared with MMS on adverse outcomes^1^.

Adverse outcomes	Number of participants (trials)	SQ-LNS events per 1000	IFA/SOC events per 1000	RR (95% CI)^2^	*P* value^3^	Heterogeneity (*I*^2^)^4^	Difference in events per 1000 (95% CI)^2^	*P* value^3^	Heterogeneity (*I*^2^)^4^
Cesarean section	1491 (2)	129	103	1.25 (0.94, 1.66)	0.125	0.00	27.0 (−2.1, 56.2)	0.069	0.00
Miscarriage	1566 (2)	16	16	1.00 (0.47, 2.13)	0.994	0.00	−0.1 (−11.3, 11.2)	0.991	0.00
Stillbirth	1566 (2)	21	11	1.57 (0.61, 4.03)	0.344	0.85	9.9 (−2.9, 22.7)	0.129	0.87
Miscarriage or stillbirth	1566 (2)	39	28	1.33 (0.76, 2.34)	0.320	0.79	14.7 (−2.9, 32.2)	0.102	0.73
Early neonatal mortality	1470 (2)	19	17	0.96 (0.44, 2.09)	0.924	0.61	4.0 (−9.1, 17.2)	0.550	0.59
Miscarriage or stillbirth or early neonatal mortality	1566 (2)	57	46	1.24 (0.81, 1.91)	0.321	0.00	11.3 (−10.7, 33.3)	0.313	0.00
Neonatal mortality	1473 (2)	23	19	1.05 (0.52, 2.10)	0.896	0.47	4.5 (−9.9, 19.0)	0.537	0.38
Mortality 0–6 mo	1483 (2)	30	28	1.00 (0.56, 1.78)	0.988	0.43	4.6 (−11.7, 20.9)	0.584	0.33

Abbreviations: CI, confidence interval; IFA, iron and folic acid supplement; LNS, lipid-based nutrient supplement; MMS, multiple micronutrient supplement; RR, relative risk; SOC, standard of care; SQ-LNS, small-quantity lipid-based nutrient supplement.

1 The quality of the evidence based on GRADE (Grading of Recommendations Assessment, Development and Evaluation) criteria was rated as low for all adverse outcomes.

2 Values are RRs: LNS compared with MMS (95% CIs). Difference in events per 1000 was calculated using the prevalence difference (95% CI) and multiplying by 1000.

3 The *P* value column corresponds to the pooled main effect 2-sided superiority testing of the intervention effect estimate and 95% CI presented in the preceding column.

4 *I*^2^ describes the percentage of variability in effect estimates that may be due to heterogeneity rather than chance. Approximately 0.3–0.6 is considered moderate heterogeneity.

#### Effect-measure modification by individual-level characteristics

For most characteristics, the *P*-for-interaction was >0.10 for all infant birth outcomes, i.e., effect modification was not evident for maternal height, BMI, education, or anemia at baseline, gestational age at start of supplementation, compliance with supplementation, household socioeconomic status, food insecurity, or sanitation (Supplemental Figure 6A–M). The *P*-for-interaction was <0.10 for ≥1 birth size outcome for 5 characteristics: child sex, birth order, maternal age, inflammation at baseline, and malaria at baseline. Effect modification estimates for maternal SQ-LNSs compared with MMSs were greater (*P*-for-interaction <0.10) among *1*) female (compared with male) infants for head circumference, HCZ, birth weight <2 kg, SGA, low BMIZ, and low HCZ (Supplemental Figures 6A1-2); *2*) first-born (compared with later-born) infants for birth weight and WAZ (Supplemental Figure 6B1); and *3*) younger participants (<25 compared with >25 y) for SGA (Supplemental Figure 6E). Effect modification by maternal inflammation was difficult to interpret; effect estimates in the comparison between maternal SQ-LNSs and MMSs were greater among participants with inflammation for birth weight, but greater among those without inflammation for HCZ and HCGAZ (Supplemental Figure 6H1). The results for effect modification by maternal malaria were not interpretable because they could not be confirmed in the sensitivity analysis restricted to anthropometric outcomes measured within 72 h of birth, due to insufficient sample sizes in the subgroup with malaria at enrollment. The results of the sensitivity analysis restricted to birth size measured within 72 h for effect modification by child sex, birth order, and maternal age are available at https://osf.io/nj5f9/ [17]; the effect estimates were similar in magnitude, but for some outcomes, the sample sizes in certain subgroups were insufficient to generate effect estimates.

Some of the above characteristics also modified the effect estimates for the comparison between maternal SQ-LNSs and MMSs with regard to a few of the anthropometric outcomes at 6 mo, but the results were not always consistent across outcomes (Supplemental Figure 6A3 and 4–6M3 and 4).

For most of the adverse outcomes, statistical power to examine effect modification was low because they are rare events, and the total sample size for this comparison, with both trials combined, was <1566.

## Discussion

In this IPD meta-analysis, data from 4 trials showed that maternal SQ-LNSs, compared with IFA or SOC, increased mean birth weight, length, head circumference, BMIZ, and MUAC and on average, reduced the incidence of LBW by 11%, newborn stunting by 17%, newborn wasting (low BMIZ) by 11%, and small head size (low HCZ) by 15%. Only 2 trials directly compared maternal SQ-LNSs and MMSs; birth outcomes did not differ significantly between these groups. Several individual-level characteristics appeared to modify the impact of maternal SQ-LNSs on certain birth outcomes. For the comparison with IFA or SOC, effect estimates for SQ-LNSs were greater among female infants (for multiple outcomes) and among participants with BMI <20 kg/m^2^ (for 2 outcomes), inflammation (for 3 outcomes), or malaria (for 4 outcomes) at enrollment or greater household food insecurity (for 4 outcomes). For the comparison with MMSs, effect estimates for SQ-LNSs were greater among female infants (for 5 outcomes), first-born infants (for 2 outcomes), and participants <25 y of age (for 3 outcomes). Some of these findings may have implications with regard to potential targeting of SQ-LNSs to vulnerable subgroups, as discussed below.

### Main effects on birth outcomes and adverse outcomes

For SQ-LNSs compared with IFA/SOC, our estimated main effects for birth outcomes are similar to those of Das et al. [13], which is expected because their analysis included 3 of the 4 trials evaluated in our IPD analysis. We report on several birth outcomes not included in the meta-analysis by Das et al. [13]: newborn BMIZ and low BMIZ and birth size for gestational age outcomes using the INTERGROWTH-21st standard. The MD in birth weight was 49 g, which is similar to the estimated impact of other types of BEP supplementation [10]. The RR of 0.89 for LBW translates into an estimated absolute difference of ∼29 events per 1000 births. For newborn stunting, wasting, and small head size, the estimated absolute differences are 32, 24, and 22 events per 1000 births, respectively. The effect estimates for birth size *z*-scores for gestational age (+0.11–0.13) were similar to those for WAZ, LAZ, and HCZ (+0.10–0.12), suggesting that the impact of SQ-LNSs on birth size was mainly attributable to improvements in fetal growth.

Regarding the comparison between SQ-LNSs and MMSs, we cannot directly compare our estimated main effects to those of Das et al. [13] because their analysis included 1 MQ-LNS trial in addition to the 2 SQ-LNS trials that are in our IPD meta-analysis. Although neither meta-analysis showed significant differences in birth outcomes between the (SQ-)LNS and MMS groups, the MDs for all of the birth size outcomes in our IPD analysis were in the same direction as those for the SQ-LNSs compared with IFA/SOC analysis, though generally of lower magnitude except for the MD in head circumference (which was almost identical between the SQ-LNS and MMS and SQ-LNS and IFA/SOC comparisons, e.g., HCGAZ +0.11 (95% CI: −0.01, 0.23) and +0.11 (95% CI: 0.02, 0.20), respectively.

Because only 2 trials have directly compared SQ-LNSs with MMSs, it is useful to examine how the estimated effects of SQ-LNSs versus IFA/SOC compare with those of MMSs versus IFA in previous meta-analyses that included a large number of trials [19,40]. Effects of MMSs compared with IFA on mean birth weight (+48 g; 95% CI: 40, 57 g) in Smith et al. [19] and RR for LBW (0.86–0.88) [19,40] are quite similar to those shown in the comparison between SQ-LNSs and IFA/SOC herein, and the results for SGA and preterm birth are also similar. However, we demonstrated positive effects of SQ-LNSs on newborn LAZ, stunting, HCZ, and small HCZ, whereas these outcomes have not been reported in the meta-analyses comparing MMSs and IFA. The MD for head circumference in the comparison between SQ-LNSs and MMSs suggests that the differences in nutrient composition between these 2 supplements (e.g., inclusion of EFAs and calcium in SQ-LNSs) may influence certain parameters of fetal growth, even if the effects on birth weight are similar.

In both the comparisons between SQ-LNSs and IFA/SOC and between SQ-LNSs and MMSs, we did not find significant differences in incidence of Cesarean section, miscarriage, stillbirths, early neonatal mortality, neonatal mortality, or mortality from birth to 6 mo of age, although it should be noted that the statistical power to detect differences in some of these rare outcomes was particularly limited.

### Effect-measure modification for birth outcomes

#### SQ-LNS compared with IFA/SOC

The impact of SQ-LNSs was greater among female than among male infants for many of the birth outcomes. Among females, effect estimates for SQ-LNSs suggested reductions of 20% for LBW, 24% for newborn stunting, 22% for newborn wasting, and 17% for small head size, and an increase in duration of gestation of +0.3 wk (95% CI: 0.15, 0.45 wk) (even when restricting the latter to pregnancies with ultrasound dating, +0.3 wk; 95% CI: 0.09, 0.51 wk). In exploratory analyses, we found that the effects on birth size were only partially explained by the increased duration of gestation, suggesting that among female fetuses, SQ-LNSs influenced both of the pathways leading to SVNs, i.e., “born too soon” and “born too small” [6]. It is noteworthy that child sex did not modify the effects of MMS (compared with that of IFA) on LBW or SGA, although it did for mortality (i.e., significant reductions in neonatal, 6-mo, and infant mortality among female but not male infants [19], mediated by duration of gestation and intrauterine growth in Tanzania [41]). It is unclear why the effects of SQ-LNSs were stronger among female fetuses. In our previous IPD meta-analysis of SQ-LNSs provided directly to infants and young children 6–23 mo of age, we also found a greater impact among females than among males for child stunting, wasting, and small head size [15] as well as anemia [42]. We interpreted this as a greater potential to respond to nutritional interventions among females than among males [15]. Males are more vulnerable to environmental stressors [43,44] and are at higher risk of morbidity and mortality in early life, which could constrain their response to nutrition interventions. Our results suggest that this vulnerability begins prior to birth and is thus likely to be biologically rather than socially driven.

For some outcomes, the effects of SQ-LNSs were greater among mothers with a low BMI at enrollment than among those with BMI ≥20 kg/m^2^. The *P*-for-interaction was significant only for low HCZ and mean MUAC, but effect estimates were also somewhat larger among low BMI mothers for most of the other birth size outcomes (though not for duration of gestation or preterm birth). Among mothers with low BMI, effect estimates for SQ-LNSs suggested reductions of 23% for newborn small head size and 20% for newborn stunting. This could be interpreted as a greater potential to benefit for infants of low BMI mothers.

Similarly, the effects of SQ-LNSs were greater among infants of mothers with inflammation or malaria at enrollment. For inflammation, the *P*-for-interaction was significant for WAZ, duration of gestation, and small head size, and there was a similar pattern for several other birth outcomes. Among mothers with inflammation, effect estimates for SQ-LNS suggested reductions of 33% for LBW, 31% for newborn stunting, 33% for newborn wasting, and 40% for small head size. For malaria, the *P*-for-interaction was significant for birth weight, WAZ, LBW, and SGA. Among mothers with malaria, effect estimates for SQ-LNS suggested reductions of 47% for LBW and 40% for SGA. These are large relative reductions in the risk of being “born too small,” and suggest that SQ-LNSs may be mitigating the adverse effects of maternal inflammation and possibly malaria on fetal growth [45,46].

The effects of SQ-LNSs were also greater among mothers in households with moderate-to-severe food insecurity than in those with less food insecurity. The *P*-for-interaction was significant for birth length, LAZ, newborn stunting, and head circumference (in centimeters), and there was a similar pattern for several other birth outcomes. Among mothers with greater food insecurity, effect estimates for SQ-LNSs suggested reductions of 16% for LBW and 25% for newborn stunting. These findings suggest a greater potential to benefit among mothers with food insecurity, perhaps because they are at greater risk of nutrient inadequacy.

The effects of SQ-LNSs on birth size outcomes tended to be greater among infants of mothers who consumed supplements >4 d/wk than in those with lower compliance, although the *P*-for-interaction was significant only for stunting (perhaps because relatively few participants had lower compliance, reducing statistical power for detecting effect modification). Among mothers with higher compliance, the effect estimates for SQ-LNSs suggested reductions of 16% for LBW, 22% for newborn stunting, 14% for newborn wasting, and 18% for small head size.

Effect modification was generally not evident for other maternal characteristics. This could be important, as it suggests that a response to maternal SQ-LNSs is not constrained by short maternal stature, low education, anemia at baseline, later gestational age at start of supplementation, or low household socioeconomic status. In the IPD meta-analysis of comparing MMSs and IFA [19], there was no impact of MMSs on SGA among mothers with low education, but there was among those with more education. This type of interaction was not observed for SQ-LNSs. In the studies of MMSs, education was categorized as none compared with ≥1 y, whereas our analysis of SQ-LNSs compared none/incomplete primary with greater than or equal to complete primary education. In the MMS meta-analysis, it is possible that mothers with no education were more likely to have diets lacking in some of the nutrients provided by SQ-LNSs but not MMSs. The lack of interaction of SQ-LNSs with maternal anemia is also noteworthy because the iron content of the SQ-LNS (20 mg) was much lower than that of IFA (60 mg) in these trials. Although this difference in iron content may influence the risk of maternal iron-deficiency anemia [[47], [48], [49]], it does not appear to compromise the impact of maternal SQ-LNSs on infant outcomes [50].

#### SQ-LNS compared with MMS

As was found for the comparison between SQ-LNSs and IFA/SOC, effect estimates for the comparison of SQ-LNSs and MMSs were greater among female than among male infants. The *P*-for-interaction with infant sex was significant for several outcomes (HCZ, SGA, birth weight <2 kg, low BMIZ, and small head size), and the effect estimates for SQ-LNSs were significant among females for LGAZ (+0.17; 95% CI: 0.00, 0.33), HCZ (+0.21; 95% CI: 0.01, 0.40), and HCGAZ (+0.19; 95% CI: 0.02, 0.37). It is noteworthy that these differences between intervention groups among females are for outcomes that reflect linear growth and head size and may be attributable to the differences in nutrient content between SQ-LNSs and MMSs, particularly EFAs (important for brain development) [51] and minerals such as calcium, potassium, and magnesium (important for linear growth).

Compared to the effects of MMSs, those of SQ-LNSs appeared to be greater among first-born than among later-born infants. The *P*-for-interaction was significant for birth weight and WAZ, and among first-born infants, the effect estimates for SQ-LNS were substantial for birth weight (+120 g; 95% CI; 35, 205 g) and HCZ (+0.31; 95% CI: 0.05, 0.58). However, there was heterogeneity between the 2 sites for this interaction, with results being driven by greater effects of SQ-LNSs among first-born infants in Ghana but not in Malawi.

The effects of SQ-LNSs on SGA were more beneficial among younger mothers than among those >25 y, and there was a similar pattern for other birth outcomes. Among infants of younger mothers, effect estimates comparing SQ-LNSs with MMSs were significant for birth weight (+69 g; 95% CI: 4, 135), WAZ (+0.16; 95% CI: 0.01, 0.31), and HCZ (+0.20; 95% CI: 0.00, 0.40). This could reflect a greater potential to benefit for infants of younger mothers (who are also more likely to be first-born), among whom improved intake of EFAs and certain minerals could be more critical.

### Effects on anthropometric status at 6 mo

An important biological and programmatic question is whether prenatal supplementation has a sustained impact on infant growth status after birth. Previous evidence on this question has been mixed [52]. In this meta-analysis, maternal SQ-LNSs (compared to IFA or SOC) reduced the prevalence of underweight at 6 mo of age by 15%, but effects on the other anthropometric outcomes were not statistically significant. Among female infants, underweight at 6 mo was reduced by 26%, whereas no effect was observed among male infants. In 3 cohorts (Bangladesh, Ghana, and half of the Malawi cohort), mothers continued to receive supplements after delivery for ≤6 mo, so it is possible that some of the impact on infant underweight at 6 mo is attributable to postpartum effects, e.g., through breast milk composition or maternal caregiving capacity. However, when we excluded the 2 cohorts in which mothers did not continue to receive supplements postpartum (Guatemala and half of the Malawi cohort), the effect estimate for underweight was weaker rather than stronger, suggesting that the reduction in underweight is likely related to SQ-LNSs received prenatally. This is an important finding because underweight among infants is a key risk factor for mortality [[53], [54], [55]].

There were no significant differences in infant anthropometric status at 6 mo in the comparison of SQ-LNSs with MMSs.

### Strengths and limitations

Strengths of this IPD meta-analysis include the high quality of the RCTs contributing to the estimates, data from diverse settings on 3 different continents, and the consistency in findings between fixed-effects and random-effects models as well as in most of the sensitivity analyses. Limitations include the relatively small number of trials (especially for the comparison between SQ-LNSs and MMSs), and limited statistical power to detect differences in rare outcomes. In one of the 4 trials, gestational age was not based on ultrasound dating but rather on the last menstrual period, which is less accurate; nonetheless, the results of the sensitivity analyses restricted to outcomes based on ultrasound dating were generally similar to those of the main analysis. The results of the effect modification analyses should be interpreted with caution because many of the potential effect modifiers are interrelated and may also be confounded by other unmeasured factors. In addition, there was variation in the methods used in each study to assess certain potential effect modifiers such as household food insecurity and socioeconomic status.

### Conclusions and implications

Maternal SQ-LNSs have substantial positive effects on birth outcomes when compared with IFA or SOC, especially among female infants and, for certain outcomes, among vulnerable mothers such as those with low BMI, inflammation, or malaria at enrollment or greater household food insecurity. Provision of SQ-LNSs during pregnancy may also reduce the prevalence of underweight among infants at 6 mo of age. An important programmatic question is whether maternal SQ-LNSs are superior to MMSs, which are lower in cost and are currently being scaled-up [56,57]. Based on this meta-analysis as well as the meta-analyses of MMSs [19,40], we conclude that SQ-LNSs and MMSs probably have similar positive effects on birth weight outcomes when comparing each with IFA. However, it is not known whether MMSs have an impact on newborn LAZ, stunting, BMIZ, wasting, HCZ, or small HCZ, all of which were improved in the comparisons between SQ-LNSs and IFA/SOC discussed herein. With only 2 trials directly comparing SQ-LNSs and MMSs, the statistical power to detect differences between intervention groups was considerably lower than was the case for the comparison between SQ-LNSs and IFA/SOC. Moreover, the MMS used in those 2 trials had 1.5–2 times the amounts of most micronutrients compared to the MMS formulation currently being scaled up (UNIMMAP), which may have reduced the likelihood of detecting differences in birth outcomes between the SQ-LNS and MMS groups. Thus, it is not known whether SQ-LNS has greater beneficial effects on birth outcomes relative to the UNIMMAP MMS formulation.

It is possible that the impact of SQ-LNSs on certain birth outcomes is superior to that of MMSs within vulnerable populations, even if the main effects on those outcomes do not differ significantly in the general population. For example, among infants of mothers <25 y of age, birth weight and head circumference were greater in the SQ-LNS group than the MMS group. The effects of MMSs (compared with those of IFA) on birth size (LBW or SGA) have not been shown to differ in younger mothers or those with low BMI [19]. The comparison of MMS with IFA IPD meta-analysis did not include household food insecurity or maternal inflammation as potential effect modifiers.

The large and consistent effect of SQ-LNSs (compared with that of IFA/SOC) among mothers with one or more biomarkers of inflammation at enrollment on several different metrics of fetal growth (ponderal, linear, and head size) is noteworthy, given that the percentage of participants in this subgroup was 40.8% in Ghana, 45.9% in Malawi, and 16.6% in Bangladesh (this information was not available for Guatemala). These findings suggest a greater potential to benefit from maternal consumption of SQ-LNSs among such mothers. The mechanisms underlying these findings require further investigation.

Further research is also needed to elucidate potential biological explanations for the stronger effects of maternal SQ-LNSs observed in female infants, compared with males, and how to overcome the constraints on the response in males. Regardless of the mechanism, the substantial reduction in risk of newborn stunting in females may have implications with regard to subsequent height during childhood, adolescence, and adulthood [58,59]. Greater maternal stature in females at the time of childbearing may reduce the risk of SGA and thereby help prevent the intergenerational transmission of impaired growth.

From a programmatic perspective, the effects among vulnerable subgroups demonstrated herein suggest that targeting provision of SQ-LNSs to younger mothers, those with low BMI, or those in households with food insecurity may be worth considering. This type of targeting is envisioned for BEP interventions in general [9], and the 2016 WHO guideline on antenatal care [60] already recommends prenatal BEP in populations with a high prevalence of undernutrition among pregnant females. Given that the SQ-LNS meets the definition of a BEP, our results support the strategy of targeting food-based supplements to pregnant females who have the greatest potential to benefit. SQ-LNSs provide less energy than most BEP supplements that have been evaluated, but at present, there is no clear dose-response relationship between the quantity of LNSs (or BEPs of other types) and birth outcomes [61]. Further research is needed to identify the optimal energy content of LNSs provided during pregnancy. However, it is noteworthy that the effects of SQ-LNSs (compared with those of IFA/SOC) on newborn stunting and head circumference in this IPD analysis were larger among mothers with greater food insecurity, despite the small amount of energy provided. This implies that improving intake of essential nutrients during pregnancy in high-risk populations is of paramount importance.

## Author contributions

The authors’ responsibilities were as follows – KGD: drafted the manuscript with input from KRW, CDA, CPS, and other coauthors; KRW, CDA, KGD, CPS: wrote the statistical analysis plan; BFA, PA, LH, NFK, JL, SM: reviewed, contributed to, and approved the statistical analysis plan; KRW, CDA: compiled the data; CDA: conducted the data analysis; KGD: responsible for final content; and all authors: read, contributed to, and approved the final manuscript.

## Funding

Supported by Bill & Melinda Gates Foundation grant OPP49817 (to KGD). The funder had no role in the design, implementation, analysis or interpretation of the data.

## Data availability

Data described in the manuscript, code book, and analytic code will not be made available because they are compiled from 4 different trials, and access is under the control of the investigators of each of those trials.

## Conflict of interest

N.F.K. is on the Editorial Board of the American Journal of Clinical Nutrition and played no role in the Journal’s evaluation of the manuscript.
