# Supplementary material for: Effects of prenatal small-quantity lipid-based nutrient supplements on pregnancy, birth, and infant outcomes: a systematic review and meta-analysis of individual participant data from randomized controlled trials in low- and middle-income countries
Source: Am J Clin Nutr. 2024 Aug 16;120(4):814–35. doi: 10.1016/j.ajcnut.2024.08.008 (PMC11473441; doi:10.1016/j.ajcnut.2024.08.008)
Supplement: Multimedia component 1 [file mmc1.zip › Maternal SQ-LNS Supplemental_2024-09-03/0_Maternal SQ-LNS Table of Contents.docx]

**Online Supporting Material**

Effects of prenatal small-quantity lipid-based nutrient supplements on pregnancy, birth and infant outcomes: a systematic review and meta-analysis of individual participant data from randomized controlled trials in low- and middle-income countries

Dewey *et al.* (2024)

**Table of Contents: Supplemental Methods**

- Supplemental Methods 1: Search strategies

**Table of Contents: Supplemental Tables**

- Supplemental Table 1. Composition of nutrient supplements
- Supplemental Table 2: Descriptive information on maternal, child and household characteristics at baseline, by trial
- Supplemental Table 3: Birth outcomes among IFA/SOC groups, by trial and pooled estimates
- Supplemental Table 4: Anthropometric outcomes at 6 mo among IFA/SOC groups, by trial and pooled estimates
- Supplemental Table 5: Adverse outcomes among IFA/SOC groups, by trial and pooled estimates
- Supplemental Table 6: Risk of bias assessment in each trial
- Supplemental Table 7A: Sensitivity analyses for main effects of SQ-LNS vs IFA/SOC on birth outcomes
- Supplemental Table 7B: Sensitivity analyses for main effects of SQ-LNS vs IFA/SOC on infant anthropometric outcomes at 6 mo of age
- Supplemental Table 7C: Sensitivity analyses for main effects of SQ-LNS vs IFA/SOC on adverse outcomes
- Supplemental Table 8: Sensitivity analyses for main effects of SQ-LNS vs IFA/SOC on birth outcomes among those with ultrasound data and among those with anthropometry within 72 h of birth

**Table of Contents: Supplemental Figures**

- Supplemental Figure 1: Summary risk of bias as a percentage of all included studies
- Supplemental figure 2: Forest plots of main effects for all outcomes, SQ-LNS vs IFA/SOC
- Supplemental figure 3: Pooled plots for infant outcomes at birth and at 6 mo, stratified by potential effect modifiers, SQ-LNS vs IFA/SOC
- Supplemental figure 4: Pooled plots for adverse outcomes stratified by potential effect modifiers, SQ-LNS vs IFA/SOC
- Supplemental figure 5: Forest plots of main effects for all birth outcomes, SQ-LNS vs MMS
- Supplemental figure 6: Pooled plots for infant outcomes at birth and at 6 mo, stratified by potential effect modifiers, SQ-LNS vs MMS
