# Supplementary material for: Effects of prenatal small-quantity lipid-based nutrient supplements on pregnancy, birth, and infant outcomes: a systematic review and meta-analysis of individual participant data from randomized controlled trials in low- and middle-income countries
Source: Am J Clin Nutr. 2024 Aug 16;120(4):814–35. doi: 10.1016/j.ajcnut.2024.08.008 (PMC11473441; doi:10.1016/j.ajcnut.2024.08.008)
Supplement: Multimedia component 1 [file mmc1.zip › Maternal SQ-LNS Supplemental_2024-09-03/1_Maternal SQ-LNS Supplemental Methods.docx]

# **Supplemental Methods 1: Search strategies**

We used the same search terms used by Das et al. (1) to search international and regional databases for studies published since that review was completed. Database of Abstracts of Review of Effect (DARE), Popline and IndMED were not included.

**Cochrane Central Register of Controlled Trials (CENTRAL) in the Cochrane Library**

#1[mh Lipids]

#2(fatty next acid*)

#3((Docosahexaenoic or Eicosapentaenoic) next acid*)

#4(PUFA or PUFAs)

#5lipid*

#6(omega next 3*)

#7(omega next 6*)

#8(soy* or peanut or groundnut or whey or sesame or cashew or chickpea or oil*)

#9{or #1-#8}

#10[mh “Dietary Supplements”]

#11[mh “Food, fortified”]

#12((diet* or food*) near/3 (fortif* or enrich* or supplement*))

#13(complement* near/3 (food* or feed*))

#14“Ready to use”

#15“point of use”

#16(RUSF or RUTF)

#17(home* near/2 fortif*)

#18{or #10-#17}

#19#9 and #18

#20(lipid next based)

#21(lipid* near/3 supplement*)

#22(lipid* near/3 nutrient*)

#23(lipid* near/3 fortif*)

#24(lipid* near/3 formulation*)

#54(lipid* near/3 enrich*)

#26(lipid* near/3 emuls*)

#27(lipid* near/3 powder*)

#28(lipid* near/3 spread*)

#29(lipid* near/3 paste*)

#30(Nutributter* or Plumpy*)

#31(LNS or iLiNS)

#32{or #20-#31}

#33#19 or #32

#34[mh Pregnancy]

#35[mh Pregnant Women]

#36[mh Prenatal Care]

#37[mh Prenatal Care]

#38(perinatal* or peri-natal* or prenatal* or pre-natal* or antenatal* or ante-natal*)

#39pregnan*

#40trimester*

#41[mh Mothers]

#42(mother* or maternal*)

#43{or #34-#42}

#44#33 and #43 in Trials

**MEDLINE Ovid (ALL), Strategy 1**

1 exp Lipids/

2 fatty acid$.tw,kf.

3 Docosahexaenoic acid$.tw,kf.

4 Eicosapentaenoic Acid$.tw,kf.

5 PUFA$.tw,kf.

6 lipid$.tw,kf.

7 (omega 3$ or omega 6$).tw,kf.

8 (soy$ or peanut or groundnut or whey or sesame or cashew or chickpea or oil$).tw,kf.

9 or/1-8

10 Dietary Supplements/

11 Food, fortified/

12 ((diet$ or food$) adj3 (fortif$ or enrich$ or supplement$)).tw,kf.

13 (complement$ adj3 (food$ or feed$)).tw,kf.

14 “Ready to use”.tw,kf.

15 (RUSF or RUTF).tw,kf.

16 “point of use”.tw,kf.

17 (home$ adj2 fortif$).tw,kf.

18 or/10-17

19 9 and 18

20 (lipid$ adj3 nutrient$).tw,kf.

21 (lipid$ adj3 supplement$).tw,kf.

22 lipid based.tw,kf.

23 (lipid$ adj3 fortif$).tw,kf.

24 (lipid$ adj3 enrich$).tw,kf.

25 (lipid$ adj2 emuls$).tw,kf.

26 (lipid$ adj2 formulation$).tw,kf.

27 (lipid$ adj3 powder$).tw,kf.

28 (lipid adj3 spread$).tw,kf.

29 (lipid$ adj3 paste$).tw,kf.

30 (Nutributter$ or Plumpy$).tw,kf.

31 (LNS$1 or iLiNS).tw,kf.

32 or/20-31

33 19 or 32

34 Pregnancy/

35 Pregnant women/

36 Prenatal care/

37 Perinatal care/

38 (perinatal$ or peri-natal$ or prenatal$ or pre-natal$ or antenatal$ or ante-natal$).tw.kf

39 prenan$.tw.kf

40 trimester$.tw.kf

41 Mothers/

42 (mother$ or maternal$).tw.kf

43 or/34-42

44 randomised controlled trial.pt

45 controlled clinical trial.pt

46 randomi#ed.ab

47 placebo$.ab

48 drug therapy.fs

49 randomly.ab

50 trial.ab

51 groups.ab

52 or/44-51

53 exp animals/ not humans.sh.

54 52 not 53

55 33 and 43 and 54

**MEDLINE Ovid (ALL), Strategy 2**

1 fatty acid$.tw,kf.

2 Docosahexaenoic acid$.tw,kf.

3 Eicosapentaenoic Acid$.tw,kf.

4 PUFA$.tw,kf.

5 lipid$.tw,kf.

6 (omega 3$ or omega 6$).tw,kf.

7 (soy$ or peanut or groundnut or whey or sesame or cashew or chickpea or oil$).tw,kf.

8 or/1-7

9 ((diet$ or food$) adj3 (fortif$ or enrich$ or supplement$)).tw,kf.

10 (complement$ adj3 (food$ or feed$)).tw,kf.

11 “Ready to use”.tw,kf.

12 (RUSF or RUTF).tw,kf.

13 “point of use”.tw,kf.

14 (home$ adj2 fortif$).tw,kf.

15 or/9-14

16 (lipid$ adj3 nutrient$).tw,kf.

17 (lipid$ adj3 supplement$).tw,kf.

18 lipid based.tw,kf.

19 (lipid$ adj3 fortif$).tw,kf.

20 (lipid$ adj3 enrich$).tw,kf.

21 (lipid$ adj2 emuls$).tw,kf.

22 (lipid$ adj2 formulation$).tw,kf.

23 (lipid$ adj3 powder$).tw,kf.

24 (lipid adj3 spread$).tw,kf.

25 (lipid$ adj3 paste$).tw,kf.

26 (Nutributter$ or Plumpy$).tw,kf.

27 (LNS$1 or iLiNS).tw,kf.

28 or/16-27

29 8 and (15 or 28)

30 (perinatal$ or peri-natal$ or prenatal$ or pre-natal$ or antenatal$ or ante-natal$).tw.kf

31 prenan$.tw.kf

32 trimester$.tw.kf

33 (mother$ or maternal$).tw.kf

34 or/30-33

35 29 and 34

36 (random$ or control$ or group$ or cluster$ or placebo$ or trial$ or assign$ or prospectiv$ or meta-analysis or systematic review or longitudinal$).tw,kf.

37 35 and 36

**Embase**

1 ‘lipid’/exp or lipid

2 ‘fatty acid*’:ti,ab,kw

3 ‘docosahexaenoic acid*’:ti,ab,kw

4 ‘eicosapentaenoic acid*’:ti,ab,kw

5 PUFA*:ti,ab,kw

6 lipid*:ti,ab,kw

7 ‘omega 3*’:ti,ab,kw or ‘omega 6*’:ti,ab,kw

8 soy*:ti,ab,kw or peanut:ti,ab,kw or groundnut:ti,ab,kw or whey:ti,ab,kw or sesame:ti,ab,kw or cashew:ti,ab,kw or chickpea:ti,ab,kw or oil*:ti,ab,kw

9 #1 or #2 or #3 or #4 or #5 or #6 or #7 or #8

10 ‘dietary supplement’:de

11 ‘fortified food’:de

12 ((diet* or food*) NEAR/3 (fortif* or enrich* or supplement*)):ti,ab,kw

13 (complement* NEAR/3 (food* or feed*)):ti,ab,kw

14 ‘Ready to use’:ti,ab,kw

15 RUSF:ti,ab,kw or RUTF:ti,ab,kw

16 ‘point of use’:ti,ab,kw

17 (home* NEAR/2 fortif*):ti,ab,kw

18 #10 or #11 or #12 or #13 or #14 or #15 or #16 or #17

19 #9 and #18

20 ‘lipid based’:ti,ab,kw

21 (lipid* NEAR/3 nutrient*):ti,ab,kw.

22 (lipid* NEAR/3 supplement*):ti,ab,kw.

23 (lipid* NEAR/3 fortif*):ti,ab,kw.

24 (lipid* NEAR/3 enrich*):ti,ab,kw.

25 (lipid* NEAR/2 emuls*):ti,ab,kw.

26 (lipid* NEAR/2 formulation*):ti,ab,kw.

27 (Lipid* NEAR/3 powder*):ti,ab,kw.

28 (lipid NEAR/3 spread*):ti,ab,kw.

29 (lipid* NEAR/3 paste*):ti,ab,kw.

30 Nutributter*:ti,ab,kw or Plumpy*:ti,ab,kw.

31 LNS:ti,ab,kw or iLiNS:ti,ab,kw.

32 #20 or #21 or #22 or #23 or #24 or #25 or #26 or #27 or #28 or #29 or #30 or #31

33 #19 or #32

34 ‘pregnancy’/exp

35 ‘prenatal care’/exp

36 ‘perinatal care’/exp

37 perinatal*:ti,ab,kw or ‘peri natal*’:ti,ab,kw or prenatal*:ti,ab,kw or ‘pre natal*’:ti,ab,kw or antenatal*:ti,ab,kw or ‘ante natal*’:ti,ab,kw

38 pregnan*:ti,ab,kw

39 trimester*:ti,ab,kw

40 ‘mother’/exp

41 mother*:ti,ab,kw or maternal*:ti,ab,kw

42 #34 or #35 or #36 or #37 or #38 or #39 or #40 or #41

#43 #33 and #42

#44 ‘animals’/exp or ‘invertebrate’/exp or ‘animal experiment’ or ‘animal model’ or ‘animal tissue’ or ‘animal cell’ or ‘nonhuman’

#45 ‘human’ or ‘normal human’ or ‘human cell’

#46 #44 and #45

#47 #44 not #46

#48 #43 not #47

#49 ‘randomized controlled trial’

#50 ‘controlled clinical trial’

#51 ‘single blind procedure’

#52 ‘double blind procedure’

#53 ‘triple blind procedure’

#54 ‘crossover procedure’

#55 crossover:ti,ab,kw or ‘cross over’:ti,ab,kw

#56 ((singl* or doubl* or tripl* or trebl*) NEAR/1 (blind* or mask*)):ti,ab,kw

#57 ‘placebo’

#58 ‘placebo’:ti,ab,kw

#59 ‘prospetive’:ti,ab,kw

#60 ‘factorial*’:ti,ab,kw

#61 ‘random*’:ti,ab,kw

#62 ‘assign*’:ti,ab,kw

#63 ‘allocat*’:ti,ab,kw

#64 ‘volunteer*’:ti,ab,kw

#65 #49 or #50 or #51 or #52 or #53 or #54 or #55 or #56 or #57 or #58 or #59 or #60 or #61 or #62 or #63 or #64

#66 #48 and #65

**CINAHL Plus EBSCOhost (Cumulative Index to Nursing and Allied Health Literature)**

S1 MH “Lipids+”

S2 TI (lipid*) or AB (lipid*)

S3 TI(Docosahexaenoic acid*) OR AB(Docosahexaenoic acid*)

S4 TI(Eicosapentaenoic acid*) OR AB( Eicosapentaenoic acid*)

S5 TI(PUFA*) OR AB(PUFA* )

S6 TI(omega 3* or omega 6*) OR AB(omega 3* or omega 6* )

S7 TI (soy* or peanut or groundnut or whey or sesame or cashew or chickpea or oil*) or AB(soy* or peanut or groundnut or whey or sesame or cashew or chickpea or oil*)

S8 TI(fatty acid*) OR AB(fatty acid* )

S9 S1 OR S2 OR S3 OR S4 OR S5 OR S6 OR S7 OR S8

S10 MH “Dietary Supplements”

S11 MH “Dietary Supplementation”

S12 MH “Food, Fortified”

S13 TI ((diet* or food*) n3 (fortif* or enrich* or supplement*)) OR AB((diet* or food*) n3 (fortif* or enrich* or supplement*))

S14 TI (complement* n3 (food* or feed*)) or AB (complement* n3 (food* or feed*))

S15 “Ready to use”

S16 (RUSF or RUTF)

S17 “point of use”

S18 TI (home* n2 fortif*) OR AB(home* n2 fortif*)

S19 S10 OR S11 OR S12 OR S13 OR S14 OR S15 OR S16 OR S17 OR S18

S20 S9 AND S19

S21 TI (lipid based) or AB (lipid based)

S22 TI(lipid* N3 supplement*) OR AB( lipid* N3 supplement*)

S23 TI(lipid* N3 nutrient*) OR AB(lipid* N3 nutrient*)

S24 TI(lipid* N3 fortif*) OR AB (lipid* N3 fortif*)

S25 TI(lipid* N3 formulation*) OR AB(lipid* N3 formulation*)

S26 TI(lipid* N3 enrich*) OR AB(lipid* N3 enrich* )

S27 TI(lipid* N3 emuls*) OR AB(lipid* N3 emuls*)

S28 TI(lipid* N3 powder*) OR AB(lipid* N3 powder*)

S29 TI(lipid N3 spread*) OR AB(lipid N3 spread*)

S30 TI(lipid* N3 paste*) OR AB(lipid* N3 paste*)

S31 TI(Nutributter* or Plumpy*) or AB(Nutributter* or Plumpy*)

S32 Nutributter* or Plumpy*

S33 TI(LNS or iLiNS) OR AB( LNS or iLiNS)

S34 S21 OR S22 OR S23 OR S24 OR S25 OR S26 OR S27 OR S28 OR S29 OR S30 OR S31 OR S32 OR S33

S35 S20 OR S34

S36 MH “Pregnancy+”

S37 MH “Pregnancy Trimesters+”

S38 MH “Prenatal Care”

S39 MH “Perinatal Care”

S40 TI(perinatal* or peri-natal* or prenatal* or pre-natal* or antenatal* or ante-natal*) OR AB(perinatal* or peri-natal* or prenatal* or pre-natal* or antenatal* or ante-natal*)

S41 pregnan*

S42 trimester*

S43 MH “Mothers”

S44 TI(mother* or maternal*) or AB(mother* or maternal*)

S45 S36 or S37 or S38 or S39 or S40 or S41 or S42 or S43 or S44

S46 S35 and S45

S47 MH “Clinical Trials+”

S48 MH random assignment

S49 MH “meta-analysis”

S50 MH “crossover design”

S51 MH “Quantitative studies”

S52 PT randomized controlled trial

S53 PT clinical trial

S54 (trial* or control* or placebo*)

S55 (“follow-up study” or “follow-up research*)

S56 (prospective* study or prospect* research)

S57 (evaluat* N2 study or evaluat* N2 research)

S58 MH “Program Evaluation”

S59 MH “Treatment Outcomes”

S60 TI(single N2 mask* or single N2 blind*) OR AB(single N2 mask* or single N2 blind*)

S61 TI(doubl* N2 mask* or doubl* N2 blind*) OR AB(doubl* N2 mask* or doubl* N2 blind*)

S62 TI(tripl* N2 mask* or tripl* N2 blind or trebl* N2 mask or trebl* N2 blind) or AB(tripl* N2 mask* or tripl* N2 blind or trebl* N2 mask or trebl* N2 blind)

S63 random*

S64 S47 or S48 or S49 or S50 or S51 or S52 or S53 or S54 or S55 or S56 or S57 or S58 or S59 or S60 or S61 or S62 or S63

S65 S46 and S64

**Web of Science: Science Citation Index (SCI), Social Sciences Citation Index (SSCI), Conference Proceedings Citation Index - Science (CPCI-S) and Conference Proceedings Citation Index -Social Science & Humanities (CPCI-SS&H)**

TS=((lipid* OR "fatty acid*" OR ((Docosahexaenoic or Eicosapentaenoic) next acid*) or PUFA OR PUFAs OR "omega 3*" OR "omega 6*" OR soy* OR peanut* OR groundnut* OR whey* OR sesame* OR cashew* OR chickpea* OR oil*))

TS=(((diet* or food*) near/3 (fortif* or enrich* or supplement*)))

TS=((complement* near/3 (food* or feed*)))

TS=(("Ready to use" or "point of use" or RUSF or RUTF or (home* near/2 fortif*)))

#2 or #3 or #4

#1 and #5

TS=(("lipid based"))

TS=((lipid* near/3 (supplement* or nutrient* or fortif* or formulation* or enrich* or emuls* or powder* or spread* or paste*)))

TS=((Nutributter* or Plumpy* OR LNS or iLiNS))

#7 or #8 or #9

#6 or #10

TS=((perinatal* or peri-natal* or prenatal or pre-natal* or antenatal* or ante-natal* or pregnan* or trimester*))

TS=((mother* or maternal*))

#12 or #13

#11 and #14

TS=((RANDOM* OR TRIAL* OR CONTROL* OR PLACEBO* OR PROSPECTIV* OR LONGITUDINAL OR BLIND* OR GROUP* OR CLUSTER* OR meta-analysis OR systematic review))

#15 and #16

TS=((RATS OR MICE OR SHEEP OR PIGS OR COWS OR CHICKS OR CHICKENS OR DUCK*))

#17 not #18

**Cochrane Database of Systematic Reviews (CDSR), part of the Cochrane Library**

#1[mh Lipids]

#2 (fatty next acid*):ti,ab

#3((Docosahexaenoic or Eicosapentaenoic) next acid*):ti,ab

#4(PUFA or PUFAs):ti,ab

#5lipid*:ti,ab

#6(omega next (3* or 6*)):ti,ab

#7(soy* or peanut or groundnut or whey or sesame or cashew or chickpea or oil*):ti,ab

#8{or #1-#7}

#9[mh “Dietary Supplements”]

#10[mh “Food, fortified”]

#11((diet* or food*) near/3 (fortif* or enrich* or supplement*)):ti,ab

#12(complement* near/3 (food* or feed*)):ti,ab

#13“Ready to use”:ti,ab

#14“point of use”:ti,ab

#15(RUSF or RUTF):ti,ab

#16(home* near/2 fortif*):ti,ab

#17(2-#16)

#18#8 and #17

#19(lipid next based):ti,ab

#20(lipid* near/3 supplement*):ti,ab

#21(lipid* near/3 nutrient*):ti,ab

#22(lipid* near/3 fortif*):ti,ab

#23(lipid* near/3 formulation*):ti,ab

#24(lipid* near/3 enrich*):ti,ab

#25(lipid* near/3 emuls*):ti,ab

#26(lipid* near/3 powder*):ti,ab

#27(lipid* near/3 spread*):ti,ab

#28(lipid* near/3 paste*):ti,ab

#29(Nutributter* or Plumpy*):ti,ab

#30(LNS* or iLiNS):ti,ab

#31{or #19-#30}

#32#18 or #31

#33[mh “Pregnancy”]

#34[mh “Pregnant Women”]

#35[mh “Prenatal Care”]

#36[mh “Perinatal Care”]

#37(perinatal* or peri-natal* or prenatal* or pre-natal* or antenatal* or ante-natal*):ti,ab

#38pregnan*:ti,ab

#39trimester*:ti,ab

#40[mh “Mothers”]

#41(mother* or maternal*):ti,ab

#42{or #33-#41}

**Epistemonikos (epistemonikos.org)**

(title:((title:(LIPID* OR FATTY ACID* OR OMEGA OR Docosahexaenoic OR Eicosapentaenoic OR soy* OR peanut OR groundnut OR whey OR sesame OR cashew OR chickpea OR oil*) OR abstract:(LIPID* OR FATTY ACID* OR OMEGA OR Docosahexaenoic OR Eicosapentaenoic OR soy* OR peanut OR groundnut OR whey OR sesame OR cashew OR chickpea OR oil*))) OR abstract:((title:(LIPID* OR FATTY ACID* OR OMEGA OR Docosahexaenoic OR Eicosapentaenoic OR soy* OR peanut OR groundnut OR whey OR sesame OR cashew OR chickpea OR oil*) OR abstract:(LIPID* OR FATTY ACID* OR OMEGA OR Docosahexaenoic OR Eicosapentaenoic OR soy* OR peanut OR groundnut OR whey OR sesame OR cashew OR chickpea OR oil*)))) AND (title:(fortif* OR enrich* OR supplement* OR "Ready to use" OR "point of use" OR RUSF OR RUTF) OR abstract:(fortif* OR enrich* OR supplement* OR "Ready to use" OR "point of use" OR RUSF OR RUTF)) AND (title:(PREGNAN* OR perinatal* OR peri-natal* OR prenatal* OR pre-natal* OR antenatal* OR ante-natal* OR trimester* OR MOTHER* OR MATERNAL*) OR abstract:(PREGNAN* OR perinatal* OR peri-natal* OR prenatal* OR pre-natal* OR antenatal* OR ante-natal* OR trimester* OR MOTHER* OR MATERNAL*))

**ClinicalTrials.gov (clinicaltrials.gov)**

CONDITION| pregnancy OR prenatal OR pre-natal OR antenatal OR ante-natal OR perinatal OR peri-natal OR mothers OR maternal

AND

INTERVENTION| lipid OR lipid-based OR LNS OR iLiNS OR nutrient supplement OR fortification OR fortified OR “ready to use” OR “point of use” OR RUSF OR RUTF OR “therapeutic food” OR paste OR spread OR blend OR Nutributter OR Plumpynut

**World Health Organization International Clinical Trials Registry Platform (WHO ICTRP; who.int/trialsearch)**

CONDITION| pregnancy OR prenatal OR pre-natal OR antenatal OR ante-natal OR perinatal OR peri-natal OR mothers OR maternal

AND

INTERVENTION| lipid OR lipid-based OR LNS OR iLiNS OR nutrient supplement OR fortification OR fortified OR “ready to use” OR “point of use” OR RUSF OR RUTF OR “therapeutic food” OR paste OR spread OR blend OR Nutributter OR Plumpynut

AND

RECRUITMENT STATUS|All

**IBECS (Índice Bibliográfico Español en Ciencias de la Salud; ibecs.isciii.es)**

WORD| lipid OR lipid-based OR LNS OR iLiNS OR nutrient supplement OR fortification OR fortified OR “ready to use” OR “point of use” OR RUSF OR RUTF OR “therapeutic food” OR paste OR spread OR blend OR Nutributter OR Plumpy OR Plumpynut

AND

WORD| pregnancy OR prenatal OR pre-natal OR antenatal OR ante-natal OR perinatal OR peri-natal OR mothers OR maternal

**SciELO (Scientific Electronic Library Online; www.scielo.br)**

(lipid or lipid-based OR LNS OR iLiNS OR nutrient supplement OR fortification OR fortified OR "ready to use" OR "point of use" OR RUSF OR RUTF OR "therapeutic food" OR paste OR spread OR blend OR nutributter OR Plumpy OR PLUMPYNUT) AND (pregnancy OR prenatal OR pre-natal OR antenatal OR ante-natal OR perinatal OR peri-natal OR mothers OR maternal)

**AIM (Africa Index Medicus; search.bvsalud.org/ghl/?lang=en&submit=Search&where=REGIONAL)**

(tw:(lipid OR lipid-based OR LNS OR iLiNS OR nutrient supplement OR fortification OR fortified OR "ready to use" OR "point of use" OR RUSF OR RUTF OR "therapeutic food" OR paste OR spread OR blend OR nutributter OR Plumpy OR PLUMPYNUT)) AND (tw:(pregnancy OR prenatal OR pre-natal OR antenatal OR ante-natal OR perinatal OR peri-natal OR mothers OR maternal))

**IMEMR (Index Medicus for the Eastern Mediterranean Region;**

**search.bvsalud.org/ghl/?lang=en&submit=Search&where=REGIONAL)**

(tw:(lipid OR lipid-based OR LNS OR iLiNS OR nutrient supplement OR fortification OR fortified OR "ready to use" OR "point of use" OR RUSF OR RUTF OR "therapeutic food" OR paste OR spread OR blend OR nutributter OR Plumpy OR PLUMPYNUT)) AND (tw:(pregnancy OR prenatal OR pre-natal OR antenatal OR ante-natal OR perinatal OR peri-natal OR mothers OR maternal))

**LILACS (Latin American and Caribbean Health Sciences Literature; lilacs.bvsalud.org/en)**

(tw:(lipid OR lipid-based OR LNS OR iLiNS OR nutrient supplement OR fortification OR fortified OR "ready to use" OR "point of use" OR RUSF OR RUTF OR "therapeutic food" OR paste OR spread OR blend OR nutributter OR Plumpy OR PLUMPYNUT)) AND (tw:(pregnancy OR prenatal OR pre-natal OR antenatal OR ante-natal OR perinatal OR peri-natal OR mothers OR maternal))

**PAHO/WHO Institutional Repository for Information Sharing (iris.paho.org/xmlui)**

(lipid or lipid-based OR LNS OR iLiNS OR nutrient supplement OR fortification OR fortified OR "ready to use" OR "point of use" OR RUSF OR RUTF OR "therapeutic food" OR paste OR spread OR blend OR nutributter OR Plumpy OR PLUMPYNUT) AND (pregnancy OR prenatal OR pre-natal OR antenatal OR ante-natal OR perinatal OR peri-natal OR mothers OR maternal)

**WPRIM (Western Pacific Index Medicus;**

**search.bvsalud.org/ghl/?lang=en&submit=Search&where=REGIONAL)**

(tw:(lipid OR lipid-based OR LNS OR iLiNS OR nutrient supplement OR fortification OR fortified OR "ready to use" OR "point of use" OR RUSF OR RUTF OR "therapeutic food" OR paste OR spread OR blend OR nutributter OR Plumpy OR PLUMPYNUT)) AND (tw:(pregnancy OR prenatal OR pre-natal OR antenatal OR ante-natal OR perinatal OR peri-natal OR mothers OR maternal))

**IMSEAR (Index Medicus for the South-East Asian Region;**

**search.bvsalud.org/ghl/?lang=en&submit=Search&where=REGIONAL)**

(tw:(lipid OR lipid-based OR LNS OR iLiNS OR nutrient supplement OR fortification OR fortified OR "ready to use" OR "point of use" OR RUSF OR RUTF OR "therapeutic food" OR paste OR spread OR blend OR nutributter OR Plumpy OR PLUMPYNUT)) AND (tw:(pregnancy OR prenatal OR pre-natal OR antenatal OR ante-natal OR perinatal OR peri-natal OR mothers OR maternal))

**Native Health Research Database (nativehealthdatabase.net)**

Keywords: (Supplement AND pregnancy)

**REFERENCES**

1. Das J.K., Hoodbhoy Z., Salam R.A., Bhutta A.Z., Valenzuela-Rubio N.G., Weise Prinzo Z., Bhutta Z.A. Lipid-based nutrient supplements for maternal, birth, and infant developmental outcomes. Cochrane Database of Systematic Reviews 2018;8:CD012610.
