# Supplementary material for: Effects of prenatal small-quantity lipid-based nutrient supplements on pregnancy, birth, and infant outcomes: a systematic review and meta-analysis of individual participant data from randomized controlled trials in low- and middle-income countries
Source: Am J Clin Nutr. 2024 Aug 16;120(4):814–35. doi: 10.1016/j.ajcnut.2024.08.008 (PMC11473441; doi:10.1016/j.ajcnut.2024.08.008)

Supplemental figure 5: Forest plots of main effects for all birth outcomes, SQ-LNS vs MMS

Contents

Supplemental figure 5A: Mean difference in birth weight (g) 3

Supplemental figure 5B: Mean difference in weight-for-age z score 4

Supplemental figure 5C: Mean difference in weight-for-gestational age z-score 5

Supplemental figure 5D: Low birth weight relative risk 6

Supplemental figure 5E: Low birth weight risk difference 7

Supplemental figure 5F: Birth weight < 2 kg relative risk 8

Supplemental figure 5G: Birth weight < 2 kg risk difference 9

Supplemental figure 5H: Small-for-gestational age relative risk 10

Supplemental figure 5I: Small-for-gestational age risk difference 11

Supplemental figure 5J: Large-for-gestational age relative risk 12

Supplemental figure 5K: Large-for-gestational age risk difference 13

Supplemental figure 5L: Mean difference in birth length (cm) 14

Supplemental figure 5M: Mean difference in length-for-age z score 15

Supplemental figure 5N: Mean difference in length-for-gestational age z-score 16

Supplemental figure 5O: Newborn stunting relative risk **17**

Supplemental figure 5P: Newborn stunting risk difference **18**

Supplemental figure 5Q: Low LGAZ relative risk **19**

Supplemental figure 5R: Low LGAZ risk difference **20**

Supplemental figure 5S: Mean difference in BMI-for-age z-score **21**

Supplemental figure 5T: Low BMIZ relative risk **22**

Supplemental figure 5U: Low BMIZ risk difference **23**

Supplemental figure 5V: Mean difference in head circumference (cm) **24**

Supplemental figure 5W: Mean difference in head circumference-for-age z score **25**

Supplemental figure 5X: Mean difference in head circumference-for-gestational age z score **26**

Supplemental figure 5Y: Low HCZ relative risk **27**

Supplemental figure 5Z: Low HCZ risk difference **28**

Supplemental figure 5AA: Low HCGAZ relative risk **29**

Supplemental figure 5AB: Low HCGAZ risk difference **30**

Supplemental figure 5AC: Mean difference in mid-upper arm circumference (cm) **31**

Supplemental figure 5AD: Mean difference in duration of gestation (wk) **32**

Supplemental figure 5AE: Preterm birth relative risk **33**

Supplemental figure 5AF: Preterm birth risk difference **34**

These figures are forest plots showing the study-level estimates of intervention effect with the pooled estimate in the bottom summary rows. Individual study estimates were generated from log binomial regression for dichotomous outcomes and simple linear regression for continuous outcomes with clustered observations using robust standard errors for cluster-randomized trials. Pooled estimates were generated using inverse variance weighting in both fixed and random effects models. For continuous outcomes the intervention effect is measured by the difference in mean of the SQ-LNS group minus MMS. For dichotomous outcomes analyzed via prevalence/risk ratios the effect estimate is the prevalence/risk in the SQ-LNS group divided by the prevalence/risk in the MMS group. For dichotomous outcomes analyzed via prevalence/risk differences the effect estimate is the prevalence/risk in the SQ-LNS group minus the prevalence/risk in the MMS group. The labels on the left y-axis correspond to trial level information. The values on the right indicate the study level effect estimate, confidence interval, and weighting for deriving the pooled estimate.

LAZ, length-for-age z-score; WLZ, weight-for-length z-score; WAZ, weight for-age z-score; MUACZ, mid-upper arm circumference z-score; BMI, body mass index; HCZ, head circumference-for-age z-score; LGAZ, length-for-gestational-age z-score; HCGAZ, head circumference-for-gestational-age z-score; BMIZ, body mass index-for-age z-score; IFA/SOC, Iron and folic acid or standard of care; MD, mean difference; MMS, multiple micronutrient supplement; MUAC, mid-upper arm circumference; PR, prevalence ratio; PD, prevalence difference; RD, risk difference; RR, relative risk; SOC, standard of care; SQ-LNS, small-quantity lipid-based nutrient supplements; WGAZ, weight-for-gestational age z-score.

Supplemental figure 5A: Mean difference in birth weight (g)

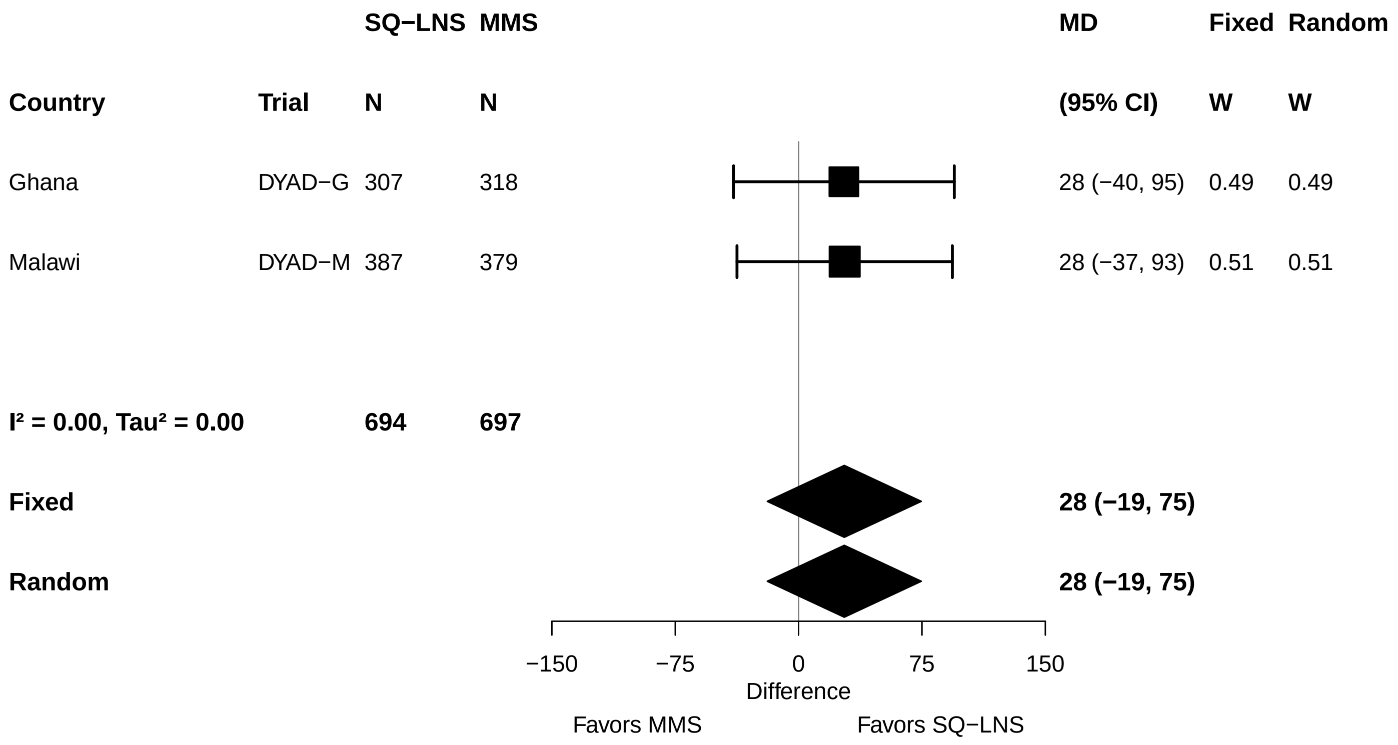

Supplemental figure 5B: Mean difference in weight-for-age z score

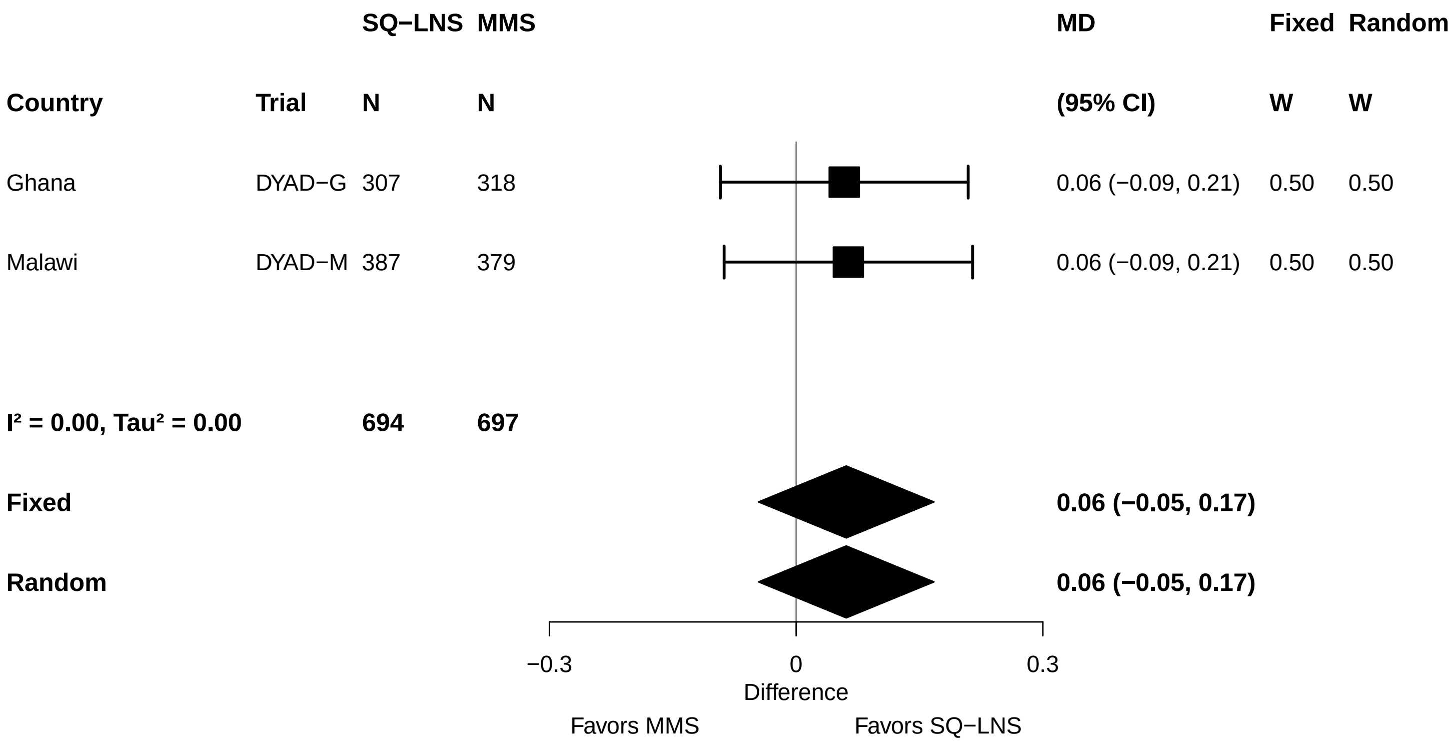

Supplemental figure 5C: Mean difference in weight-for-gestational age z-score

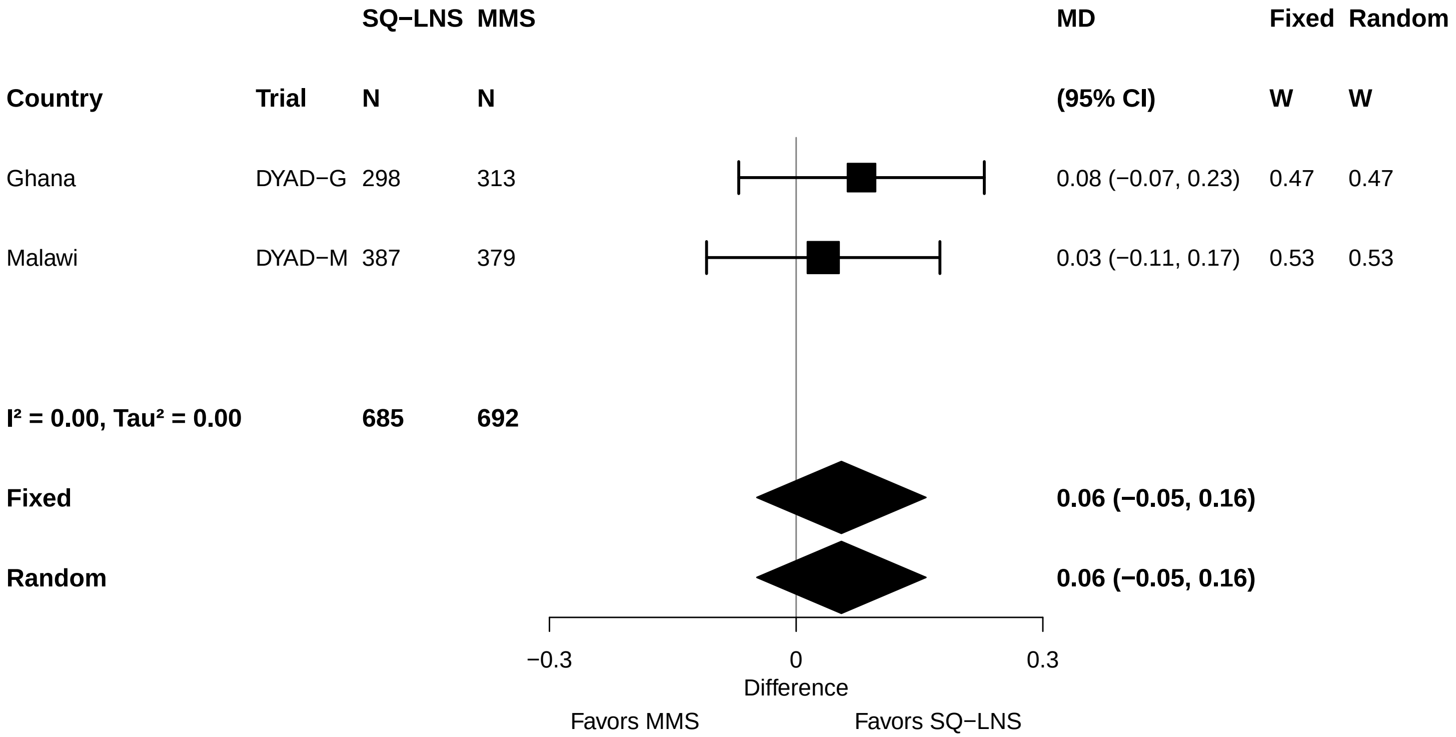

Supplemental figure 5D: Low birth weight relative risk

Country	Trial	SQ−LNS N	MMS N	RR (95% CI)	Fixed W	Random W
Ghana	DYAD−G	307	318	0.87 (0.53, 1.43)	0.34	0.34
Malawi	DYAD−M	387	379	0.89 (0.62, 1.27)	0.66	0.66
**I² = 0.00, Tau² = 0.00**		**694**	**697**			
**Fixed**				**0.88 (0.66, 1.18)**		
**Random**				**0.88 (0.66, 1.18)**		

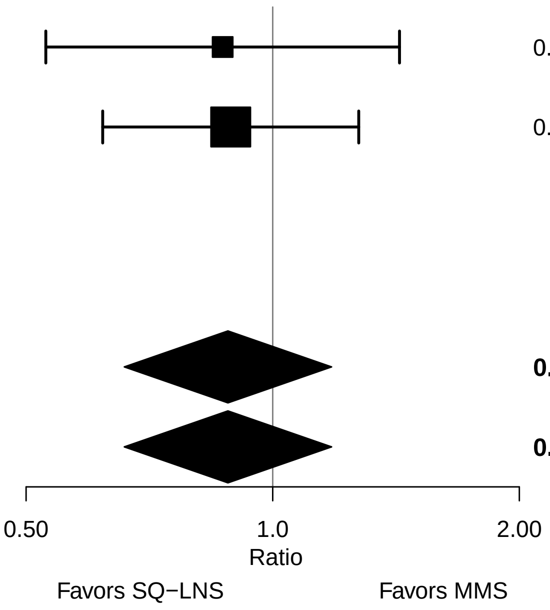

Supplemental figure 5E: Low birth weight risk difference

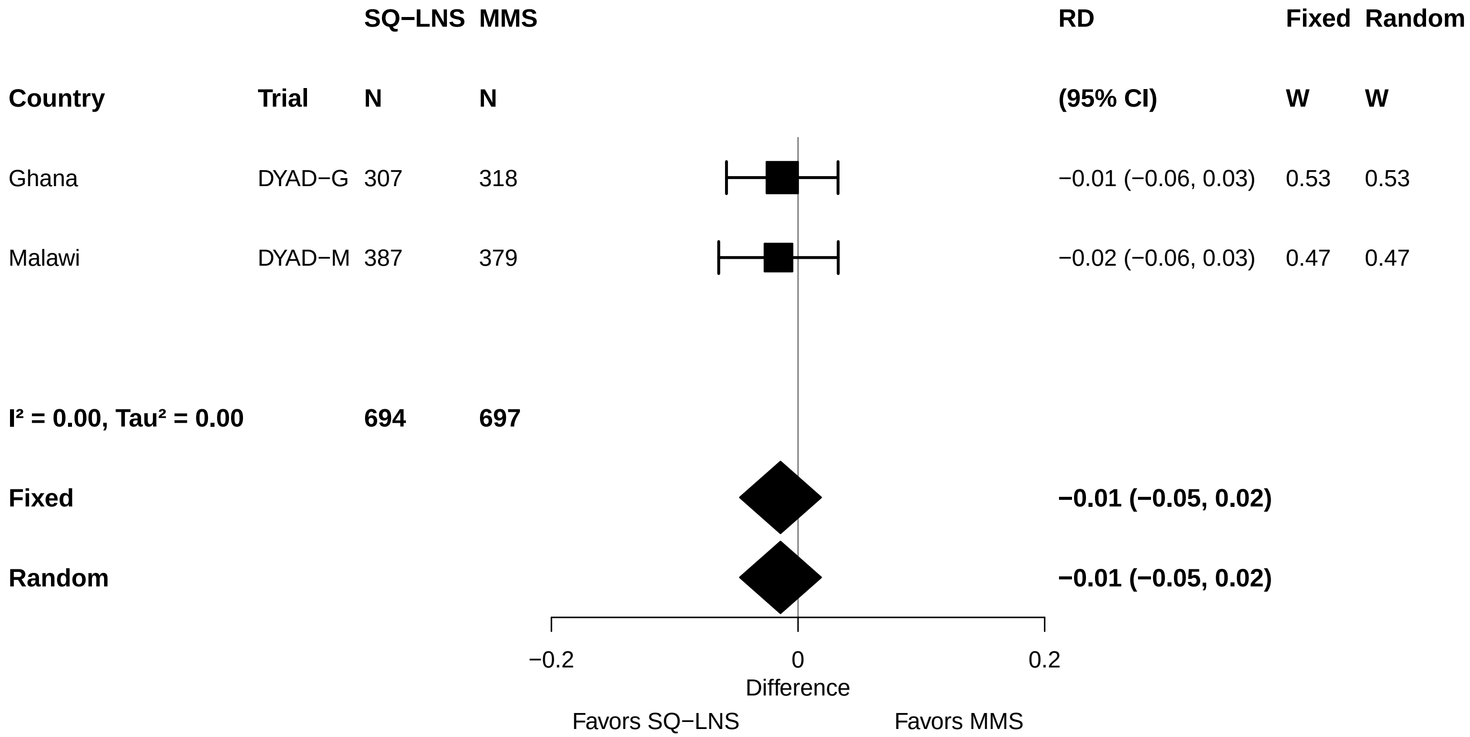

Supplemental figure 5F: Birth weight < 2 kg relative risk

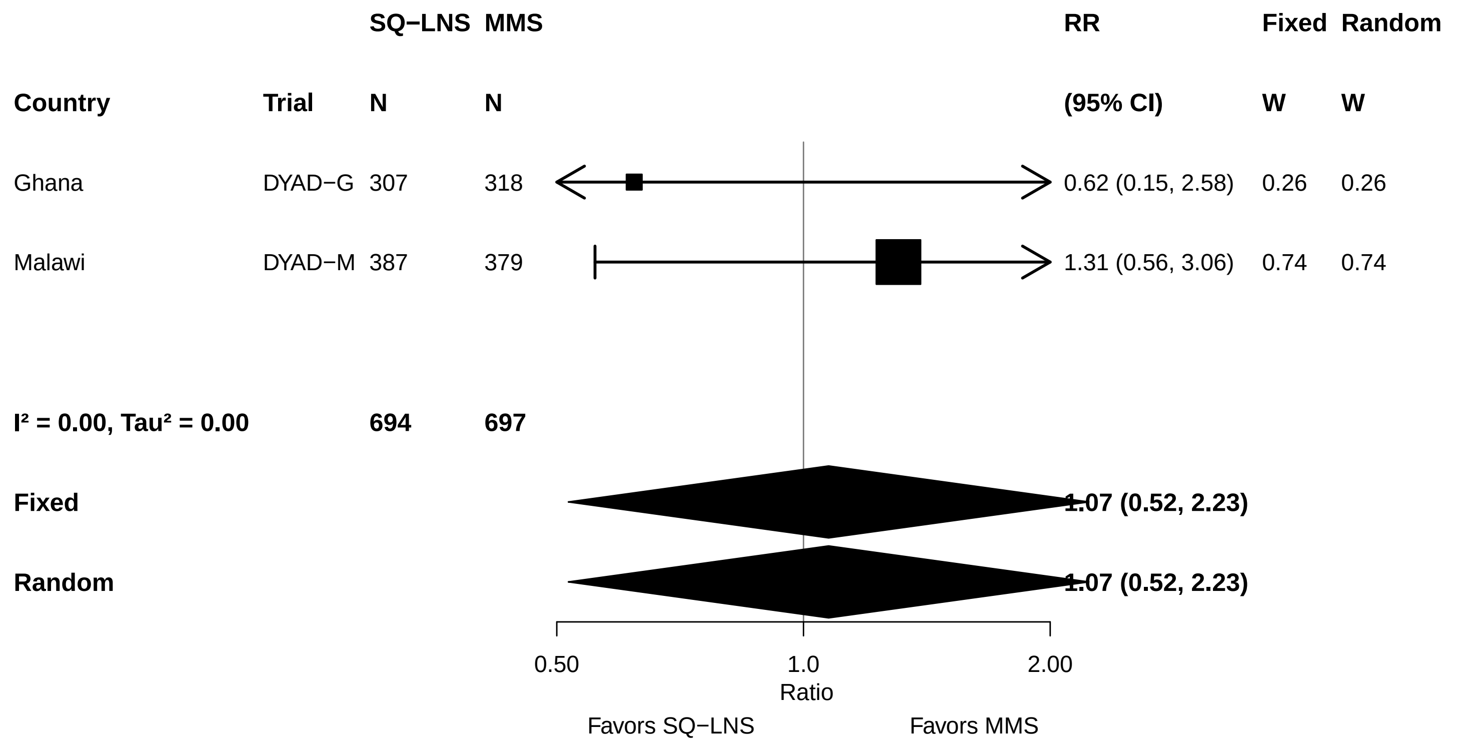

Supplemental figure 5G: Birth weight < 2 kg risk difference

Country	Trial	SQ−LNS N	MMS N	RD (95% CI)	Fixed W	Random W
Ghana	DYAD−G	307	318	−0.01 (−0.02, 0.01)	0.63	0.63
Malawi	DYAD−M	387	379	0.01 (−0.02, 0.03)	0.37	0.37
**I² = 0.00, Tau² = 0.00**		**694**	**697**			
**Fixed**				**0.00 (−0.02, 0.01)**		
**Random**				**0.00 (−0.02, 0.01)**		

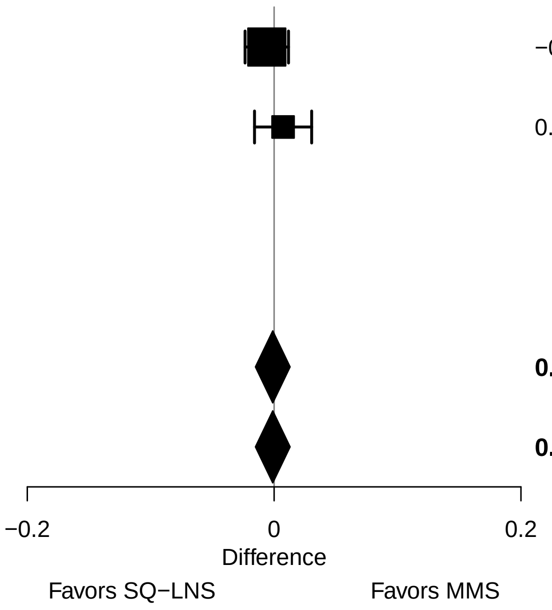

Supplemental figure 5H: Small-for-gestational age relative risk

Country	Trial	SQ−LNS N	MMS N	RR (95% CI)	Fixed W	Random W
Ghana	DYAD−G	307	318	0.91 (0.66, 1.25)	0.37	0.37
Malawi	DYAD−M	387	379	1.00 (0.78, 1.28)	0.63	0.63
**I² = 0.00, Tau² = 0.00**		**694**	**697**			
**Fixed**				**0.96 (0.79, 1.17)**		
**Random**				**0.96 (0.79, 1.17)**		

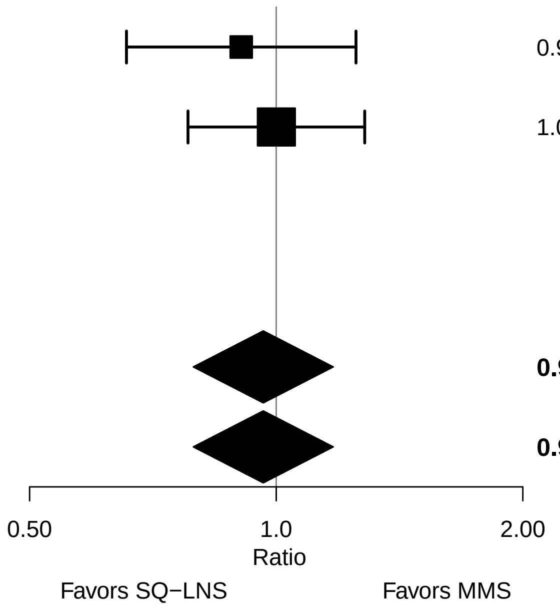

Supplemental figure 5I: Small-for-gestational age risk difference

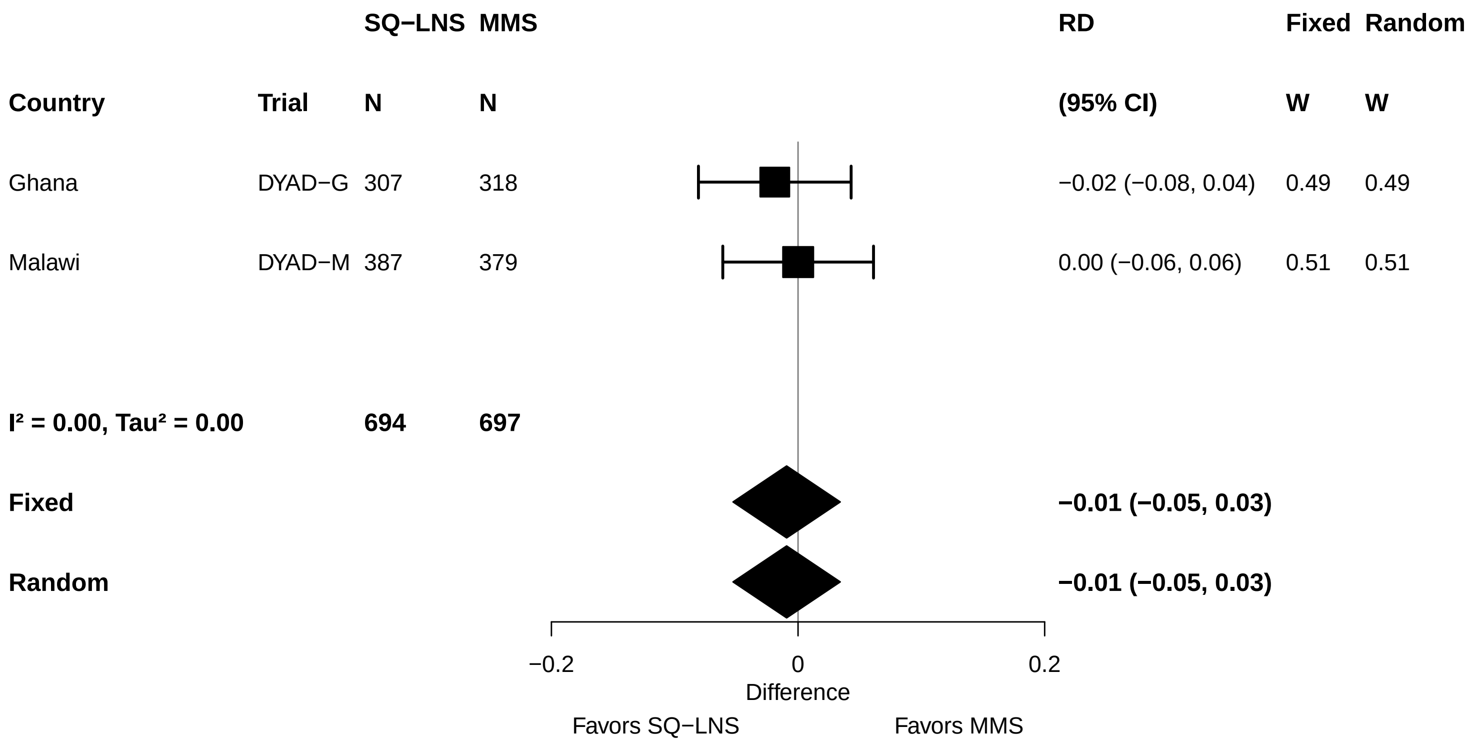

Supplemental figure 5J: Large-for-gestational age relative risk

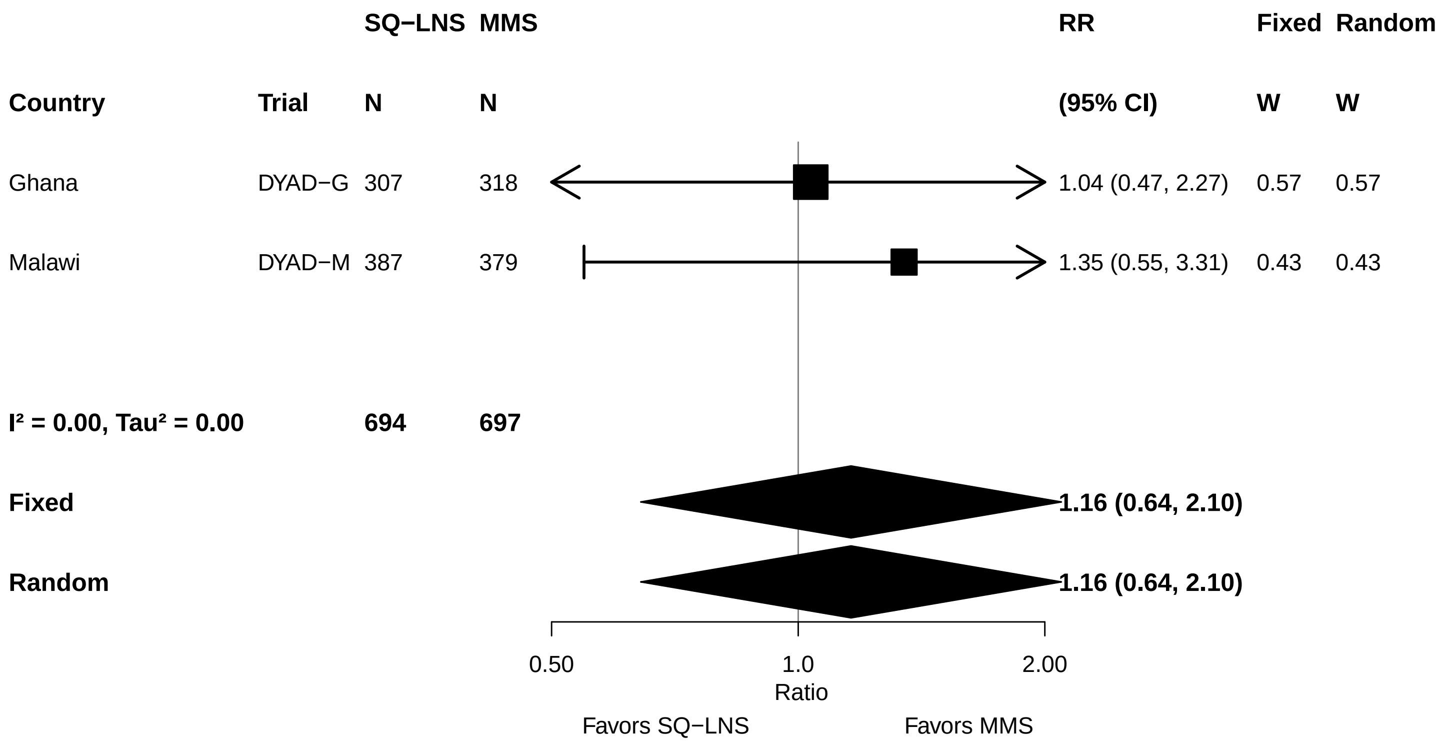

Supplemental figure 5K: Large-for-gestational age risk difference

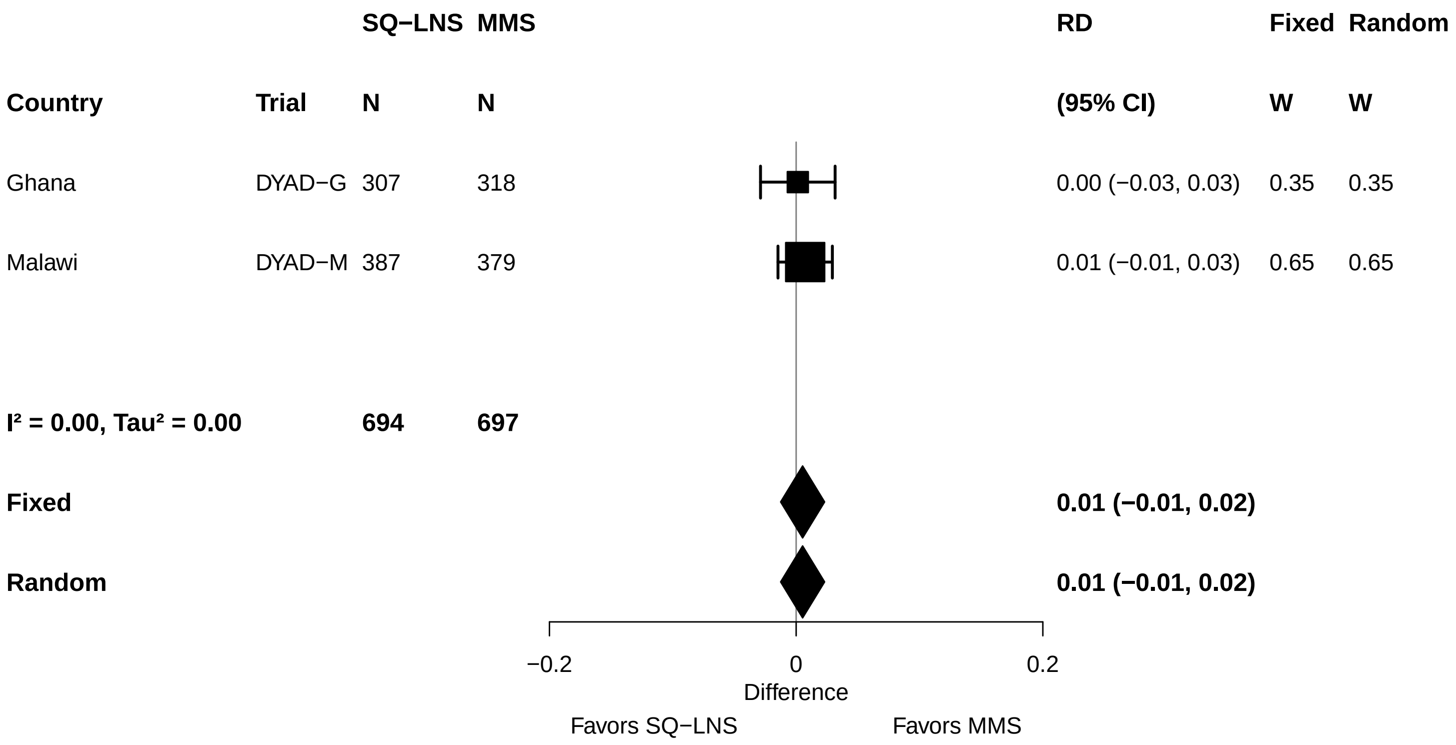

Supplemental figure 5L: Mean difference in birth length (cm)

Country	Trial	SQ−LNS N	MMS N	MD (95% CI)	Fixed W	Random W
Ghana	DYAD−G	307	318	0.18 (−0.17, 0.52)	0.50	0.50
Malawi	DYAD−M	253	259	0.13 (−0.21, 0.47)	0.50	0.50
**I² = 0.00, Tau² = 0.00**		**560**	**577**			
**Fixed**				**0.16 (−0.09, 0.40)**		
**Random**				**0.16 (−0.09, 0.40)**		

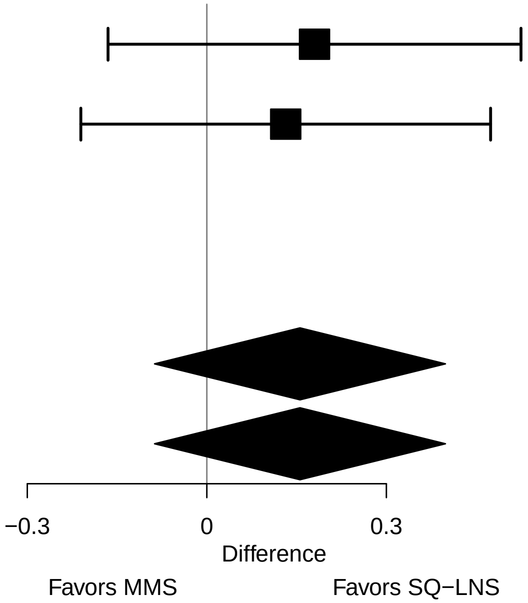

Supplemental figure 5M: Mean difference in length-for-age z score

Country	Trial	SQ−LNS N	MMS N	MD (95% CI)	Fixed W	Random W
Ghana	DYAD−G	307	318	0.07 (−0.10, 0.25)	0.51	0.51
Malawi	DYAD−M	253	259	0.07 (−0.11, 0.26)	0.49	0.49
**I² = 0.00, Tau² = 0.00**		**560**	**577**			
**Fixed**				**0.07 (−0.05, 0.20)**		
**Random**				**0.07 (−0.05, 0.20)**		

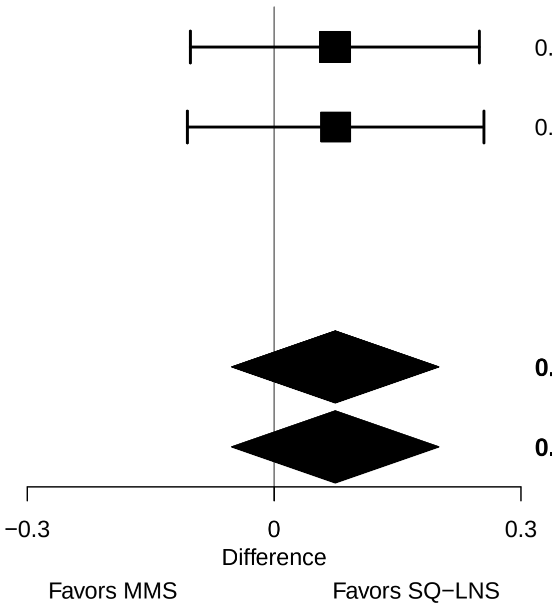

Supplemental figure 5N: Mean difference in length-for-gestational age z-score

Country	Trial	SQ−LNS N	MMS N	MD (95% CI)	Fixed W	Random W
Ghana	DYAD−G	298	313	0.07 (−0.09, 0.23)	0.55	0.55
Malawi	DYAD−M	253	259	0.09 (−0.09, 0.27)	0.45	0.45
**I² = 0.00, Tau² = 0.00**		**551**	**572**			
**Fixed**				**0.08 (−0.04, 0.20)**		
**Random**				**0.08 (−0.04, 0.20)**		

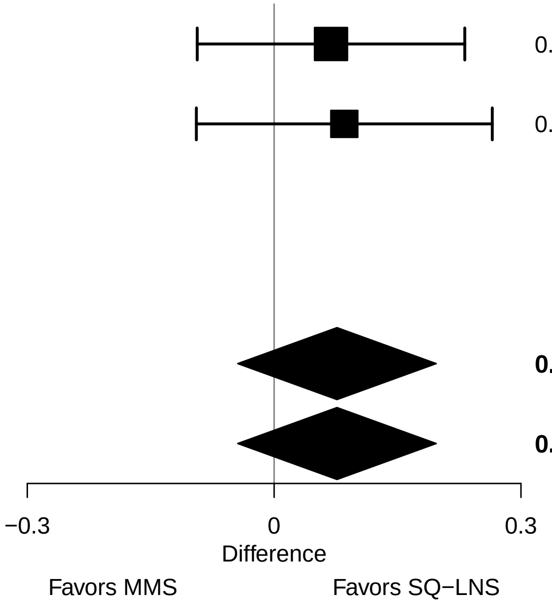

Supplemental figure 5O: Newborn stunting relative risk

Country	Trial	SQ−LNS N	MMS N	RR (95% CI)	Fixed W	Random W
Ghana	DYAD−G	307	318	0.97 (0.61, 1.56)	0.47	0.47
Malawi	DYAD−M	253	259	1.15 (0.74, 1.79)	0.53	0.53
**I² = 0.00, Tau² = 0.00**		**560**	**577**			
**Fixed**				**1.06 (0.77, 1.47)**		
**Random**				**1.06 (0.77, 1.47)**		

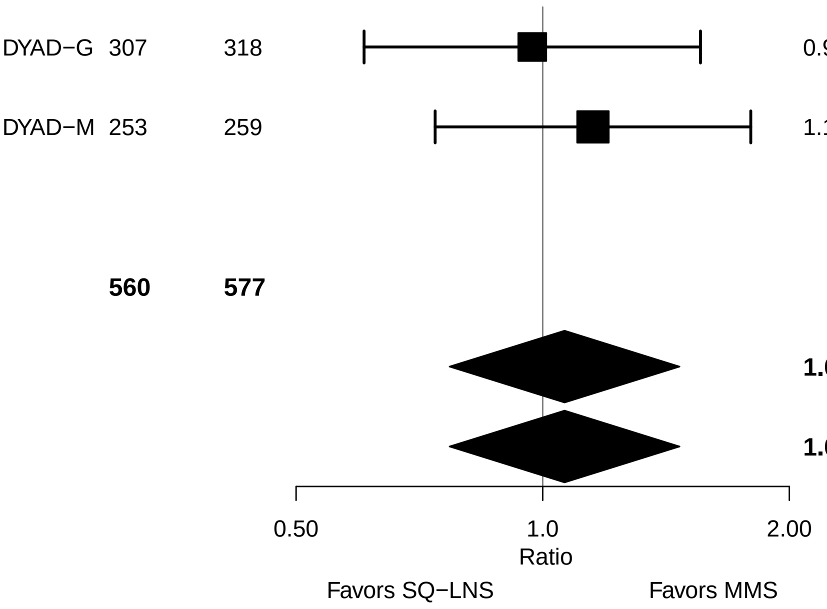

Supplemental figure 5P: Newborn stunting risk difference

Country	Trial	SQ−LNS N	MMS N	RD (95% CI)	Fixed W	Random W
Ghana	DYAD−G	307	318	0.00 (−0.05, 0.04)	0.61	0.61
Malawi	DYAD−M	253	259	0.02 (−0.04, 0.08)	0.39	0.39
**I² = 0.00, Tau² = 0.00**		**560**	**577**			
**Fixed**				**0.01 (−0.03, 0.04)**		
**Random**				**0.01 (−0.03, 0.04)**		

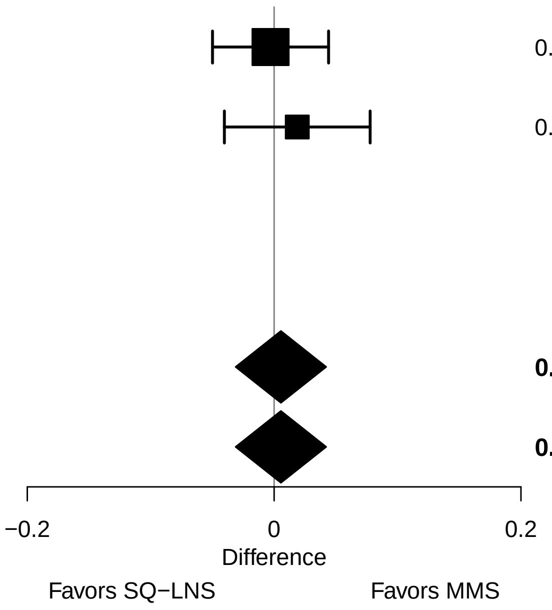

Supplemental figure 5Q: Low LGAZ relative risk

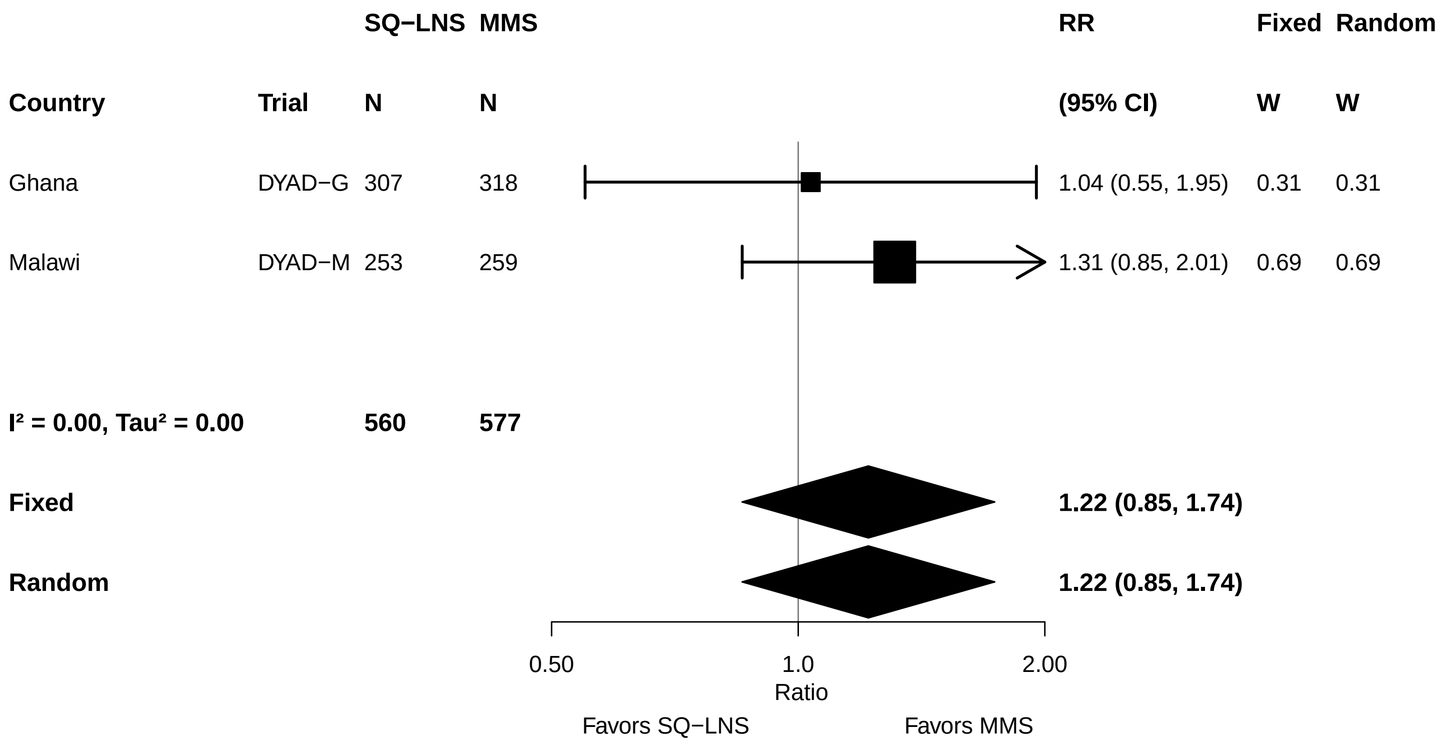

Supplemental figure 5R: Low LGAZ risk difference

Country	Trial	SQ−LNS N	MMS N	RD (95% CI)	Fixed W	Random W
Ghana	DYAD−G	307	318	0.00 (−0.03, 0.04)	0.73	0.73
Malawi	DYAD−M	253	259	0.04 (−0.02, 0.10)	0.27	0.27
**I² = 0.01, Tau² = 0.00**		**560**	**577**			
**Fixed**				**0.01 (−0.02, 0.04)**		
**Random**				**0.01 (−0.02, 0.04)**		

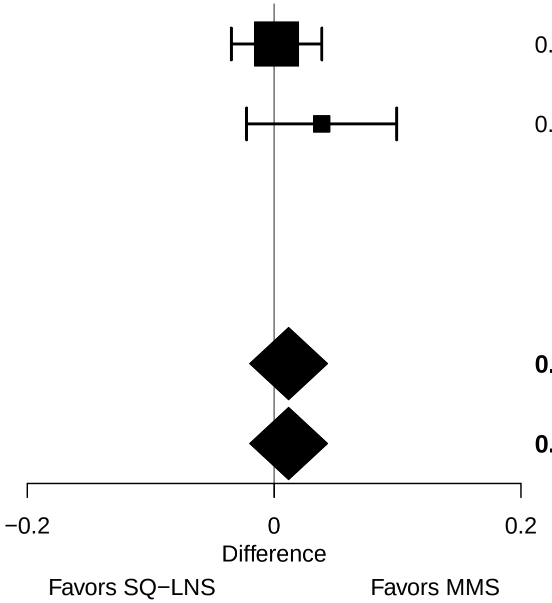

Supplemental figure 5S: Mean difference in BMI-for-age z-score

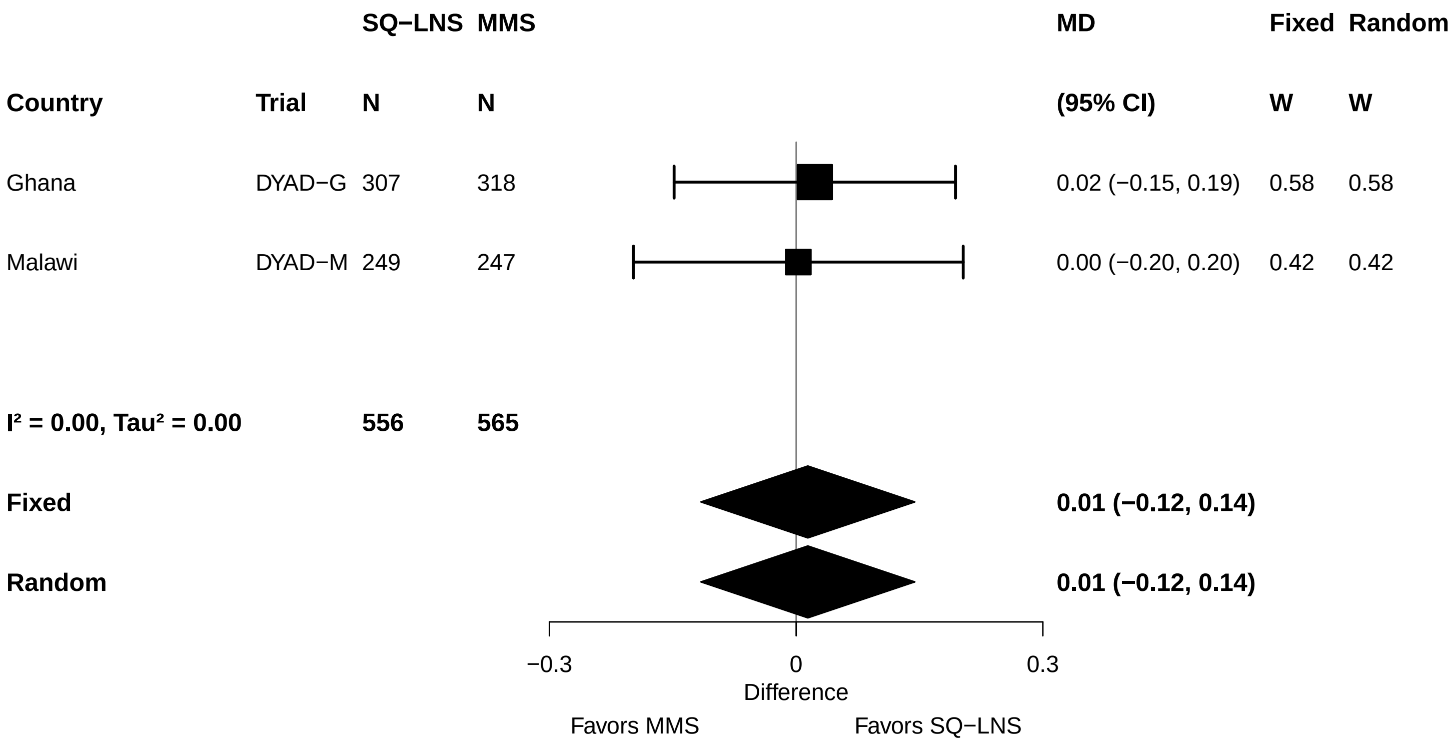

Supplemental figure 5T: Low BMIZ relative risk

Country	Trial	SQ−LNS N	MMS N	RR (95% CI)	Fixed W	Random W
Ghana	DYAD−G	307	318	0.78 (0.43, 1.40)	0.54	0.53
Malawi	DYAD−M	249	247	1.32 (0.69, 2.52)	0.46	0.47
**I² = 0.30, Tau² = 0.04**		**556**	**565**			
**Fixed**				**0.99 (0.64, 1.53)**		
**Random**				**1.00 (0.59, 1.68)**		

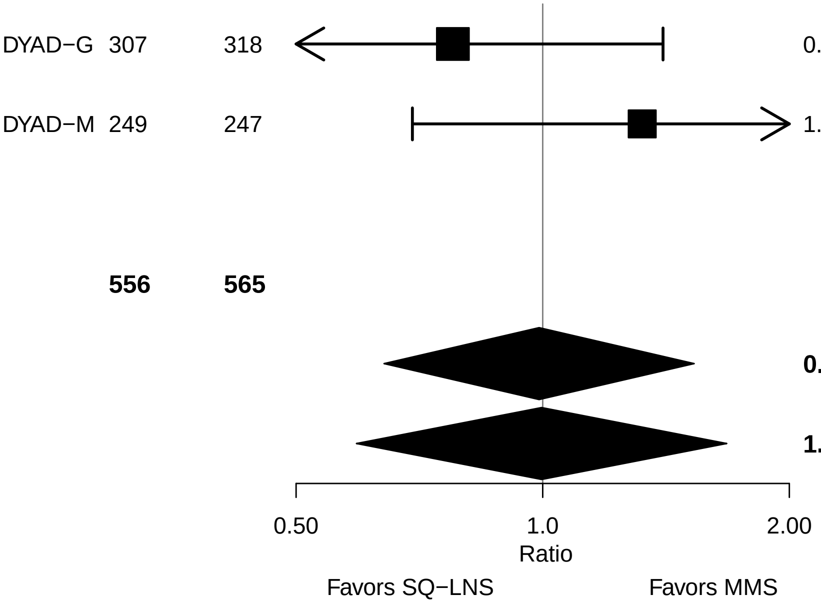

Supplemental figure 5U: Low BMIZ risk difference

Country	Trial	SQ−LNS N	MMS N	RD (95% CI)	Fixed W	Random W
Ghana	DYAD−G	307	318	−0.02 (−0.06, 0.02)	0.57	0.55
Malawi	DYAD−M	249	247	0.02 (−0.03, 0.06)	0.43	0.45
**I² = 0.30, Tau² = 0.00**		**556**	**565**			
**Fixed**				**0.00 (−0.03, 0.03)**		
**Random**				**0.00 (−0.04, 0.03)**		

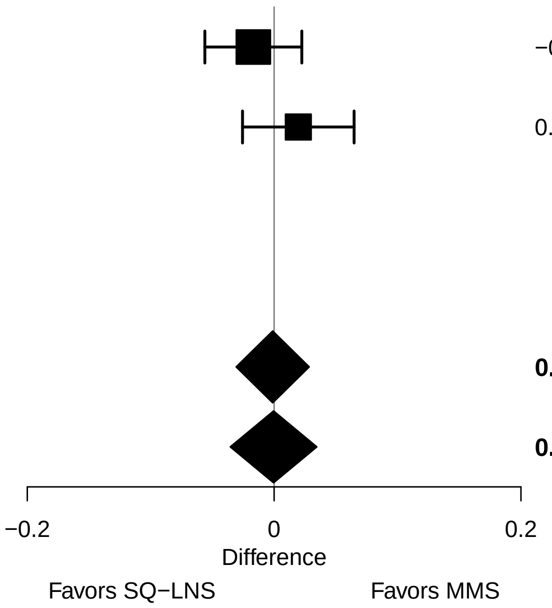

Supplemental figure 5V: Mean difference in head circumference (cm)

Country	Trial	SQ−LNS N	MMS N	MD (95% CI)	Fixed W	Random W
Ghana	DYAD−G	307	318	0.24 (0.00, 0.48)	0.48	0.48
Malawi	DYAD−M	253	260	0.06 (−0.17, 0.29)	0.52	0.52
**I² = 0.08, Tau² = 0.00**		**560**	**578**			
**Fixed**				**0.14 (−0.02, 0.31)**		
**Random**				**0.14 (−0.03, 0.32)**		

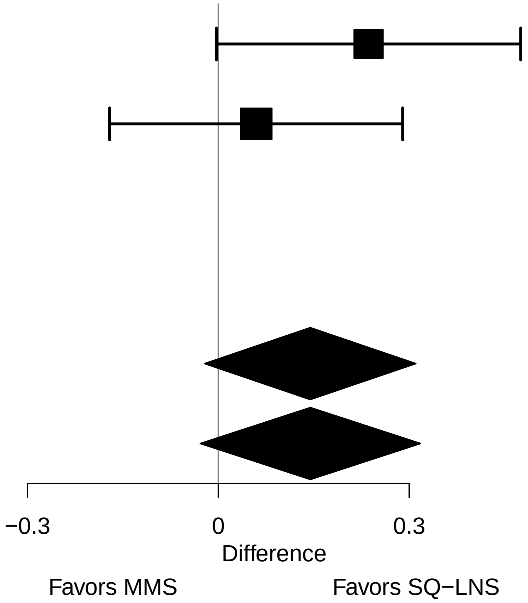

Supplemental figure 5W: Mean difference in head circumference-for-age z score

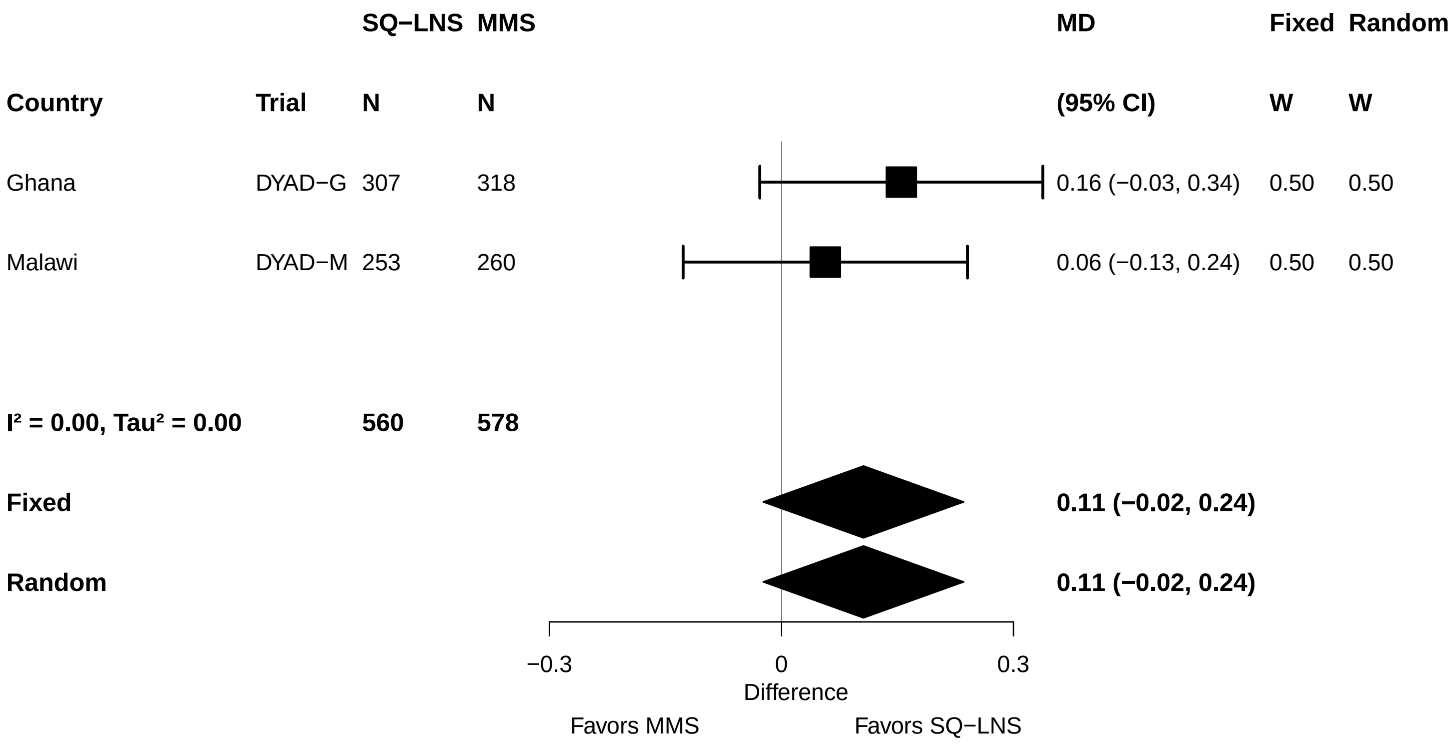

Supplemental figure 5X: Mean difference in head circumference-for-gestational age z score

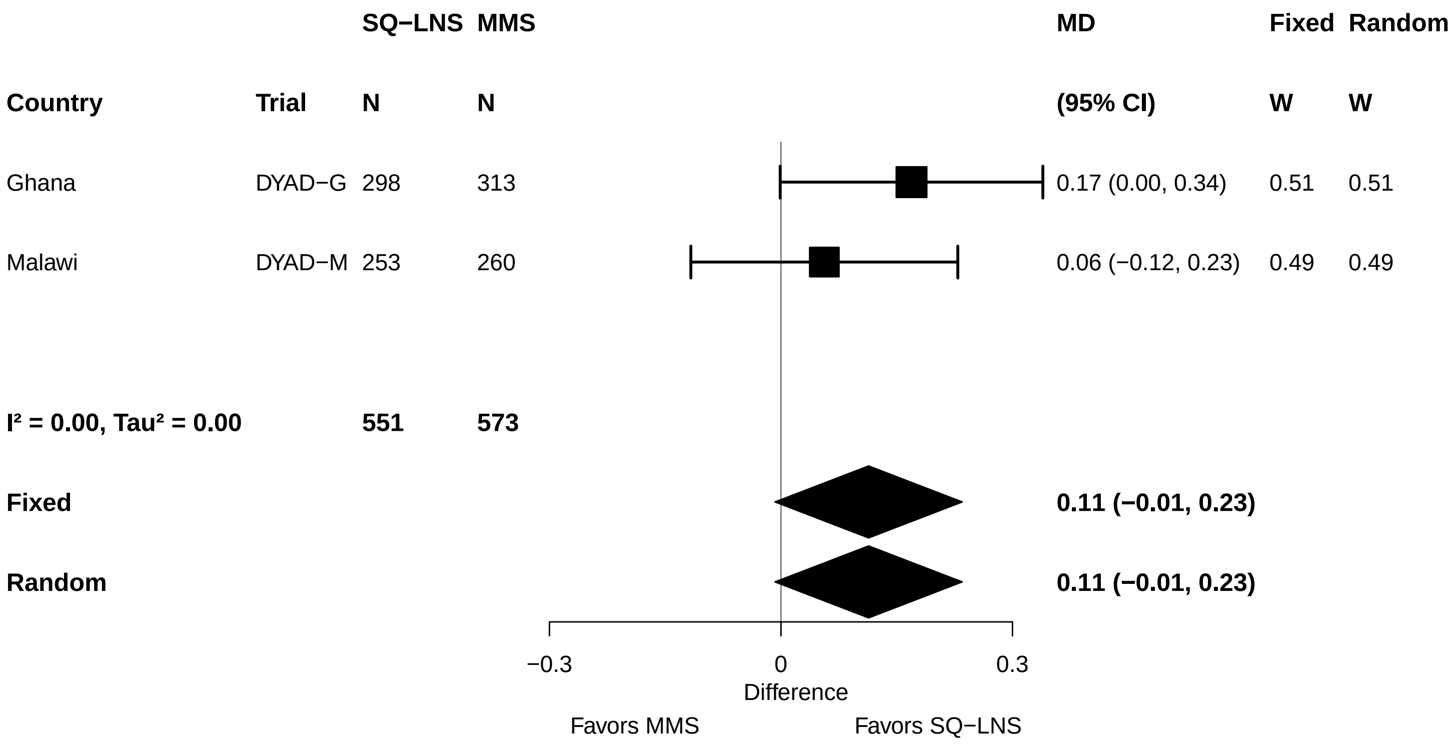

Supplemental figure 5Y: Low HCZ relative risk

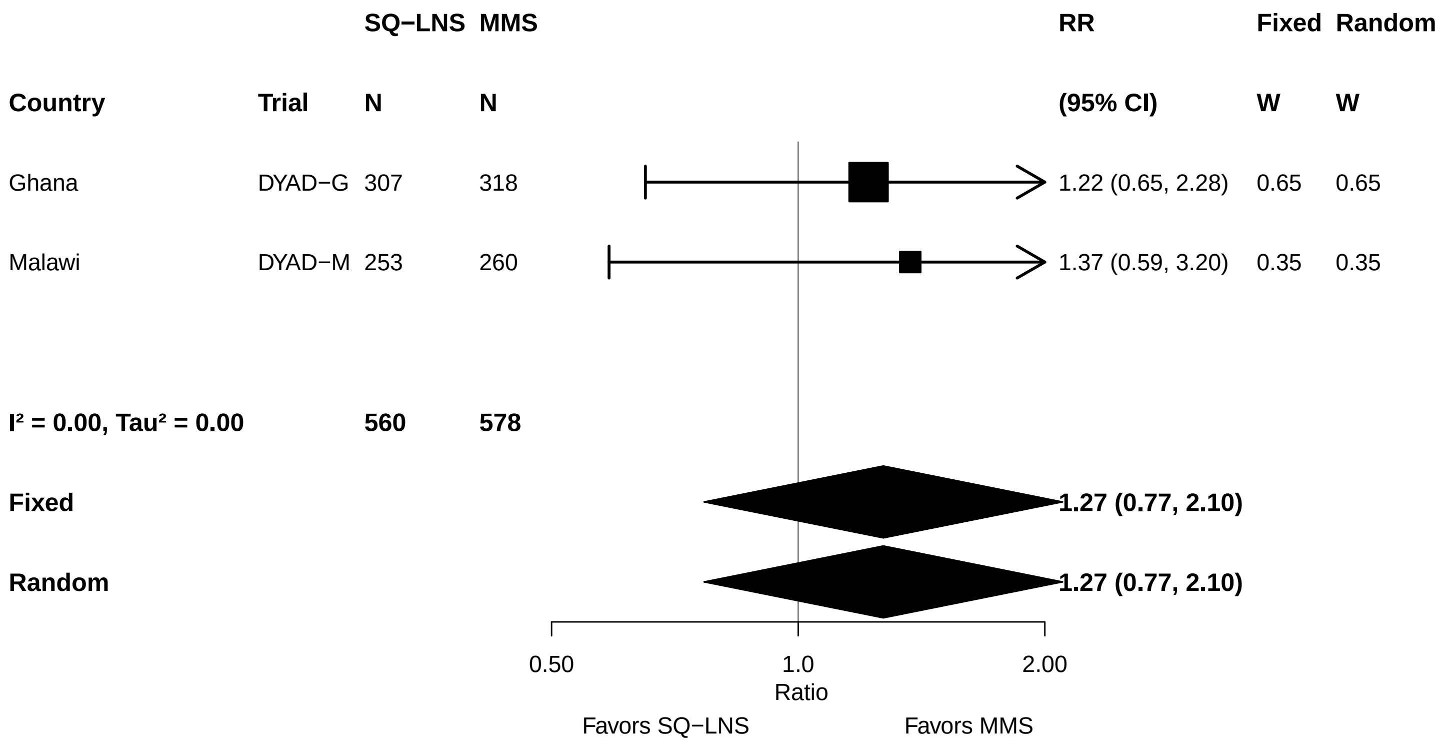

Supplemental figure 5Z: Low HCZ risk difference

Country	Trial	SQ−LNS N	MMS N	RD (95% CI)	Fixed W	Random W
Ghana	DYAD−G	307	318	0.01 (−0.03, 0.05)	0.46	0.46
Malawi	DYAD−M	253	260	0.01 (−0.02, 0.05)	0.54	0.54
**I² = 0.00, Tau² = 0.00**		**560**	**578**			
**Fixed**				**0.01 (−0.01, 0.04)**		
**Random**				**0.01 (−0.01, 0.04)**		

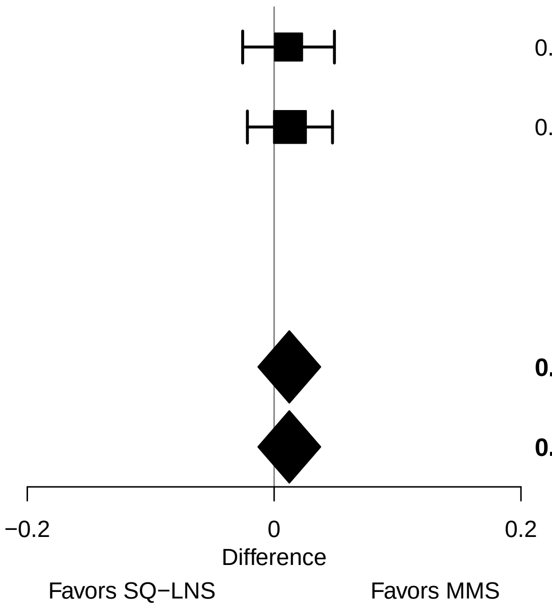

Supplemental figure 5AA: Low HCGAZ relative risk

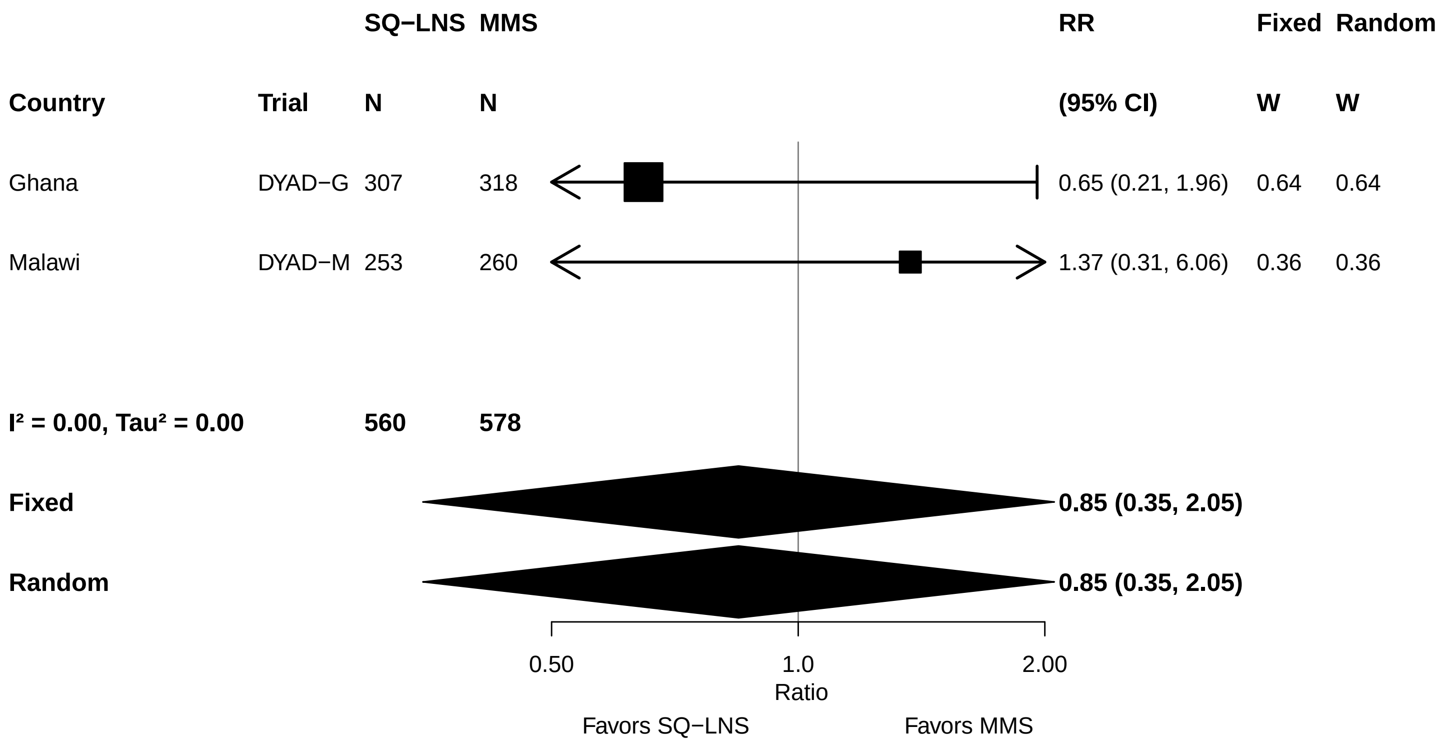

Supplemental figure 5AB: Low HCGAZ risk difference

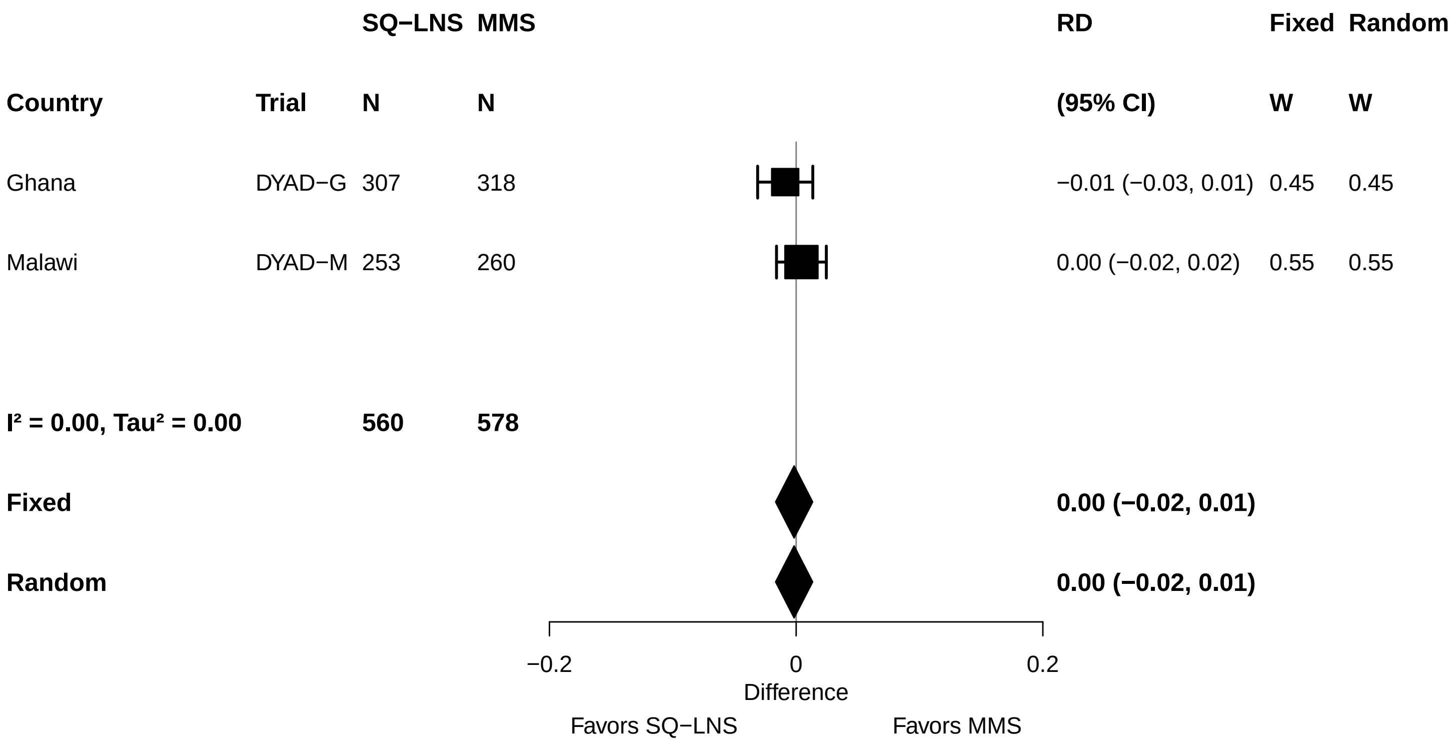

Supplemental figure 5AC: Mean difference in mid-upper arm circumference (cm)

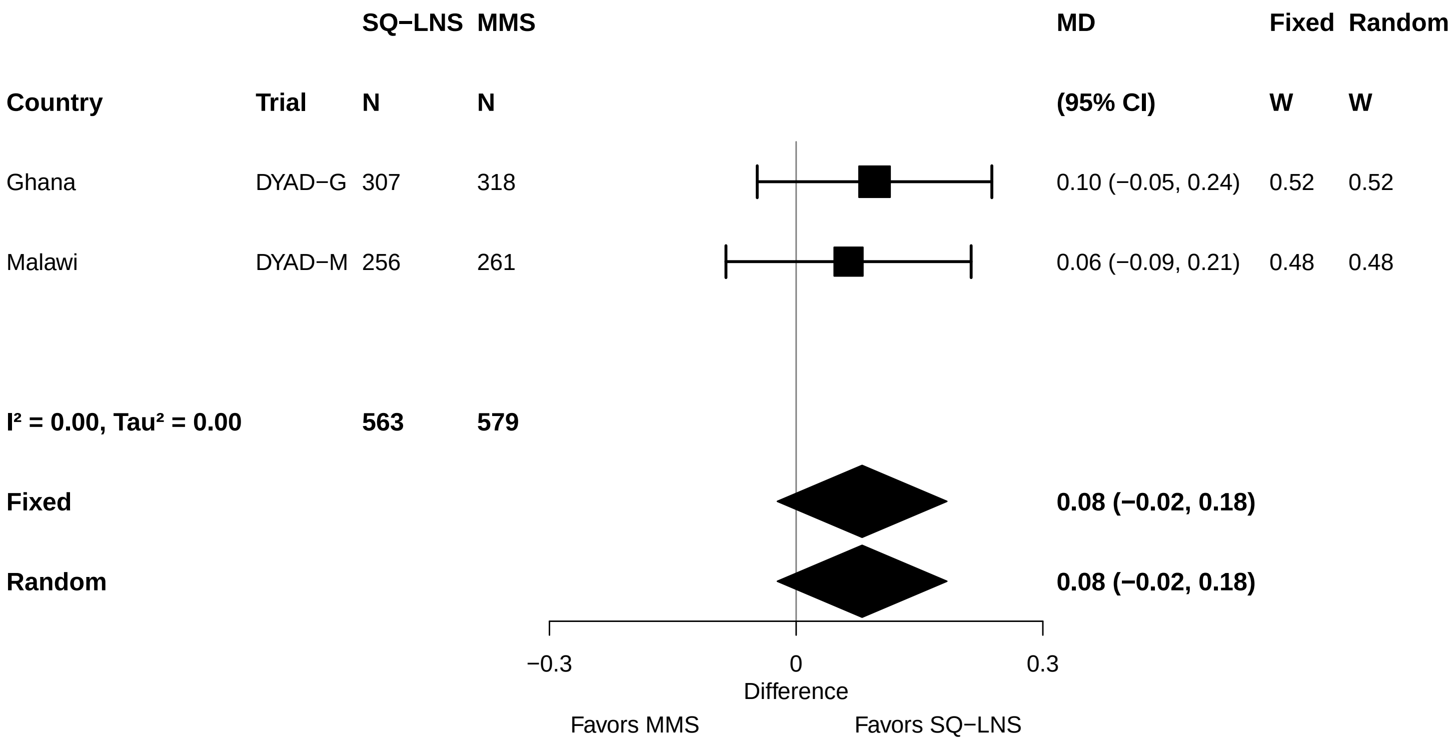

Supplemental figure 5AD: Mean difference in duration of gestation (wk)

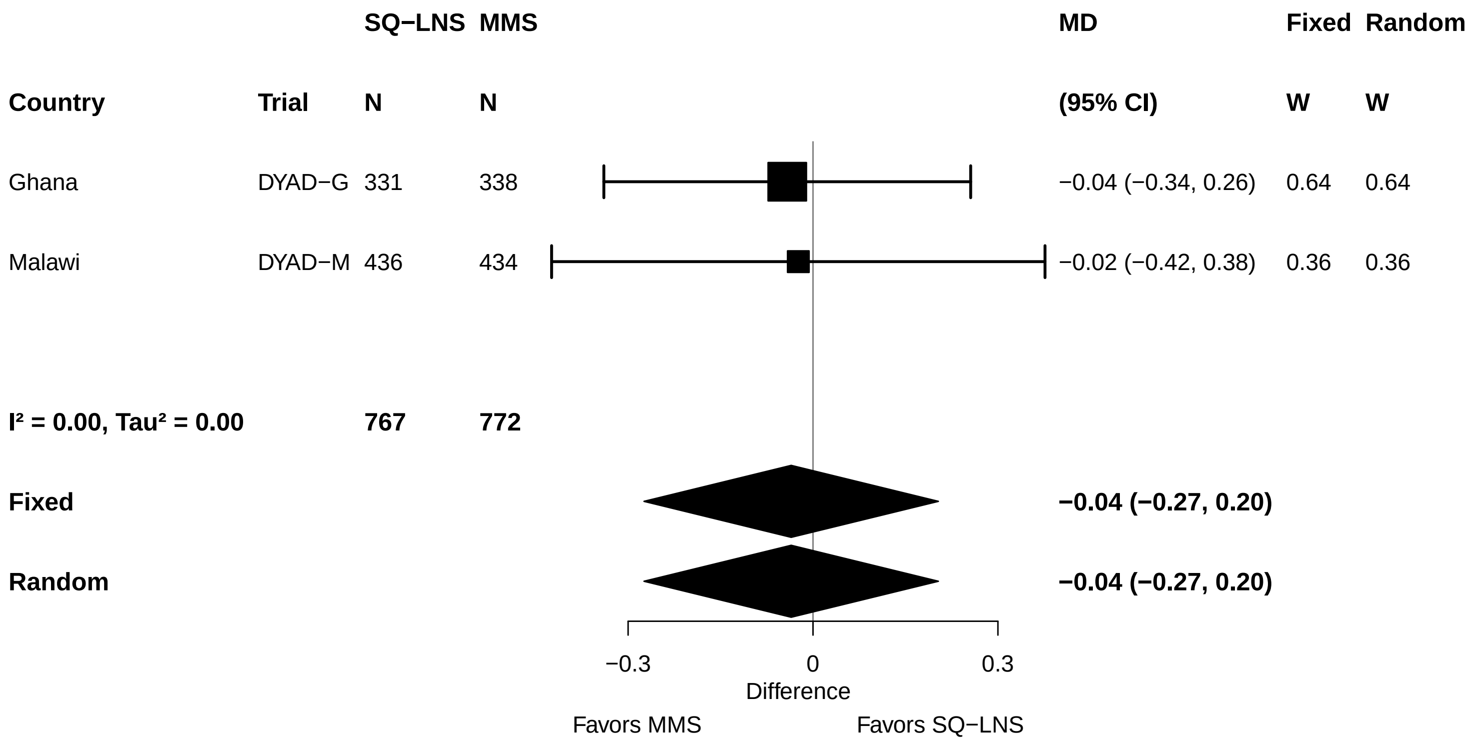

Supplemental figure 5AE: Preterm birth relative risk

Country	Trial	SQ−LNS N	MMS N	RR (95% CI)	Fixed W	Random W
Ghana	DYAD−G	331	338	1.49 (0.88, 2.50)	0.38	0.38
Malawi	DYAD−M	436	434	1.07 (0.71, 1.60)	0.62	0.62
**I² = 0.00, Tau² = 0.00**		**767**	**772**			
**Fixed**				**1.21 (0.88, 1.66)**		
**Random**				**1.21 (0.88, 1.66)**		

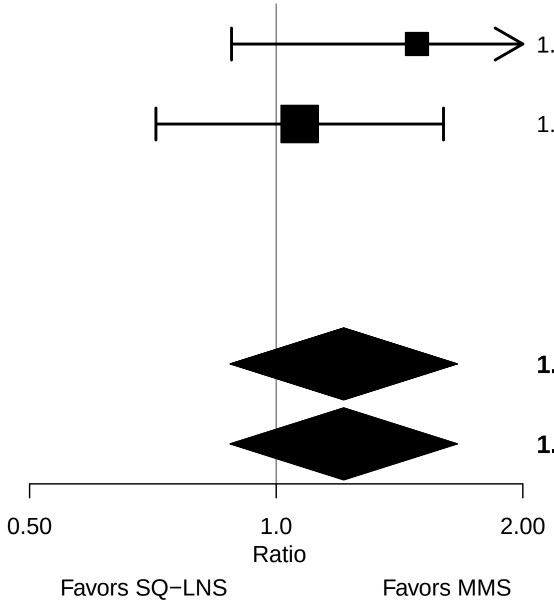

Supplemental figure 5AF: Preterm birth risk difference

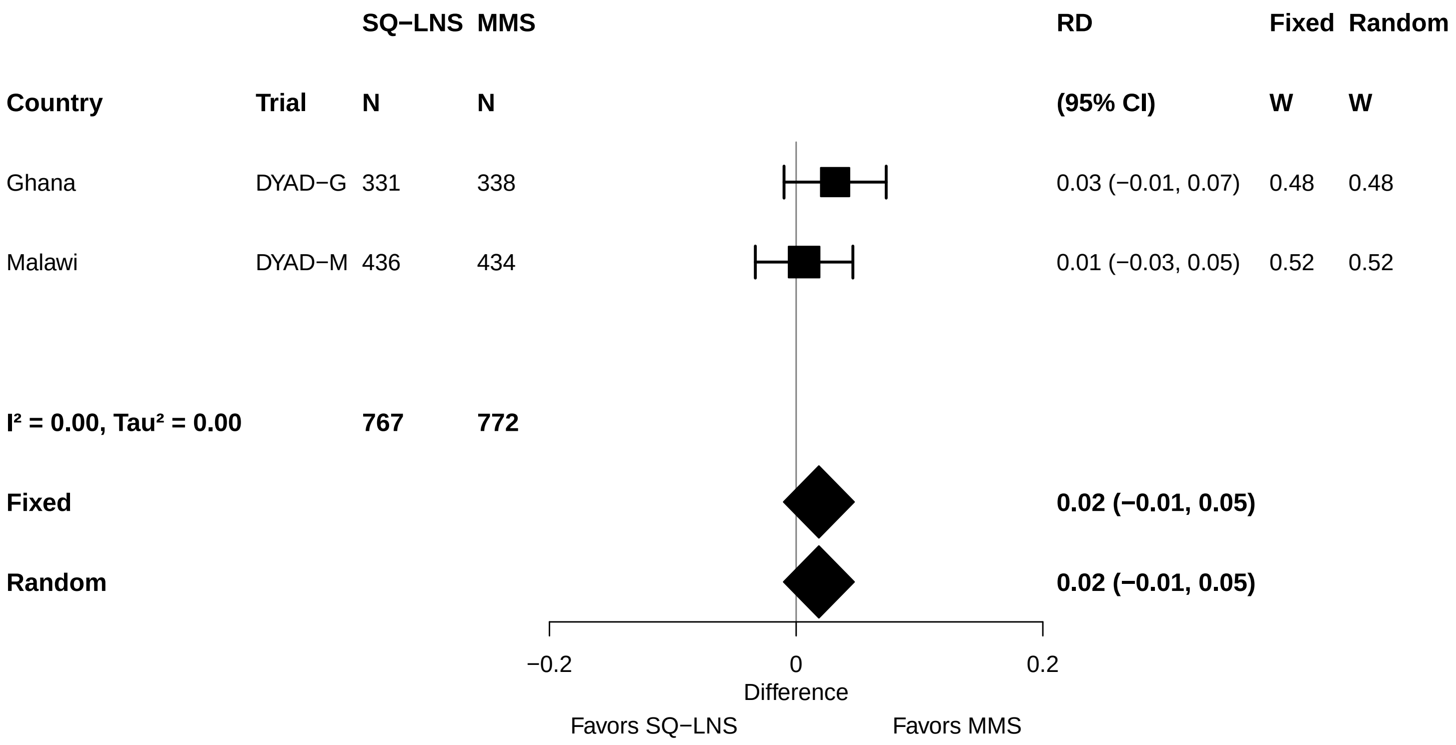

Supplement: Multimedia component 1 [file mmc1.zip › Maternal SQ-LNS Supplemental_2024-09-03/10_Maternal SQ-LNS Supplemental figure 5.pdf]
