# Supplementary material for: Effects of prenatal small-quantity lipid-based nutrient supplements on pregnancy, birth, and infant outcomes: a systematic review and meta-analysis of individual participant data from randomized controlled trials in low- and middle-income countries
Source: Am J Clin Nutr. 2024 Aug 16;120(4):814–35. doi: 10.1016/j.ajcnut.2024.08.008 (PMC11473441; doi:10.1016/j.ajcnut.2024.08.008)

Supplemental figure 6: Pooled plots for infant outcomes at birth and at 6 mo, stratified by potential effect modifiers, SQ-LNS vs MMS

Contents

Supplemental figure 6A: Sex **3**
6A1: Mean differences for birth outcomes . . . 3
6A2: Relative risks for birth outcomes . . . 4
6A3: Mean differences for 6 mo outcomes . . . 5
6A4: Prevalence ratios for 6 mo outcomes . . . 6

Supplemental figure 6B: Birth order **7**
6B1: Mean differences for birth outcomes . . . 7
6B2: Relative risks for birth outcomes . . . 8
6B3: Mean differences for 6 mo outcomes . . . 9
6B4: Prevalence ratios for 6 mo outcomes . . . 10

Supplemental figure 6C: Maternal height **11**
6C1: Mean differences for birth outcomes . . . 11
6C2: Relative risks for birth outcomes . . . 12
6C3: Mean differences for 6 mo outcomes . . . 13
6C4: Prevalence ratios for 6 mo outcomes . . . 14

Supplemental figure 6D: Maternal BMI **15**
6D1: Mean differences for birth outcomes . . . 15
6D2: Relative risks for birth outcomes . . . 16
6D3: Mean differences for 6 mo outcomes . . . 17
6D4: Prevalence ratios for 6 mo outcomes . . . 18

Supplemental figure 6E: Maternal age **19**
6E1: Mean differences for birth outcomes . . . 19
6E2: Relative risks for birth outcomes . . . 20
6E3: Mean differences for 6 mo outcomes . . . 21
6E4: Prevalence ratios for 6 mo outcomes . . . 22

Supplemental figure 6F: Maternal education **23**
6F1: Mean differences for birth outcomes . . . 23
6F2: Relative risks for birth outcomes . . . 24
6F3: Mean differences for 6 mo outcomes . . . 25
6F4: Prevalence ratios for 6 mo outcomes . . . 26

Supplemental figure 6G: Baseline anemia status **27**
6G1: Mean differences for birth outcomes . . . 27
6G2: Relative risks for birth outcomes . . . 28
6G3: Mean differences for 6 mo outcomes . . . 29
6G4: Prevalence ratios for 6 mo outcomes . . . 30

Supplemental figure 6H: Baseline inflammation status **31**
6H1: Mean differences for birth outcomes . . . 31
6H2: Relative risks for birth outcomes . . . 32
6H3: Mean differences for 6 mo outcomes . . . 33
6H4: Prevalence ratios for 6 mo outcomes . . . 34

Supplemental figure 6I: Baseline malaria status **35**
6I1: Mean differences for birth outcomes . . . 35
6I2: Relative risks for birth outcomes . . . 36
6I3: Mean differences for 6 mo outcomes . . . 37
6I4: Prevalence ratios for 6 mo outcomes . . . 38

Supplemental figure 6J: Gestational age at supplementation **39**
6J1: Mean differences for birth outcomes . . . 39
6J2: Relative risks for birth outcomes . . . 40
6J3: Mean differences for 6 mo outcomes . . . 41
6J4: Prevalence ratios for 6 mo outcomes . . . 42

Supplemental figure 6K: Compliance with supplementation **43**
6K1: Mean differences for birth outcomes . . . 43
6K2: Relative risks for birth outcomes . . . 44
6K3: Mean differences for 6 mo outcomes . . . 45
6K4: Prevalence ratios for 6 mo outcomes . . . 46

Supplemental figure 6L: Household socio-economic status **47**
6L1: Mean differences for birth outcomes . . . 47
6L2: Relative risks for birth outcomes . . . 48
6L3: Mean differences for 6 mo outcomes . . . 49
6L4: Prevalence ratios for 6 mo outcomes . . . 50

Supplemental figure 6M: Household food security **51**
6M1: Mean differences for birth outcomes 51
6M2: Relative risks for birth outcomes 52
6M3: Mean differences for 6 mo outcomes 53
6M4: Prevalence ratios for 6 mo outcomes 54

These figures show pooled effects of SQ-LNS within study-level and individual-level characteristic subgroups along with the p-for-interaction. For definitions of effect modifiers, see Box 1 in the main paper. Individual study estimates were generated from log-binomial regression for dichotomous outcomes and simple linear regression for continuous outcomes with clustered observations using robust standard errors for cluster-randomized trials. Pooled sub-group estimates and statistical testing of the pooled interaction term were generated using inverse-variance weighting. For continuous outcomes the intervention effect is measured by the difference in mean of the SQ-LNS group minus MMS. For dichotomous outcomes analyzed via prevalence/risk ratios, the effect estimate is the prevalence/risk in the SQ-LNS group divided by the prevalence/risk in the MMS group. For dichotomous outcomes analyzed via prevalence/risk differences, the effect estimate is the prevalence/risk in the SQ-LNS group minus the prevalence/risk in the IMMS group. The labels on the left y-axis correspond to the characteristic subgroups and their sample sizes. The values on the right indicate the pooled prevalence ratio and confidence interval within that subgroup.

LAZ, length-for-age z-score; WLZ, weight-for-length z-score; WAZ, weight for-age z-score; MUACZ, mid-upper arm circumference z-score; BMI, body mass index; HCZ, head circumference-for-age z-score; LGAZ, length-for-gestational-age z-score; HCGAZ, head circumference-for-gestational-age z-score; BMIZ, body mass index-for-age z-score; IFA/SOC, Iron and folic acid or standard of care; MD, mean difference; MMS, multiple micronutrient supplement; MUAC, mid-upper arm circumference; PR, prevalence ratio; PD, prevalence difference; RD, risk difference; RR, relative risk; SOC, standard of care; SQ-LNS, small-quantity lipid-based nutrient supplements; WGAZ, weight-for-gestational age z-score.

Supplemental figure 6A: Sex

6A1: Mean differences for birth outcomes

Outcome (p−for−interaction)	SQ−LNS N	MMS N	Mean difference (95% CI)
Birth weight (kg) (p=0.503)			
Male	344	329	0.01 (−0.06, 0.08)
Female	350	368	0.04 (−0.02, 0.10)
Weight−for−age z score (p=0.393)			
Male	344	329	0.01 (−0.14, 0.17)
Female	350	368	0.11 (−0.04, 0.26)
Weight−for−gestational age z−score (p=0.606)			
Male	341	327	0.03 (−0.12, 0.18)
Female	344	365	0.08 (−0.06, 0.23)
Birth length (cm) (p=0.116)			
Male	274	273	−0.06 (−0.39, 0.28)
Female	286	304	0.33 (−0.01, 0.66)
Length−for−age z−score (p=0.120)			
Male	274	273	−0.03 (−0.21, 0.14)
Female	286	304	0.17 (−0.01, 0.35)
Length−for−gestational age z−score (p=0.128)			
Male	271	271	−0.02 (−0.19, 0.15)
Female	280	301	0.17 (0.00, 0.33)
BMI z−score (p=0.852)			
Male	272	268	0.00 (−0.18, 0.18)
Female	284	297	0.03 (−0.16, 0.21)
Head circumference (cm) (p=0.097)			
Male	275	274	−0.01 (−0.22, 0.21)
Female	285	304	0.26 (0.03, 0.50)
Head circumference−for−age z−score (p=0.098)			
Male	275	274	−0.01 (−0.18, 0.16)
Female	285	304	0.21 (0.01, 0.40)
Head circumference−for−gestational age z score (p=0.177)			
Male	272	272	0.03 (−0.14, 0.20)
Female	279	301	0.19 (0.02, 0.37)
Mid−upper arm circumference (cm) (p=0.758)			
Male	277	274	0.05 (−0.09, 0.20)
Female	286	305	0.09 (−0.06, 0.24)
Duration (wk) (p=0.195)			
Male	376	363	−0.16 (−0.46, 0.15)
Female	376	394	0.13 (−0.16, 0.42)

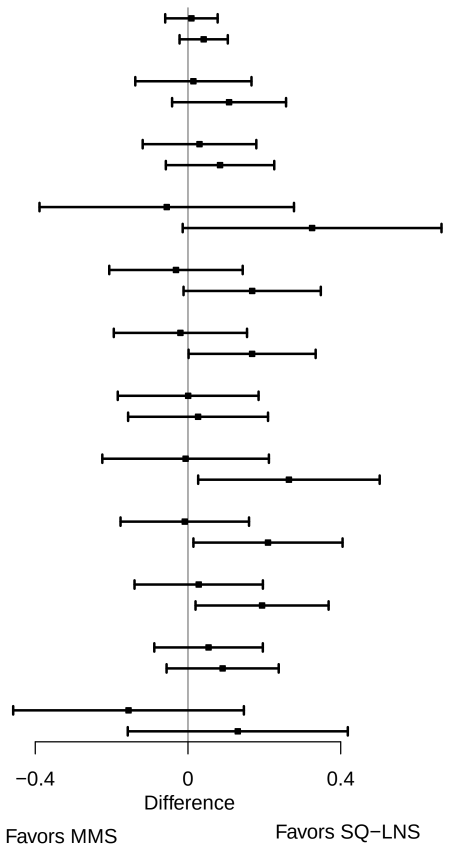

Supplemental figure 6A: Sex

6A2: Relative risks for birth outcomes

Outcome (p−for−interaction)	SQ−LNS N	MMS N	Relative risk (95% CI)
Low birth weight (p=0.301)			
Male	344	329	1.10 (0.67, 1.82)
Female	350	368	0.80 (0.56, 1.14)
Birth weight < 2 kg (p=0.079)			
Male	344	329	2.10 (0.74, 5.95)
Female	350	368	0.48 (0.15, 1.57)
Small−for−gestational age (p=0.094)			
Male	344	329	1.13 (0.86, 1.47)
Female	350	368	0.80 (0.60, 1.08)
Large−for−gestational age (p=0.204)			
Male	344	329	1.73 (0.69, 4.34)
Female	350	368	0.82 (0.36, 1.84)
Newborn stunting (p=0.128)			
Male	274	273	1.46 (0.87, 2.44)
Female	286	304	0.86 (0.56, 1.32)
Low LGAZ (p=0.414)			
Male	274	273	1.44 (0.87, 2.39)
Female	286	304	1.04 (0.63, 1.73)
Low BMIZ (p=0.006)			
Male	272	268	2.04 (0.98, 4.26)
Female	284	297	0.64 (0.35, 1.17)
Low HCZ (p=0.097)			
Male	275	274	2.55 (0.91, 7.14)
Female	285	304	0.93 (0.50, 1.70)
Preterm birth (p=0.159)			
Male	376	363	1.50 (0.89, 2.55)
Female	376	394	0.97 (0.61, 1.52)

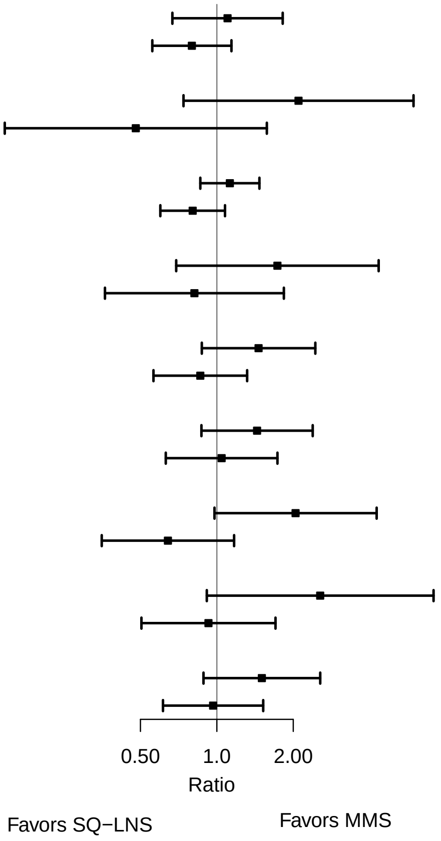

Supplemental figure 6A: Sex

6A3: Mean differences for 6 mo outcomes

Outcome (p−for−interaction)	SQ−LNS N	MMS N	Mean difference (95% CI)
Weight−for−age z score (p=0.185)			
Male	313	310	−0.10 (−0.29, 0.09)
Female	318	351	0.07 (−0.10, 0.23)
Length−for−age z−score (p=0.451)			
Male	313	309	−0.02 (−0.20, 0.15)
Female	319	348	0.07 (−0.09, 0.22)
Weight−for−length z−score (p=0.269)			
Male	313	309	−0.11 (−0.29, 0.08)
Female	318	348	0.03 (−0.13, 0.20)
Head circumference−for−age z−score (p=0.912)			
Male	313	310	−0.02 (−0.17, 0.14)
Female	319	351	−0.03 (−0.17, 0.12)
MUAC−for−age z−score (p=0.549)			
Male	313	310	−0.06 (−0.23, 0.11)
Female	319	351	0.02 (−0.14, 0.18)

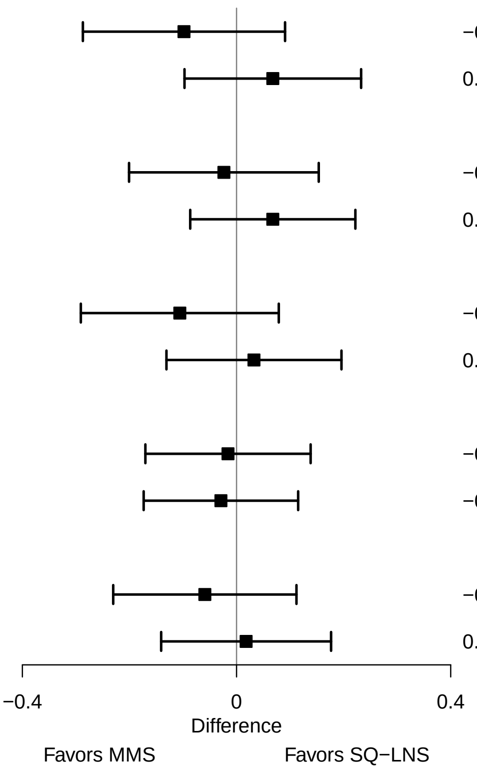

Supplemental figure 6A: Sex

6A4: Prevalence ratios for 6 mo outcomes

Outcome (p−for−interaction)	SQ−LNS N	MMS N	Prevalence ratio (95% CI)
Underweight (p=0.092)			
Male	313	310	1.47 (0.91, 2.38)
Female	318	351	0.75 (0.41, 1.36)
Stunted (p=0.869)			
Male	313	309	1.02 (0.75, 1.38)
Female	319	348	1.07 (0.72, 1.57)
Wasted (p=0.392)			
Male	313	309	1.32 (0.46, 3.76)
Female	318	348	0.82 (0.29, 2.34)
Low HCAZ (p=0.157)			
Male	313	310	0.99 (0.59, 1.67)
Female	319	351	0.55 (0.29, 1.03)
Low MUAC (p=0.626)			
Male	313	310	0.76 (0.34, 1.71)
Female	319	351	1.01 (0.65, 1.58)
Acute malnutrition (p=0.663)			
Male	313	309	0.86 (0.41, 1.77)
Female	318	348	1.07 (0.69, 1.65)

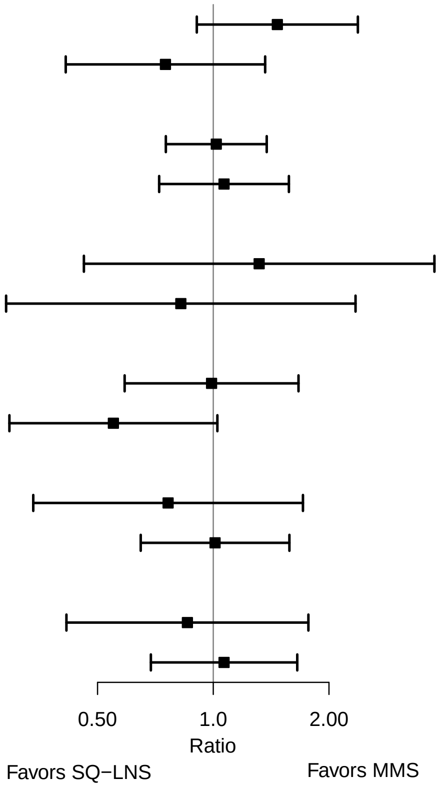

Supplemental figure 6B: Birth order

6B1: Mean differences for birth outcomes

Outcome (p−for−interaction)	SQ−LNS N	MMS N	Mean difference (95% CI)
Birth weight (kg) (p=0.033)			
Later born	511	521	0.00 (−0.05, 0.06)
Firstborn	183	175	0.12 (0.04, 0.21)
Weight−for−age z score (p=0.022)			
Later born	511	521	0.00 (−0.13, 0.12)
Firstborn	183	175	0.28 (0.08, 0.48)
Weight−for−gestational age z−score (p=0.362)			
Later born	508	516	0.03 (−0.09, 0.15)
Firstborn	177	175	0.15 (−0.04, 0.34)
Birth length (cm) (p=0.209)			
Later born	412	430	0.07 (−0.20, 0.34)
Firstborn	148	146	0.39 (−0.10, 0.89)
Length−for−age z−score (p=0.182)			
Later born	412	430	0.03 (−0.11, 0.17)
Firstborn	148	146	0.21 (−0.04, 0.47)
Length−for−gestational age z−score (p=0.353)			
Later born	409	425	0.05 (−0.09, 0.19)
Firstborn	142	146	0.18 (−0.06, 0.41)
BMI z−score (p=0.260)			
Later born	410	421	−0.03 (−0.18, 0.12)
Firstborn	146	143	0.17 (−0.09, 0.42)
Head circumference (cm) (p=0.138)			
Later born	412	431	0.07 (−0.12, 0.26)
Firstborn	148	146	0.39 (0.05, 0.74)
Head circumference−for−age z−score (p=0.109)			
Later born	412	431	0.05 (−0.10, 0.19)
Firstborn	148	146	0.31 (0.05, 0.58)
Head circumference−for−gestational age z score (p=0.253)			
Later born	409	426	0.07 (−0.07, 0.21)
Firstborn	142	146	0.26 (0.02, 0.50)
Mid−upper arm circumference (cm) (p=0.205)			
Later born	415	432	0.04 (−0.08, 0.16)
Firstborn	148	146	0.20 (0.01, 0.38)
Duration (wk) (p=0.140)			
Later born	554	569	−0.15 (−0.41, 0.12)
Firstborn	213	202	0.24 (−0.27, 0.76)

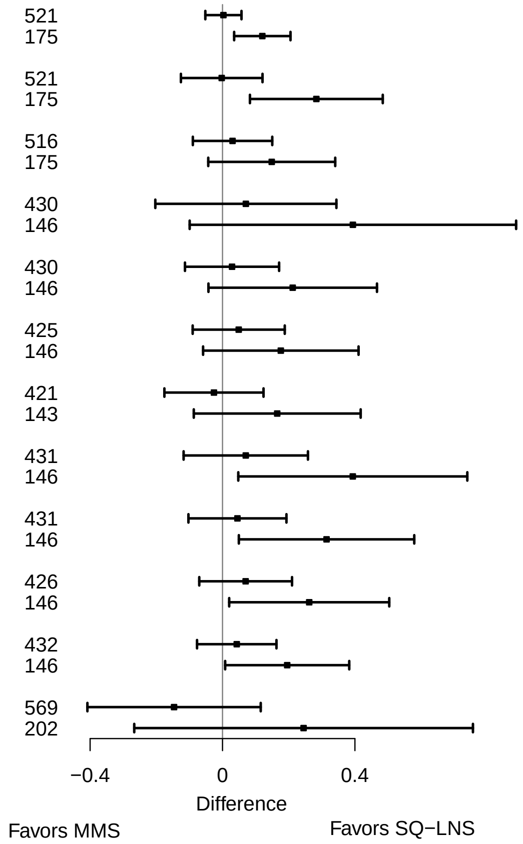

Supplemental figure 6B: Birth order

6B2: Relative risks for birth outcomes

Outcome (p−for−interaction)	SQ−LNS N	MMS N	Relative risk (95% CI)
Low birth weight (p=0.266)			
Later born	511	521	1.00 (0.69, 1.45)
Firstborn	183	175	0.71 (0.44, 1.15)
Birth weight < 2 kg (p=0.881)			
Later born	511	521	1.12 (0.45, 2.76)
Firstborn	183	175	0.98 (0.28, 3.42)
Small−for−gestational age (p=0.396)			
Later born	511	521	1.03 (0.80, 1.31)
Firstborn	183	175	0.84 (0.60, 1.17)
Newborn stunting (p=0.526)			
Later born	412	430	1.16 (0.78, 1.74)
Firstborn	148	146	0.90 (0.52, 1.57)
Low LGAZ (p=0.782)			
Later born	412	430	1.19 (0.78, 1.81)
Firstborn	148	146	1.26 (0.63, 2.52)
Low BMIZ (p=0.596)			
Later born	410	421	1.12 (0.64, 1.98)
Firstborn	146	143	0.84 (0.38, 1.89)
Low HCZ (p=0.181)			
Later born	412	431	1.60 (0.84, 3.04)
Firstborn	148	146	0.75 (0.31, 1.84)
Low HCGAZ (p=0.893)			
Later born	412	431	0.90 (0.30, 2.74)
Firstborn	148	146	0.74 (0.17, 3.27)
Preterm birth (p=0.814)			
Later born	554	569	1.23 (0.82, 1.84)
Firstborn	213	202	1.13 (0.67, 1.91)

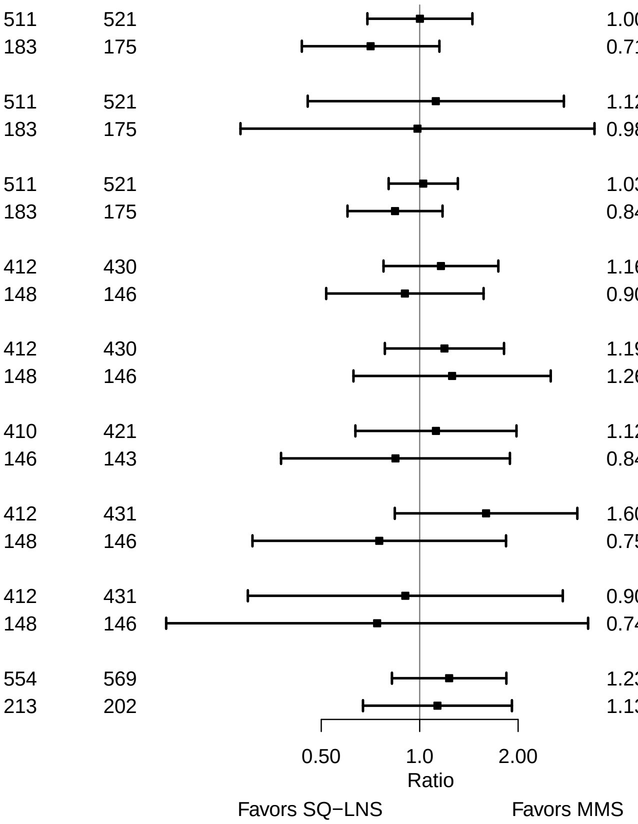

Supplemental figure 6B: Birth order

6B3: Mean differences for 6 mo outcomes

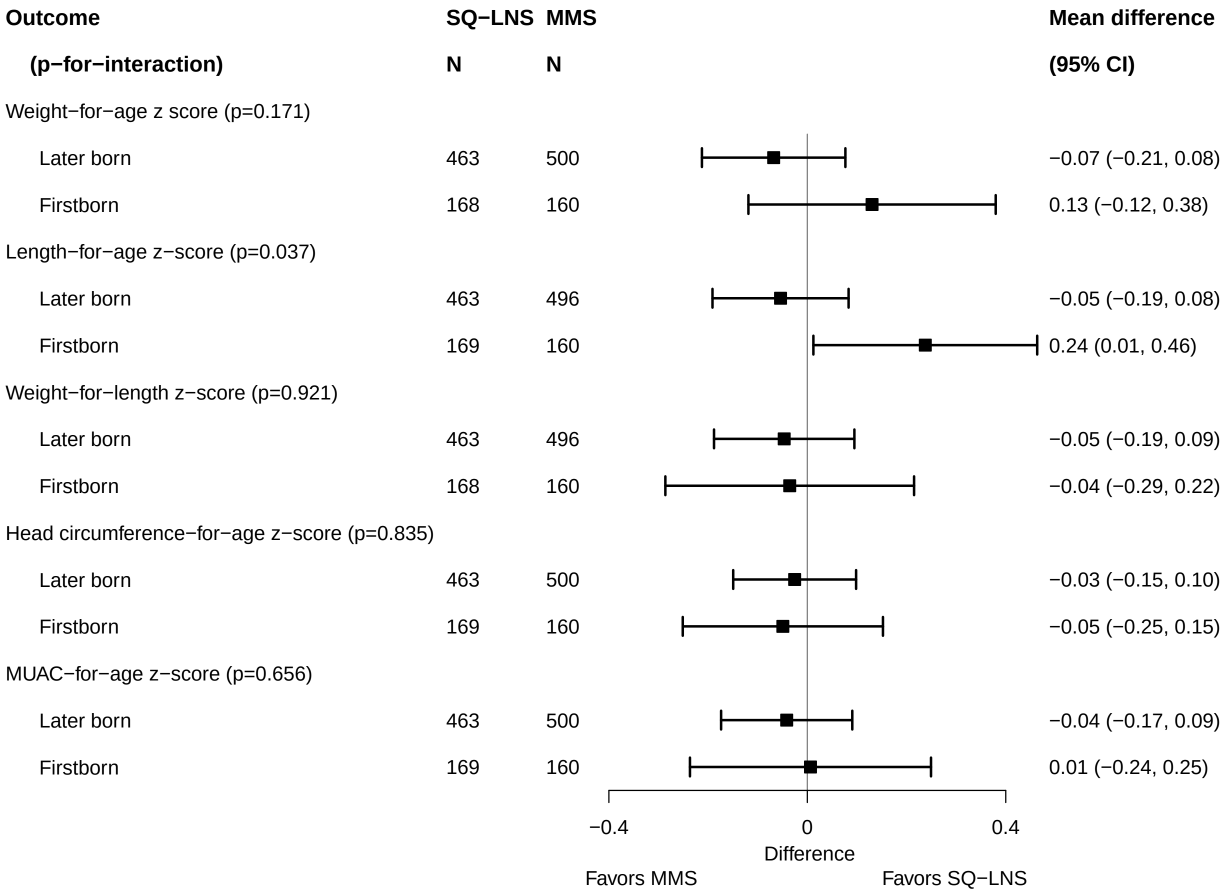

Supplemental figure 6B: Birth order

6B4: Prevalence ratios for 6 mo outcomes

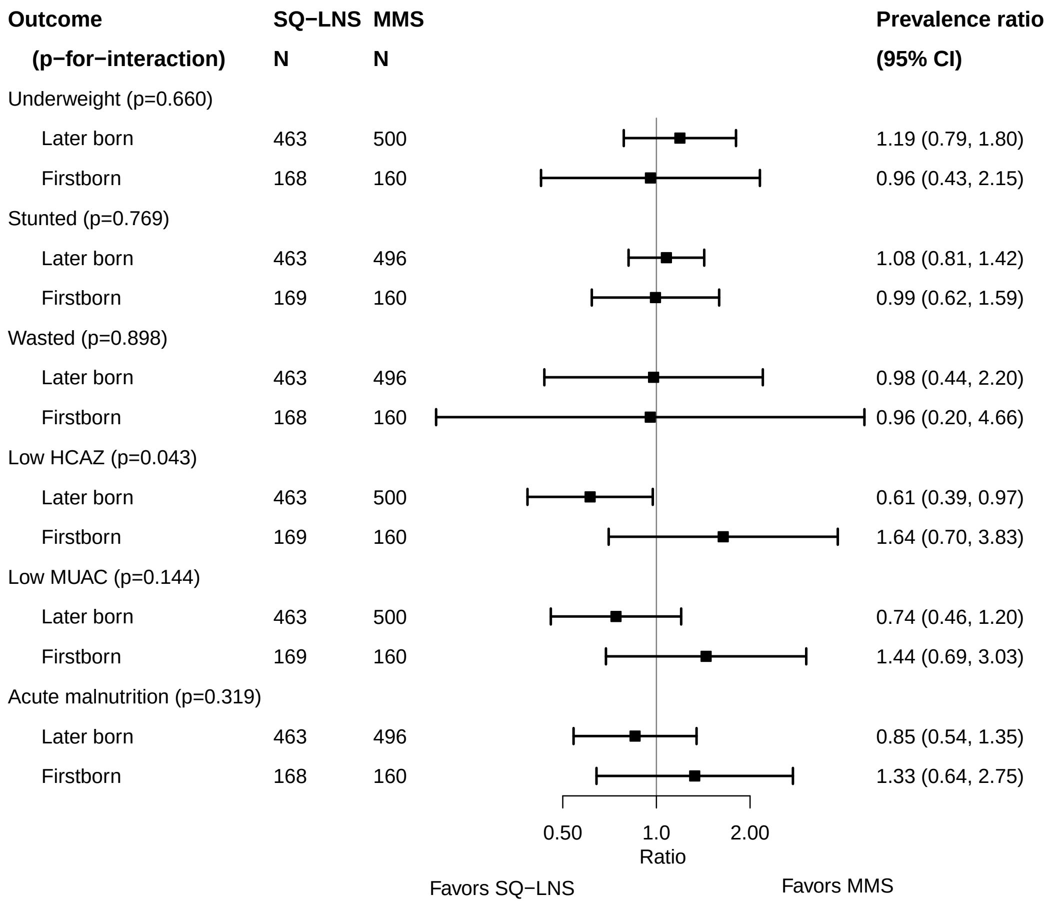

Supplemental figure 6C: Maternal height

6C1: Mean differences for birth outcomes

Outcome (p−for−interaction)	SQ−LNS N	MMS N	Mean difference (95% CI)
Birth weight (kg) (p=0.704)			
At least 150.1 cm	633	623	0.02 (−0.03, 0.07)
Less than 150.1 cm	56	65	0.12 (−0.02, 0.26)
Weight−for−age z score (p=0.725)			
At least 150.1 cm	633	623	0.05 (−0.06, 0.16)
Less than 150.1 cm	56	65	0.26 (−0.07, 0.60)
Weight−for−gestational age z−score (p=0.850)			
At least 150.1 cm	624	618	0.05 (−0.06, 0.16)
Less than 150.1 cm	56	65	0.11 (−0.20, 0.42)
Birth length (cm) (p=0.530)			
At least 150.1 cm	517	522	0.16 (−0.09, 0.41)
Less than 150.1 cm	38	47	0.01 (−0.82, 0.83)
Length−for−age z−score (p=0.721)			
At least 150.1 cm	517	522	0.07 (−0.06, 0.20)
Less than 150.1 cm	38	47	0.02 (−0.42, 0.46)
Length−for−gestational age z−score (p=0.242)			
At least 150.1 cm	508	517	0.09 (−0.03, 0.22)
Less than 150.1 cm	38	47	−0.18 (−0.57, 0.21)
BMI z−score (p=0.295)			
At least 150.1 cm	514	512	−0.01 (−0.14, 0.13)
Less than 150.1 cm	37	45	0.42 (−0.03, 0.87)
Head circumference (cm) (p=0.484)			
At least 150.1 cm	516	523	0.16 (−0.02, 0.33)
Less than 150.1 cm	39	47	0.09 (−0.43, 0.62)
Head circumference−for−age z−score (p=0.701)			
At least 150.1 cm	516	523	0.11 (−0.03, 0.25)
Less than 150.1 cm	39	47	0.08 (−0.35, 0.51)
Head circumference−for−gestational age z score (p=0.338)			
At least 150.1 cm	507	518	0.13 (0.00, 0.26)
Less than 150.1 cm	39	47	−0.11 (−0.48, 0.27)
Mid−upper arm circumference (cm) (p=0.548)			
At least 150.1 cm	519	524	0.09 (−0.02, 0.20)
Less than 150.1 cm	39	47	0.15 (−0.21, 0.51)
Duration (wk) (p=0.169)			
At least 150.1 cm	696	683	−0.10 (−0.35, 0.14)
Less than 150.1 cm	66	80	0.79 (−0.16, 1.74)

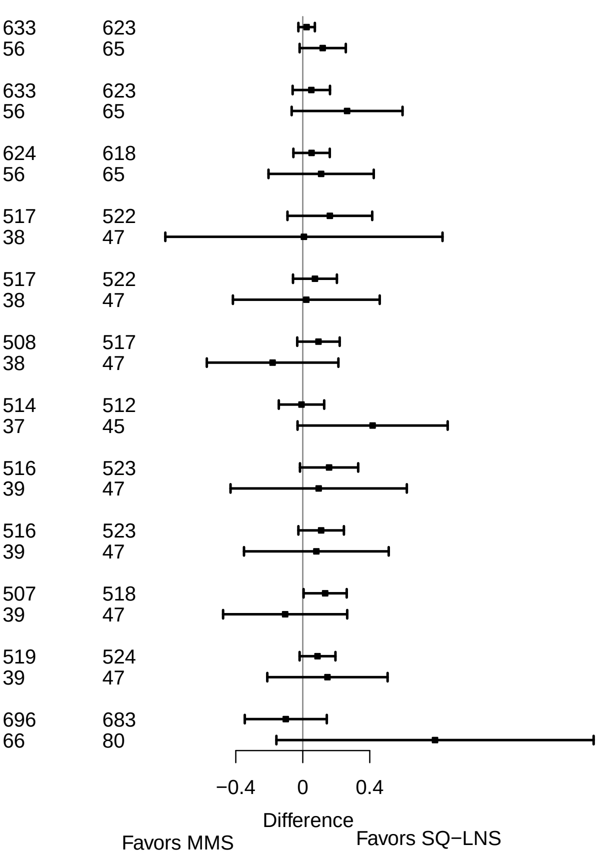

Supplemental figure 6C: Maternal height

6C2: Relative risks for birth outcomes

**Outcome (p−for−interaction)**	**SQ−LNS N**	**MMS N**	**Relative risk (95% CI)**
Small−for−gestational age (p=0.881)			
At least 150.1 cm	633	623	0.97 (0.78, 1.19)
Less than 150.1 cm	56	65	0.96 (0.54, 1.72)
Newborn stunting (p=0.583)			
At least 150.1 cm	517	522	1.04 (0.74, 1.48)
Less than 150.1 cm	38	47	1.42 (0.60, 3.36)
Low LGAZ (p=0.228)			
At least 150.1 cm	517	522	1.14 (0.78, 1.66)
Less than 150.1 cm	38	47	2.46 (0.79, 7.65)
Preterm birth (p=0.901)			
At least 150.1 cm	696	683	1.25 (0.89, 1.76)
Less than 150.1 cm	66	80	1.07 (0.44, 2.65)

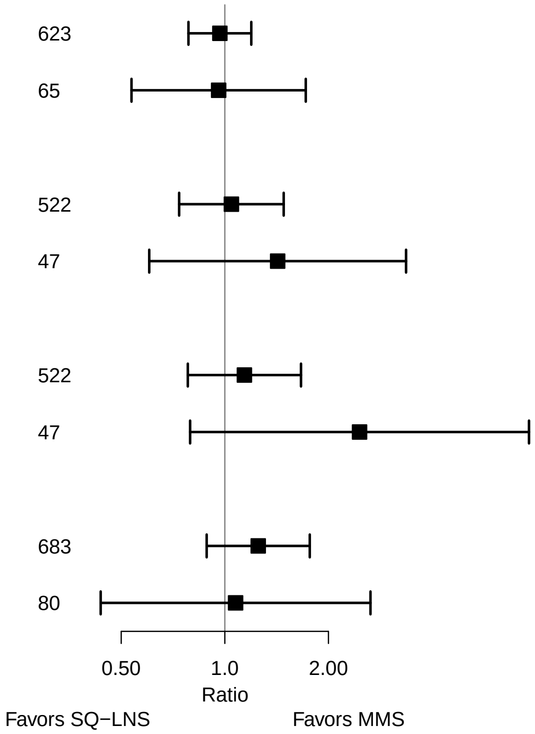

Supplemental figure 6C: Maternal height

6C3: Mean differences for 6 mo outcomes

Outcome (p−for−interaction)	SQ−LNS N	MMS N	Mean difference (95% CI)
Weight−for−age z score (p=0.171)			
At least 150.1 cm	576	583	−0.01 (−0.14, 0.13)
Less than 150.1 cm	50	70	−0.32 (−0.71, 0.07)
Length−for−age z−score (p=0.942)			
At least 150.1 cm	577	580	0.00 (−0.12, 0.12)
Less than 150.1 cm	50	69	−0.03 (−0.42, 0.36)
Weight−for−length z−score (p=0.051)			
At least 150.1 cm	576	580	−0.01 (−0.14, 0.12)
Less than 150.1 cm	50	69	−0.44 (−0.83, −0.04)
Head circumference−for−age z−score (p=0.450)			
At least 150.1 cm	577	583	−0.01 (−0.12, 0.10)
Less than 150.1 cm	50	70	−0.21 (−0.53, 0.12)
MUAC−for−age z−score (p=0.056)			
At least 150.1 cm	577	583	0.00 (−0.12, 0.12)
Less than 150.1 cm	50	70	−0.39 (−0.77, 0.00)

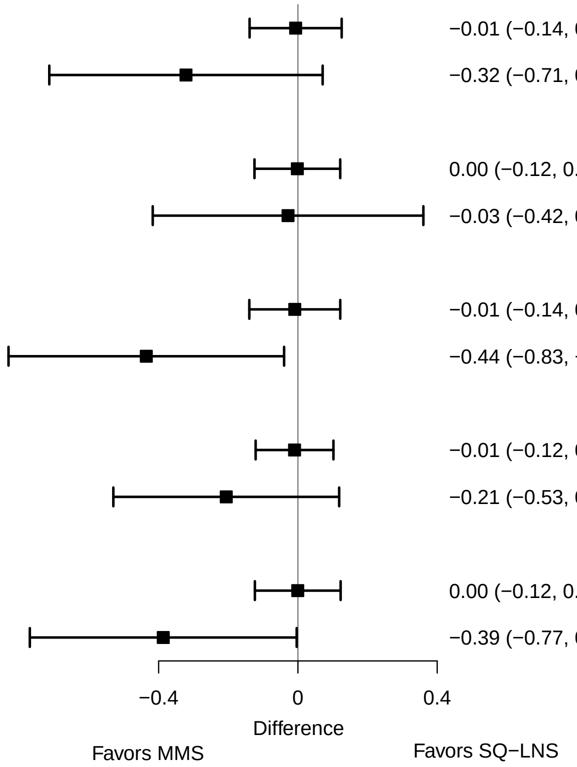

Supplemental figure 6C: Maternal height

6C4: Prevalence ratios for 6 mo outcomes

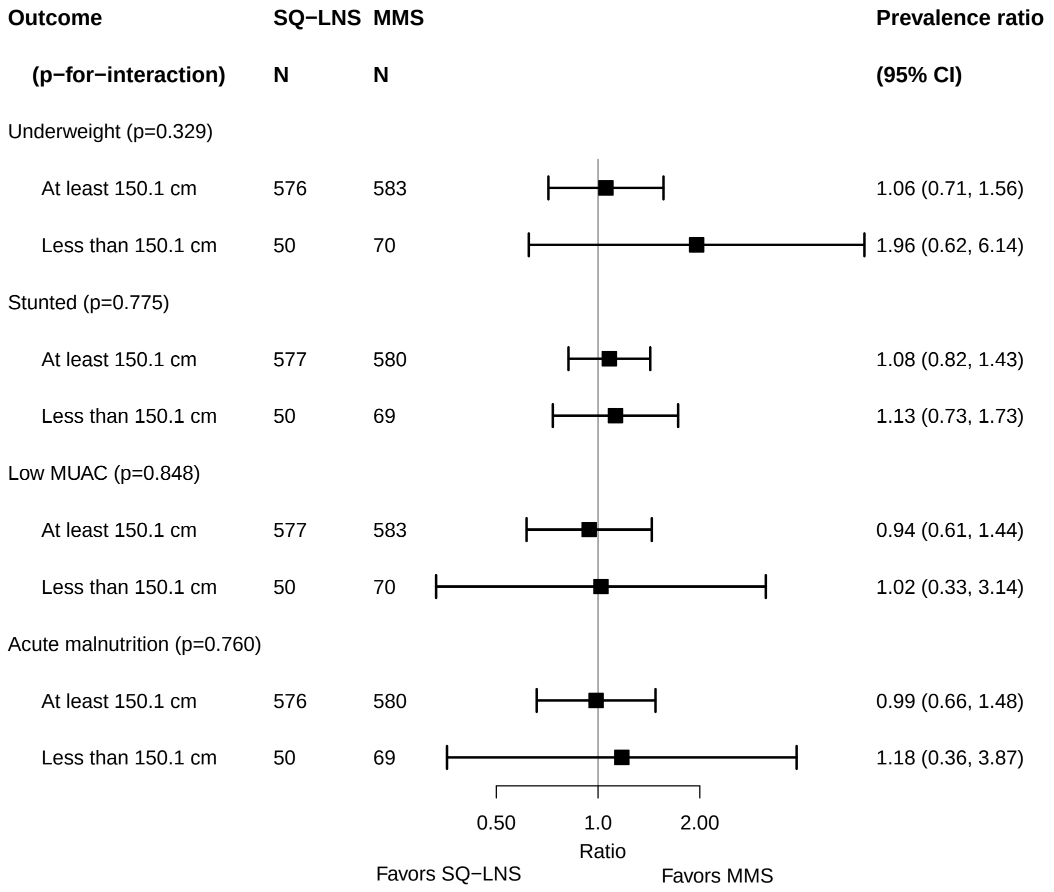

Supplemental figure 6D: Maternal BMI

6D1: Mean differences for birth outcomes

Outcome (p−for−interaction)	SQ−LNS N	MMS N	Mean difference (95% CI)
Birth weight (kg) (p=0.779)			
At least 20 kg/m²	454	434	0.03 (−0.03, 0.09)
Less than 20 kg/m²	234	253	0.02 (−0.06, 0.09)
Weight−for−age z score (p=0.840)			
At least 20 kg/m²	454	434	0.06 (−0.07, 0.19)
Less than 20 kg/m²	234	253	0.05 (−0.13, 0.22)
Weight−for−gestational age z−score (p=0.686)			
At least 20 kg/m²	447	430	0.07 (−0.06, 0.20)
Less than 20 kg/m²	232	252	0.01 (−0.16, 0.18)
Birth length (cm) (p=0.656)			
At least 20 kg/m²	389	382	0.19 (−0.11, 0.50)
Less than 20 kg/m²	165	186	0.09 (−0.32, 0.49)
Length−for−age z−score (p=0.587)			
At least 20 kg/m²	389	382	0.09 (−0.06, 0.25)
Less than 20 kg/m²	165	186	0.04 (−0.18, 0.25)
Length−for−gestational age z−score (p=0.772)			
At least 20 kg/m²	382	378	0.08 (−0.06, 0.23)
Less than 20 kg/m²	163	185	0.05 (−0.17, 0.27)
BMI z−score (p=0.319)			
At least 20 kg/m²	388	377	−0.03 (−0.19, 0.12)
Less than 20 kg/m²	162	179	0.11 (−0.12, 0.34)
Head circumference (cm) (p=0.730)			
At least 20 kg/m²	387	383	0.18 (−0.03, 0.39)
Less than 20 kg/m²	167	186	0.07 (−0.20, 0.33)
Head circumference−for−age z−score (p=0.688)			
At least 20 kg/m²	387	383	0.13 (−0.03, 0.30)
Less than 20 kg/m²	167	186	0.05 (−0.17, 0.26)
Head circumference−for−gestational age z score (p=0.744)			
At least 20 kg/m²	380	379	0.11 (−0.04, 0.26)
Less than 20 kg/m²	165	185	0.11 (−0.10, 0.32)
Mid−upper arm circumference (cm) (p=0.837)			
At least 20 kg/m²	390	383	0.09 (−0.04, 0.22)
Less than 20 kg/m²	167	187	0.06 (−0.11, 0.22)
Duration (wk) (p=0.610)			
At least 20 kg/m²	504	481	−0.02 (−0.31, 0.27)
Less than 20 kg/m²	257	281	−0.13 (−0.56, 0.30)

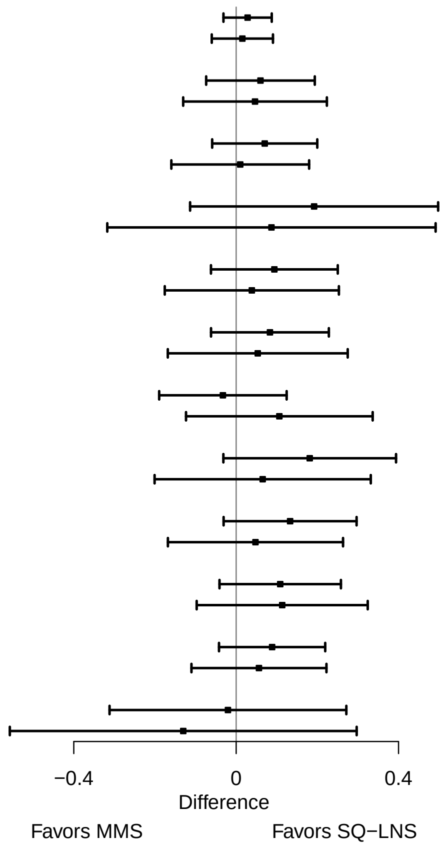

Supplemental figure 6D: Maternal BMI

6D2: Relative risks for birth outcomes

Outcome (p−for−interaction)	SQ−LNS N	MMS N	Relative risk (95% CI)
Low birth weight (p=0.635)			
At least 20 kg/m²	454	434	0.93 (0.64, 1.36)
Less than 20 kg/m²	234	253	0.80 (0.50, 1.28)
Small−for−gestational age (p=0.975)			
At least 20 kg/m²	454	434	0.94 (0.72, 1.23)
Less than 20 kg/m²	234	253	0.98 (0.73, 1.33)
Newborn stunting (p=0.481)			
At least 20 kg/m²	389	382	0.98 (0.66, 1.47)
Less than 20 kg/m²	165	186	1.28 (0.74, 2.23)
Low LGAZ (p=0.571)			
At least 20 kg/m²	389	382	1.12 (0.69, 1.82)
Less than 20 kg/m²	165	186	1.43 (0.84, 2.44)
Low BMIZ (p=0.143)			
At least 20 kg/m²	388	377	1.16 (0.67, 2.04)
Less than 20 kg/m²	162	179	0.69 (0.32, 1.49)
Low HCZ (p=0.549)			
At least 20 kg/m²	387	383	1.40 (0.76, 2.56)
Less than 20 kg/m²	167	186	1.02 (0.40, 2.63)
Low HCGAZ (p=0.764)			
At least 20 kg/m²	387	383	0.74 (0.26, 2.13)
Less than 20 kg/m²	167	186	1.16 (0.22, 6.22)
Preterm birth (p=0.471)			
At least 20 kg/m²	504	481	1.14 (0.77, 1.69)
Less than 20 kg/m²	257	281	1.39 (0.79, 2.44)

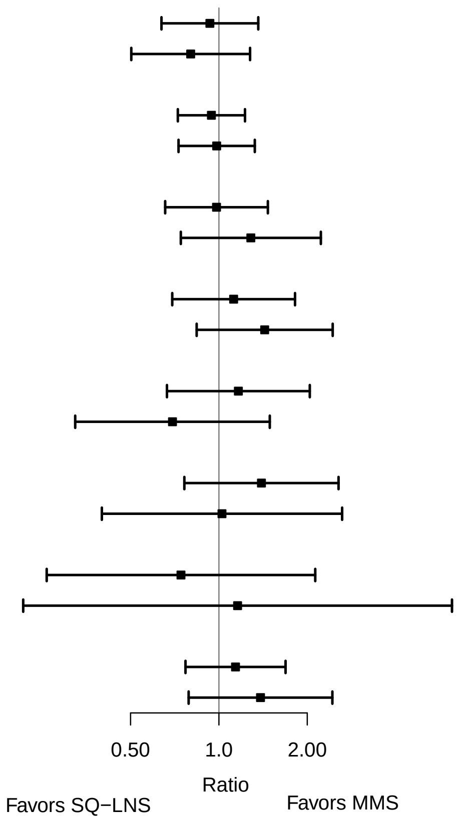

Supplemental figure 6D: Maternal BMI

6D3: Mean differences for 6 mo outcomes

Outcome (p−for−interaction)	SQ−LNS N	MMS N	Mean difference (95% CI)
Weight−for−age z score (p=0.529)			
At least 20 kg/m²	414	414	0.00 (−0.15, 0.15)
Less than 20 kg/m²	211	238	−0.08 (−0.29, 0.13)
Length−for−age z−score (p=0.824)			
At least 20 kg/m²	414	411	0.00 (−0.14, 0.15)
Less than 20 kg/m²	212	237	0.02 (−0.19, 0.22)
Weight−for−length z−score (p=0.295)			
At least 20 kg/m²	414	411	−0.01 (−0.16, 0.14)
Less than 20 kg/m²	211	237	−0.14 (−0.35, 0.08)
Head circumference−for−age z−score (p=0.493)			
At least 20 kg/m²	414	414	−0.03 (−0.16, 0.09)
Less than 20 kg/m²	212	238	0.01 (−0.17, 0.20)
MUAC−for−age z−score (p=0.070)			
At least 20 kg/m²	414	414	0.04 (−0.10, 0.18)
Less than 20 kg/m²	212	238	−0.16 (−0.36, 0.04)

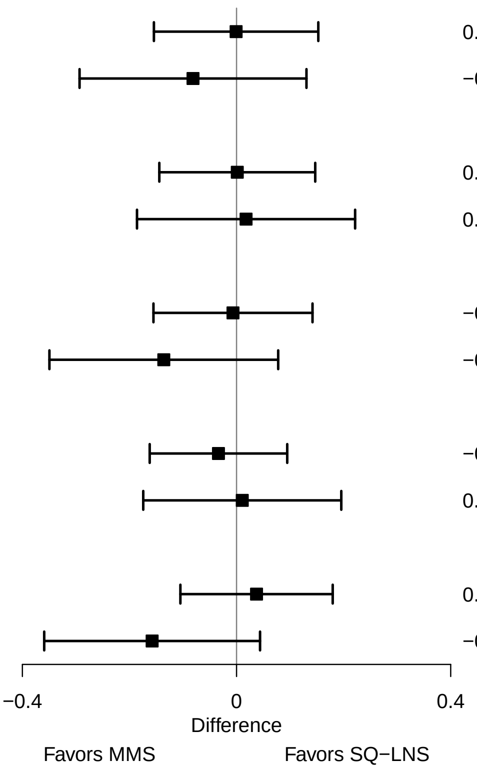

Supplemental figure 6D: Maternal BMI

6D4: Prevalence ratios for 6 mo outcomes

Outcome (p−for−interaction)	SQ−LNS N	MMS N	Prevalence ratio (95% CI)
Underweight (p=0.509)			
At least 20 kg/m²	414	414	1.01 (0.59, 1.76)
Less than 20 kg/m²	211	238	1.32 (0.78, 2.24)
Stunted (p=0.872)			
At least 20 kg/m²	414	411	1.09 (0.79, 1.52)
Less than 20 kg/m²	212	237	0.98 (0.68, 1.41)
Low HCAZ (p=0.737)			
At least 20 kg/m²	414	414	0.72 (0.42, 1.25)
Less than 20 kg/m²	212	238	0.83 (0.46, 1.52)
Low MUAC (p=0.369)			
At least 20 kg/m²	414	414	0.84 (0.47, 1.50)
Less than 20 kg/m²	212	238	1.08 (0.62, 1.89)
Acute malnutrition (p=0.327)			
At least 20 kg/m²	414	411	0.88 (0.49, 1.56)
Less than 20 kg/m²	211	237	1.18 (0.70, 1.98)

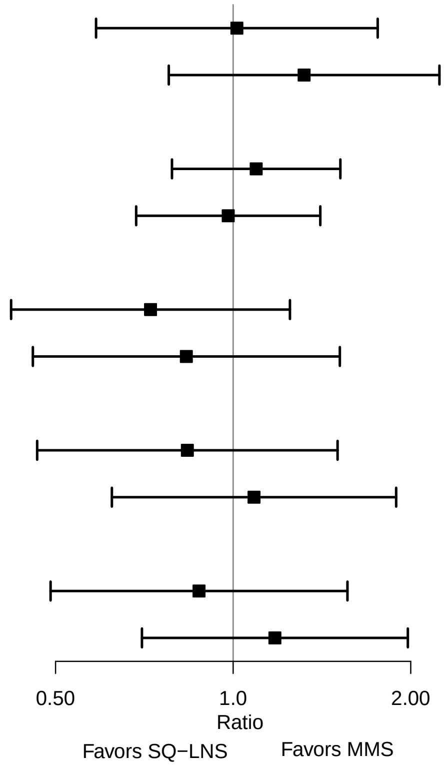

Supplemental figure 6E: Maternal age

6E1: Mean differences for birth outcomes

Outcome (p−for−interaction)	SQ−LNS N	MMS N	Mean difference (95% CI)
Birth weight (kg) (p=0.143)			
At least 25 y	381	396	0.00 (−0.06, 0.06)
Less than 25 y	313	301	0.07 (0.00, 0.13)
Weight−for−age z score (p=0.130)			
At least 25 y	381	396	−0.01 (−0.15, 0.14)
Less than 25 y	313	301	0.16 (0.01, 0.31)
Weight−for−gestational age z−score (p=0.795)			
At least 25 y	379	392	0.05 (−0.09, 0.19)
Less than 25 y	306	300	0.07 (−0.08, 0.22)
Birth length (cm) (p=0.222)			
At least 25 y	327	344	0.04 (−0.27, 0.35)
Less than 25 y	233	233	0.33 (−0.06, 0.71)
Length−for−age z−score (p=0.225)			
At least 25 y	327	344	0.01 (−0.15, 0.17)
Less than 25 y	233	233	0.16 (−0.04, 0.36)
Length−for−gestational age z−score (p=0.479)			
At least 25 y	325	340	0.04 (−0.11, 0.19)
Less than 25 y	226	232	0.13 (−0.06, 0.33)
BMI z−score (p=0.892)			
At least 25 y	326	336	0.01 (−0.16, 0.18)
Less than 25 y	230	229	0.03 (−0.18, 0.23)
Head circumference (cm) (p=0.173)			
At least 25 y	327	345	0.06 (−0.16, 0.27)
Less than 25 y	233	233	0.26 (0.00, 0.52)
Head circumference−for−age z−score (p=0.191)			
At least 25 y	327	345	0.04 (−0.13, 0.21)
Less than 25 y	233	233	0.20 (0.00, 0.40)
Head circumference−for−gestational age z score (p=0.347)			
At least 25 y	325	341	0.07 (−0.09, 0.23)
Less than 25 y	226	232	0.19 (−0.01, 0.38)
Mid−upper arm circumference (cm) (p=0.563)			
At least 25 y	330	345	0.06 (−0.08, 0.20)
Less than 25 y	233	234	0.12 (−0.03, 0.26)
Duration (wk) (p=0.206)			
At least 25 y	414	425	−0.17 (−0.48, 0.14)
Less than 25 y	353	347	0.14 (−0.23, 0.52)

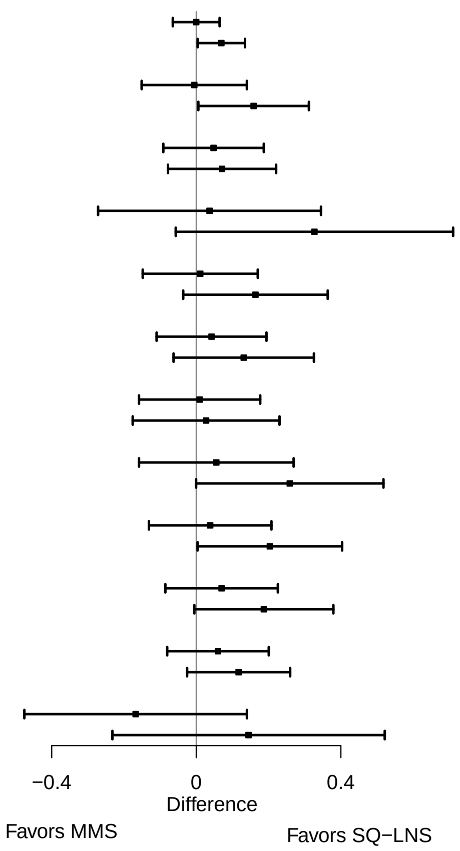

Supplemental figure 6E: Maternal age

6E2: Relative risks for birth outcomes

Outcome (p−for−interaction)	SQ−LNS N	MMS N	Relative risk (95% CI)
Low birth weight (p=0.272)			
At least 25 y	381	396	1.03 (0.68, 1.56)
Less than 25 y	313	301	0.75 (0.49, 1.13)
Birth weight < 2 kg (p=0.684)			
At least 25 y	381	396	1.18 (0.42, 3.28)
Less than 25 y	313	301	0.97 (0.34, 2.76)
Small−for−gestational age (p=0.039)			
At least 25 y	381	396	1.19 (0.89, 1.59)
Less than 25 y	313	301	0.79 (0.61, 1.03)
Large−for−gestational age (p=0.984)			
At least 25 y	381	396	1.12 (0.51, 2.46)
Less than 25 y	313	301	1.20 (0.48, 3.02)
Newborn stunting (p=0.828)			
At least 25 y	327	344	1.09 (0.67, 1.77)
Less than 25 y	233	233	1.04 (0.68, 1.59)
Low LGAZ (p=0.680)			
At least 25 y	327	344	1.12 (0.68, 1.86)
Less than 25 y	233	233	1.31 (0.80, 2.17)
Low BMIZ (p=0.268)			
At least 25 y	326	336	1.22 (0.65, 2.29)
Less than 25 y	230	229	0.86 (0.46, 1.60)
Low HCZ (p=0.296)			
At least 25 y	327	345	1.58 (0.82, 3.06)
Less than 25 y	233	233	0.91 (0.41, 2.02)
Preterm birth (p=0.313)			
At least 25 y	414	425	1.48 (0.92, 2.38)
Less than 25 y	353	347	0.99 (0.64, 1.54)

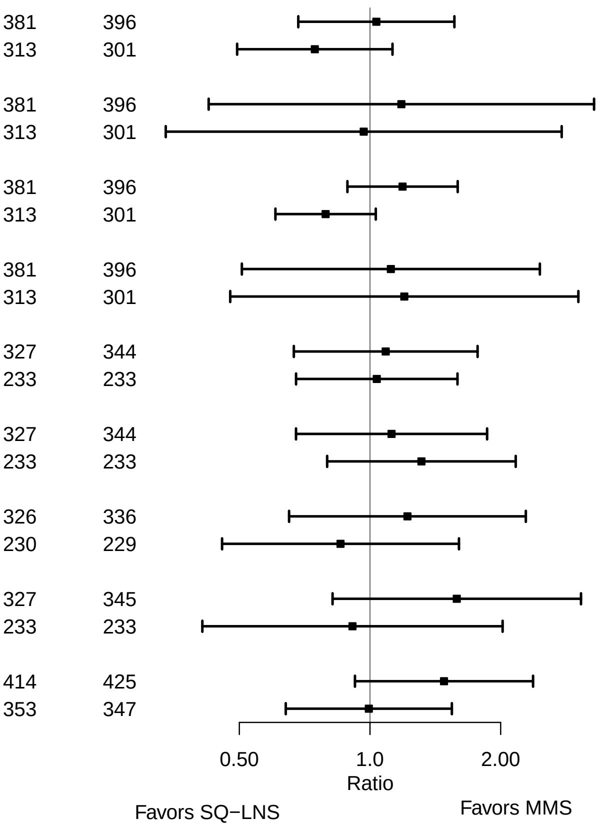

Supplemental figure 6E: Maternal age

6E3: Mean differences for 6 mo outcomes

Outcome (p−for−interaction)	SQ−LNS N	MMS N	Mean difference (95% CI)
Weight−for−age z score (p=0.397)			
At least 25 y	341	379	−0.06 (−0.23, 0.11)
Less than 25 y	290	282	0.05 (−0.13, 0.23)
Length−for−age z−score (p=0.707)			
At least 25 y	342	376	0.00 (−0.16, 0.16)
Less than 25 y	290	281	0.04 (−0.13, 0.21)
Weight−for−length z−score (p=0.426)			
At least 25 y	341	376	−0.08 (−0.25, 0.08)
Less than 25 y	290	281	0.02 (−0.16, 0.20)
Head circumference−for−age z−score (p=0.404)			
At least 25 y	342	379	0.02 (−0.12, 0.16)
Less than 25 y	290	282	−0.08 (−0.24, 0.08)
MUAC−for−age z−score (p=0.341)			
At least 25 y	342	379	−0.08 (−0.23, 0.08)
Less than 25 y	290	282	0.04 (−0.13, 0.22)

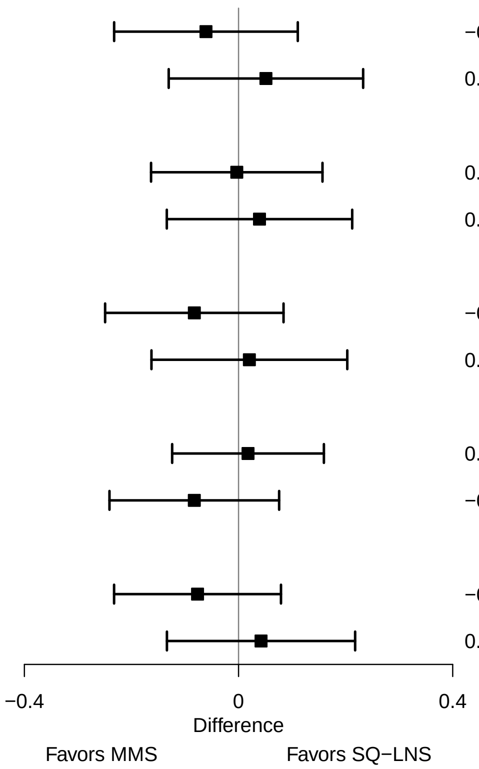

Supplemental figure 6E: Maternal age

6E4: Prevalence ratios for 6 mo outcomes

Outcome (p−for−interaction)	SQ−LNS N	MMS N	Prevalence ratio (95% CI)
Underweight (p=0.651)			
At least 25 y	341	379	1.22 (0.77, 1.92)
Less than 25 y	290	282	1.05 (0.56, 1.98)
Stunted (p=0.311)			
At least 25 y	342	376	1.18 (0.87, 1.60)
Less than 25 y	290	281	0.90 (0.60, 1.33)
Wasted (p=0.742)			
At least 25 y	341	376	1.00 (0.40, 2.53)
Less than 25 y	290	281	0.97 (0.32, 2.97)
Low HCAZ (p=0.044)			
At least 25 y	342	379	0.53 (0.30, 0.92)
Less than 25 y	290	282	1.23 (0.68, 2.25)
Low MUAC (p=0.432)			
At least 25 y	342	379	0.83 (0.50, 1.39)
Less than 25 y	290	282	1.09 (0.58, 2.05)
Acute malnutrition (p=0.482)			
At least 25 y	341	376	0.90 (0.55, 1.48)
Less than 25 y	290	281	1.12 (0.62, 2.03)

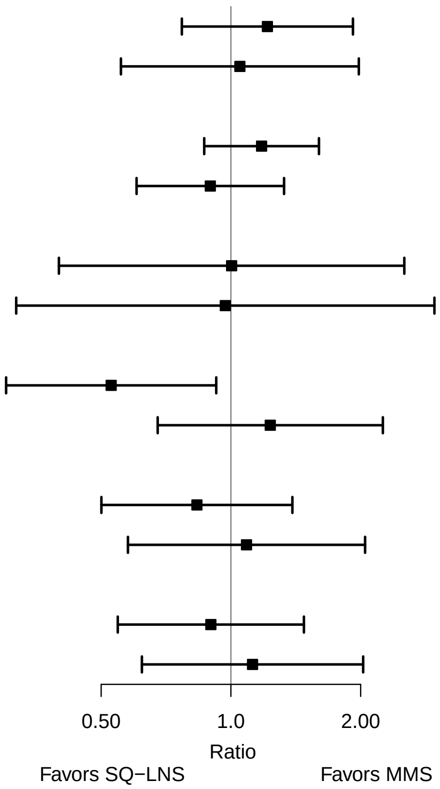

Supplemental figure 6F: Maternal education

6F1: Mean differences for birth outcomes

Outcome (p−for−interaction)	SQ−LNS N	MMS N	Mean difference (95% CI)
Birth weight (kg) (p=0.391)			
Primary or greater	305	318	0.01 (−0.06, 0.08)
Incomplete or no formal	386	377	0.04 (−0.02, 0.11)
Weight−for−age z score (p=0.338)			
Primary or greater	305	318	0.02 (−0.13, 0.18)
Incomplete or no formal	386	377	0.10 (−0.04, 0.24)
Weight−for−gestational age z−score (p=0.729)			
Primary or greater	299	314	0.06 (−0.09, 0.21)
Incomplete or no formal	383	376	0.06 (−0.08, 0.20)
Birth length (cm) (p=0.637)			
Primary or greater	287	295	0.22 (−0.14, 0.58)
Incomplete or no formal	271	280	0.10 (−0.23, 0.43)
Length−for−age z−score (p=0.650)			
Primary or greater	287	295	0.10 (−0.09, 0.28)
Incomplete or no formal	271	280	0.05 (−0.12, 0.22)
Length−for−gestational age z−score (p=0.442)			
Primary or greater	281	291	0.10 (−0.07, 0.27)
Incomplete or no formal	268	279	0.05 (−0.12, 0.21)
BMI z−score (p=0.252)			
Primary or greater	286	294	−0.03 (−0.21, 0.16)
Incomplete or no formal	268	270	0.08 (−0.10, 0.26)
Head circumference (cm) (p=0.927)			
Primary or greater	287	295	0.20 (−0.04, 0.45)
Incomplete or no formal	271	281	0.10 (−0.13, 0.33)
Head circumference−for−age z−score (p=0.991)			
Primary or greater	287	295	0.14 (−0.05, 0.33)
Incomplete or no formal	271	281	0.08 (−0.10, 0.25)
Head circumference−for−gestational age z score (p=0.816)			
Primary or greater	281	291	0.17 (−0.01, 0.34)
Incomplete or no formal	268	280	0.07 (−0.10, 0.24)
Mid−upper arm circumference (cm) (p=0.835)			
Primary or greater	287	295	0.10 (−0.05, 0.25)
Incomplete or no formal	274	282	0.07 (−0.07, 0.21)
Duration (wk) (p=0.978)			
Primary or greater	329	343	−0.04 (−0.35, 0.27)
Incomplete or no formal	433	421	−0.05 (−0.39, 0.30)

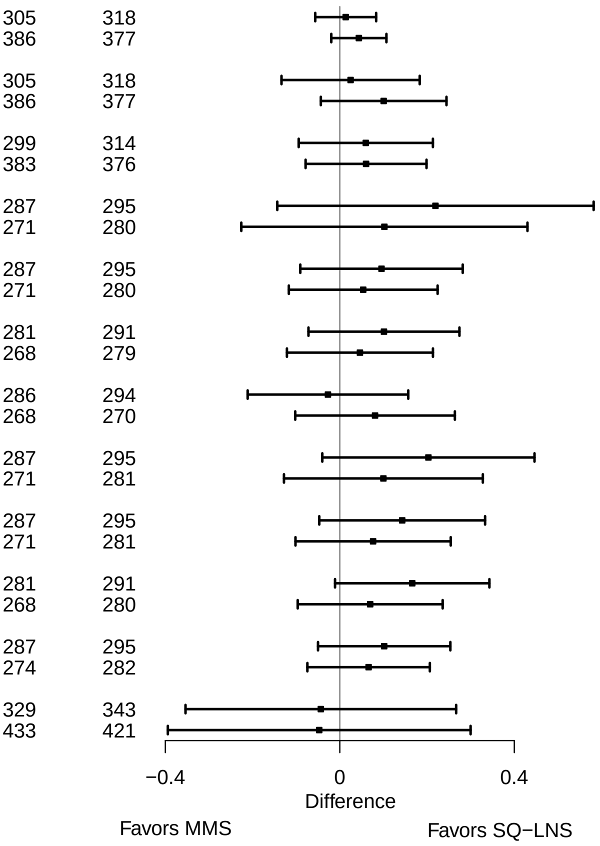

Supplemental figure 6F: Maternal education

6F2: Relative risks for birth outcomes

Outcome (p−for−interaction)	SQ−LNS N	MMS N	Relative risk (95% CI)
Low birth weight (p=0.791)			
Primary or greater	305	318	0.91 (0.58, 1.43)
Incomplete or no formal	386	377	0.87 (0.59, 1.27)
Small−for−gestational age (p=0.623)			
Primary or greater	305	318	0.97 (0.72, 1.31)
Incomplete or no formal	386	377	0.95 (0.73, 1.24)
Large−for−gestational age (p=0.842)			
Primary or greater	305	318	1.04 (0.46, 2.36)
Incomplete or no formal	386	377	1.32 (0.56, 3.11)
Newborn stunting (p=0.448)			
Primary or greater	287	295	0.90 (0.56, 1.46)
Incomplete or no formal	271	280	1.23 (0.79, 1.90)
Low LGAZ (p=0.381)			
Primary or greater	287	295	1.31 (0.69, 2.45)
Incomplete or no formal	271	280	1.11 (0.72, 1.73)
Low BMIZ (p=0.569)			
Primary or greater	286	294	0.91 (0.50, 1.63)
Incomplete or no formal	268	270	1.06 (0.55, 2.06)
Low HCZ (p=0.316)			
Primary or greater	287	295	1.03 (0.52, 2.03)
Incomplete or no formal	271	281	1.67 (0.77, 3.61)
Preterm birth (p=0.708)			
Primary or greater	329	343	1.29 (0.78, 2.15)
Incomplete or no formal	433	421	1.15 (0.76, 1.76)

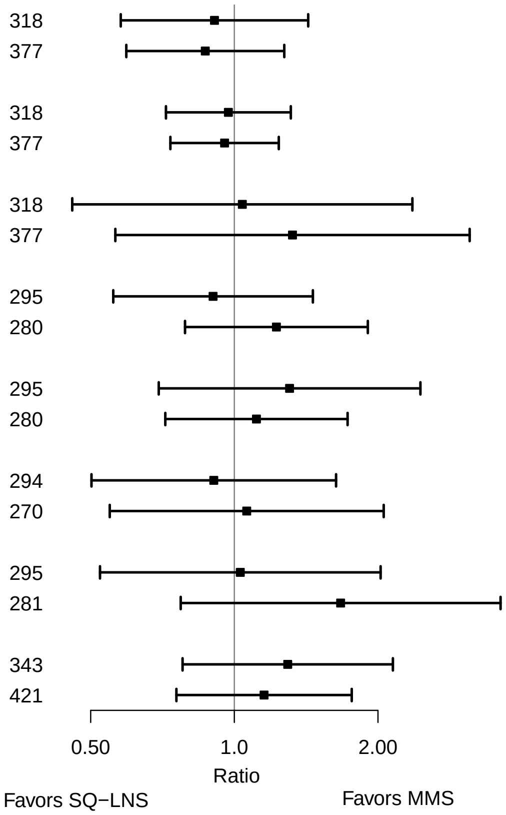

Supplemental figure 6F: Maternal education

6F3: Mean differences for 6 mo outcomes

Outcome (p−for−interaction)	SQ−LNS N	MMS N	Mean difference (95% CI)
Weight−for−age z score (p=0.127)			
Primary or greater	277	292	−0.09 (−0.27, 0.09)
Incomplete or no formal	353	367	0.05 (−0.12, 0.22)
Length−for−age z−score (p=0.289)			
Primary or greater	278	291	−0.02 (−0.19, 0.15)
Incomplete or no formal	353	364	0.05 (−0.11, 0.21)
Weight−for−length z−score (p=0.210)			
Primary or greater	277	291	−0.11 (−0.29, 0.07)
Incomplete or no formal	353	364	0.03 (−0.14, 0.20)
Head circumference−for−age z−score (p=0.295)			
Primary or greater	278	292	−0.04 (−0.19, 0.11)
Incomplete or no formal	353	367	0.00 (−0.14, 0.15)
MUAC−for−age z−score (p=0.105)			
Primary or greater	278	292	−0.12 (−0.28, 0.05)
Incomplete or no formal	353	367	0.07 (−0.09, 0.23)

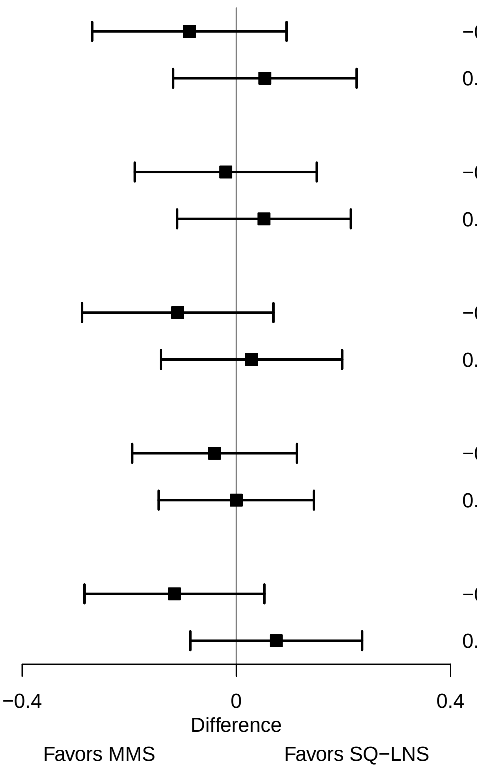

Supplemental figure 6F: Maternal education

6F4: Prevalence ratios for 6 mo outcomes

Outcome (p−for−interaction)	SQ−LNS N	MMS N	Prevalence ratio (95% CI)
Underweight (p=0.931)			
Primary or greater	277	292	1.08 (0.56, 2.11)
Incomplete or no formal	353	367	1.13 (0.73, 1.76)
Stunted (p=0.714)			
Primary or greater	278	291	1.26 (0.78, 2.03)
Incomplete or no formal	353	364	1.00 (0.75, 1.32)
Wasted (p=0.946)			
Primary or greater	277	291	1.26 (0.39, 4.07)
Incomplete or no formal	353	364	0.83 (0.34, 2.01)
Low HCAZ (p=0.703)			
Primary or greater	278	292	0.82 (0.45, 1.50)
Incomplete or no formal	353	367	0.75 (0.44, 1.27)
Acute malnutrition (p=0.709)			
Primary or greater	277	291	0.97 (0.53, 1.79)
Incomplete or no formal	353	364	0.97 (0.60, 1.58)

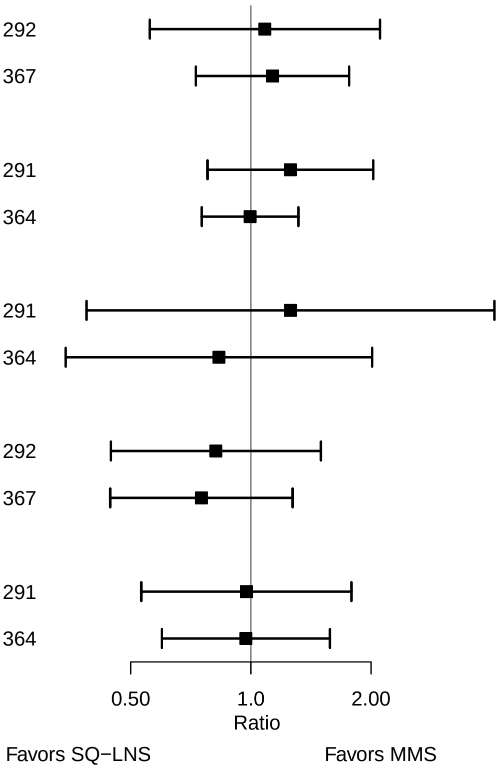

Supplemental figure 6G: Baseline anemia status

6G1: Mean differences for birth outcomes

**Outcome (p−for−interaction)**	**SQ−LNS N**	**MMS N**	**Mean difference (95% CI)**
Birth weight (kg) (p=0.577)			
Anemia	282	298	0.05 (−0.03, 0.12)
No anemia	412	398	0.02 (−0.04, 0.08)
Weight−for−age z score (p=0.486)			
Anemia	282	298	0.11 (−0.06, 0.29)
No anemia	412	398	0.03 (−0.10, 0.17)
Weight−for−gestational age z−score (p=0.623)			
Anemia	277	296	0.03 (−0.13, 0.19)
No anemia	408	395	0.08 (−0.06, 0.21)
Birth length (cm) (p=0.497)			
Anemia	221	244	0.07 (−0.32, 0.46)
No anemia	339	332	0.23 (−0.08, 0.54)
Length−for−age z−score (p=0.601)			
Anemia	221	244	0.04 (−0.16, 0.24)
No anemia	339	332	0.10 (−0.06, 0.26)
Length−for−gestational age z−score (p=0.322)			
Anemia	216	242	0.01 (−0.18, 0.21)
No anemia	335	329	0.13 (−0.02, 0.29)
BMI z−score (p=0.207)			
Anemia	218	236	0.12 (−0.10, 0.33)
No anemia	338	328	−0.06 (−0.22, 0.11)
Head circumference (cm) (p=0.798)			
Anemia	221	244	0.14 (−0.11, 0.40)
No anemia	339	333	0.16 (−0.06, 0.38)
Head circumference−for−age z−score (p=0.922)			
Anemia	221	244	0.12 (−0.08, 0.32)
No anemia	339	333	0.11 (−0.06, 0.28)
Head circumference−for−gestational age z score (p=0.560)			
Anemia	216	242	0.08 (−0.11, 0.27)
No anemia	335	330	0.14 (−0.02, 0.30)
Mid−upper arm circumference (cm) (p=0.747)			
Anemia	222	244	0.07 (−0.09, 0.24)
No anemia	341	334	0.10 (−0.03, 0.23)
Duration (wk) (p=0.660)			
Anemia	318	334	−0.11 (−0.50, 0.28)
No anemia	449	437	0.00 (−0.30, 0.30)

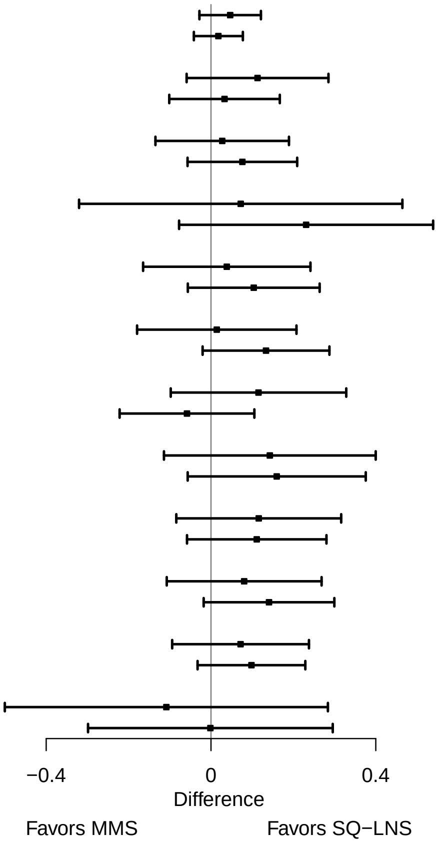

Supplemental figure 6G: Baseline anemia status

6G2: Relative risks for birth outcomes

Outcome (p−for−interaction)	SQ−LNS N	MMS N	Relative risk (95% CI)
Low birth weight (p=0.740)			
Anemia	282	298	0.84 (0.55, 1.28)
No anemia	412	398	0.92 (0.61, 1.39)
Birth weight < 2 kg (p=0.547)			
Anemia	282	298	0.96 (0.38, 2.45)
No anemia	412	398	1.47 (0.42, 5.18)
Small−for−gestational age (p=0.391)			
Anemia	282	298	0.88 (0.65, 1.19)
No anemia	412	398	1.05 (0.80, 1.36)
Large−for−gestational age (p=0.611)			
Anemia	282	298	0.96 (0.38, 2.46)
No anemia	412	398	1.29 (0.60, 2.78)
Newborn stunting (p=0.664)			
Anemia	221	244	1.16 (0.73, 1.84)
No anemia	339	332	0.98 (0.61, 1.58)
Low LGAZ (p=0.168)			
Anemia	221	244	1.61 (0.91, 2.86)
No anemia	339	332	0.97 (0.60, 1.57)
Low BMIZ (p=0.245)			
Anemia	218	236	0.77 (0.38, 1.52)
No anemia	338	328	1.31 (0.71, 2.42)
Low HCZ (p=0.235)			
Anemia	221	244	0.89 (0.41, 1.95)
No anemia	339	333	1.73 (0.82, 3.64)
Preterm birth (p=0.636)			
Anemia	318	334	1.10 (0.73, 1.67)
No anemia	449	437	1.36 (0.83, 2.23)

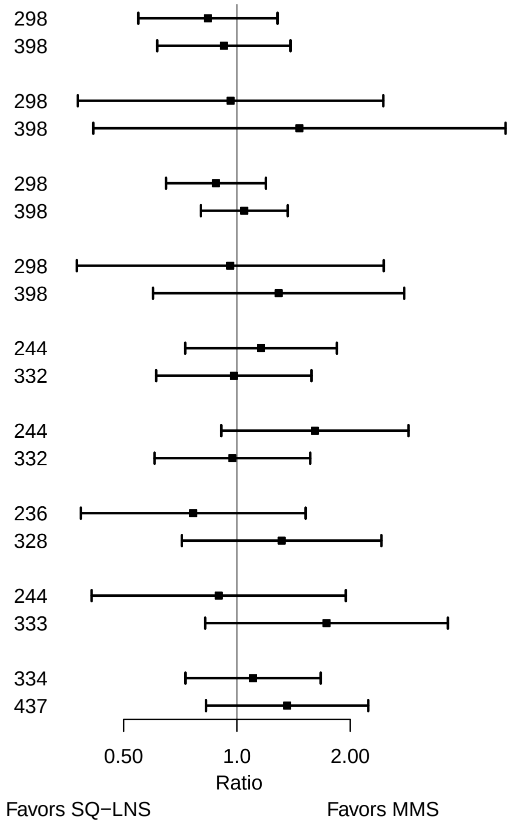

Supplemental figure 6G: Baseline anemia status

6G3: Mean differences for 6 mo outcomes

**Outcome (p−for−interaction)**	**SQ−LNS N**	**MMS N**	**Mean difference (95% CI)**
Weight−for−age z score (p=0.767)			
Anemia	258	279	0.01 (−0.19, 0.21)
No anemia	373	381	−0.03 (−0.19, 0.13)
Length−for−age z−score (p=0.551)			
Anemia	259	277	0.06 (−0.12, 0.25)
No anemia	373	379	−0.01 (−0.17, 0.14)
Weight−for−length z−score (p=0.952)			
Anemia	258	277	−0.04 (−0.24, 0.15)
No anemia	373	379	−0.04 (−0.20, 0.12)
Head circumference−for−age z−score (p=0.789)			
Anemia	259	279	−0.05 (−0.22, 0.13)
No anemia	373	381	−0.02 (−0.15, 0.11)
MUAC−for−age z−score (p=0.972)			
Anemia	259	279	−0.02 (−0.21, 0.17)
No anemia	373	381	−0.02 (−0.17, 0.13)

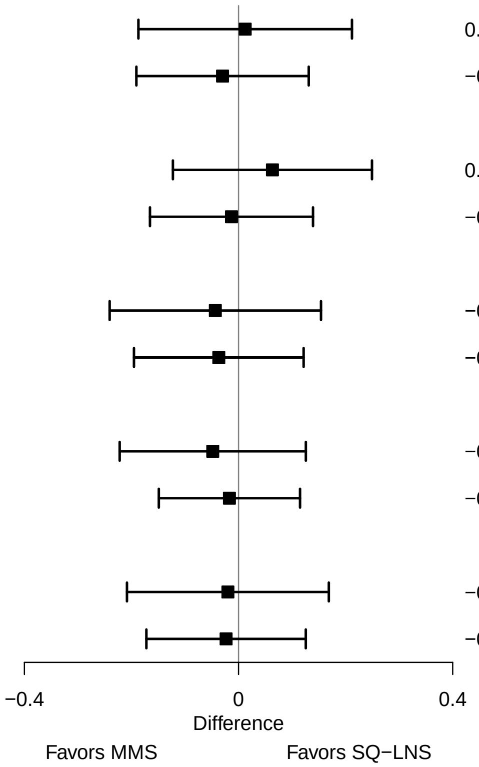

Supplemental figure 6G: Baseline anemia status

6G4: Prevalence ratios for 6 mo outcomes

Outcome (p−for−interaction)	SQ−LNS N	MMS N	Prevalence ratio (95% CI)
Underweight (p=0.216)			
Anemia	258	279	0.89 (0.51, 1.55)
No anemia	373	381	1.42 (0.84, 2.39)
Stunted (p=0.397)			
Anemia	259	277	1.18 (0.83, 1.69)
No anemia	373	379	0.95 (0.69, 1.33)
Low HCAZ (p=0.567)			
Anemia	259	279	0.87 (0.54, 1.41)
No anemia	373	381	0.68 (0.35, 1.32)
Low MUAC (p=0.632)			
Anemia	259	279	1.00 (0.58, 1.72)
No anemia	373	381	0.84 (0.47, 1.51)
Acute malnutrition (p=0.605)			
Anemia	258	277	1.08 (0.64, 1.82)
No anemia	373	379	0.89 (0.50, 1.58)

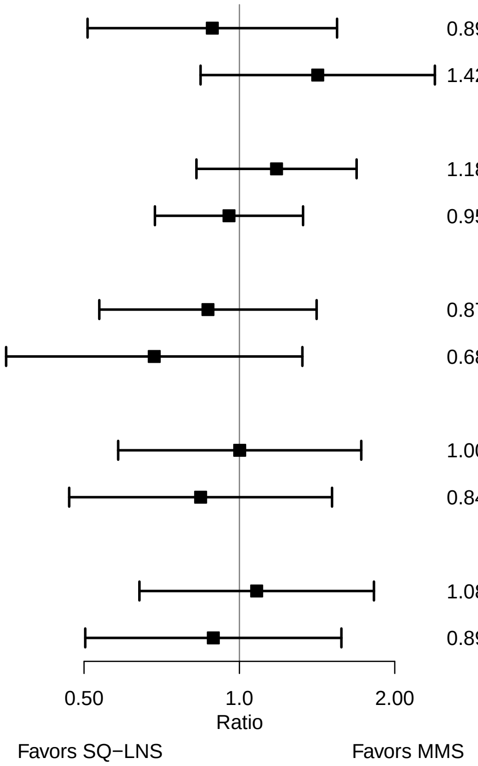

Supplemental figure 6H: Baseline inflammation status

6H1: Mean differences for birth outcomes

Outcome (p−for−interaction)	SQ−LNS N	MMS N	Mean difference (95% CI)
Birth weight (kg) (p=0.080)			
Not inflamed	384	421	0.00 (−0.05, 0.06)
Inflamed	296	267	0.09 (0.01, 0.17)
Weight−for−age z score (p=0.106)			
Not inflamed	384	421	0.01 (−0.12, 0.15)
Inflamed	296	267	0.19 (0.02, 0.37)
Weight−for−gestational age z−score (p=0.704)			
Not inflamed	377	420	0.05 (−0.08, 0.18)
Inflamed	294	263	0.09 (−0.08, 0.25)
Birth length (cm) (p=0.758)			
Not inflamed	317	353	0.17 (−0.12, 0.46)
Inflamed	232	216	0.24 (−0.17, 0.66)
Length−for−age z−score (p=0.992)			
Not inflamed	317	353	0.10 (−0.05, 0.25)
Inflamed	232	216	0.10 (−0.11, 0.31)
Length−for−gestational age z−score (p=0.920)			
Not inflamed	310	352	0.08 (−0.07, 0.23)
Inflamed	230	212	0.07 (−0.13, 0.27)
BMI z−score (p=0.661)			
Not inflamed	314	348	0.05 (−0.12, 0.21)
Inflamed	231	209	−0.01 (−0.23, 0.21)
Head circumference (cm) (p=0.173)			
Not inflamed	316	354	0.26 (0.05, 0.48)
Inflamed	233	216	0.04 (−0.21, 0.30)
Head circumference−for−age z−score (p=0.097)			
Not inflamed	316	354	0.22 (0.05, 0.38)
Inflamed	233	216	0.00 (−0.20, 0.20)
Head circumference−for−gestational age z score (p=0.052)			
Not inflamed	309	353	0.22 (0.06, 0.38)
Inflamed	231	212	−0.02 (−0.21, 0.17)
Mid−upper arm circumference (cm) (p=0.841)			
Not inflamed	317	355	0.11 (−0.01, 0.24)
Inflamed	234	216	0.09 (−0.08, 0.26)
Duration (wk) (p=0.460)			
Not inflamed	426	470	−0.06 (−0.36, 0.24)
Inflamed	325	292	0.12 (−0.27, 0.50)

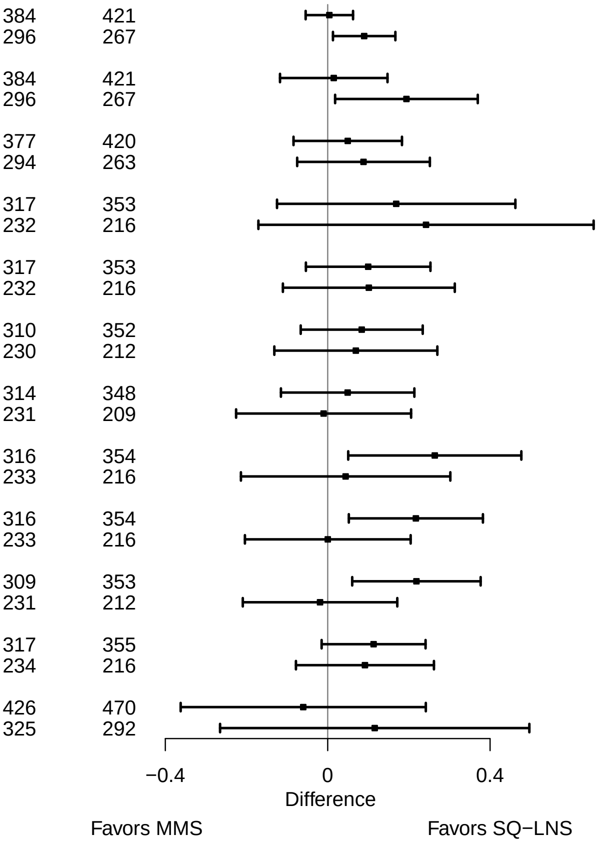

Supplemental figure 6H: Baseline inflammation status

6H2: Relative risks for birth outcomes

Outcome (p−for−interaction)	SQ−LNS N	MMS N	Relative risk (95% CI)
Low birth weight (p=0.220)			
Not inflamed	384	421	0.97 (0.64, 1.47)
Inflamed	296	267	0.65 (0.42, 1.02)
Small−for−gestational age (p=0.880)			
Not inflamed	384	421	0.93 (0.71, 1.22)
Inflamed	296	267	0.97 (0.71, 1.31)
Large−for−gestational age (p=0.287)			
Not inflamed	384	421	0.93 (0.40, 2.17)
Inflamed	296	267	1.54 (0.65, 3.65)
Newborn stunting (p=0.879)			
Not inflamed	317	353	1.00 (0.64, 1.55)
Inflamed	232	216	0.96 (0.57, 1.62)
Low LGAZ (p=0.999)			
Not inflamed	317	353	1.17 (0.71, 1.94)
Inflamed	232	216	1.19 (0.71, 1.99)
Low BMIZ (p=0.912)			
Not inflamed	314	348	0.93 (0.53, 1.63)
Inflamed	231	209	1.03 (0.50, 2.12)
Low HCZ (p=0.165)			
Not inflamed	316	354	1.47 (0.70, 3.06)
Inflamed	233	216	0.66 (0.29, 1.50)
Low HCGAZ (p=0.751)			
Not inflamed	316	354	0.73 (0.21, 2.57)
Inflamed	233	216	1.14 (0.31, 4.23)
Preterm birth (p=0.213)			
Not inflamed	426	470	1.37 (0.88, 2.13)
Inflamed	325	292	0.85 (0.53, 1.39)

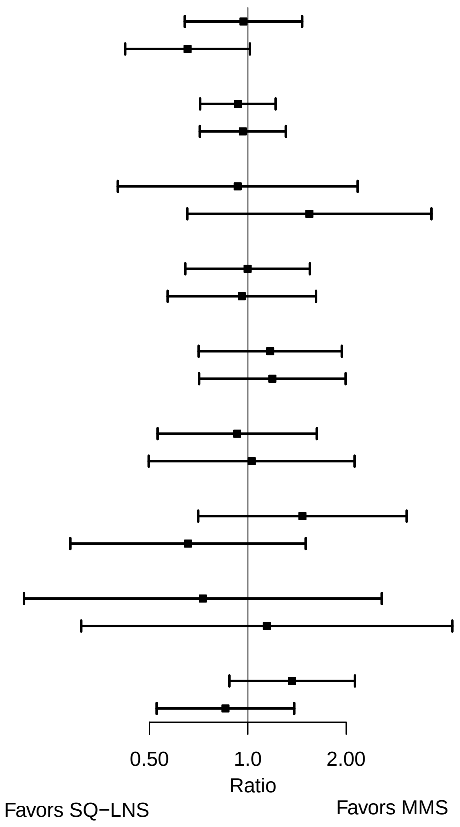

Supplemental figure 6H: Baseline inflammation status

6H3: Mean differences for 6 mo outcomes

Outcome (p−for−interaction)	SQ−LNS N	MMS N	Mean difference (95% CI)
Weight−for−age z score (p=0.686)			
Not inflamed	349	401	−0.01 (−0.17, 0.15)
Inflamed	270	252	0.04 (−0.16, 0.24)
Length−for−age z−score (p=0.421)			
Not inflamed	350	399	0.00 (−0.16, 0.15)
Inflamed	270	250	0.09 (−0.09, 0.28)
Weight−for−length z−score (p=0.843)			
Not inflamed	349	399	−0.02 (−0.18, 0.15)
Inflamed	270	250	−0.05 (−0.24, 0.15)
Head circumference−for−age z−score (p=0.782)			
Not inflamed	350	401	0.00 (−0.13, 0.14)
Inflamed	270	252	−0.03 (−0.20, 0.13)
MUAC−for−age z−score (p=0.838)			
Not inflamed	350	401	−0.01 (−0.16, 0.14)
Inflamed	270	252	0.01 (−0.18, 0.20)

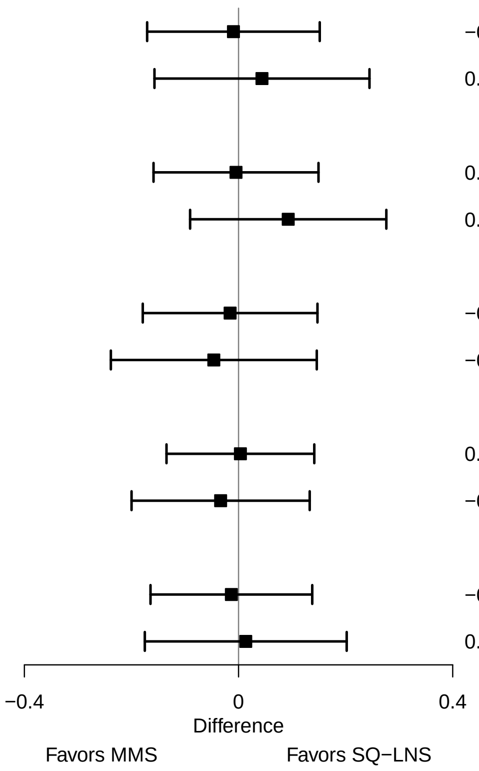

Supplemental figure 6H: Baseline inflammation status

6H4: Prevalence ratios for 6 mo outcomes

**Outcome (p−for−interaction)**	**SQ−LNS N**	**MMS N**	**Prevalence ratio (95% CI)**
Underweight (p=0.160)			
Not inflamed	349	401	1.28 (0.75, 2.19)
Inflamed	270	252	0.75 (0.42, 1.34)
Stunted (p=0.689)			
Not inflamed	350	399	0.98 (0.71, 1.36)
Inflamed	270	250	1.05 (0.72, 1.54)
Wasted (p=0.863)			
Not inflamed	349	399	1.02 (0.39, 2.65)
Inflamed	270	250	0.93 (0.30, 2.89)
Low HCAZ (p=0.984)			
Not inflamed	350	401	0.75 (0.43, 1.31)
Inflamed	270	252	0.75 (0.42, 1.34)
Low MUAC (p=0.092)			
Not inflamed	350	401	1.32 (0.76, 2.30)
Inflamed	270	252	0.62 (0.33, 1.17)
Acute malnutrition (p=0.097)			
Not inflamed	349	399	1.36 (0.80, 2.28)
Inflamed	270	250	0.68 (0.37, 1.24)

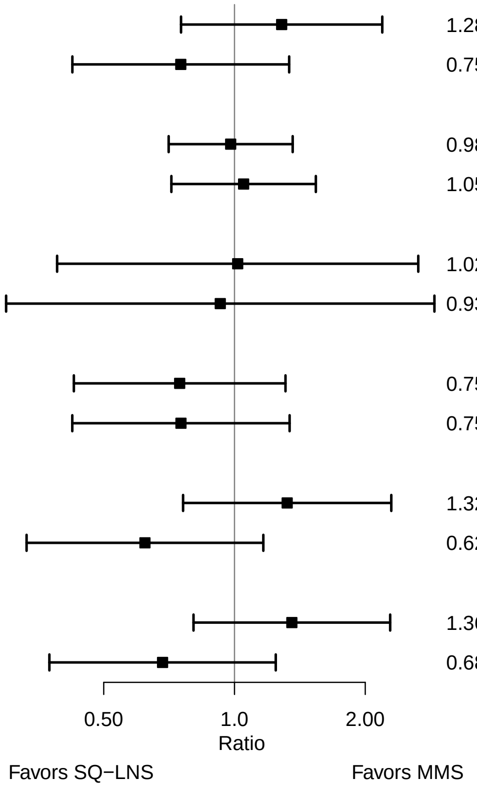

Supplemental figure 6I: Baseline malaria status

6I1: Mean differences for birth outcomes

Outcome (p−for−interaction)	SQ−LNS N	MMS N	Mean difference (95% CI)
Birth weight (kg) (p=0.635)			
No malaria	574	582	0.03 (−0.02, 0.08)
Malaria	118	114	−0.02 (−0.12, 0.09)
Weight−for−age z score (p=0.741)			
No malaria	574	582	0.07 (−0.05, 0.18)
Malaria	118	114	−0.04 (−0.28, 0.20)
Weight−for−gestational age z−score (p=0.078)			
No malaria	567	578	0.10 (−0.02, 0.21)
Malaria	116	113	−0.16 (−0.39, 0.07)
Birth length (cm) (p=0.021)			
No malaria	470	493	0.28 (0.02, 0.54)
Malaria	89	83	−0.51 (−1.17, 0.15)
Length−for−age z−score (p=0.024)			
No malaria	470	493	0.14 (0.00, 0.27)
Malaria	89	83	−0.26 (−0.60, 0.08)
Length−for−gestational age z−score (p=0.001)			
No malaria	463	489	0.17 (0.04, 0.29)
Malaria	87	82	−0.39 (−0.72, −0.06)
BMI z−score (p=0.857)			
No malaria	467	487	0.02 (−0.12, 0.16)
Malaria	88	77	−0.02 (−0.35, 0.30)
Head circumference (cm) (p=0.615)			
No malaria	471	494	0.17 (−0.02, 0.35)
Malaria	88	83	−0.02 (−0.41, 0.36)
Head circumference−for−age z−score (p=0.752)			
No malaria	471	494	0.12 (−0.02, 0.26)
Malaria	88	83	−0.01 (−0.31, 0.29)
Head circumference−for−gestational age z score (p=0.335)			
No malaria	464	490	0.15 (0.01, 0.28)
Malaria	86	82	−0.05 (−0.35, 0.25)
Mid−upper arm circumference (cm) (p=0.195)			
No malaria	473	495	0.11 (0.00, 0.22)
Malaria	89	83	−0.08 (−0.34, 0.17)
Duration (wk) (p=0.588)			
No malaria	629	639	−0.07 (−0.33, 0.19)
Malaria	136	132	0.02 (−0.58, 0.62)

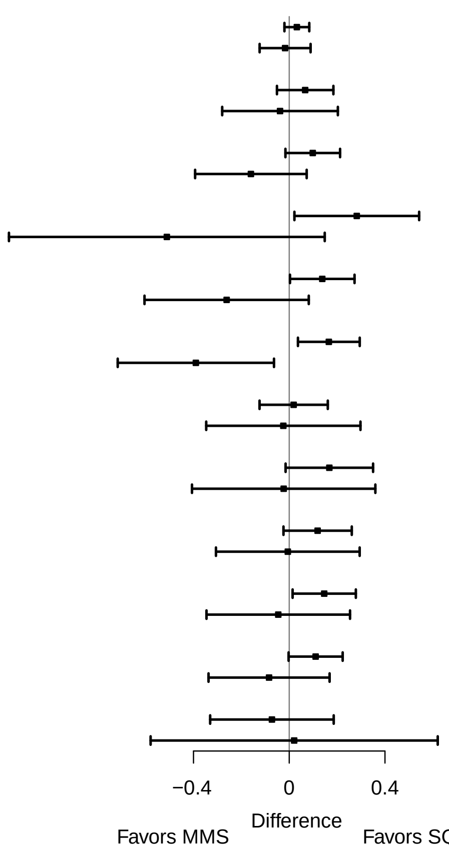

Supplemental figure 6I: Baseline malaria status

6I2: Relative risks for birth outcomes

Outcome (p−for−interaction)	SQ−LNS N	MMS N	Relative risk (95% CI)
Small−for−gestational age (p=0.717)			
No malaria	574	582	0.94 (0.75, 1.17)
Malaria	118	114	1.05 (0.66, 1.65)
Newborn stunting (p=0.005)			
No malaria	470	493	0.83 (0.58, 1.20)
Malaria	89	83	3.19 (1.35, 7.53)
Low LGAZ (p=0.066)			
No malaria	470	493	0.99 (0.66, 1.49)
Malaria	89	83	2.40 (1.06, 5.44)

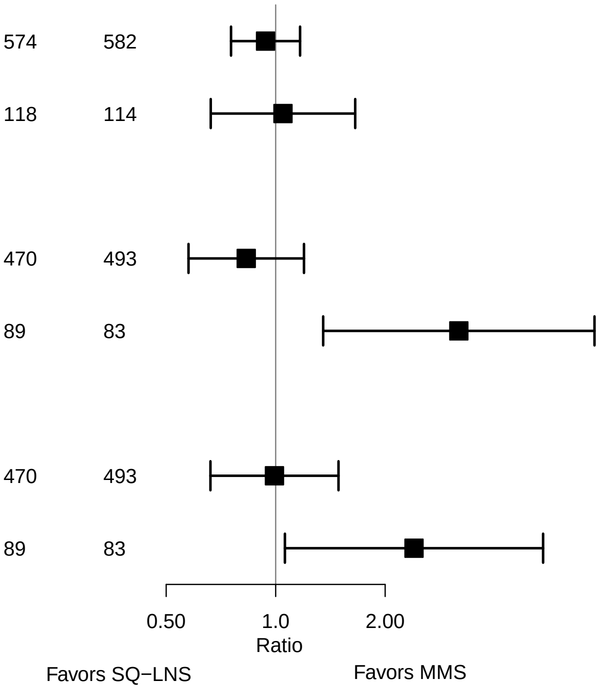

Supplemental figure 6I: Baseline malaria status

6I3: Mean differences for 6 mo outcomes

Outcome (p−for−interaction)	SQ−LNS N	MMS N	Mean difference (95% CI)
Weight−for−age z score (p=0.509)			
No malaria	523	552	0.01 (−0.13, 0.14)
Malaria	106	108	−0.12 (−0.45, 0.20)
Length−for−age z−score (p=0.102)			
No malaria	524	548	0.06 (−0.06, 0.19)
Malaria	106	108	−0.20 (−0.48, 0.09)
Weight−for−length z−score (p=0.768)			
No malaria	523	548	−0.05 (−0.18, 0.09)
Malaria	106	108	0.00 (−0.33, 0.32)
Head circumference−for−age z−score (p=0.529)			
No malaria	524	552	−0.01 (−0.12, 0.11)
Malaria	106	108	−0.12 (−0.38, 0.14)
MUAC−for−age z−score (p=0.952)			
No malaria	524	552	−0.02 (−0.15, 0.11)
Malaria	106	108	−0.02 (−0.31, 0.27)

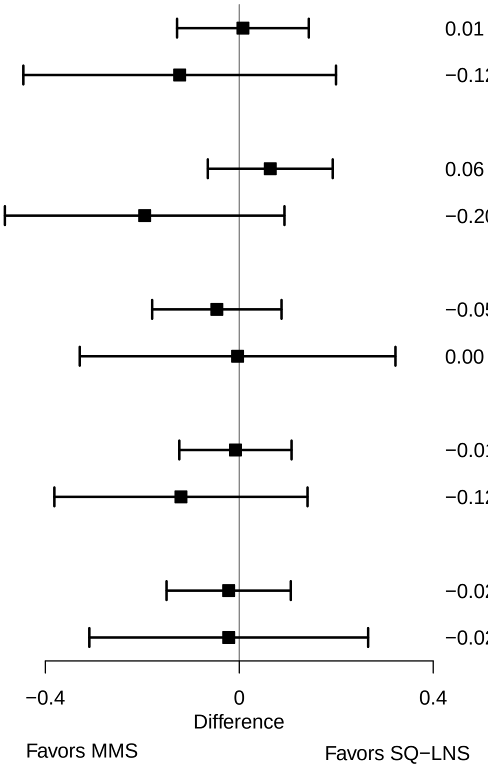

Supplemental figure 6I: Baseline malaria status

6I4: Prevalence ratios for 6 mo outcomes

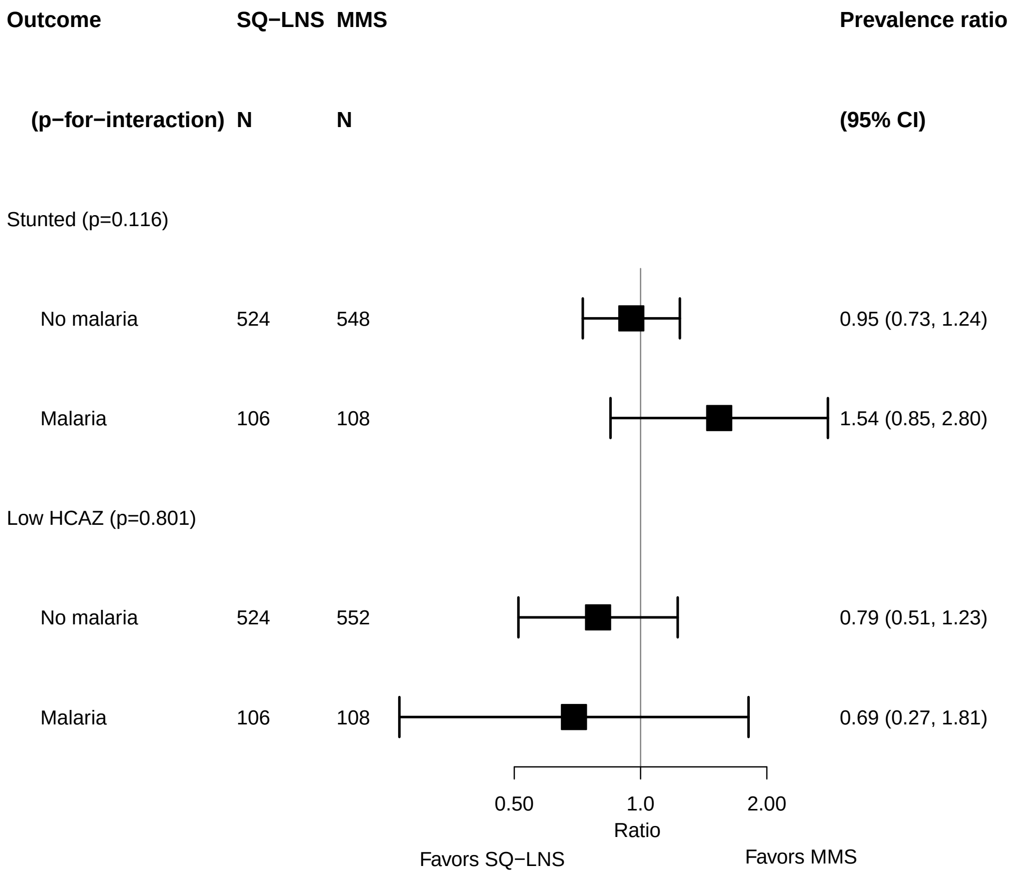

Supplemental figure 6J: Gestational age at supplementation

6J1: Mean differences for birth outcomes

Outcome (p−for−interaction)	SQ−LNS N	MMS N	Mean difference (95% CI)
Birth weight (kg) (p=0.812)			
< 14 wks	120	111	0.04 (−0.07, 0.14)
>= 14 wks	574	586	0.03 (−0.03, 0.08)
Weight−for−age z score (p=0.755)			
< 14 wks	120	111	0.10 (−0.14, 0.33)
>= 14 wks	574	586	0.05 (−0.07, 0.17)
Weight−for−gestational age z−score (p=0.472)			
< 14 wks	115	110	0.15 (−0.09, 0.38)
>= 14 wks	570	582	0.04 (−0.08, 0.15)
Birth length (cm) (p=0.674)			
< 14 wks	105	95	0.11 (−0.43, 0.65)
>= 14 wks	455	482	0.18 (−0.09, 0.45)
Length−for−age z−score (p=0.785)			
< 14 wks	105	95	0.07 (−0.21, 0.35)
>= 14 wks	455	482	0.08 (−0.06, 0.22)
Length−for−gestational age z−score (p=0.898)			
< 14 wks	100	94	0.08 (−0.20, 0.36)
>= 14 wks	451	478	0.08 (−0.05, 0.22)
BMI z−score (p=0.436)			
< 14 wks	105	95	0.13 (−0.17, 0.42)
>= 14 wks	451	470	−0.01 (−0.15, 0.14)
Head circumference (cm) (p=0.903)			
< 14 wks	104	95	0.22 (−0.16, 0.59)
>= 14 wks	456	483	0.14 (−0.04, 0.33)
Head circumference−for−age z−score (p=0.897)			
< 14 wks	104	95	0.19 (−0.11, 0.48)
>= 14 wks	456	483	0.10 (−0.04, 0.24)
Head circumference−for−gestational age z score (p=0.921)			
< 14 wks	99	94	0.17 (−0.11, 0.46)
>= 14 wks	452	479	0.12 (−0.02, 0.25)
Mid−upper arm circumference (cm) (p=0.419)			
< 14 wks	105	95	0.18 (−0.06, 0.42)
>= 14 wks	458	484	0.06 (−0.05, 0.17)
Duration (wk) (p=0.467)			
< 14 wks	135	125	0.19 (−0.37, 0.75)
>= 14 wks	632	647	−0.08 (−0.34, 0.18)

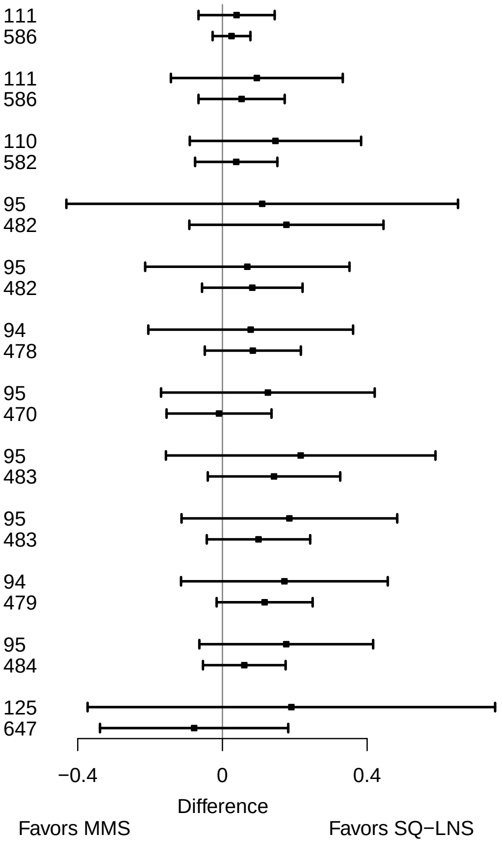

Supplemental figure 6J: Gestational age at supplementation

6J2: Relative risks for birth outcomes

Outcome (p−for−interaction)	SQ−LNS N	MMS N	Relative risk (95% CI)
Low birth weight (p=0.886)			
< 14 wks	120	111	0.92 (0.43, 1.97)
>= 14 wks	574	586	0.87 (0.64, 1.20)
Small−for−gestational age (p=0.830)			
< 14 wks	120	111	1.01 (0.62, 1.65)
>= 14 wks	574	586	0.96 (0.77, 1.19)
Newborn stunting (p=0.386)			
< 14 wks	105	95	1.53 (0.62, 3.75)
>= 14 wks	455	482	1.00 (0.71, 1.42)
Low LGAZ (p=0.341)			
< 14 wks	105	95	1.77 (0.77, 4.08)
>= 14 wks	455	482	1.11 (0.75, 1.65)
Low BMIZ (p=0.672)			
< 14 wks	105	95	0.86 (0.30, 2.46)
>= 14 wks	451	470	1.01 (0.61, 1.64)
Low HCZ (p=0.881)			
< 14 wks	104	95	1.43 (0.34, 6.10)
>= 14 wks	456	483	1.24 (0.72, 2.13)
Low HCGAZ (p=0.990)			
< 14 wks	104	95	0.91 (0.17, 4.86)
>= 14 wks	456	483	0.80 (0.28, 2.29)
Preterm birth (p=0.399)			
< 14 wks	135	125	1.01 (0.47, 2.14)
>= 14 wks	632	647	1.25 (0.87, 1.78)

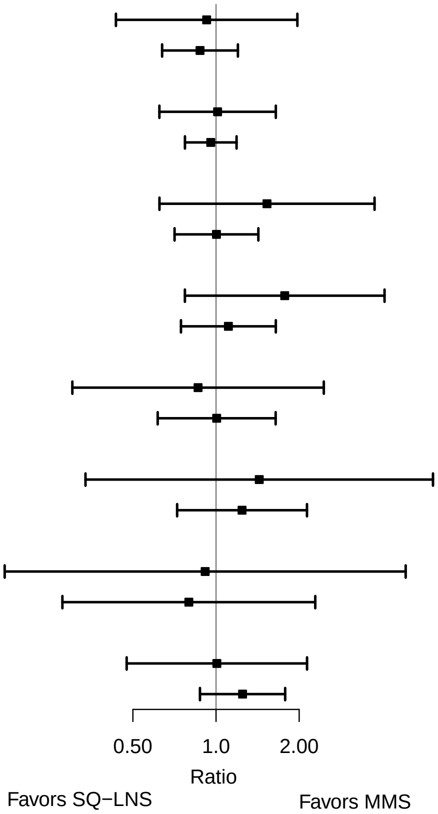

Supplemental figure 6J: Gestational age at supplementation

6J3: Mean differences for 6 mo outcomes

Outcome (p−for−interaction)	SQ−LNS N	MMS N	Mean difference (95% CI)
Weight−for−age z score (p=0.256)			
< 14 wks	114	111	0.15 (−0.16, 0.46)
>= 14 wks	517	550	−0.05 (−0.18, 0.09)
Length−for−age z−score (p=0.581)			
< 14 wks	114	111	0.09 (−0.20, 0.37)
>= 14 wks	518	546	0.00 (−0.13, 0.13)
Weight−for−length z−score (p=0.262)			
< 14 wks	114	111	0.11 (−0.19, 0.42)
>= 14 wks	517	546	−0.07 (−0.20, 0.07)
Head circumference−for−age z−score (p=0.123)			
< 14 wks	114	111	0.17 (−0.09, 0.43)
>= 14 wks	518	550	−0.07 (−0.18, 0.05)
MUAC−for−age z−score (p=0.416)			
< 14 wks	114	111	0.07 (−0.20, 0.35)
>= 14 wks	518	550	−0.04 (−0.17, 0.09)

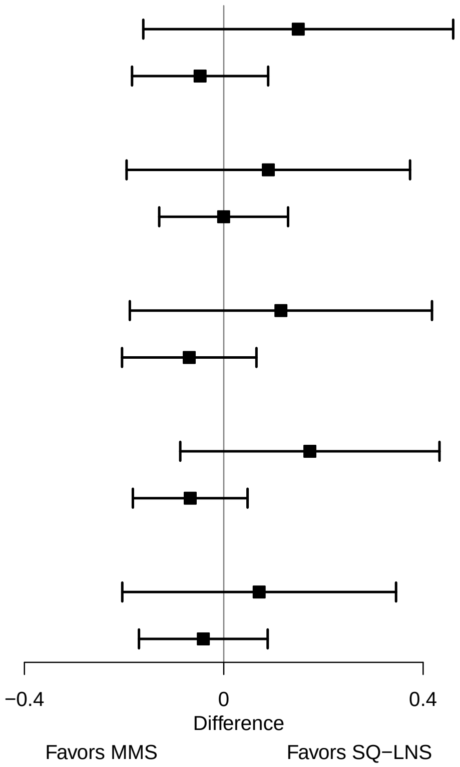

Supplemental figure 6J: Gestational age at supplementation

6J4: Prevalence ratios for 6 mo outcomes

Outcome (p−for−interaction)	SQ−LNS N	MMS N	Prevalence ratio (95% CI)
Underweight (p=0.561)			
< 14 wks	114	111	0.88 (0.35, 2.22)
>= 14 wks	517	550	1.19 (0.80, 1.79)
Stunted (p=0.221)			
< 14 wks	114	111	0.76 (0.40, 1.44)
>= 14 wks	518	546	1.11 (0.86, 1.44)
Wasted (p=0.396)			
< 14 wks	114	111	0.50 (0.09, 2.84)
>= 14 wks	517	546	1.14 (0.52, 2.49)
Low HCAZ (p=0.806)			
< 14 wks	114	111	0.72 (0.24, 2.10)
>= 14 wks	518	550	0.82 (0.53, 1.27)
Low MUAC (p=0.867)			
< 14 wks	114	111	0.87 (0.35, 2.17)
>= 14 wks	518	550	0.93 (0.60, 1.44)
Acute malnutrition (p=0.972)			
< 14 wks	114	111	0.97 (0.40, 2.36)
>= 14 wks	517	546	0.98 (0.65, 1.49)

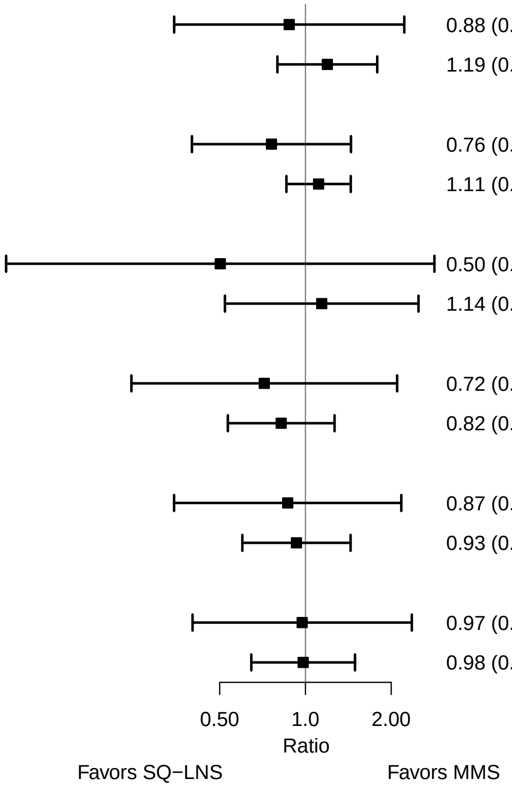

Supplemental figure 6K: Compliance with supplementation

6K1: Mean differences for birth outcomes

Outcome (p−for−interaction)	SQ−LNS N	MMS N	Mean difference (95% CI)
Birth weight (kg) (p=0.677)			
At least 4/7 d	607	621	0.03 (−0.02, 0.08)
Less than 4/7 d	84	75	0.06 (−0.09, 0.21)
Weight−for−age z score (p=0.417)			
At least 4/7 d	607	621	0.05 (−0.06, 0.16)
Less than 4/7 d	84	75	0.19 (−0.16, 0.54)
Weight−for−gestational age z−score (p=0.297)			
At least 4/7 d	602	617	0.04 (−0.07, 0.15)
Less than 4/7 d	80	74	0.23 (−0.08, 0.55)
Birth length (cm) (p=0.800)			
At least 4/7 d	487	513	0.15 (−0.10, 0.40)
Less than 4/7 d	73	63	0.29 (−0.60, 1.18)
Length−for−age z−score (p=0.747)			
At least 4/7 d	487	513	0.07 (−0.06, 0.20)
Less than 4/7 d	73	63	0.14 (−0.30, 0.59)
Length−for−gestational age z−score (p=0.569)			
At least 4/7 d	482	509	0.09 (−0.04, 0.21)
Less than 4/7 d	69	62	−0.01 (−0.39, 0.37)
BMI z−score (p=0.298)			
At least 4/7 d	483	501	−0.01 (−0.14, 0.12)
Less than 4/7 d	73	63	0.21 (−0.25, 0.66)
Head circumference (cm) (p=0.285)			
At least 4/7 d	487	514	0.11 (−0.06, 0.29)
Less than 4/7 d	73	63	0.46 (−0.14, 1.05)
Head circumference−for−age z−score (p=0.219)			
At least 4/7 d	487	514	0.08 (−0.06, 0.21)
Less than 4/7 d	73	63	0.36 (−0.08, 0.80)
Head circumference−for−gestational age z score (p=0.363)			
At least 4/7 d	482	510	0.09 (−0.04, 0.22)
Less than 4/7 d	69	62	0.30 (−0.10, 0.70)
Mid−upper arm circumference (cm) (p=0.888)			
At least 4/7 d	490	515	0.08 (−0.03, 0.19)
Less than 4/7 d	73	63	0.12 (−0.16, 0.40)
Duration (wk) (p=0.358)			
At least 4/7 d	658	674	0.00 (−0.25, 0.26)
Less than 4/7 d	105	95	−0.32 (−0.95, 0.31)

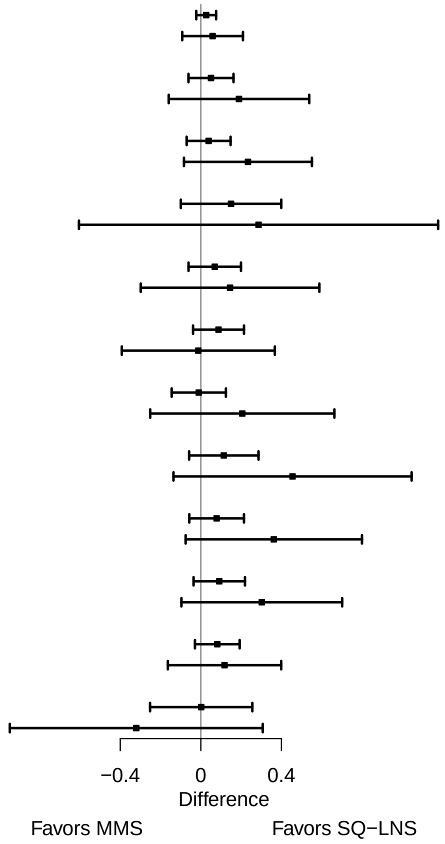

Supplemental figure 6K: Compliance with supplementation

6K2: Relative risks for birth outcomes

Outcome (p−for−interaction)	SQ−LNS N	MMS N	Relative risk (95% CI)
Low birth weight (p=0.460)			
At least 4/7 d	607	621	0.92 (0.67, 1.27)
Less than 4/7 d	84	75	0.68 (0.33, 1.41)
Small−for−gestational age (p=0.213)			
At least 4/7 d	607	621	1.01 (0.81, 1.24)
Less than 4/7 d	84	75	0.67 (0.38, 1.16)
Newborn stunting (p=0.589)			
At least 4/7 d	487	513	1.10 (0.77, 1.58)
Less than 4/7 d	73	63	0.82 (0.38, 1.74)
Low LGAZ (p=0.767)			
At least 4/7 d	487	513	1.25 (0.85, 1.84)
Less than 4/7 d	73	63	0.98 (0.38, 2.51)
Low BMIZ (p=0.148)			
At least 4/7 d	483	501	1.17 (0.72, 1.92)
Less than 4/7 d	73	63	0.44 (0.16, 1.23)
Low HCZ (p=0.113)			
At least 4/7 d	487	514	1.55 (0.89, 2.70)
Less than 4/7 d	73	63	0.48 (0.13, 1.83)
Preterm birth (p=0.861)			
At least 4/7 d	658	674	1.17 (0.82, 1.68)
Less than 4/7 d	105	95	1.34 (0.65, 2.74)

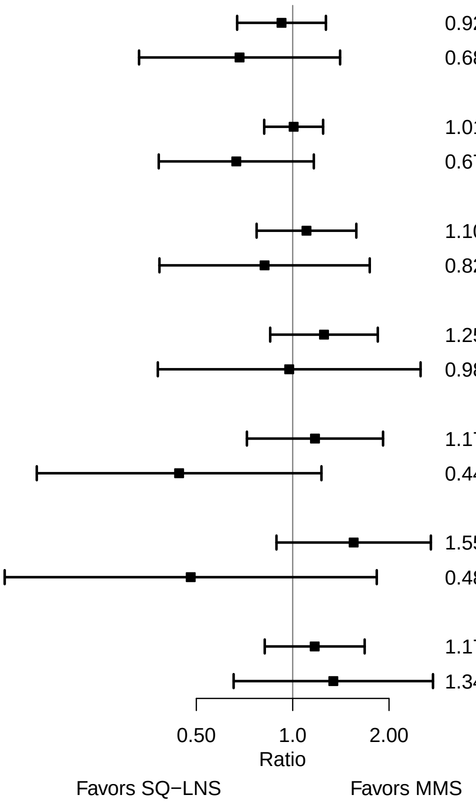

Supplemental figure 6K: Compliance with supplementation

6K3: Mean differences for 6 mo outcomes

Outcome (p−for−interaction)	SQ−LNS N	MMS N	Mean difference (95% CI)
Weight−for−age z score (p=0.454)			
At least 4/7 d	556	596	−0.04 (−0.17, 0.09)
Less than 4/7 d	73	64	0.13 (−0.27, 0.53)
Length−for−age z−score (p=0.737)			
At least 4/7 d	557	592	0.02 (−0.10, 0.14)
Less than 4/7 d	73	64	−0.06 (−0.43, 0.31)
Weight−for−length z−score (p=0.230)			
At least 4/7 d	556	592	−0.07 (−0.20, 0.06)
Less than 4/7 d	73	64	0.16 (−0.21, 0.54)
Head circumference−for−age z−score (p=0.601)			
At least 4/7 d	557	596	−0.04 (−0.15, 0.08)
Less than 4/7 d	73	64	0.05 (−0.25, 0.36)
MUAC−for−age z−score (p=0.985)			
At least 4/7 d	557	596	−0.03 (−0.15, 0.09)
Less than 4/7 d	73	64	0.01 (−0.35, 0.37)

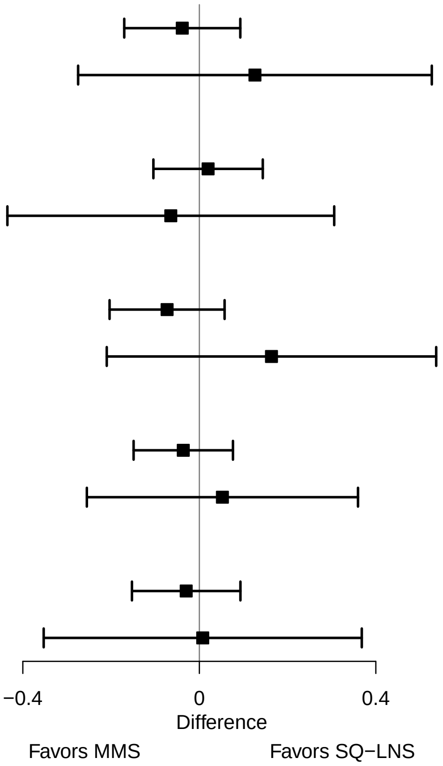

Supplemental figure 6K: Compliance with supplementation

6K4: Prevalence ratios for 6 mo outcomes

Outcome (p−for−interaction)	SQ−LNS N	MMS N	Prevalence ratio (95% CI)
Underweight (p=0.831)			
At least 4/7 d	556	596	1.14 (0.78, 1.66)
Less than 4/7 d	73	64	1.36 (0.23, 8.09)
Stunted (p=0.593)			
At least 4/7 d	557	592	1.07 (0.83, 1.37)
Less than 4/7 d	73	64	0.84 (0.28, 2.49)
Low HCAZ (p=0.454)			
At least 4/7 d	557	596	0.71 (0.46, 1.09)
Less than 4/7 d	73	64	1.29 (0.28, 5.87)
Low MUAC (p=0.600)			
At least 4/7 d	557	596	0.92 (0.61, 1.38)
Less than 4/7 d	73	64	1.34 (0.31, 5.81)
Acute malnutrition (p=0.675)			
At least 4/7 d	556	592	0.98 (0.66, 1.45)
Less than 4/7 d	73	64	1.34 (0.31, 5.81)

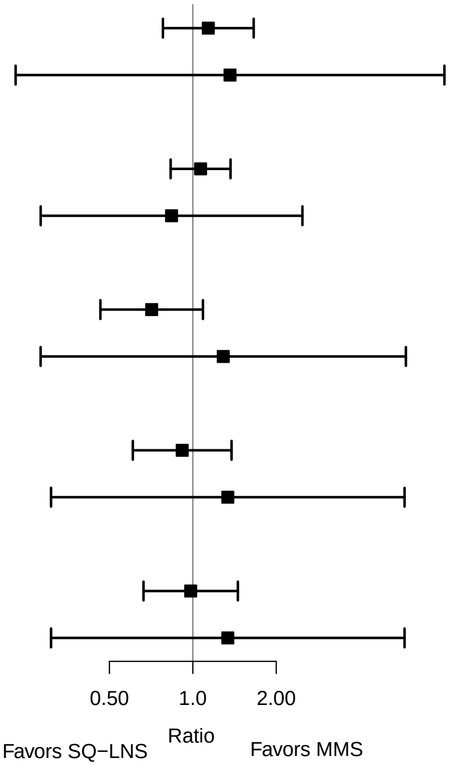

Supplemental figure 6L: Household socio-economic status

6L1: Mean differences for birth outcomes

Outcome (p−for−interaction)	SQ−LNS N	MMS N	Mean difference (95% CI)
Birth weight (kg) (p=0.982)			
At least median	305	343	0.03 (−0.04, 0.10)
Less than median	386	350	0.03 (−0.03, 0.10)
Weight−for−age z score (p=0.875)			
At least median	305	343	0.06 (−0.09, 0.22)
Less than median	386	350	0.07 (−0.07, 0.22)
Weight−for−gestational age z−score (p=0.864)			
At least median	303	339	0.05 (−0.10, 0.20)
Less than median	379	349	0.07 (−0.08, 0.21)
Birth length (cm) (p=0.748)			
At least median	248	288	0.21 (−0.11, 0.53)
Less than median	310	287	0.12 (−0.23, 0.48)
Length−for−age z−score (p=0.806)			
At least median	248	288	0.10 (−0.07, 0.26)
Less than median	310	287	0.06 (−0.12, 0.25)
Length−for−gestational age z−score (p=0.556)			
At least median	246	284	0.12 (−0.05, 0.28)
Less than median	303	286	0.05 (−0.13, 0.22)
BMI z−score (p=0.927)			
At least median	246	284	0.03 (−0.15, 0.21)
Less than median	308	280	0.04 (−0.14, 0.22)
Head circumference (cm) (p=0.728)			
At least median	246	288	0.20 (−0.02, 0.42)
Less than median	312	288	0.10 (−0.14, 0.34)
Head circumference−for−age z−score (p=0.688)			
At least median	246	288	0.15 (−0.03, 0.33)
Less than median	312	288	0.07 (−0.11, 0.26)
Head circumference−for−gestational age z score (p=0.582)			
At least median	244	284	0.16 (−0.01, 0.33)
Less than median	305	287	0.07 (−0.10, 0.24)
Mid−upper arm circumference (cm) (p=0.934)			
At least median	248	288	0.11 (−0.04, 0.26)
Less than median	313	289	0.09 (−0.05, 0.23)
Duration (wk) (p=0.251)			
At least median	335	379	0.11 (−0.19, 0.41)
Less than median	427	383	−0.17 (−0.52, 0.19)

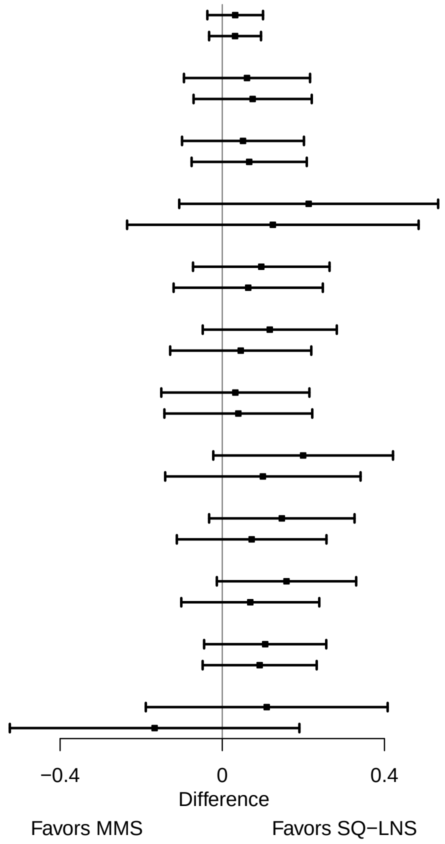

Supplemental figure 6L: Household socio-economic status

6L2: Relative risks for birth outcomes

Outcome (p−for−interaction)	SQ−LNS N	MMS N	Relative risk (95% CI)
Low birth weight (p=0.822)			
At least median	305	343	0.85 (0.54, 1.34)
Less than median	386	350	0.91 (0.61, 1.34)
Birth weight < 2 kg (p=0.333)			
At least median	305	343	1.71 (0.56, 5.18)
Less than median	386	350	0.72 (0.27, 1.93)
Small−for−gestational age (p=0.884)			
At least median	305	343	0.97 (0.71, 1.32)
Less than median	386	350	0.94 (0.73, 1.22)
Large−for−gestational age (p=0.804)			
At least median	305	343	1.24 (0.52, 2.96)
Less than median	386	350	1.08 (0.47, 2.48)
Newborn stunting (p=0.250)			
At least median	248	288	0.82 (0.48, 1.40)
Less than median	310	287	1.19 (0.79, 1.80)
Low LGAZ (p=0.928)			
At least median	248	288	1.18 (0.65, 2.15)
Less than median	310	287	1.20 (0.77, 1.86)
Low BMIZ (p=0.127)			
At least median	246	284	1.39 (0.69, 2.82)
Less than median	308	280	0.68 (0.38, 1.23)
Low HCZ (p=0.675)			
At least median	246	288	1.05 (0.43, 2.55)
Less than median	312	288	1.30 (0.70, 2.41)
Preterm birth (p=0.257)			
At least median	335	379	0.97 (0.59, 1.59)
Less than median	427	383	1.47 (0.94, 2.30)

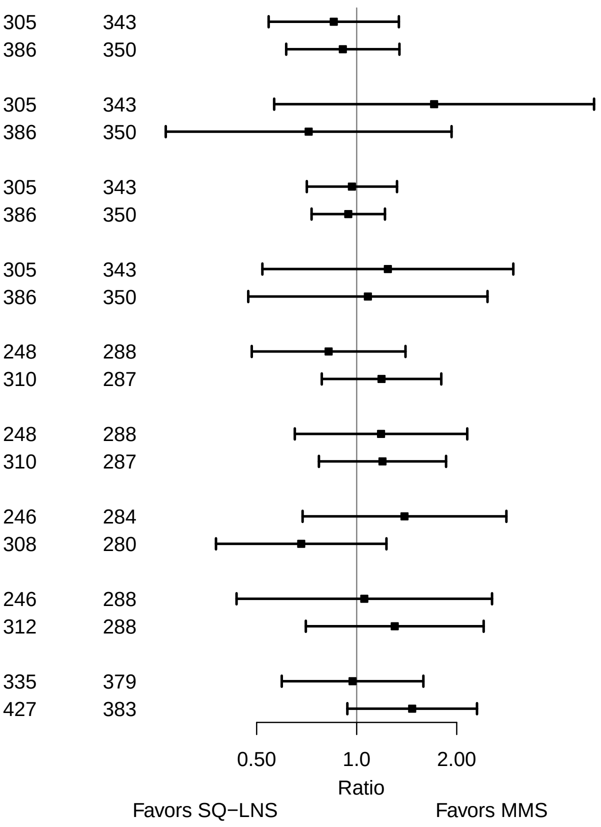

Supplemental figure 6L: Household socio-economic status

6L3: Mean differences for 6 mo outcomes

Outcome (p−for−interaction)	SQ−LNS N	MMS N	Mean difference (95% CI)
Weight−for−age z score (p=0.717)			
At least median	267	325	−0.03 (−0.22, 0.15)
Less than median	361	332	0.01 (−0.16, 0.19)
Length−for−age z−score (p=0.463)			
At least median	268	322	−0.03 (−0.20, 0.14)
Less than median	361	331	0.06 (−0.11, 0.22)
Weight−for−length z−score (p=0.949)			
At least median	267	322	−0.04 (−0.22, 0.14)
Less than median	361	331	−0.03 (−0.20, 0.14)
Head circumference−for−age z−score (p=0.914)			
At least median	268	325	−0.02 (−0.17, 0.14)
Less than median	361	332	−0.04 (−0.18, 0.11)
MUAC−for−age z−score (p=0.857)			
At least median	268	325	−0.03 (−0.19, 0.14)
Less than median	361	332	−0.01 (−0.17, 0.16)

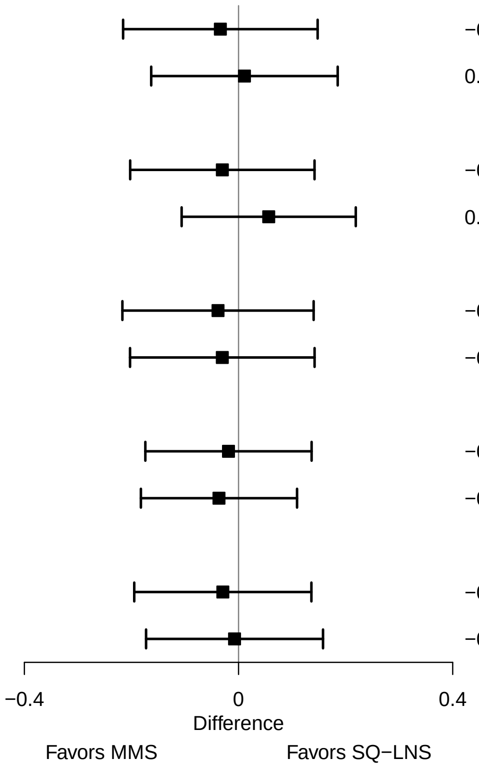

Supplemental figure 6L: Household socio-economic status

6L4: Prevalence ratios for 6 mo outcomes

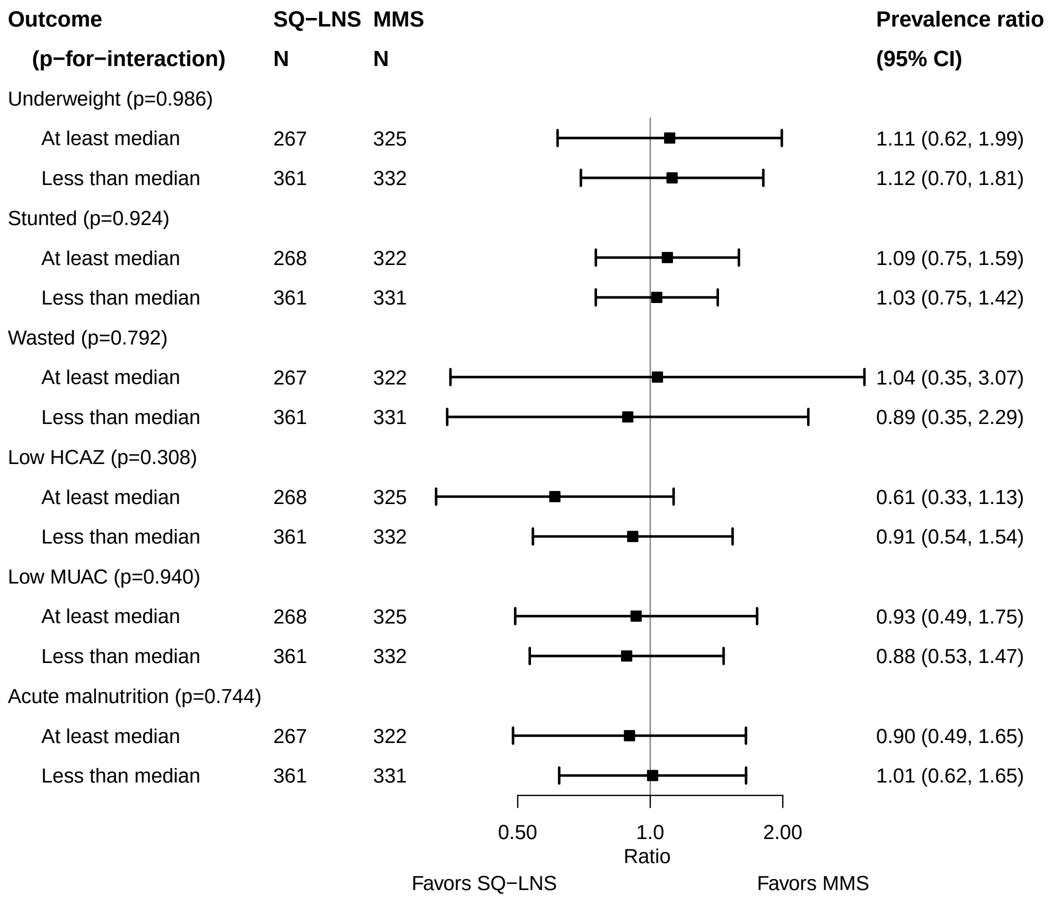

Supplemental figure 6M: Household food security

6M1: Mean differences for birth outcomes

Outcome (p−for−interaction)	SQ−LNS N	MMS N	Mean difference (95% CI)
Birth weight (kg) (p=0.715)			
Mild to secure	354	334	0.02 (−0.05, 0.08)
Moderate to severe	336	354	0.04 (−0.03, 0.10)
Weight−for−age z score (p=0.731)			
Mild to secure	354	334	0.04 (−0.11, 0.19)
Moderate to severe	336	354	0.08 (−0.07, 0.23)
Weight−for−gestational age z−score (p=0.727)			
Mild to secure	347	330	0.04 (−0.10, 0.19)
Moderate to severe	334	353	0.06 (−0.09, 0.21)
Birth length (cm) (p=0.559)			
Mild to secure	307	298	0.11 (−0.21, 0.44)
Moderate to severe	249	272	0.23 (−0.13, 0.60)
Length−for−age z−score (p=0.608)			
Mild to secure	307	298	0.05 (−0.12, 0.22)
Moderate to severe	249	272	0.12 (−0.07, 0.31)
Length−for−gestational age z−score (p=0.597)			
Mild to secure	300	294	0.05 (−0.10, 0.21)
Moderate to severe	247	271	0.12 (−0.07, 0.31)
BMI z−score (p=0.810)			
Mild to secure	307	298	0.00 (−0.18, 0.17)
Moderate to severe	245	261	0.03 (−0.17, 0.22)
Head circumference (cm) (p=0.373)			
Mild to secure	308	299	0.25 (0.00, 0.49)
Moderate to severe	248	272	0.03 (−0.19, 0.26)
Head circumference−for−age z−score (p=0.400)			
Mild to secure	308	299	0.18 (−0.01, 0.37)
Moderate to severe	248	272	0.03 (−0.14, 0.21)
Head circumference−for−gestational age z score (p=0.293)			
Mild to secure	301	295	0.19 (0.02, 0.37)
Moderate to severe	246	271	0.03 (−0.14, 0.20)
Mid−upper arm circumference (cm) (p=0.274)			
Mild to secure	309	299	0.03 (−0.11, 0.17)
Moderate to severe	250	273	0.13 (−0.02, 0.28)
Duration (wk) (p=0.383)			
Mild to secure	390	365	0.03 (−0.28, 0.33)
Moderate to severe	371	393	−0.16 (−0.53, 0.21)

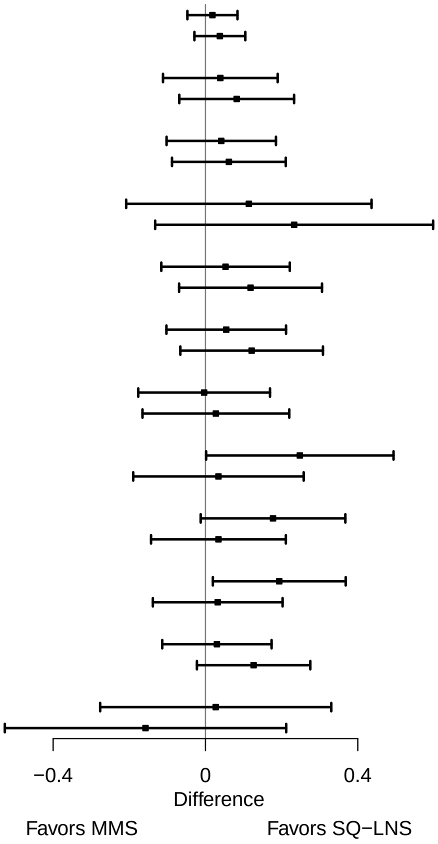

Supplemental figure 6M: Household food security

6M2: Relative risks for birth outcomes

Outcome (p−for−interaction)	SQ−LNS N	MMS N	Relative risk (95% CI)
Low birth weight (p=0.539)			
Mild to secure	354	334	0.98 (0.64, 1.49)
Moderate to severe	336	354	0.81 (0.54, 1.22)
Birth weight < 2 kg (p=0.194)			
Mild to secure	354	334	1.62 (0.57, 4.58)
Moderate to severe	336	354	0.60 (0.18, 2.06)
Small−for−gestational age (p=0.515)			
Mild to secure	354	334	1.02 (0.77, 1.36)
Moderate to severe	336	354	0.92 (0.70, 1.22)
Large−for−gestational age (p=0.445)			
Mild to secure	354	334	0.87 (0.36, 2.11)
Moderate to severe	336	354	1.49 (0.67, 3.30)
Newborn stunting (p=0.446)			
Mild to secure	307	298	0.93 (0.57, 1.51)
Moderate to severe	249	272	1.22 (0.79, 1.90)
Low LGAZ (p=0.287)			
Mild to secure	307	298	1.50 (0.84, 2.68)
Moderate to severe	249	272	1.07 (0.68, 1.69)
Low BMIZ (p=0.312)			
Mild to secure	307	298	1.17 (0.66, 2.07)
Moderate to severe	245	261	0.85 (0.41, 1.78)
Low HCZ (p=0.627)			
Mild to secure	308	299	1.16 (0.57, 2.34)
Moderate to severe	248	272	1.50 (0.69, 3.25)
Low HCGAZ (p=0.692)			
Mild to secure	308	299	0.92 (0.28, 3.06)
Moderate to severe	248	272	0.66 (0.16, 2.74)
Preterm birth (p=0.982)			
Mild to secure	390	365	1.27 (0.78, 2.06)
Moderate to severe	371	393	1.15 (0.74, 1.79)

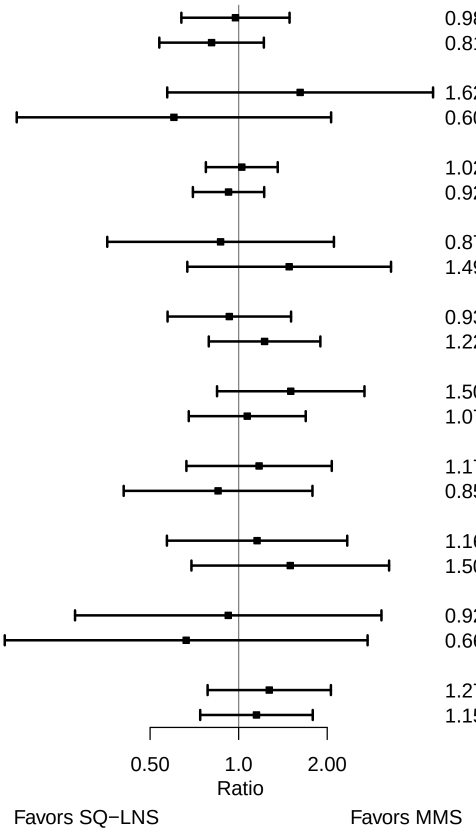

Supplemental figure 6M: Household food security

6M3: Mean differences for 6 mo outcomes

Outcome (p−for−interaction)	SQ−LNS N	MMS N	Mean difference (95% CI)
Weight−for−age z score (p=0.628)			
Mild to secure	319	314	0.02 (−0.15, 0.20)
Moderate to severe	309	339	−0.05 (−0.23, 0.13)
Length−for−age z−score (p=0.803)			
Mild to secure	319	312	0.05 (−0.11, 0.21)
Moderate to severe	310	338	0.00 (−0.17, 0.17)
Weight−for−length z−score (p=0.566)			
Mild to secure	319	312	−0.01 (−0.18, 0.17)
Moderate to severe	309	338	−0.08 (−0.25, 0.10)
Head circumference−for−age z−score (p=0.243)			
Mild to secure	319	314	0.05 (−0.10, 0.20)
Moderate to severe	310	339	−0.10 (−0.25, 0.05)
MUAC−for−age z−score (p=0.551)			
Mild to secure	319	314	0.00 (−0.16, 0.16)
Moderate to severe	310	339	−0.04 (−0.22, 0.13)

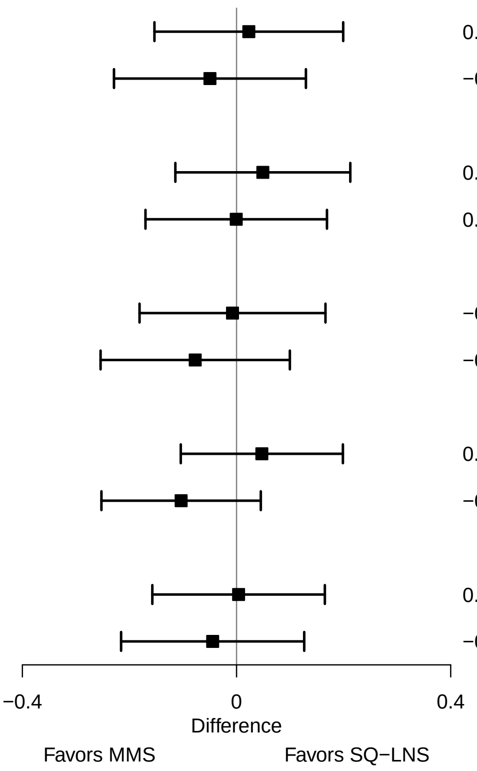

Supplemental figure 6M: Household food security

6M4: Prevalence ratios for 6 mo outcomes

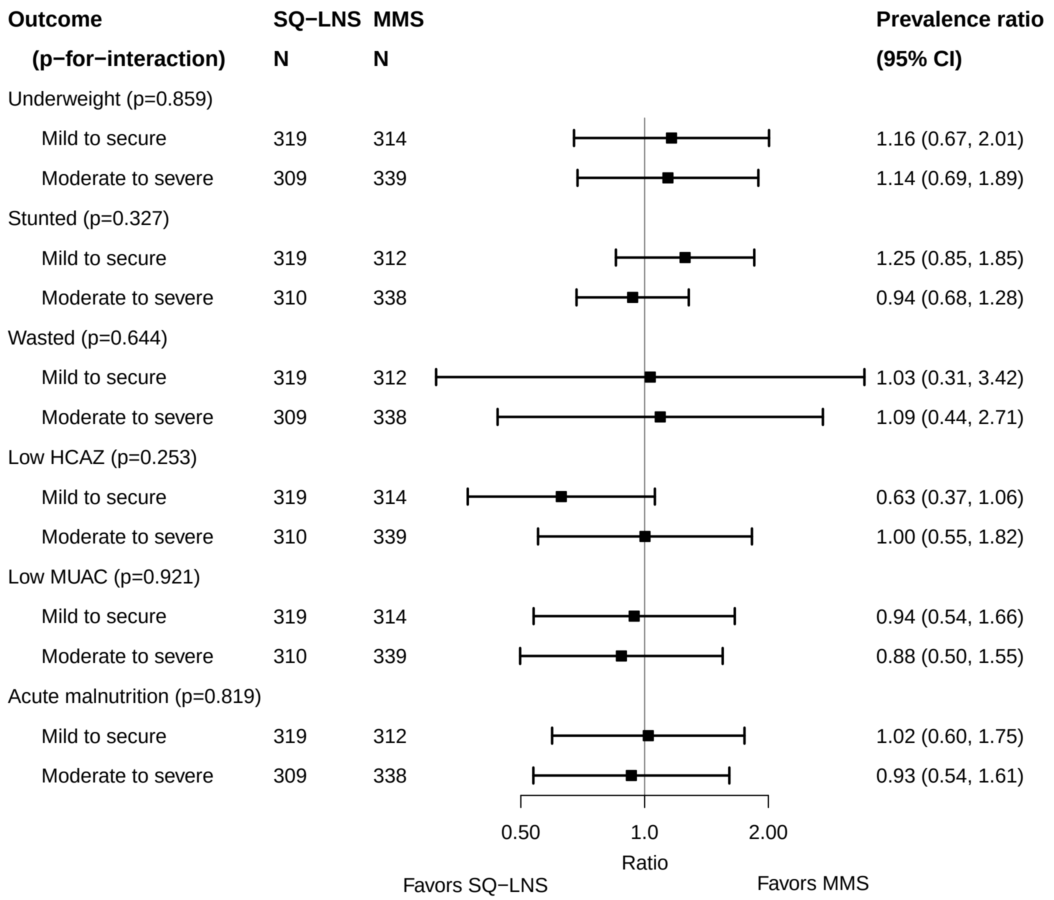

Supplement: Multimedia component 1 [file mmc1.zip › Maternal SQ-LNS Supplemental_2024-09-03/11_Maternal SQ-LNS Supplemental figure 6.pdf]
