# Supplementary material for: Effects of prenatal small-quantity lipid-based nutrient supplements on pregnancy, birth, and infant outcomes: a systematic review and meta-analysis of individual participant data from randomized controlled trials in low- and middle-income countries
Source: Am J Clin Nutr. 2024 Aug 16;120(4):814–35. doi: 10.1016/j.ajcnut.2024.08.008 (PMC11473441; doi:10.1016/j.ajcnut.2024.08.008)
Supplement: Multimedia component 1 [file mmc1.zip › Maternal SQ-LNS Supplemental_2024-09-03/3_Maternal SQ-LNS Supplemental table 6.docx]

**Supplemental Table 6:** **Risk of bias assessment in each trial**

| **Country** | **Author** | **Random sequence generation** | **Allocation concealment** | **Blinding participants** | **Outcome assessment^1^** | **Incomplete outcome** | **Selective reporting** | **Other** |
| --- | --- | --- | --- | --- | --- | --- | --- | --- |
| Bangladesh | Mridha 2016 (31) | low | low | high | low | low | low | low |
| Ghana | Adu Afarwuah 2015 (35) | low | low | high | low | low | low | low |
| Malawi | Ashorn 2015 (36) | low | low | high | low | low | low | low |
| Guatemala | Hambidge 2019 (37) | low | low | high | unclear | low | low | low |

^1^Due to the nature of the intervention, blinding of participants was not possible. We considered birth outcome assessment to be at low risk of bias only when it was clearly specified that data collectors who performed the anthropometric measurements were not aware of group allocation, and it would be unlikely that they could easily become aware of group allocation (i.e. observation of interventions materials in study communities, non-intervention passive control arms, etc.)

| **Adu-Afarwuah 2016 (35)** |  |  |
| --- | --- | --- |
| **Bias** | **Authors’ judgement** | **Support for judgement** |
| Random sequence generation (selection bias) | Low risk | **Quote**: “women were randomly allocated into one of 3 groups by using a computer- generated scheme (SAS version 9.3; SAS Institute) in blocks of 9…the study statistician at UC Davis, who designed the randomization scheme”  **Comment:** adequately done |
| Allocation concealment (selection bias) | Low risk | **Quote**: “Sheets bearing supplement allocations represented by 6 different color codes (3 for IFA and 3 for MMN) and an inscription “LNS” (for the LNS group) and numbered 1–1320 were placed in opaque envelopes and stacked in increasing order. At each enrollment, the study nurse shuffled the 9 topmost envelopes in the stack, and the woman picked one to reveal allocation.”  **Comment:** adequately done |
| Blinding of participants and personnel (performance bias) | High risk | **Quote:** “Two individuals in Ghana who were independent of the research team color-coded the capsules by placing color stickers (which also included the letter P or L to indicate pregnancy or lactation) on the blister packs of IFA and MMN, so that no investigator, study worker, or participant knew the identities of the capsules except by the colors. Each fieldworker received from the field supervisor the coded allocations of only those women assigned to that fieldworker for follow-up home visits and was  told not to reveal those codes to anyone. It was not possible to blind the fieldworkers and study participants to those consuming capsules vs. LNS (because of the starkly different characteristics)”  **Comment:** not done |
| Blinding of outcome assessment (detection bias) | Low risk | **Quote**: “…but none of the maternal or newborn anthropometrists was aware of the code allocations. Likewise, data analysts remained blinded until all preliminary analyses had been completed, and the allocation codes were broken.”  **Comment:** adequately done |
| Incomplete outcome data (attrition bias) | Low risk | **Attrition:** Pregnancy outcomes: IFA group = 349/441; MMN group = 354/439; LNS group = 354/440; Birth anthropometric outcomes: IFA group = 305/441; MMN group = 318/439; LNS: 307/440  **Comment:** Reason for exclusion of participants provided, no attrition for pregnancy outcomes; reasons for attrition provided (additional details in Adu-Afarwuah *et al.* AJCN 2016) |
| Selective reporting (reporting bias) | Low risk | **Comment**: The trial was registered at clinicaltrials.gov (NCT00970866); SAP available online; outcomes described in the methods section reported in the results section |
| Other bias | Low risk | **Quote: “…**discovered that some mislabeling of IFA and MMN supplements had occurred due to miscommunication, and as a result, some women in the IFA and MMN groups had  received both IFA and MMN supplements during pregnancy. Throughout the study, we kept full records of when any batch of supplement arrived in Ghana, the date the distribution of each batch was started, and the batch number of each blister pack delivered to each woman at every home visit. Therefore, it was possible later to identify which subjects received both the IFA and MMN supplements during pregnancy…When we discovered the mislabeling (described above), 510 women had been enrolled. At the recommendation of the Data and Safety Monitoring Board and the funder, we calculated  the sample size by using the same parameters as before, but assuming 20% attrition based on data up to that point, and determined that if we enrolled an additional target number of 810 women (270/group), there would be sufficient statistical power to examine pregnancy outcomes even if we excluded women in the IFA and MMN groups who had the mixed “exposure” and those in the LNS groups who were pregnant during the same period”.  **Comment**: adequately done |
| **Funding**  Funded by a grant to the University of California, Davis, from the Bill & Melinda Gates Foundation. | | |

| **Ashorn 2015 (36)** |  |  |
| --- | --- | --- |
| **Bias** | **Authors’ judgement** | **Support for judgement** |
| Random sequence generation (selection bias) | Low risk | **Quote:** “A study statistician not involved in data collection generated 4 randomization ode lists in blocks of 9 (one list for each of the 4 enrollment sites). In the randomization process, each participant number was allocated one of 9 possible letter codes (A, B, C, D,  E, H, J, K, or M). Each letter code corresponded to one of the 3 interventions (i.e., each intervention matched with 3 separate letter codes). Another researcher not involved with the iLiNSDYAD trial then created individual randomization slips, each containing one unique identification number and the corresponding letter code.  **Comment**: adequately done. |
| Allocation concealment (selection bias) | Low risk | **Quote**: “The researcher sealed the slips into individual opaque randomization envelopes, marked each envelope with the trial name and an individual participant number, and sorted the envelopes in 4 stacks (one for each site), each ordered by the participant number shown on the envelope…For the actual enrollment and group allocation, a randomizer picked and shuffled the randomization envelopes for the 6 lowest participant numbers that had not yet been assigned to any participant. He or she then asked the potential participant to choose one, without showing her the envelope identifiers. The number on the envelope chosen by the woman became her participant number, and the contents of the envelope indicated her group allocation (in letter codes)”.  **Comment:** adequately done |
| Blinding of participants and personnel (performance bias) | High risk | **Quote:** “The IFA and MMN interventions were provided by using double-masked procedures—that is, the capsules looked identical, and neither the participants nor the research team members were aware of the nutrient contents of the supplement capsules.  For the LNS group, we used single-masked procedures—that is, field workers who delivered the supplements knew which mothers were receiving LNS (but not a difference between IFA and MMN), and the participants were advised not to disclose information about their supplements to anyone other than an iLiNS team member.”  **Comment:** not done |
| Blinding of outcome assessment (detection bias) | Low risk | **Quote:** “The data collectors who performed the anthropometric measurements or assessed other outcomes were not aware of group allocation. Researchers responsible for the data cleaning remained blind to the trial code until the database was fully cleaned.  **Comment**: adequately done |
| Incomplete outcome data (attrition bias) | Low risk | **Attrition:**  Pregnancy outcomes: IFA group = 437/463; MMN group = 434/466; LNS group = 436/462; Birth weight: IFA group = 388/437; MMN group = 381/434; LNS: 394/436  **Quote:** “An analysis that included the twins and used the number of fetuses as a covariate also gave essentially similar results, and so did the Heckman’s selection models that adjusted for the potential correlation between a tendency of missing data on outcome  values and their actual values (details not shown)”; ” Internal validity could have been compromised by a relatively large number of missing data, delay in anthropometric measurements of some participants, temporary discontinuation of the LNS distribution during the trial, and our inability to directly observe the consumption of the study supplements. Because the results were robust to several sensitivity analyses, we believe these factors did not significantly bias our conclusions. However, the smaller sample size than originally intended (due to budget reduction) limited the statistical power of the study. Therefore, although the results do not support the study hypothesis, they also  do not rule out a modest intervention effect on birth size.”  **Comment:** adequately done |
| Selective reporting (reporting bias) | Low risk | **Comment**: The trial was registered at clinicaltrials.gov (NCT01239693); SAP available online; outcomes described in the methods section reported in the results section |
| Other bias | Low risk | **Quote:** “During the trial implementation, international organizations involved in medium-quantity LNS distribution to children with acute malnutrition made a recommendation on a new quality assurance procedure for all such products. The recommendation involved the testing of LNS for the presence of *Cronobacter sakazakii* bacteria and in clinical practice withholding of the use of untested products or those that were found to contain any *C. sakazakii.* After consultation with members of the trial’s data  safety and monitoring board, the study team decided to withhold further distribution of LNS to the iLiNS-DYAD trial participants until the recommended testing had been completed. Because of this episode, a total of 160 pregnant women in the LNS group  missed their study supplement for a period ranging from 1 to 20 d between 1 August 2012 and 21 August 2012. Of these women, 127 were provided with IFA capsules (1 capsule/d) while LNS was on hold; the other 33 were not located at their homes during  the IFA distribution.”  **Comment:** adequately done, disrupted exposure time < 3 weeks, any potential bias to the null hypothesis |
| **Funding**  Supported in part by a grant to the University of California, Davis, from the Bill & Melinda Gates Foundation, with additional funding from the Office of Health, Infectious Diseases, and Nutrition, Bureau for Global Health, US Agency for International Development (USAID) under terms of cooperative agreement AID-OAAA-1200005, through the Food and Nutrition Technical Assistance III Project (FANTA), managed by FHI 360. For data management and statistical analysis, the team received additional support from the Academy of Finland grant 252075 and the Medical Research Fund of Tampere University Hospital grant 9M004. YBC was supported by the Singapore Ministry of Health’s National Medical Council under its Clinician Scientist Award. | | |

| **Mridha 2016 (31)** |  |  |
| --- | --- | --- |
| **Bias** | **Authors’ judgement** | **Support for judgement** |
| Random sequence generation (selection bias) | Low risk | **Quote:** “For the randomization, the study statistician at UC Davis first stratified the 64 clusters by subdistrict and union, and then assigned each cluster to 1 of 4 sets containing 16 clusters each. This procedure was then replicated several thousand times, and each randomization was tested for balance across groups with respect to mean cluster population, number of clinics and health workers per 1000 people, number of health-/nutrition-related nongovernmental organizations in the cluster, and the source of funding for the CHDP, as well as the SD of the cluster population size. The final randomization to the 4 arms was then chosen at random from the acceptable potential randomizations; and the letters A, B, C, and D were assigned to the 4 sets, randomly permuting them by sorting on a randomly generated, uniformly distributed number (with the use of SAS for Windows, release 9.2; SAS Institute) and assigning them respectively to control, child-only MNP, child-only LNS, and comprehensive LNS treatments”.  **Comment:** adequately done |
| Allocation concealment (selection bias) | Low risk | **Quote:** “ The final randomization to the 4 arms was then chosen at random from the acceptable potential randomizations; and the letters A, B, C, and D were assigned to the 4 sets, randomly permuting them by sorting on a randomly generated, uniformly distributed number (with the use of SAS for Windows, release 9.2; SAS Institute) and assigning them respectively to control, child-only MNP, child-only LNS, and comprehensive LNS treatments”.  **Comment**: central randomization of a cluster-randomized trial |
| Blinding of participants and personnel (performance bias) | High risk | **Comment**: participant blinding not possible due to the nature of the intervention (LNS vs. IFA) |
| Blinding of outcome assessment (detection bias) | Low risk | **Quote:** “The trial was a researcher-blind, longitudinal, cluster randomized effectiveness trial”; “ Data collection was performed by 2 separate teams: the “SDU visit team,” which collected clinical and anthropometric data at the SDU, and the “home visit team,” which enrolled mothers and collected baseline and follow-up data at participants’ homes”… “To the extent possible, both study evaluation teams were kept blind to group assignment, although this was difficult for home visit team members because they might have seen supplements in the home”; “researchers responsible for the collection of outcome data were kept blind to study assignment.”  **Comment:** adequately done |
| Incomplete outcome data (attrition bias) | Low risk | **Attrition**: LNS = 898/1047; IFA = 2551/2964  **Comment:** reasons given for loss to follow-up; missing outcome data balanced in numbers across intervention groups |
| Selective reporting (reporting bias) | Low risk | **Comment:** The trial was registered at ClinicalTrials.gov (NCT01715038); outcomes described in the methods section reported in the results section |
| Other bias | Low risk | **Quote:** “LNS-PL distribution was interrupted from 8 August to 20 October 2012 to comply with a new quality-control criterion for ready-to-use supplementary foods implemented by the World Food Program, which required the absence of Cronobacter  sakazakii (i.e., no samples testing positive at any amount) …During the interruption, women in all of the arms received IFA.”; ” A separate exploratory analysis was conducted to examine the effect of the intervention on children who were born before the interruption of LNS-PLs. To account for the interruption in LNS-PLs, we tested models that included the number of days the woman was participating in the study during the interruption period. However, we found that this did not improve the model fit compared with including the time interval variable and its interaction with intervention group in the model, and therefore we did not continue with this approach.”  **Comment:** adequately done |
| **Funding**  Supported by the Office of Health, Infectious Diseases, and Nutrition, Bureau for Global Health, US Agency for International Development (USAID) under the terms of cooperative agreement AID-OAA-A-12-00005, through the Food and Nutrition Technical Assistance III Project (FANTA), managed by FHI 360. Our research intervention was incorporated into the community heath and development program of LAMB, which was supported by Plan-Bangladesh in 6 of the 11 study unions. Nutriset S.A.S. prepared the lipid-based nutrient supplements for this trial, and Hudson Pharmaceuticals Ltd. prepared the iron and folic acid tablets. | | |

| **Hambidge 2019 (37)** |  |  |
| --- | --- | --- |
| **Bias** | **Authors’ judgement** | **Support for judgement** |
| Random sequence generation (selection bias) | Low risk | **Quote: “**The DCC *(Data Coordinating Center at RTI International)* created the randomization scheme, centrally generating the allocation sequence for each site. To ensure geographic balance, a permuted block design stratified by GN clusters was used for assigning individual participants to a trial arm. The allocation ratio was 1:1:1 within blocks which randomly varied between sizes of 3, 6, or 9 for each site. Once the responsible home visitor research assistant identified an eligible participant, they received the random assignment generated by the site data manager from the centralized computerized data management system maintained by the DCC.”  **Comment:** adequately done |
| Allocation concealment (selection bias) | Low risk | **Quote:** Once the responsible home visitor research assistant identified an eligible participant, they received the random assignment generated by the site data manager from the centralized computerized data management system maintained by the DCC.”  **Comment**: adequately done |
| Blinding of participants and personnel (performance bias) | High risk | **Quote:** “This was an individually randomized, nonmasked, multisite controlled efficacy trial”  **Comment**: participant blinding not possible due to the nature of the intervention (LNS pre-conception vs. LNS at the end of the 1^st^ trimester vs. standard of care) |
| Blinding of outcome assessment (detection bias) | Unclear risk | **Quote:** All anthropometry was performed by trained assessment teams who were not involved in the biweekly home visits.  **Comment:** unclear if the anthropometrists were blinded to intervention assignment |
| Incomplete outcome data (attrition bias) | Low risk | **Attrition**: data were not provided for Guatemala individually, but for all sites in this multi-site trial combined.  **Comment:** reasons given for loss to follow-up; missing outcome data balanced in numbers across intervention groups |
| Selective reporting (reporting bias) | Low risk | **Comment:** The trial was registered at ClinicalTrials.gov (NCT01883193); study protocol is available online; outcomes described in the methods section reported in the results section |
| Other bias | Low risk | **Comment:** no other potential sources of bias reported |
| **Funding**  Supported by Bill &Melinda Gates Foundation grant OPP1055867 and *Eunice Kennedy Shriver* National Institute of Child Health and Human Development and Office of Dietary Supplements, NIH grant U10 HD  076474. | | |
