# Supplementary material for: Effects of prenatal small-quantity lipid-based nutrient supplements on pregnancy, birth, and infant outcomes: a systematic review and meta-analysis of individual participant data from randomized controlled trials in low- and middle-income countries
Source: Am J Clin Nutr. 2024 Aug 16;120(4):814–35. doi: 10.1016/j.ajcnut.2024.08.008 (PMC11473441; doi:10.1016/j.ajcnut.2024.08.008)
Supplement: Multimedia component 1 [file mmc1.zip › Maternal SQ-LNS Supplemental_2024-09-03/4_Maternal SQ-LNS Supplemental table 7.docx]

**Supplemental Table 7A: Sensitivity analyses for main effects of SQ-LNS vs IFA/SOC on birth outcomes^1^**

| **Birth outcomes** | **MD or RR (95% CI)^2^**  **N (trials); p-value**  **Excluding gestational age not measured by ultrasound** | **MD or RR (95% CI)^2^**  **N (trials); p-value**  **Anthropometry within 72 h of birth** | **MD or RR (95% CI)^2^**  **N (trials); p-value**  **Excluding Guatemala** | **MD or RR (95% CI)^2^**  **N (trials); p-value**  **Including pre-conception** |
| --- | --- | --- | --- | --- |
| Birth weight (g) | N/A | 49.3 (26.2, 72.3)  4468 (4); p<0.001 | 47.6 (22.1, 73.0)  4834 (3); p<0.001 | 42.8 (20.2, 65.5)  5461 (4); p<0.001 |
| Weight-for-age z score (WAZ) | N/A | 0.12 (0.07, 0.18)  4468 (4); p<0.001 | 0.12 (0.05, 0.18)  4834 (3); p<0.001 | 0.10 (0.05, 0.16)  5461 (4); p<0.001 |
| Weight-for-gestational age z-score (WGAZ) | 0.13 (0.05, 0.21)  1717 (3); p=0.001 | 0.15 (0.07, 0.24)  1129 (3); p=0.001 | 0.09 (-0.02, 0.19)  1370 (2); p=0.099 | 0.11 (0.04, 0.19)  1871 (3); p=0.003 |
| Low birth weight (LBW) | N/A | 0.91 (0.82, 1.01)  4468 (4); p=0.075 | 0.90 (0.80, 1.00)  4834 (3); p=0.054 | 0.90 (0.82, 1.00)  5461 (4); p=0.048 |
| Birth weight < 2 kg | N/A | 0.75 (0.57, 1.00)  4035 (3); p=0.050 | 0.78 (0.60, 1.02)  4834 (3); p=0.069 | 0.81 (0.63, 1.05)  5461 (4); p=0.108 |
| Small-for-gestational age (SGA) | 0.86 (0.73, 1.01)  1717 (3); p=0.070 | 0.97 (0.92, 1.02)  4378 (4); p=0.243 | 0.97 (0.92, 1.02)  4834 (3); p=0.199 | 0.96 (0.92, 1.01)  5335 (4); p=0.133 |
| Large-for-gestational age (LGA) | 0.95 (0.53, 1.72)  1370 (2); p=0.878 | 1.64 (0.81, 3.32)  3786 (2); p=0.168 | 1.00 (0.61, 1.65)  4834 (3); p=0.984 | 1.00 (0.61, 1.65)  4834 (3); p=0.984 |
| Birth length (cm) | N/A | 0.19 (0.08, 0.31)  4217 (3); p=0.001 | 0.19 (0.07, 0.31)  4575 (3); p=0.002 | 0.15 (0.04, 0.25)  5202 (4); p=0.005 |
| Length-for-age z score (LAZ) | N/A | 0.11 (0.05, 0.17)  4217 (3); p<0.001 | 0.11 (0.04, 0.17)  4575 (3); p=0.001 | 0.09 (0.03, 0.14)  5202 (4); p=0.002 |
| Length-for-gestational age z-score (LGAZ) | 0.13 (0.05, 0.21)  1460 (3); p=0.002 | 0.14 (0.05, 0.23)  880 (2); p=0.002 | 0.10 (-0.03, 0.22)  1113 (2); p=0.123 | 0.09 (0.03, 0.15)  1614 (3); p=0.004 |
| Newborn stunting | N/A | 0.83 (0.75, 0.93)  4217 (3); p=0.001 | 0.84 (0.74, 0.95)  4575 (3); p=0.005 | 0.87 (0.78, 0.97)  5202 (4); p=0.014 |
| Low LGAZ | 0.95 (0.71, 1.26)  1460 (3); p=0.713 | 0.90 (0.79, 1.03)  4127 (3); p=0.128 | 0.90 (0.80, 1.02)  4575 (3); p=0.099 | 0.91 (0.81, 1.03)  5076 (4); p=0.123 |
| BMI-for-age z-score (BMIZ) | N/A | 0.11 (0.04, 0.17)  4217 (3); p=0.001 | 0.10 (0.03, 0.17)  4563 (3); p=0.004 | 0.09 (0.03, 0.16)  5190 (4); p=0.004 |
| Low BMIZ | N/A | 0.89 (0.81, 0.98)  4217 (3); p=0.016 | 0.90 (0.81, 0.99)  4563 (3); p=0.029 | 0.90 (0.82, 0.99)  5190 (4); p=0.035 |
| Head circumference (cm) | N/A | 0.10 (0.03, 0.18)  4218 (3); p=0.005 | 0.12 (0.04, 0.20)  4577 (3); p=0.003 | 0.10 (0.03, 0.17)  5204 (4); p=0.006 |
| Head circumference-for-age z score (HCZ) | N/A | 0.10 (0.04, 0.15)  4218 (3); p=0.001 | 0.10 (0.04, 0.17)  4577 (3); p=0.002 | 0.09 (0.03, 0.14)  5204 (4); p=0.003 |
| Head circumference-for-gestational age z score (HCGAZ) | 0.11 (0.02, 0.20)  1461 (3); p=0.019 | 0.13 (0.03, 0.24)  880 (2); p=0.014 | 0.13 (0.00, 0.25)  1114 (2); p=0.048 | 0.09 (-0.01, 0.19)  1615 (3); p=0.067 |
| Low HCZ | N/A | 0.87 (0.76, 0.99)  4218 (3); p=0.030 | 0.84 (0.75, 0.96)  4577 (3); p=0.008 | 0.86 (0.76, 0.97)  5204 (4); p=0.013 |
| Low HCGAZ | 0.74 (0.37, 1.51)  1461 (3); p=0.414 | 0.88 (0.76, 1.03)  4128 (3); p=0.105 | 0.88 (0.76, 1.02)  4577 (3); p=0.081 | 0.88 (0.77, 1.02)  5078 (4); p=0.092 |
| Mid-upper arm circumference (MUAC) (cm) | N/A | 0.08 (0.01, 0.14)  3786 (2); p=0.027 | 0.08 (0.02, 0.14)  4581 (3); p=0.009 | 0.08 (0.02, 0.14)  4581 (3); p=0.009 |
| Duration of gestation (wk) | 0.15 (-0.03, 0.33)  1880 (3); p=0.107 | N/A | 0.14 (0.01, 0.27)  4981 (3); p=0.031 | 0.10 (-0.02, 0.22)  5509 (4); p=0.105 |
| Preterm birth | 0.90 (0.68, 1.18)  1880 (3); p=0.446 | N/A | 0.94 (0.80, 1.10)  4981 (3); p=0.432 | 0.96 (0.82, 1.12)  5509 (4); p=0.612 |

^1^BMIZ, BMI-for-age z-score; HCGAZ, head circumference-for-gestational age z-score; HCZ, head circumference-for-age z-score; IFA, iron and folic acid supplement; LAZ, length-for-age z-score; LBW, low birth weight; LGA, large-for-gestational age; LGAZ, length-for-gestational age z-score; MD, mean difference; MUAC, mid-upper arum circumference; RR, relative risk; SGA, small-for-gestational age; SOC, standard of care; SQ-LNS, small-quantity lipid-based nutrient supplement; WAZ, weight-for-age z-score; WGAZ, weight-for-gestational age z-score

^2^For continuous outcomes, values are MDs: LNS – IFA/SOC (95% CIs). For binary outcomes, values are RRs: LNS compared with IFA/SOC (95% CIs).

**Supplemental Table 7B: Sensitivity analyses for main effects of SQ-LNS vs IFA/SOC on infant anthropometric outcomes at 6 mo of age^1^**

| **Infant anthropometric outcomes at 6 mo** | **MD or PR (95% CI)^2^**  **N (trials); p-value**  **Excluding Women First and DYAD-M Simple Follow-up** | **MD or PR (95% CI)^2^**  **N (trials); p-value**  **Excluding Guatemala** | **MD or PR (95% CI)^2^**  **N (trials); p-value**  **Including pre-conception** |
| --- | --- | --- | --- |
| Weight-for-age z-score (WAZ) | 0.02 (-0.06, 0.11)  4322 (3); p=0.589 | 0.02 (-0.06, 0.11)  4596 (3); p=0.602 | 0.05 (-0.02, 0.12)  5210 (4); p=0.176 |
| Underweight | 0.96 (0.80, 1.17)  4322 (3); p=0.714 | 0.96 (0.80, 1.16)  4596 (3); p=0.684 | 0.90 (0.78, 1.03)  5210 (4); p=0.123 |
| Length-for-age z-score (LAZ) | 0.04 (-0.03, 0.12)  4325 (3); p=0.267 | 0.04 (-0.04, 0.11)  4599 (3); p=0.311 | 0.06 (0.00, 0.13)  5213 (4); p=0.063 |
| Stunted | 0.95 (0.82, 1.10)  4325 (3); p=0.480 | 0.93 (0.81, 1.07)  4599 (3); p=0.325 | 0.88 (0.79, 0.99)  5213 (4); p=0.037 |
| Weight-for-length z-score (WLZ) | -0.01 (-0.10, 0.07)  4321 (3); p=0.769 | -0.01 (-0.09, 0.07)  4595 (3); p=0.824 | 0.00 (-0.07, 0.07)  5209 (4); p=0.960 |
| Wasted | 0.90 (0.65, 1.24)  4321 (3); p=0.507 | 0.95 (0.69, 1.31)  4595 (3); p=0.749 | 0.94 (0.69, 1.27)  5209 (4); p=0.675 |
| Head circumference-for-age z-score (HCZ) | 0.01 (-0.06, 0.07)  4324 (3); p=0.838 | 0.01 (-0.06, 0.07)  4598 (3); p=0.836 | 0.00 (-0.05, 0.06)  5209 (4); p=0.886 |
| Low HCZ | 0.91 (0.80, 1.04)  4324 (3); p=0.154 | 0.91 (0.81, 1.03)  4598 (3); p=0.149 | 0.93 (0.83, 1.05)  5209 (4); p=0.232 |
| MUAC-for-age z-score (MUACZ) | -0.01 (-0.09, 0.08)  4326 (3); p=0.896 | 0.00 (-0.07, 0.08)  4600 (3); p=0.949 | 0.00 (-0.07, 0.08)  4600 (3); p=0.949 |
| Low MUAC [MUACZ < -2 SD or MUAC < 125 mm] | 1.01 (0.78, 1.32)  4326 (3); p=0.930 | 1.00 (0.78, 1.29)  4600 (3); p=0.987 | 1.00 (0.78, 1.29)  4600 (3); p=0.987 |
| Acute malnutrition [WLZ < -2 SD or MUAC < 125 mm] | 0.99 (0.78, 1.25)  4321 (3); p=0.918 | 1.00 (0.79, 1.25)  4595 (3); p=0.970 | 1.00 (0.79, 1.25)  4595 (3); p=0.970 |

^1^HCZ, head circumference-for-age z-score; IFA, iron and folic acid supplement; LAZ, length-for-age z-score; MD, mean difference; MUAC, mid-upper arum circumference; MUACZ, mid-upper arm circumference-for-age z-score; PR, prevalence ratio; SOC, standard of care; SQ-LNS, small-quantity lipid-based nutrient supplement; WAZ, weight-for-age z-score; WLZ, weight-for-length z-score

^2^For continuous outcomes, values are MDs: LNS – IFA/SOC (95% CIs). For binary outcomes, values are PRs: LNS compared with IFA/SOC (95% CIs).

**Supplemental Table 7C: Sensitivity analyses for main effects of SQ-LNS vs IFA/SOC on adverse outcomes^1^**

| **Adverse outcomes** | **RR (95% CI)**  **N (trials); p-value**  **Excluding Women First and DYAD-M Simple Follow-up** | **RR (95% CI)**  **N (trials); p-value**  **Excluding Guatemala** | **RR (95% CI)**  **N (trials); p-value**  **Including pre-conception** |
| --- | --- | --- | --- |
| Cesarian-Section | 1.11 (0.90, 1.38)  4916 (3); p=0.339 | 1.12 (0.91, 1.38)  5228 (3); p=0.281 | 1.03 (0.90, 1.19)  5891 (4); p=0.642 |
| Miscarriage | 0.88 (0.69, 1.13)  5240 (3); p=0.319 | 0.89 (0.69, 1.13)  5565 (3); p=0.337 | 0.86 (0.70, 1.06)  6327 (4); p=0.150 |
| Stillbirth | 1.16 (0.78, 1.72)  5240 (3); p=0.457 | 1.24 (0.85, 1.81)  5565 (3); p=0.270 | 1.32 (0.91, 1.92)  6327 (4); p=0.146 |
| Miscarriage or stillbirth | 0.97 (0.77, 1.21)  5240 (3); p=0.785 | 1.00 (0.80, 1.25)  5565 (3); p=0.999 | 0.98 (0.82, 1.17)  6327 (4); p=0.793 |
| Early neonatal mortality | 0.78 (0.47, 1.32)  4746 (3); p=0.356 | 0.76 (0.47, 1.24)  5053 (3); p=0.277 | 0.76 (0.48, 1.20)  5698 (4); p=0.233 |
| Miscarriage or stillbirth or early neonatal mortality | 0.92 (0.74, 1.14)  5240 (3); p=0.445 | 0.94 (0.76, 1.16)  5565 (3); p=0.569 | 0.94 (0.78, 1.11)  6327 (4); p=0.454 |
| Neonatal mortality | 0.90 (0.57, 1.40)  4766 (3); p=0.635 | 0.84 (0.55, 1.28)  5076 (3); p=0.414 | 0.88 (0.60, 1.30)  5720 (4); p=0.521 |
| Mortality 0-6 mo | 0.99 (0.70, 1.40)  4809 (3); p=0.955 | 0.97 (0.69, 1.35)  5121 (3); p=0.843 | 0.98 (0.72, 1.33)  5754 (4); p=0.893 |

^1^GRADE, Grading of Recommendations Assessment, Development and Evaluation; IFA, iron and folic acid supplement; RR, relative risk; SOC, standard of care; SQ-LNS, small-quantity lipid-based nutrient supplement

^2^Values are RRs: LNS compared with IFA/SOC (95% CIs).
