# Supplementary material for: Effects of prenatal small-quantity lipid-based nutrient supplements on pregnancy, birth, and infant outcomes: a systematic review and meta-analysis of individual participant data from randomized controlled trials in low- and middle-income countries
Source: Am J Clin Nutr. 2024 Aug 16;120(4):814–35. doi: 10.1016/j.ajcnut.2024.08.008 (PMC11473441; doi:10.1016/j.ajcnut.2024.08.008)

Supplemental figure 2: Forest plots of main effects for all outcomes, SQ-LNS vs IFA/SOC

Contents

Supplemental figure 2A: Mean difference in birth weight (g) **5**

Supplemental figure 2B: Mean difference in birth weight-for-age z score **6**

Supplemental figure 2C: Mean difference in weight-for-gestational age z-score **7**

Supplemental figure 2D: Low birth weight relative risk **8**

Supplemental figure 2E: Low birth weight risk difference **9**

Supplemental figure 2F: Birth weight < 2 kg relative risk **10**

Supplemental figure 2G: Birth weight < 2 kg risk difference **11**

Supplemental figure 2H: Small-for-gestational age relative risk **12**

Supplemental figure 2I: Small-for-gestational age risk difference **13**

Supplemental figure 2J: Large-for-gestational age relative risk **14**

Supplemental figure 2K: Large-for-gestational age risk difference **15**

Supplemental figure 2L: Mean difference in birth length (cm) **16**

Supplemental figure 2M: Mean difference in birth length-for-age z score **17**

Supplemental figure 2N: Mean difference in length-for-gestational age z-score **18**

Supplemental figure 2O: Newborn stunting relative risk **19**

Supplemental figure 2P: Newborn stunting risk difference **20**

Supplemental figure 2Q: Low LGAZ relative risk **21**

Supplemental figure 2R: Low LGAZ risk difference **22**

Supplemental figure 2S: Mean difference in birth BMI-for-age z-score **23**

Supplemental figure 2T: Low BMIZ relative risk **24**

Supplemental figure 2U: Low BMIZ risk difference **25**

Supplemental figure 2V: Mean difference in birth head circumference (cm) **26**

Supplemental figure 2W: Mean difference in birth head circumference-for-age z score **27**

Supplemental figure 2X: Mean difference in birth head circumference-for-gestational age z score **28**

Supplemental figure 2Y: Low HCZ relative risk **29**

Supplemental figure 2Z: Low HCZ risk difference **30**

Supplemental figure 2AA: Low HCGAZ relative risk **31**

Supplemental figure 2AB: Low HCGAZ risk difference **32**

Supplemental figure 2AC: Mean difference in birth mid-upper arm circumference (cm) **33**

Supplemental figure 2AD: Mean difference in duration of gestation (wk) **34**

Supplemental figure 2AE: Preterm birth relative risk **35**

Supplemental figure 2AF: Preterm birth risk difference **36**

Supplemental figure 2AG: Mean difference in 6 mo weight-for-age z-score 37

Supplemental figure 2AH: 6 mo underweight prevalence ratio 38

Supplemental figure 2AI: 6 mo underweight prevalence difference 39

Supplemental figure 2AJ: Mean difference in 6 mo length-for-age z-score 40

Supplemental figure 2AK: 6 mo stunting prevalence ratio 41

Supplemental figure 2AL: 6 mo stunting prevalence difference 42

Supplemental figure 2AM: Mean difference in 6 mo weight-for-length z-score 43

Supplemental figure 2AN: 6 mo wasting prevalence ratio 44

Supplemental figure 2AO: 6 mo wasting prevalence difference 45

Supplemental figure 2AP: Mean difference in 6 mo head circumference-for-age z-score 46

Supplemental figure 2AQ: 6 mo low HCZ prevalence ratio 47

Supplemental figure 2AR: 6 mo low HCZ prevalence difference 48

Supplemental figure 2AS: Mean difference in 6 mo MUAC-for-age z-score 49

Supplemental figure 2AT: 6 mo low MUAC prevalence ratio 50

Supplemental figure 2AU: 6 mo low MUAC prevalence difference 51

Supplemental figure 2AV: 6 mo acute malnutrition prevalence ratio 52

Supplemental figure 2AW: 6 mo acute malnutrition prevalence difference 53

Supplemental figure 2AX: Cesarian-section relative risk 54

Supplemental figure 2AY: Cesarian-section risk difference **55**

Supplemental figure 2AZ: Miscarriage relative risk **56**

Supplemental figure 2BA: Miscarriage risk difference **57**

Supplemental figure 2BB: Stillbirth relative risk **58**

Supplemental figure 2BC: Stillbirth risk difference **59**

Supplemental figure 2BD: Miscarriage or stillbirth relative risk **60**

Supplemental figure 2BE: Miscarriage or stillbirth risk difference **61**

Supplemental figure 2BF: Early neonatal mortality relative risk **62**

Supplemental figure 2BG: Early neonatal mortality risk difference **63**

Supplemental figure 2BH: Miscarriage or stillbirth or early neonatal mortality relative risk **64**

Supplemental figure 2BI: Miscarriage or stillbirth or early neonatal mortality risk difference **65**

Supplemental figure 2BJ: Neonatal mortality relative risk **66**

Supplemental figure 2BK: Neonatal mortality risk difference **67**

Supplemental figure 2BL: Mortality 0-6 mo relative risk **68**

Supplemental figure 2BM: Mortality 0-6 mo risk difference **69**

These figures are forest plots showing the study-level estimates of intervention effect with the pooled estimate in the bottom summary rows. Individual study estimates were generated from log binomial regression for dichotomous outcomes and simple linear regression for continuous outcomes with clustered observations using robust standard errors for cluster-randomized trials. Pooled estimates were generated using inverse variance weighting in both fixed and random effects models. For continuous outcomes the intervention effect is measured by the difference in mean of the SQ-LNS group minus IFA/SOC. For dichotomous outcomes analyzed via prevalence/risk ratios the effect estimate is the prevalence/risk in the SQ-LNS group divided by the prevalence/risk in the IFA/SOC group. For dichotomous outcomes analyzed via prevalence/risk differences the effect estimate is the prevalence/risk in the SQ-LNS group minus the prevalence/risk in the IFA/SOC group. The labels on the left y-axis correspond to trial level information. The values on the right indicate the study level effect estimate, confidence interval, and weighting for deriving the pooled estimate.

LAZ, length-for-age z-score; WLZ, weight-for-length z-score; WAZ, weight for-age z-score; MUACZ, mid-upper arm circumference z-score; BMI, body mass index; HCZ, head circumference-for-age z-score; LGAZ, length-for-gestational-age z-score; HCGAZ, head circumference-for-gestational-age z-score; BMIZ, body mass index-for-age z-score; IFA/SOC, Iron and folic acid or standard of care; MD, mean difference; MMS, multiple micronutrient supplement; MUAC, mid-upper arm circumference; PR, prevalence ratio; PD, prevalence difference; RD, risk difference; RR, relative risk; SOC, standard of care; SQ-LNS, small-quantity lipid-based nutrient supplements; WGAZ, weight-for-gestational age z-score.

Supplemental figure 2A: Mean difference in birth weight (g)

Country	Trial	SQ−LNS N	IFA/SOC N	MD (95% CI)	Fixed W	Random W
Ghana	DYAD−G	307	305	80 (12, 148)	0.11	0.11
Malawi	DYAD−M	387	386	48 (−16, 112)	0.12	0.12
Bangladesh	RDNS	898	2551	41 (11, 71)	0.55	0.55
Guatemala	WOMEN FIRST	223	216	53 (4, 101)	0.21	0.21
**I² = 0.00, Tau² = 0.00**		**1815**	**3458**			
**Fixed**				**49 (26, 71)**		
**Random**				**49 (26, 71)**		

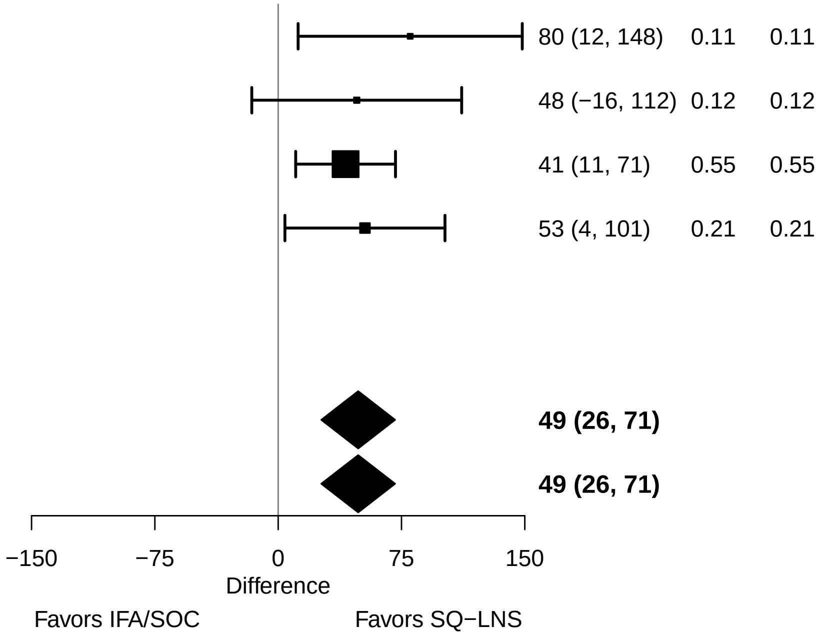

Supplemental figure 2B: Mean difference in birth weight-for-age z score

Country	Trial	SQ−LNS N	IFA/SOC N	MD (95% CI)	Fixed W	Random W
Ghana	DYAD−G	307	305	0.18 (0.03, 0.33)	0.13	0.13
Malawi	DYAD−M	387	386	0.11 (−0.04, 0.26)	0.14	0.14
Bangladesh	RDNS	898	2551	0.10 (0.03, 0.18)	0.54	0.54
Guatemala	WOMEN FIRST	223	216	0.13 (0.01, 0.26)	0.20	0.20
**I² = 0.00, Tau² = 0.00**		**1815**	**3458**			
**Fixed**				**0.12 (0.06, 0.17)**		
**Random**				**0.12 (0.06, 0.17)**		

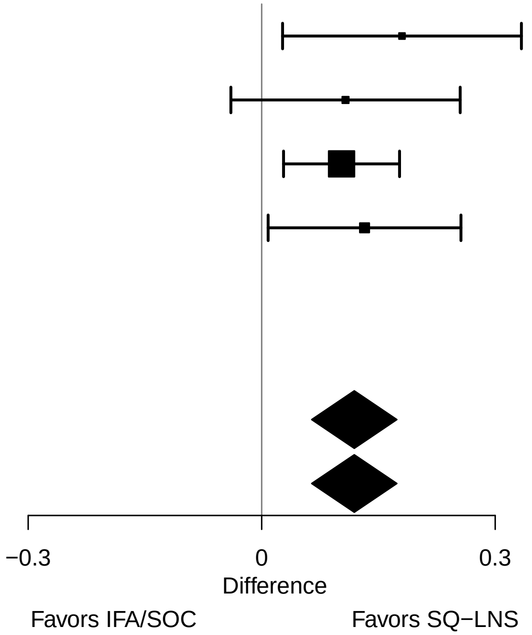

Supplemental figure 2C: Mean difference in weight-for-gestational age z-score

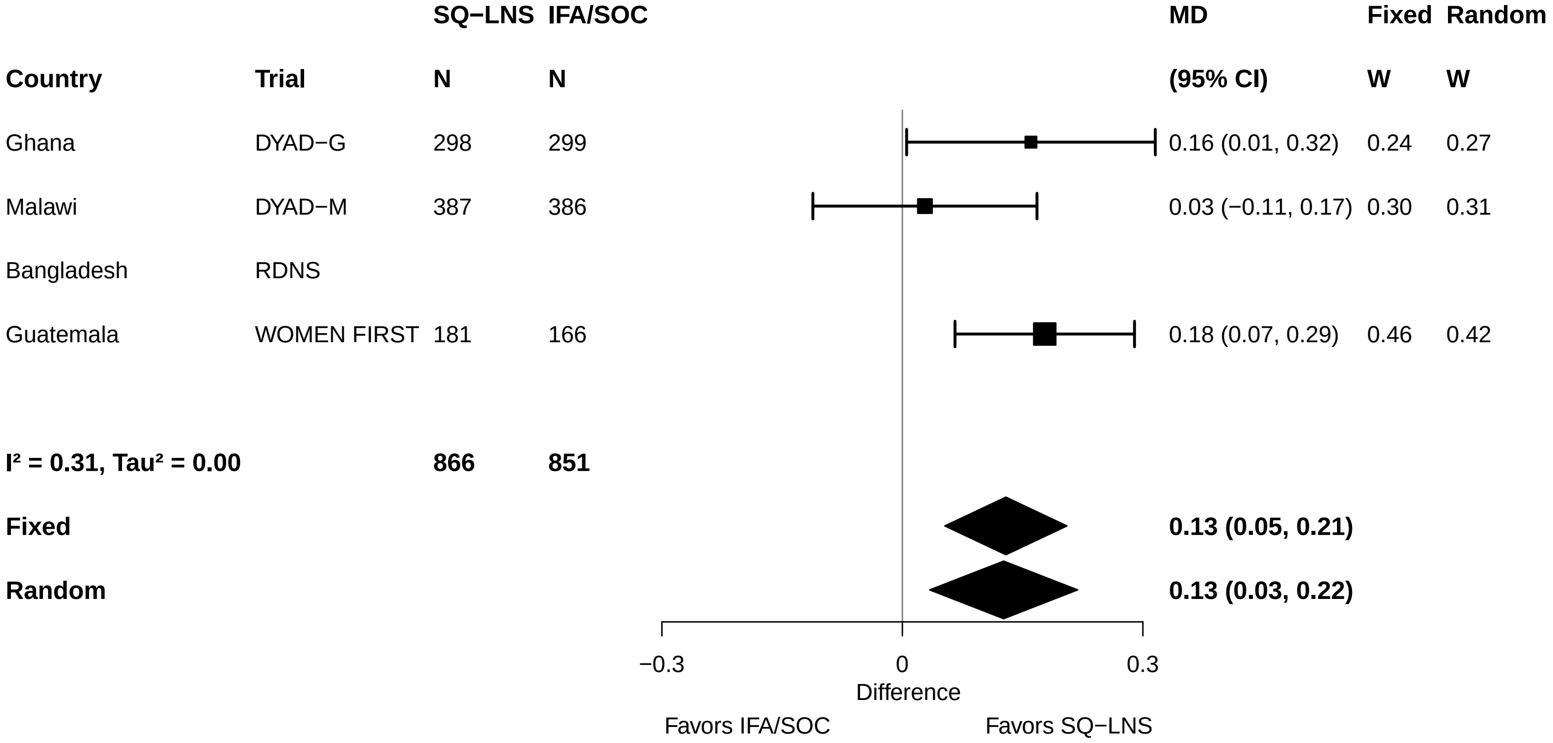

Supplemental figure 2D: Low birth weight relative risk

Country	Trial	SQ−LNS N	IFA/SOC N	RR (95% CI)	Fixed W	Random W
Ghana	DYAD−G	307	305	0.62 (0.39, 0.98)	0.05	0.05
Malawi	DYAD−M	387	386	0.92 (0.64, 1.32)	0.08	0.08
Bangladesh	RDNS	898	2551	0.92 (0.82, 1.03)	0.78	0.78
Guatemala	WOMEN FIRST	223	216	0.84 (0.60, 1.19)	0.09	0.09
**I² = 0.00, Tau² = 0.00**		**1815**	**3458**			
**Fixed**				**0.89 (0.80, 0.99)**		
**Random**				**0.89 (0.80, 0.99)**		

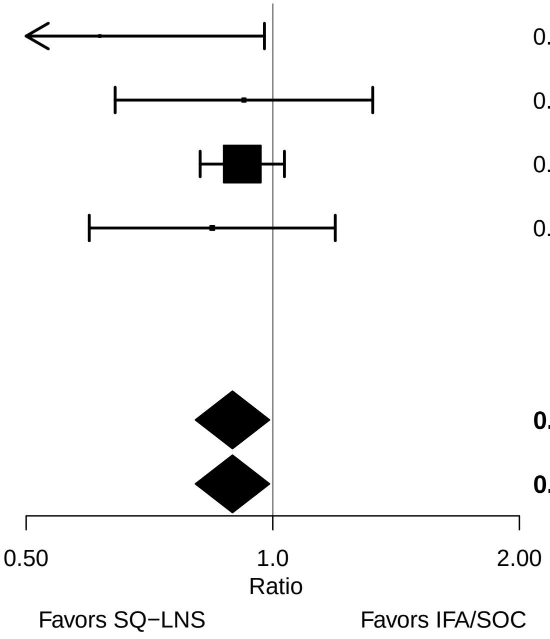

Supplemental figure 2E: Low birth weight risk difference

Country	Trial	SQ−LNS N	IFA/SOC N	RD (95% CI)	Fixed W	Random W
Ghana	DYAD−G	307	305	−0.05 (−0.10, 0.00)	0.21	0.21
Malawi	DYAD−M	387	386	−0.01 (−0.06, 0.04)	0.22	0.22
Bangladesh	RDNS	898	2551	−0.03 (−0.07, 0.01)	0.31	0.31
Guatemala	WOMEN FIRST	223	216	−0.02 (−0.07, 0.02)	0.26	0.26
**I² = 0.00, Tau² = 0.00**		**1815**	**3458**			
**Fixed**				**−0.03 (−0.05, −0.01)**		
**Random**				**−0.03 (−0.05, −0.01)**		

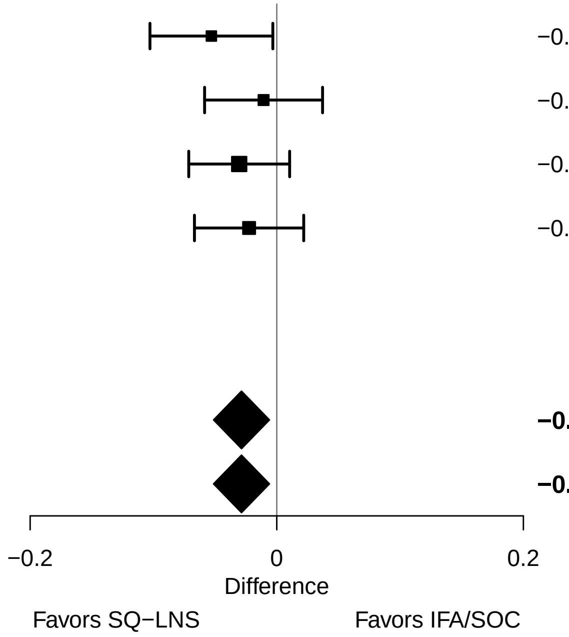

Supplemental figure 2F: Birth weight < 2 kg relative risk

Country	Trial	SQ−LNS N	IFA/SOC N	RR (95% CI)	Fixed W	Random W
Ghana	DYAD−G	307	305	0.43 (0.11, 1.63)	0.04	0.04
Malawi	DYAD−M	387	386	1.00 (0.45, 2.19)	0.11	0.11
Bangladesh	RDNS	898	2551	0.78 (0.59, 1.04)	0.84	0.84
Guatemala	WOMEN FIRST	223	216	0.48 (0.04, 5.69)	0.01	0.01
**I² = 0.00, Tau² = 0.00**		**1815**	**3458**			
**Fixed**				**0.78 (0.60, 1.01)**		
**Random**				**0.78 (0.60, 1.01)**		

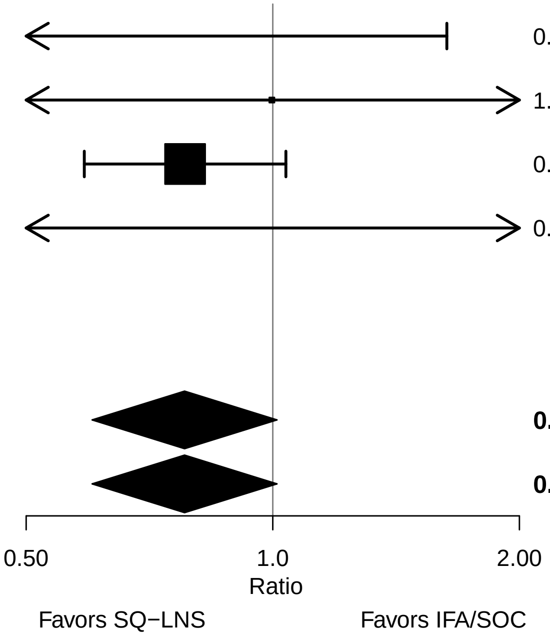

Supplemental figure 2G: Birth weight < 2 kg risk difference

Country	Trial	SQ−LNS N	IFA/SOC N	RD (95% CI)	Fixed W	Random W
Ghana	DYAD−G	307	305	−0.01 (−0.03, 0.01)	0.21	0.21
Malawi	DYAD−M	387	386	0.00 (−0.02, 0.02)	0.14	0.14
Bangladesh	RDNS	898	2551	−0.02 (−0.03, 0.00)	0.29	0.29
Guatemala	WOMEN FIRST	223	216	0.00 (−0.02, 0.01)	0.36	0.36
**I² = 0.00, Tau² = 0.00**		**1815**	**3458**			
**Fixed**				**−0.01 (−0.02, 0.00)**		
**Random**				**−0.01 (−0.02, 0.00)**		

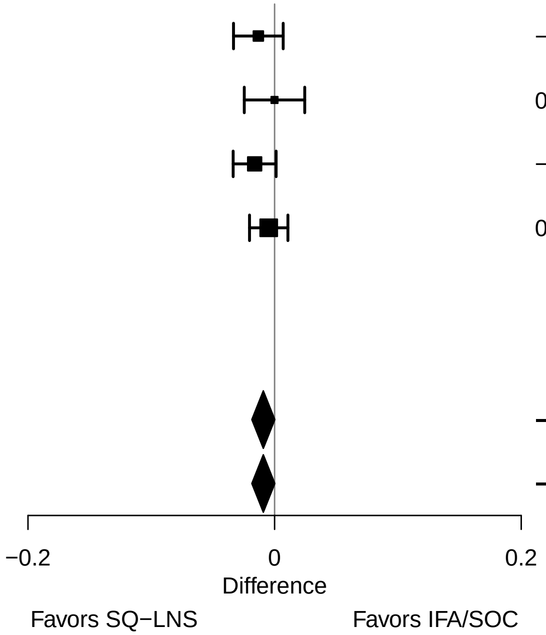

Supplemental figure 2H: Small-for-gestational age relative risk

Country	Trial	SQ−LNS N	IFA/SOC N	RR (95% CI)	Fixed W	Random W
Ghana	DYAD−G	307	305	0.82 (0.60, 1.12)	0.02	0.02
Malawi	DYAD−M	387	386	0.96 (0.75, 1.22)	0.04	0.04
Bangladesh	RDNS	898	2551	0.97 (0.92, 1.02)	0.91	0.91
Guatemala	WOMEN FIRST	181	166	0.77 (0.56, 1.07)	0.02	0.02
**I² = 0.00, Tau² = 0.00**		**1773**	**3408**			
**Fixed**				**0.96 (0.92, 1.01)**		
**Random**				**0.96 (0.92, 1.01)**		

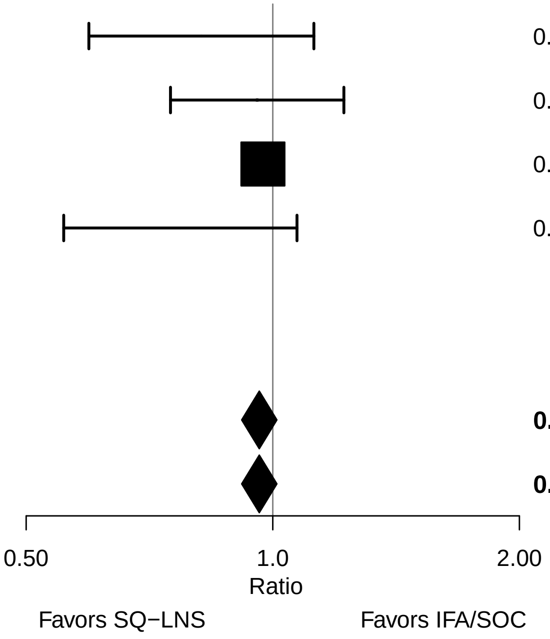

Supplemental figure 2I: Small-for-gestational age risk difference

Country	Trial	SQ−LNS N	IFA/SOC N	RD (95% CI)	Fixed W	Random W
Ghana	DYAD−G	307	305	−0.04 (−0.10, 0.02)	0.14	0.14
Malawi	DYAD−M	387	386	−0.01 (−0.07, 0.05)	0.15	0.15
Bangladesh	RDNS	898	2551	−0.02 (−0.05, 0.01)	0.62	0.62
Guatemala	WOMEN FIRST	181	166	−0.06 (−0.14, 0.02)	0.10	0.10
**I² = 0.00, Tau² = 0.00**		**1773**	**3408**			
**Fixed**				**−0.02 (−0.05, 0.00)**		
**Random**				**−0.02 (−0.05, 0.00)**		

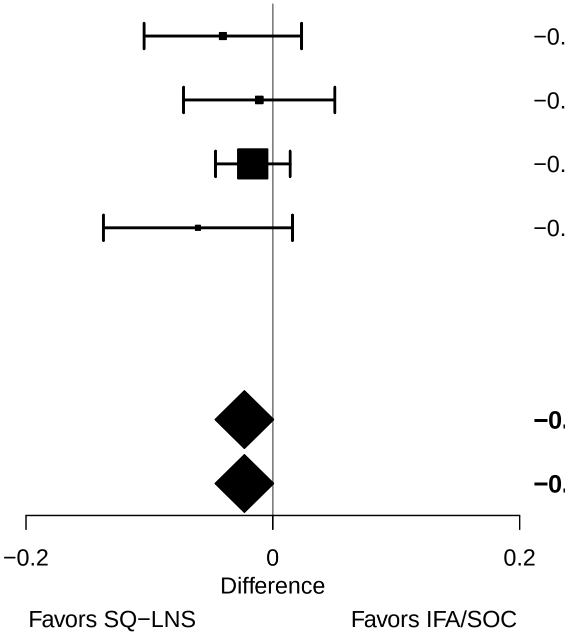

Supplemental figure 2J: Large-for-gestational age relative risk

**Country**	**Trial**	**SQ−LNS N**	**IFA/SOC N**	**RR (95% CI)**	**Fixed W**	**Random W**
Ghana	DYAD−G	307	305	0.99 (0.45, 2.18)	0.40	0.40
Malawi	DYAD−M	387	386	0.84 (0.38, 1.86)	0.39	0.39
Bangladesh	RDNS	898	2551	1.42 (0.48, 4.17)	0.21	0.21
Guatemala	WOMEN FIRST					
**I² = 0.00, Tau² = 0.00**		**1592**	**3242**			
**Fixed**				**1.00 (0.61, 1.65)**		
**Random**				**1.00 (0.61, 1.65)**		

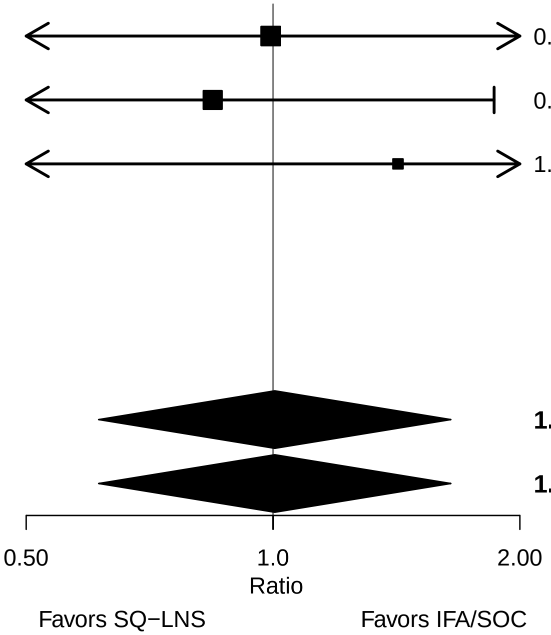

Supplemental figure 2K: Large-for-gestational age risk difference

Country	Trial	SQ−LNS N	IFA/SOC N	RD (95% CI)	Fixed W	Random W
Ghana	DYAD−G	307	305	0.00 (−0.03, 0.03)	0.02	0.02
Malawi	DYAD−M	387	386	−0.01 (−0.03, 0.02)	0.03	0.03
Bangladesh	RDNS	898	2551	0.00 (0.00, 0.01)	0.95	0.95
Guatemala	WOMEN FIRST					
**$I^2 = 0.00$, $Tau^2 = 0.00$**		**1592**	**3242**			
**Fixed**				**0.00 (0.00, 0.01)**		
**Random**				**0.00 (0.00, 0.01)**		

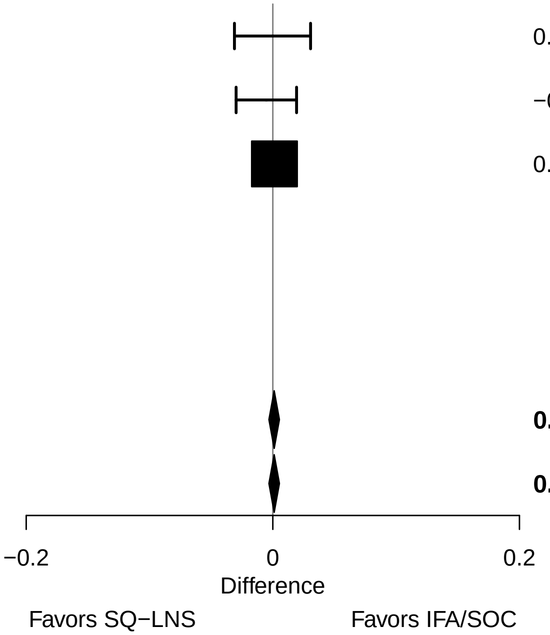

Supplemental figure 2L: Mean difference in birth length (cm)

Country	Trial	SQ−LNS N	IFA/SOC N	MD (95% CI)	Fixed W	Random W
Ghana	DYAD−G	307	305	0.25 (−0.05, 0.55)	0.12	0.12
Malawi	DYAD−M	253	263	0.30 (−0.07, 0.66)	0.08	0.08
Bangladesh	RDNS	897	2550	0.17 (0.03, 0.31)	0.57	0.57
Guatemala	WOMEN FIRST	223	216	0.22 (−0.01, 0.44)	0.23	0.23
**I² = 0.00, Tau² = 0.00**		**1680**	**3334**			
**Fixed**				**0.20 (0.09, 0.31)**		
**Random**				**0.20 (0.09, 0.31)**		

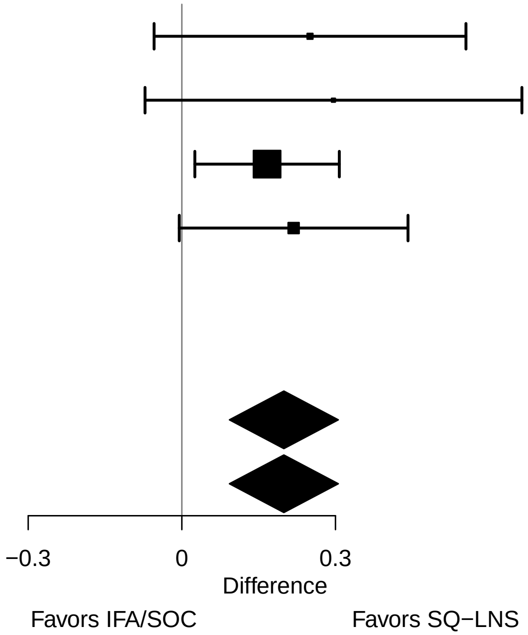

Supplemental figure 2M: Mean difference in birth length-for-age z score

Country	Trial	SQ−LNS N	IFA/SOC N	MD (95% CI)	Fixed W	Random W
Ghana	DYAD−G	307	305	0.12 (−0.04, 0.28)	0.13	0.13
Malawi	DYAD−M	253	263	0.17 (−0.02, 0.37)	0.09	0.09
Bangladesh	RDNS	897	2550	0.09 (0.02, 0.17)	0.60	0.60
Guatemala	WOMEN FIRST	223	216	0.14 (0.01, 0.27)	0.19	0.19
**I² = 0.00, Tau² = 0.00**		**1680**	**3334**			
**Fixed**				**0.11 (0.06, 0.17)**		
**Random**				**0.11 (0.06, 0.17)**		

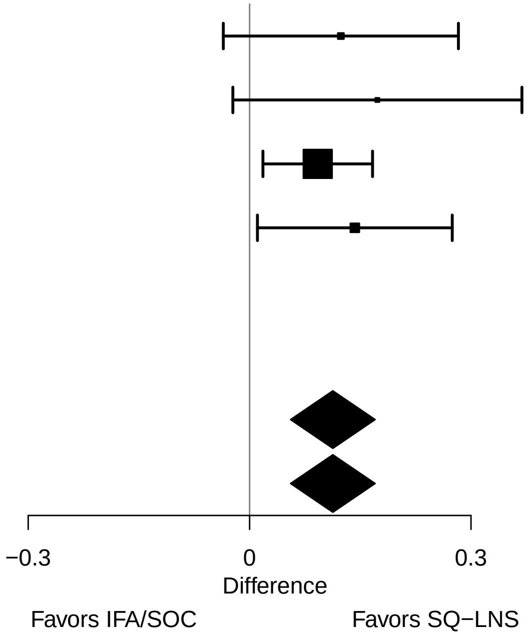

Supplemental figure 2N: Mean difference in length-for-gestational age z-score

Country	Trial	SQ−LNS N	IFA/SOC N	MD (95% CI)	Fixed W	Random W
Ghana	DYAD−G	298	299	0.10 (−0.06, 0.26)	0.26	0.26
Malawi	DYAD−M	253	263	0.09 (−0.11, 0.28)	0.17	0.17
Bangladesh	RDNS					
Guatemala	WOMEN FIRST	181	166	0.15 (0.04, 0.26)	0.57	0.57
**I² = 0.00, Tau² = 0.00**		**732**	**728**			
**Fixed**				**0.13 (0.05, 0.21)**		
**Random**				**0.13 (0.05, 0.21)**		

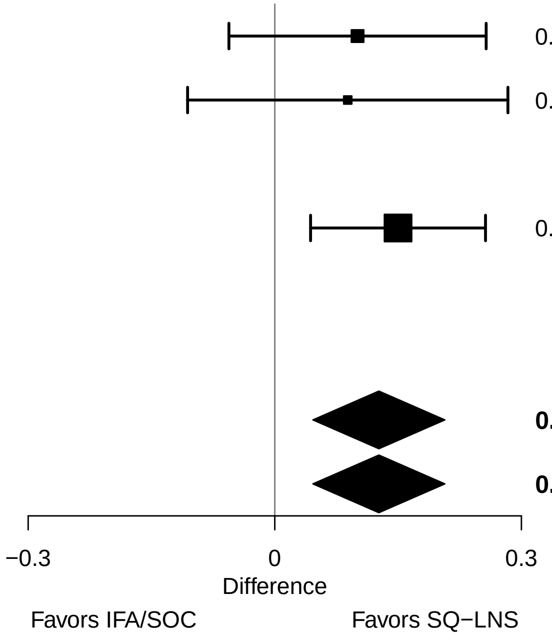

Supplemental figure 2O: Newborn stunting relative risk

Country	Trial	SQ−LNS N	IFA/SOC N	RR (95% CI)	Fixed W	Random W
Ghana	DYAD−G	307	305	0.99 (0.61, 1.61)	0.06	0.06
Malawi	DYAD−M	253	263	0.85 (0.57, 1.28)	0.08	0.08
Bangladesh	RDNS	897	2550	0.83 (0.73, 0.95)	0.74	0.74
Guatemala	WOMEN FIRST	223	216	0.73 (0.53, 1.02)	0.12	0.12
**I² = 0.00, Tau² = 0.00**		**1680**	**3334**			
**Fixed**				**0.83 (0.74, 0.93)**		
**Random**				**0.83 (0.74, 0.93)**		

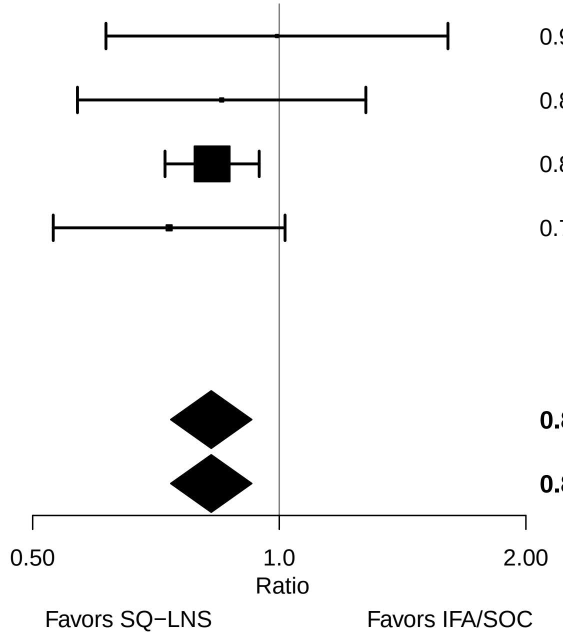

Supplemental figure 2P: Newborn stunting risk difference

Country	Trial	SQ−LNS N	IFA/SOC N	RD (95% CI)	Fixed W	Random W
Ghana	DYAD−G	307	305	0.00 (−0.05, 0.05)	0.17	0.17
Malawi	DYAD−M	253	263	−0.03 (−0.09, 0.04)	0.10	0.10
Bangladesh	RDNS	897	2550	−0.04 (−0.07, −0.01)	0.51	0.51
Guatemala	WOMEN FIRST	223	216	−0.04 (−0.08, 0.00)	0.22	0.22
**I² = 0.00, Tau² = 0.00**		**1680**	**3334**			
**Fixed**				**−0.03 (−0.05, −0.01)**		
**Random**				**−0.03 (−0.05, −0.01)**		

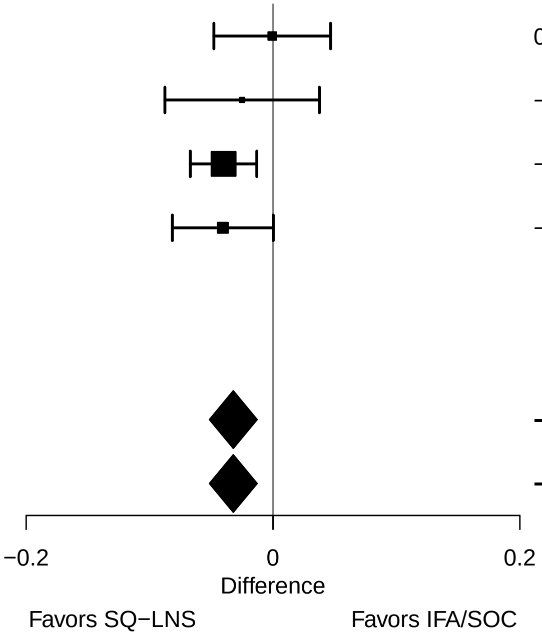

Supplemental figure 2Q: Low LGAZ relative risk

Country	Trial	SQ−LNS N	IFA/SOC N	RR (95% CI)	Fixed W	Random W
Ghana	DYAD−G	307	305	1.19 (0.61, 2.32)	0.03	0.03
Malawi	DYAD−M	253	263	0.93 (0.63, 1.36)	0.09	0.09
Bangladesh	RDNS	897	2550	0.89 (0.78, 1.01)	0.83	0.83
Guatemala	WOMEN FIRST	181	166	0.85 (0.49, 1.46)	0.05	0.05
**I² = 0.00, Tau² = 0.00**		**1638**	**3284**			
**Fixed**				**0.90 (0.80, 1.01)**		
**Random**				**0.90 (0.80, 1.01)**		

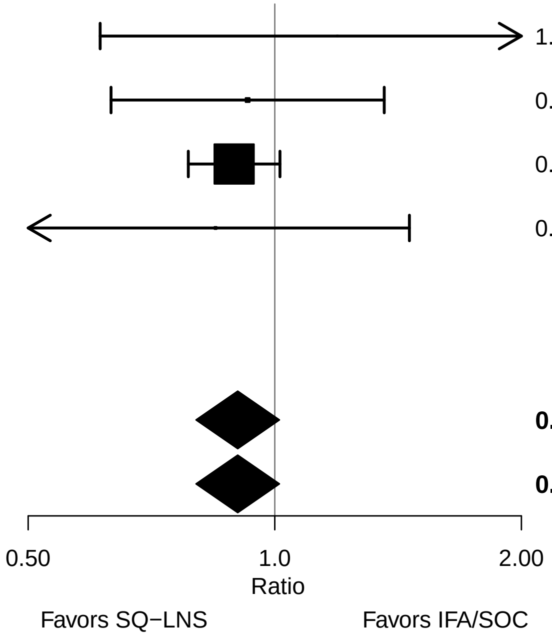

Supplemental figure 2R: Low LGAZ risk difference

Country	Trial	SQ−LNS N	IFA/SOC N	RD (95% CI)	Fixed W	Random W
Ghana	DYAD−G	307	305	0.01 (−0.03, 0.05)	0.24	0.24
Malawi	DYAD−M	253	263	−0.01 (−0.08, 0.05)	0.07	0.07
Bangladesh	RDNS	897	2550	−0.02 (−0.05, 0.00)	0.49	0.49
Guatemala	WOMEN FIRST	181	166	−0.01 (−0.05, 0.03)	0.19	0.19
**I² = 0.00, Tau² = 0.00**		**1638**	**3284**			
**Fixed**				**−0.01 (−0.03, 0.01)**		
**Random**				**−0.01 (−0.03, 0.01)**		

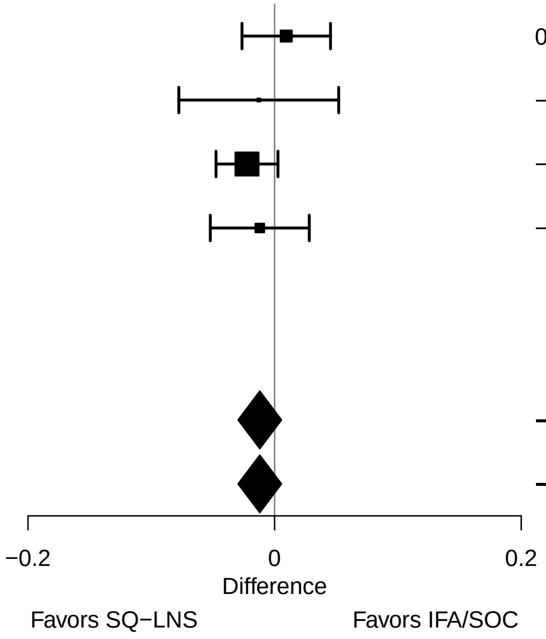

Supplemental figure 2S: Mean difference in birth BMI-for-age z-score

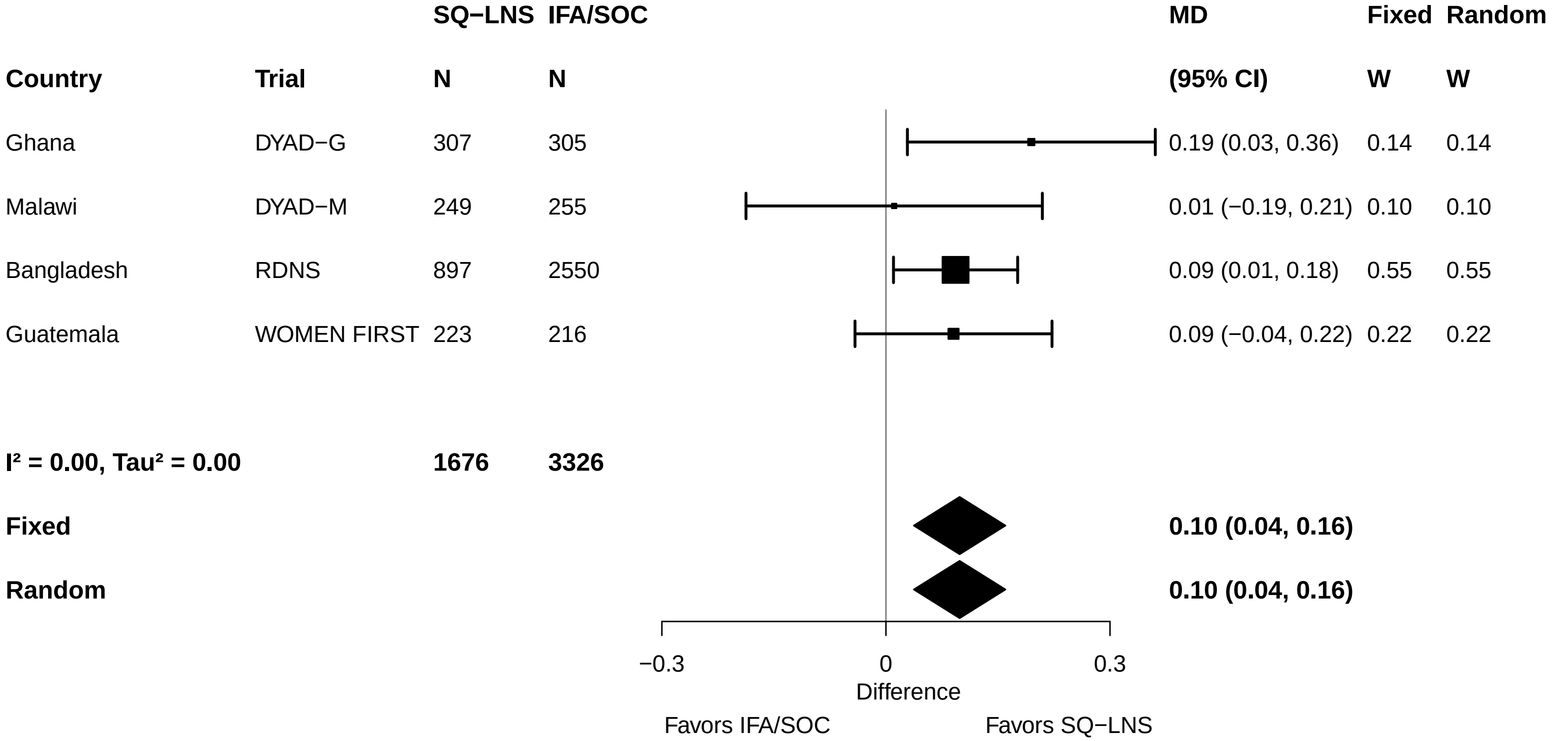

Supplemental figure 2T: Low BMIZ relative risk

Country	Trial	SQ−LNS N	IFA/SOC N	RR (95% CI)	Fixed W	Random W
Ghana	DYAD−G	307	305	0.54 (0.31, 0.94)	0.03	0.15
Malawi	DYAD−M	249	255	1.14 (0.62, 2.10)	0.02	0.12
Bangladesh	RDNS	897	2550	0.91 (0.82, 1.00)	0.92	0.62
Guatemala	WOMEN FIRST	223	216	0.76 (0.40, 1.46)	0.02	0.11
**I² = 0.26, Tau² = 0.02**		**1676**	**3326**			
**Fixed**				**0.89 (0.81, 0.98)**		
**Random**				**0.85 (0.67, 1.08)**		

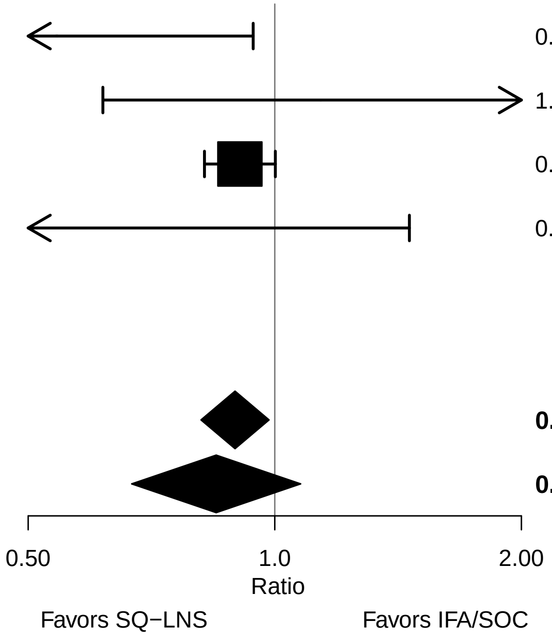

Supplemental figure 2U: Low BMIZ risk difference

Country	Trial	SQ−LNS N	IFA/SOC N	RD (95% CI)	Fixed W	Random W
Ghana	DYAD−G	307	305	−0.05 (−0.09, −0.01)	0.19	0.21
Malawi	DYAD−M	249	255	0.01 (−0.04, 0.06)	0.17	0.19
Bangladesh	RDNS	897	2550	−0.03 (−0.06, 0.00)	0.37	0.34
Guatemala	WOMEN FIRST	223	216	−0.02 (−0.05, 0.02)	0.27	0.27
**I² = 0.20, Tau² = 0.00**		**1676**	**3326**			
**Fixed**				**−0.02 (−0.04, 0.00)**		
**Random**				**−0.02 (−0.05, 0.00)**		

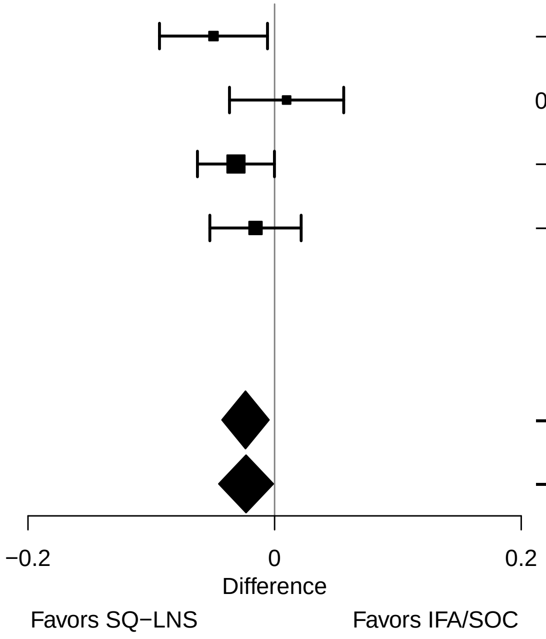

Supplemental figure 2V: Mean difference in birth head circumference (cm)

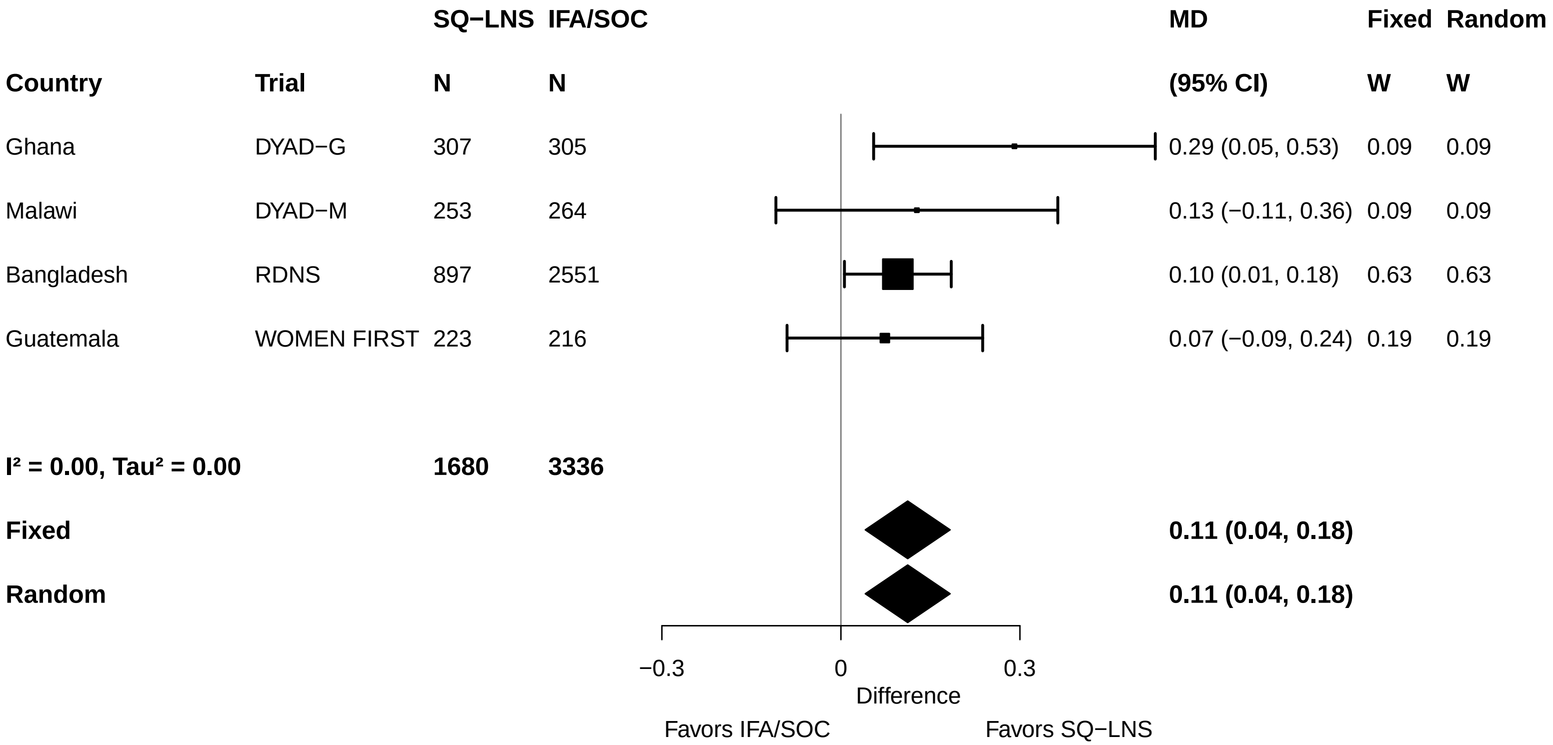

Supplemental figure 2W: Mean difference in birth head circumference-for-age z score

Country	Trial	SQ−LNS N	IFA/SOC N	MD (95% CI)	Fixed W	Random W
Ghana	DYAD−G	307	305	0.21 (0.03, 0.39)	0.10	0.10
Malawi	DYAD−M	253	264	0.12 (−0.07, 0.31)	0.09	0.09
Bangladesh	RDNS	897	2551	0.08 (0.01, 0.16)	0.62	0.62
Guatemala	WOMEN FIRST	223	216	0.09 (−0.04, 0.23)	0.19	0.19
**I² = 0.00, Tau² = 0.00**		**1680**	**3336**			
**Fixed**				**0.10 (0.04, 0.16)**		
**Random**				**0.10 (0.04, 0.16)**		

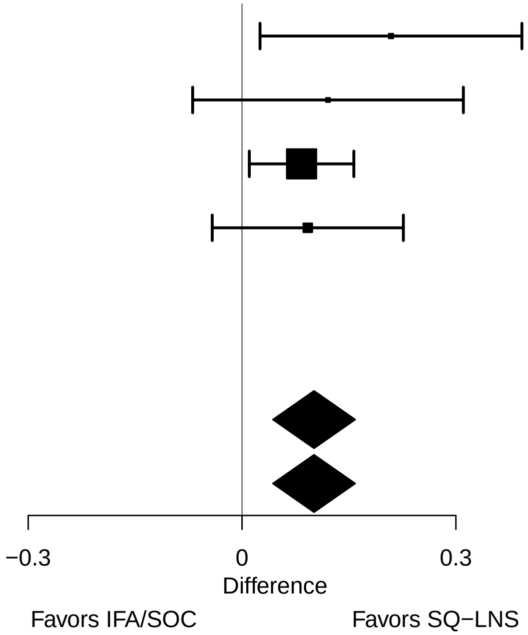

Supplemental figure 2X: Mean difference in birth head circumference-for-gestational age z score

Country	Trial	SQ−LNS N	IFA/SOC N	MD (95% CI)	Fixed W	Random W
Ghana	DYAD−G	298	299	0.19 (0.02, 0.37)	0.27	0.27
Malawi	DYAD−M	253	264	0.06 (−0.12, 0.24)	0.27	0.27
Bangladesh	RDNS					
Guatemala	WOMEN FIRST	181	166	0.09 (−0.04, 0.23)	0.46	0.46
**I² = 0.00, Tau² = 0.00**		**732**	**729**			
**Fixed**				**0.11 (0.02, 0.20)**		
**Random**				**0.11 (0.02, 0.20)**		

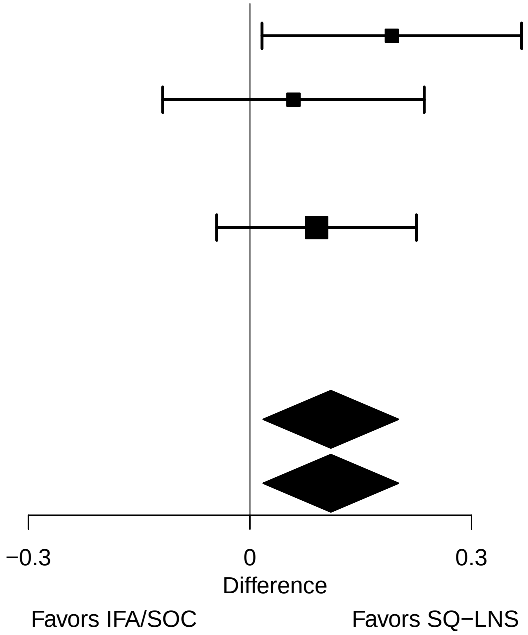

Supplemental figure 2Y: Low HCZ relative risk

Country	Trial	SQ−LNS N	IFA/SOC N	RR (95% CI)	Fixed W	Random W
Ghana	DYAD−G	307	305	0.83 (0.47, 1.47)	0.05	0.05
Malawi	DYAD−M	253	264	0.78 (0.38, 1.62)	0.03	0.03
Bangladesh	RDNS	897	2551	0.85 (0.75, 0.96)	0.89	0.89
Guatemala	WOMEN FIRST	223	216	0.97 (0.53, 1.77)	0.04	0.04
**I² = 0.00, Tau² = 0.00**		**1680**	**3336**			
**Fixed**				**0.85 (0.75, 0.96)**		
**Random**				**0.85 (0.75, 0.96)**		

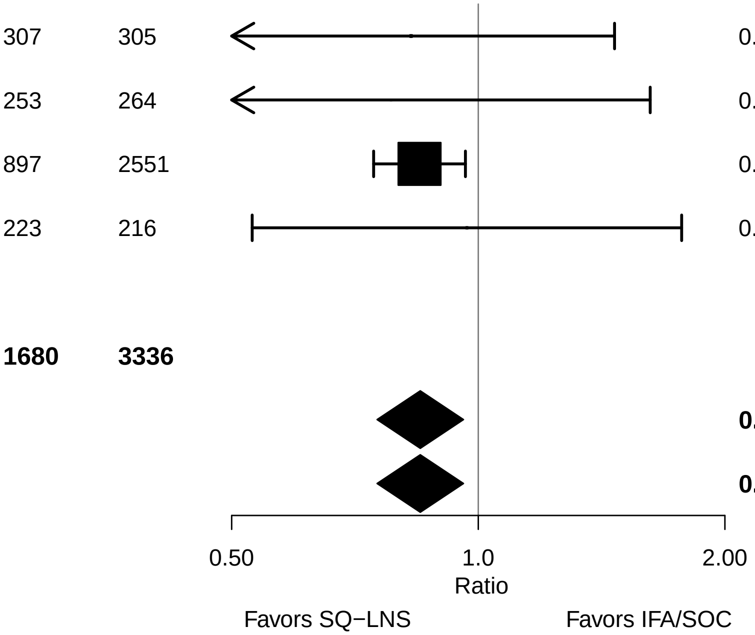

Supplemental figure 2Z: Low HCZ risk difference

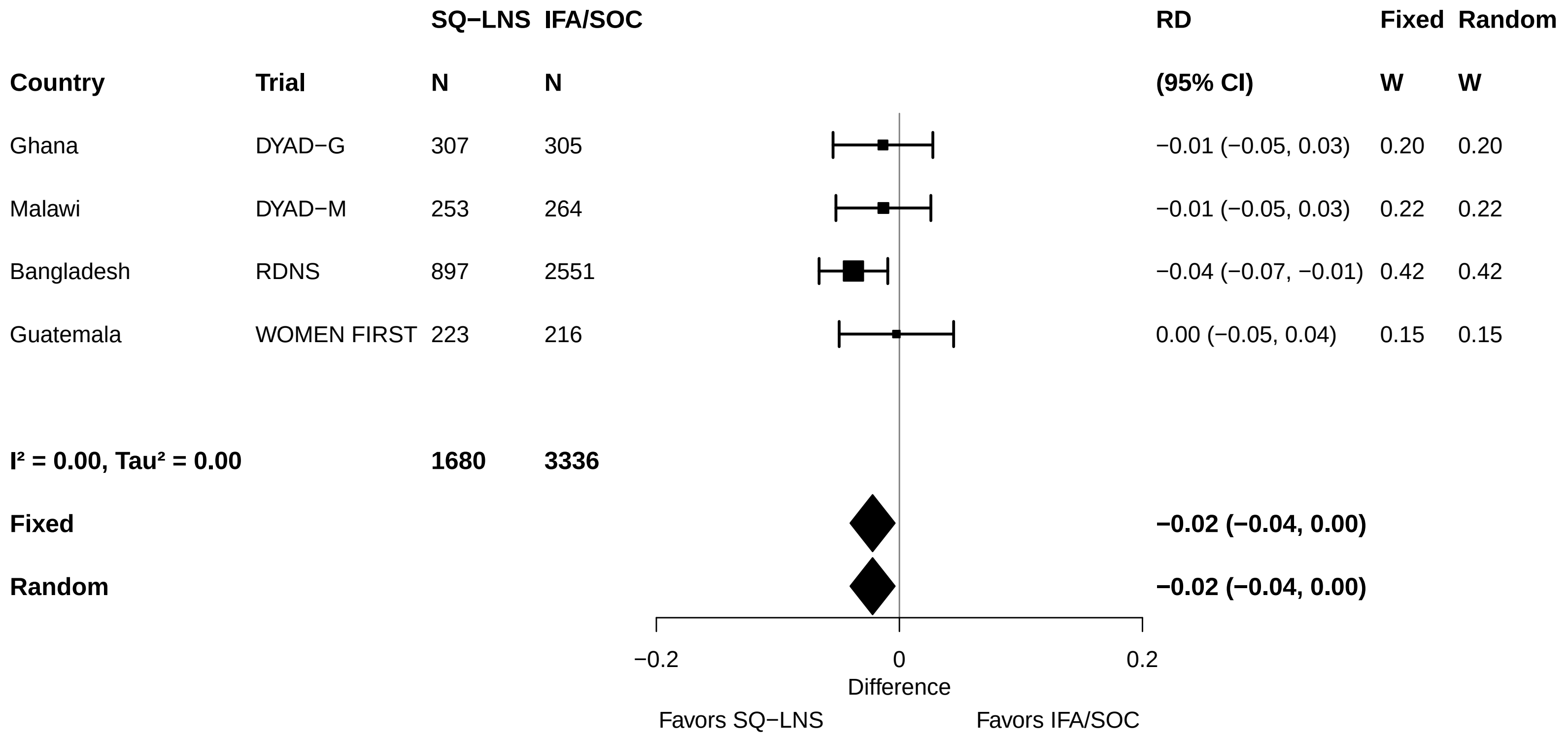

Supplemental figure 2AA: Low HCGAZ relative risk

Country	Trial	SQ−LNS N	IFA/SOC N	RR (95% CI)	Fixed W	Random W
Ghana	DYAD−G	307	305	0.50 (0.17, 1.44)	0.02	0.02
Malawi	DYAD−M	253	264	2.09 (0.39, 11.29)	0.01	0.01
Bangladesh	RDNS	897	2551	0.88 (0.76, 1.02)	0.96	0.96
Guatemala	WOMEN FIRST	181	166	0.73 (0.23, 2.32)	0.02	0.02
**I² = 0.00, Tau² = 0.00**		**1638**	**3286**			
**Fixed**				**0.88 (0.76, 1.01)**		
**Random**				**0.88 (0.76, 1.01)**		

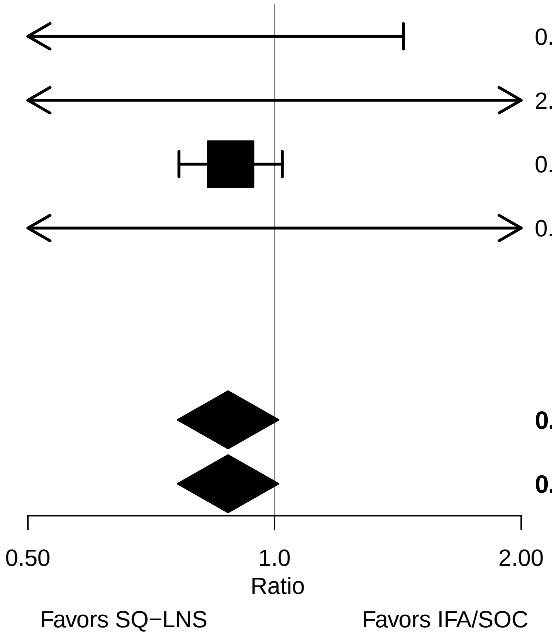

Supplemental figure 2AB: Low HCGAZ risk difference

Country	Trial	SQ−LNS N	IFA/SOC N	RD (95% CI)	Fixed W	Random W
Ghana	DYAD−G	307	305	−0.02 (−0.04, 0.01)	0.24	0.25
Malawi	DYAD−M	253	264	0.01 (−0.01, 0.03)	0.41	0.34
Bangladesh	RDNS	897	2551	−0.02 (−0.05, 0.00)	0.23	0.24
Guatemala	WOMEN FIRST	181	166	−0.01 (−0.04, 0.03)	0.13	0.17
**I² = 0.32, Tau² = 0.00**		**1638**	**3286**			
**Fixed**				**−0.01 (−0.02, 0.01)**		
**Random**				**−0.01 (−0.02, 0.01)**		

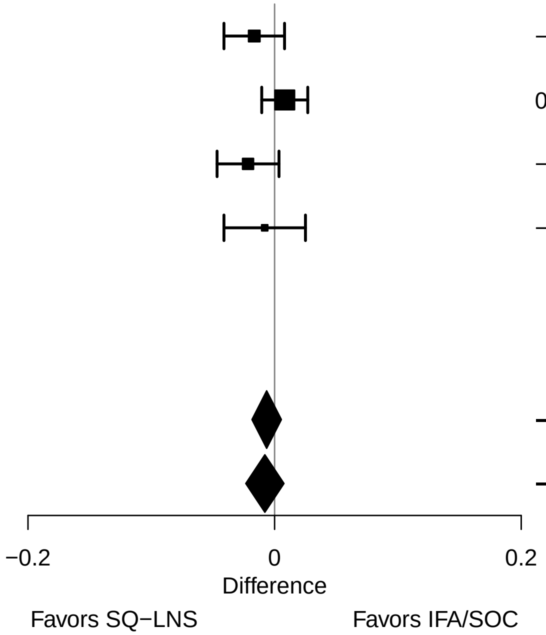

Supplemental figure 2AC: Mean difference in birth mid-upper arm circumference (cm)

Country	Trial	SQ−LNS N	IFA/SOC N	MD (95% CI)	Fixed W	Random W
Ghana	DYAD−G	307	305	0.16 (0.01, 0.31)	0.16	0.16
Malawi	DYAD−M	256	264	0.11 (−0.05, 0.27)	0.14	0.14
Bangladesh	RDNS	898	2551	0.06 (−0.02, 0.13)	0.69	0.69
Guatemala	WOMEN FIRST					
**I² = 0.00, Tau² = 0.00**		**1461**	**3120**			
**Fixed**				**0.08 (0.02, 0.14)**		
**Random**				**0.08 (0.02, 0.14)**		

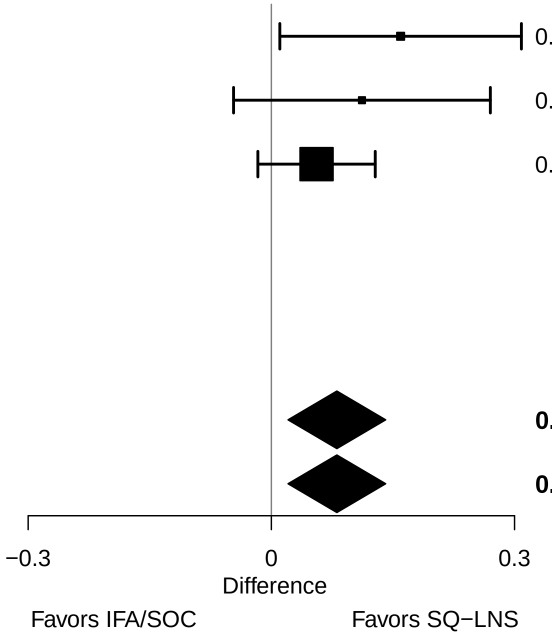

Supplemental figure 2AD: Mean difference in duration of gestation (wk)

Country	Trial	SQ−LNS N	IFA/SOC N	MD (95% CI)	Fixed W	Random W
Ghana	DYAD−G	331	328	0.14 (−0.16, 0.44)	0.15	0.15
Malawi	DYAD−M	436	437	0.19 (−0.21, 0.59)	0.09	0.09
Bangladesh	RDNS	898	2551	0.13 (−0.02, 0.29)	0.60	0.60
Guatemala	WOMEN FIRST	190	177	0.03 (−0.27, 0.33)	0.16	0.16
**I² = 0.00, Tau² = 0.00**		**1855**	**3493**			
**Fixed**				**0.12 (0.01, 0.24)**		
**Random**				**0.12 (0.01, 0.24)**		

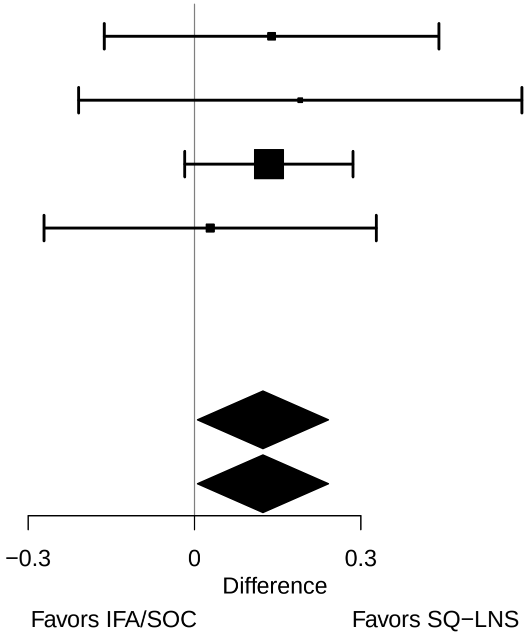

Supplemental figure 2AE: Preterm birth relative risk

Country	Trial	SQ−LNS N	IFA/SOC N	RR (95% CI)	Fixed W	Random W
Ghana	DYAD−G	331	328	0.99 (0.62, 1.58)	0.12	0.12
Malawi	DYAD−M	436	437	0.85 (0.58, 1.24)	0.18	0.18
Bangladesh	RDNS	898	2551	0.95 (0.78, 1.16)	0.65	0.65
Guatemala	WOMEN FIRST	190	177	1.00 (0.51, 1.94)	0.06	0.06
**I² = 0.00, Tau² = 0.00**		**1855**	**3493**			
**Fixed**				**0.94 (0.80, 1.10)**		
**Random**				**0.94 (0.80, 1.10)**		

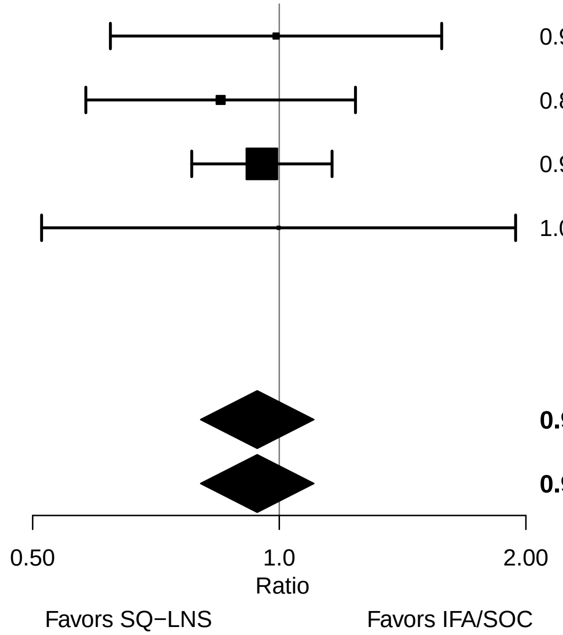

Supplemental figure 2AF: Preterm birth risk difference

Country	Trial	SQ−LNS N	IFA/SOC N	RD (95% CI)	Fixed W	Random W
Ghana	DYAD−G	331	328	0.00 (−0.05, 0.04)	0.17	0.17
Malawi	DYAD−M	436	437	−0.02 (−0.06, 0.02)	0.20	0.20
Bangladesh	RDNS	898	2551	−0.01 (−0.03, 0.02)	0.50	0.50
Guatemala	WOMEN FIRST	190	177	0.00 (−0.05, 0.05)	0.13	0.13
**I² = 0.00, Tau² = 0.00**		**1855**	**3493**			
**Fixed**				**−0.01 (−0.03, 0.01)**		
**Random**				**−0.01 (−0.03, 0.01)**		

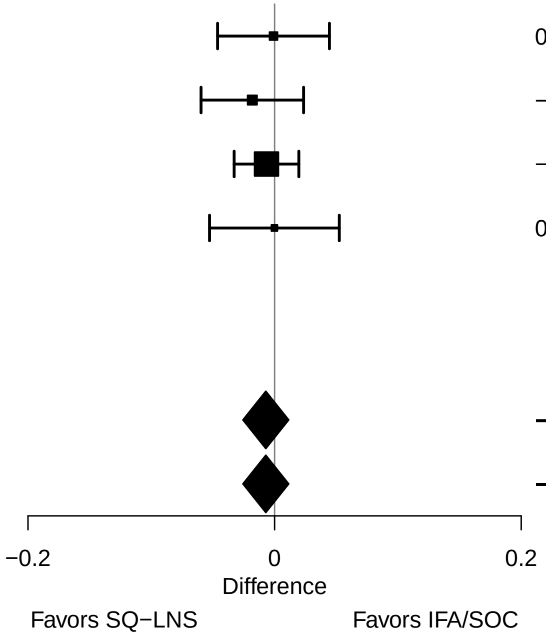

Supplemental figure 2AG: Mean difference in 6 mo weight-for-age z-score

Country	Trial	SQ−LNS N	IFA/SOC N	MD (95% CI)	Fixed W	Random W
Ghana	DYAD−G	281	272	0.07 (−0.11, 0.26)	0.16	0.16
Malawi	DYAD−M	350	364	0.00 (−0.16, 0.17)	0.21	0.21
Bangladesh	RDNS	866	2463	0.01 (−0.10, 0.13)	0.43	0.43
Guatemala	WOMEN FIRST	219	214	0.17 (0.00, 0.33)	0.21	0.21
**I² = 0.00, Tau² = 0.00**		**1716**	**3313**			
**Fixed**				**0.05 (−0.02, 0.13)**		
**Random**				**0.05 (−0.02, 0.13)**		

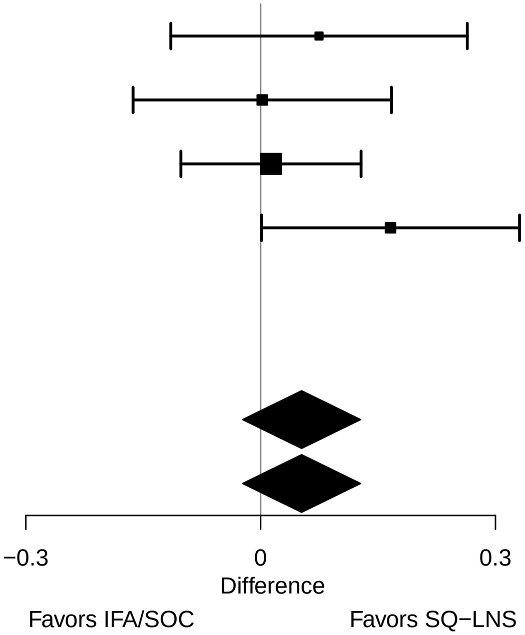

Supplemental figure 2AH: 6 mo underweight prevalence ratio

Country	Trial	SQ−LNS N	IFA/SOC N	PR (95% CI)	Fixed W	Random W
Ghana	DYAD−G	281	272	0.85 (0.48, 1.48)	0.07	0.11
Malawi	DYAD−M	350	364	1.04 (0.66, 1.65)	0.11	0.15
Bangladesh	RDNS	866	2463	0.96 (0.77, 1.20)	0.48	0.41
Guatemala	WOMEN FIRST	219	214	0.67 (0.52, 0.87)	0.33	0.33
**I² = 0.41, Tau² = 0.01**		**1716**	**3313**			
**Fixed**				**0.85 (0.73, 0.99)**		
**Random**				**0.85 (0.70, 1.04)**		

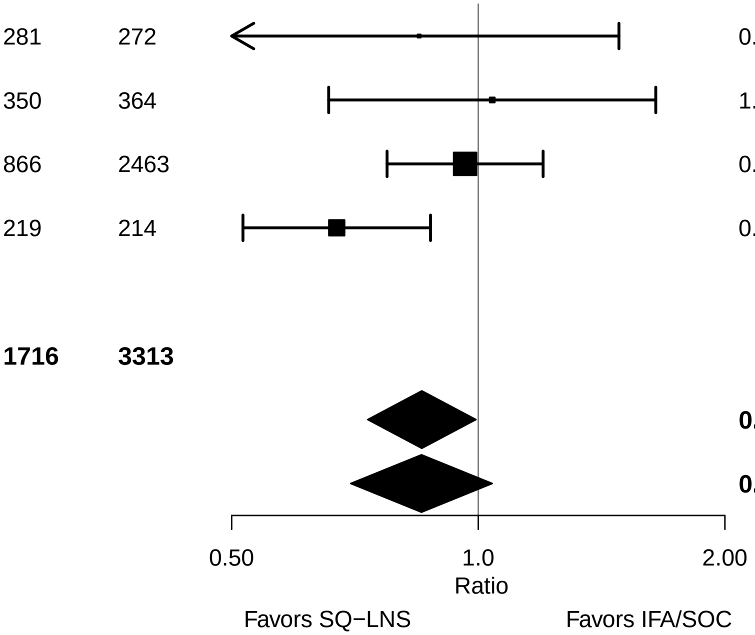

Supplemental figure 2AI: 6 mo underweight prevalence difference

Country	Trial	SQ−LNS N	IFA/SOC N	RD (95% CI)	Fixed W	Random W
Ghana	DYAD−G	281	272	−0.01 (−0.06, 0.03)	0.18	0.20
Malawi	DYAD−M	350	364	0.00 (−0.04, 0.05)	0.21	0.23
Bangladesh	RDNS	866	2463	−0.01 (−0.05, 0.03)	0.22	0.23
Guatemala	WOMEN FIRST	219	214	−0.05 (−0.08, −0.02)	0.38	0.34
**I² = 0.37, Tau² = 0.00**		**1716**	**3313**			
**Fixed**				**−0.02 (−0.04, 0.00)**		
**Random**				**−0.02 (−0.04, 0.00)**		

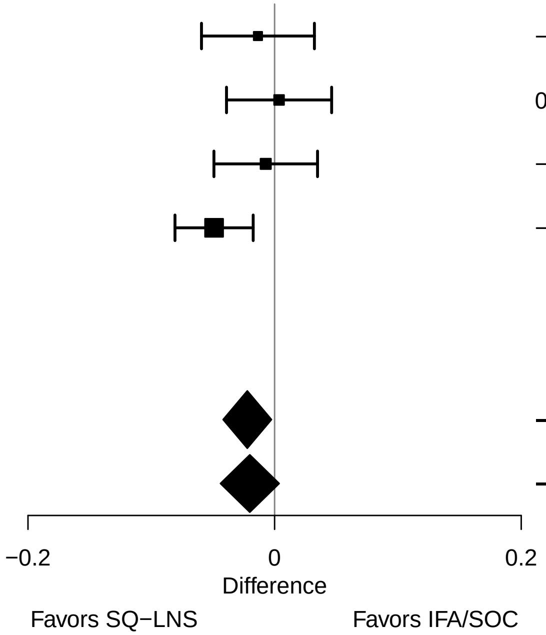

Supplemental figure 2AJ: Mean difference in 6 mo length-for-age z-score

Country	Trial	SQ−LNS N	IFA/SOC N	MD (95% CI)	Fixed W	Random W
Ghana	DYAD−G	282	274	0.08 (−0.09, 0.25)	0.16	0.17
Malawi	DYAD−M	350	365	0.02 (−0.15, 0.18)	0.17	0.18
Bangladesh	RDNS	866	2462	0.03 (−0.06, 0.13)	0.53	0.52
Guatemala	WOMEN FIRST	219	214	0.20 (0.02, 0.39)	0.14	0.14
**I² = 0.02, Tau² = 0.00**		**1717**	**3315**			
**Fixed**				**0.06 (−0.01, 0.13)**		
**Random**				**0.06 (−0.01, 0.13)**		

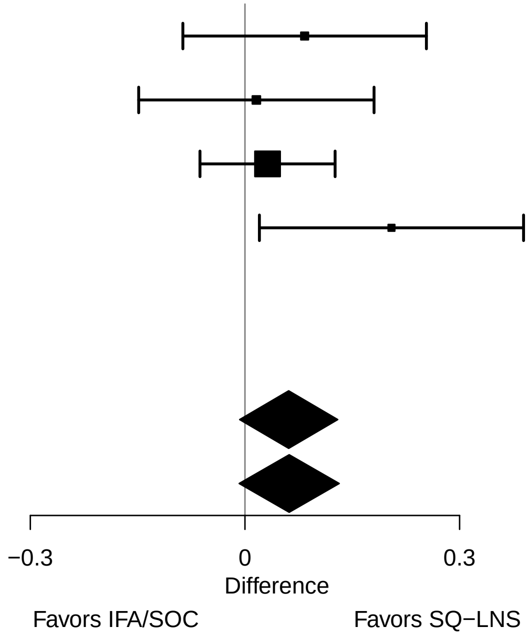

Supplemental figure 2AK: 6 mo stunting prevalence ratio

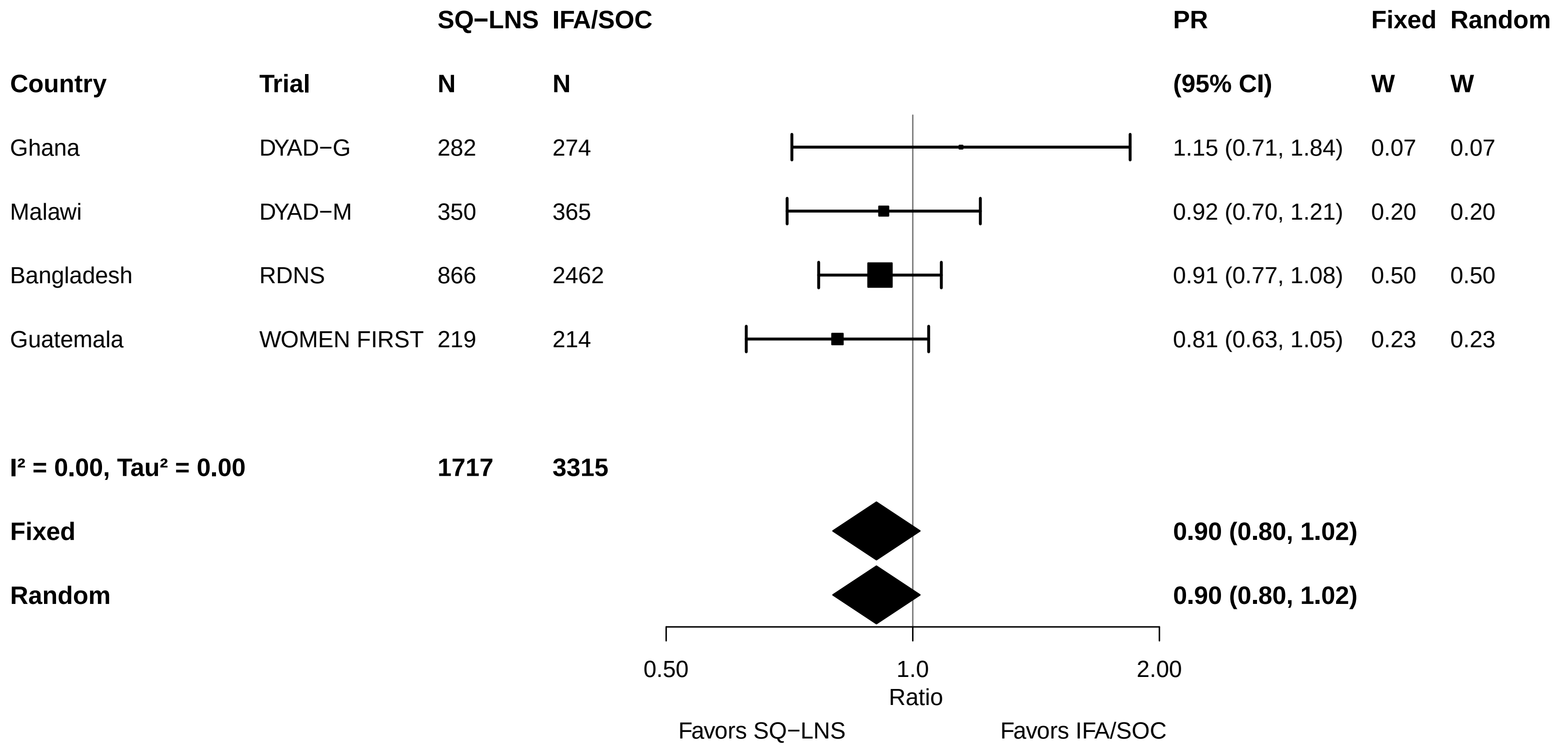

Supplemental figure 2AL: 6 mo stunting prevalence difference

Country	Trial	SQ−LNS N	IFA/SOC N	RD (95% CI)	Fixed W	Random W
Ghana	DYAD−G	282	274	0.01 (−0.04, 0.07)	0.25	0.25
Malawi	DYAD−M	350	365	−0.02 (−0.08, 0.04)	0.18	0.18
Bangladesh	RDNS	866	2462	−0.02 (−0.06, 0.02)	0.43	0.43
Guatemala	WOMEN FIRST	219	214	−0.06 (−0.12, 0.01)	0.15	0.15
**I² = 0.00, Tau² = 0.00**		**1717**	**3315**			
**Fixed**				**−0.02 (−0.04, 0.01)**		
**Random**				**−0.02 (−0.04, 0.01)**		

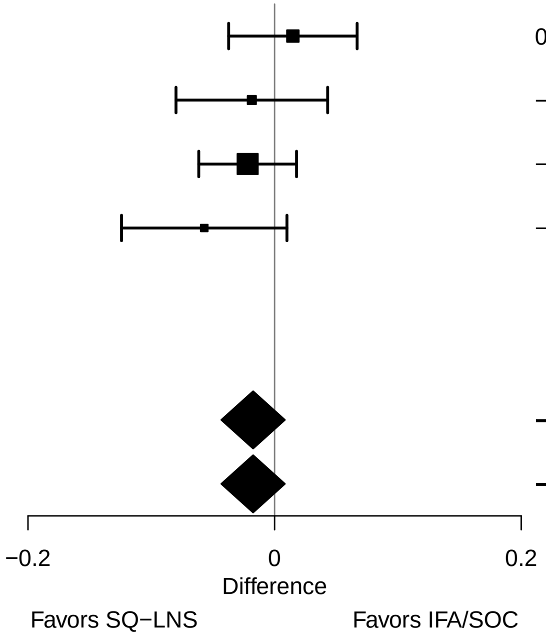

Supplemental figure 2AM: Mean difference in 6 mo weight-for-length z-score

Country	Trial	SQ−LNS N	IFA/SOC N	MD (95% CI)	Fixed W	Random W
Ghana	DYAD−G	281	272	0.03 (−0.16, 0.22)	0.15	0.15
Malawi	DYAD−M	350	364	−0.03 (−0.20, 0.14)	0.18	0.18
Bangladesh	RDNS	866	2462	−0.02 (−0.12, 0.09)	0.48	0.48
Guatemala	WOMEN FIRST	219	214	0.05 (−0.12, 0.22)	0.18	0.18
**I² = 0.00, Tau² = 0.00**		**1716**	**3312**			
**Fixed**				**0.00 (−0.07, 0.07)**		
**Random**				**0.00 (−0.07, 0.07)**		

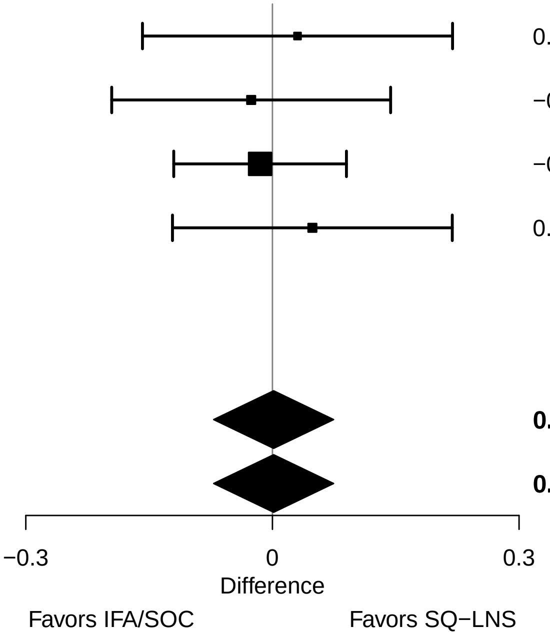

Supplemental figure 2AN: 6 mo wasting prevalence ratio

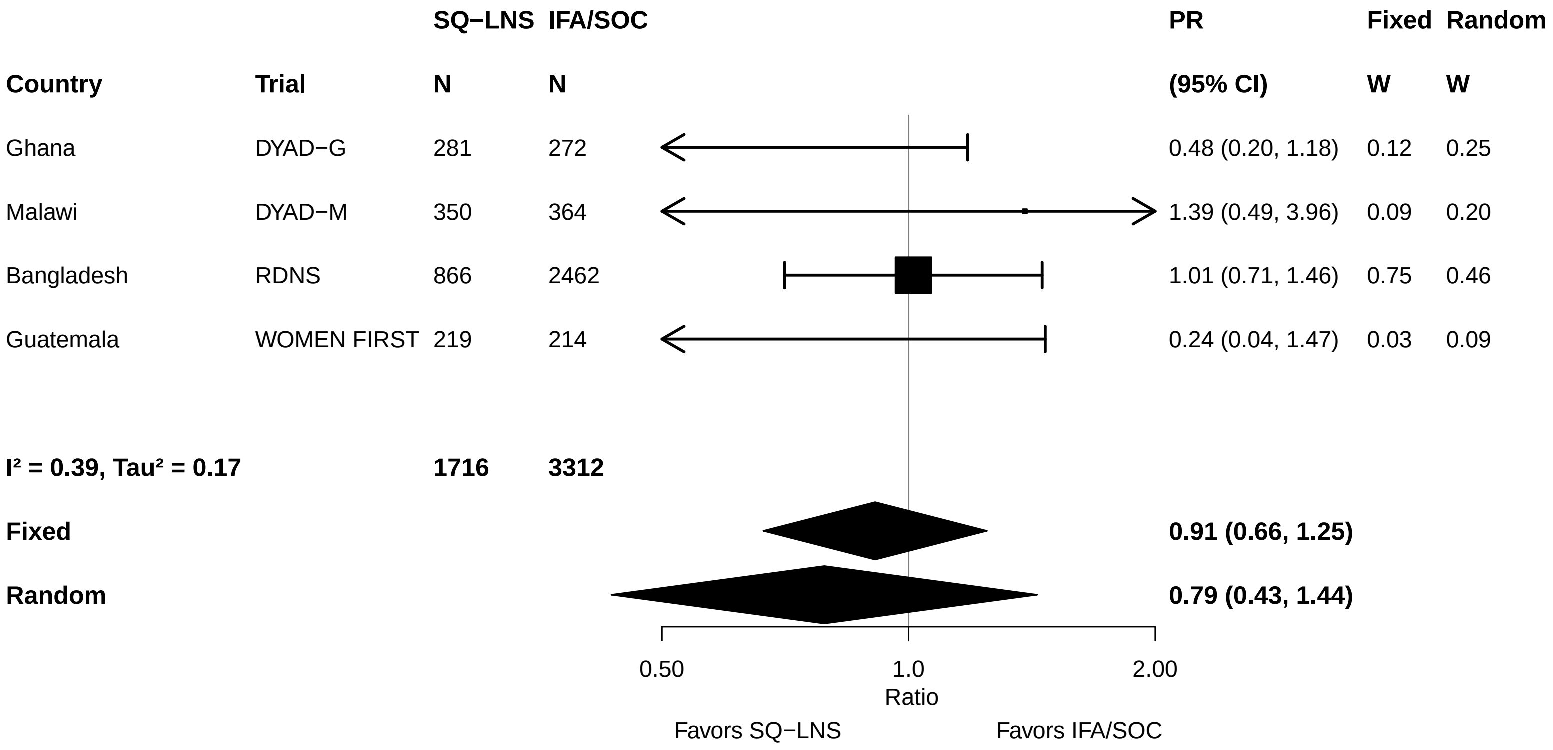

Supplemental figure 2AO: 6 mo wasting prevalence difference

Country	Trial	SQ−LNS N	IFA/SOC N	RD (95% CI)	Fixed W	Random W
Ghana	DYAD−G	281	272	−0.03 (−0.06, 0.01)	0.09	0.13
Malawi	DYAD−M	350	364	0.01 (−0.01, 0.03)	0.21	0.24
Bangladesh	RDNS	866	2462	0.00 (−0.02, 0.02)	0.31	0.30
Guatemala	WOMEN FIRST	219	214	−0.01 (−0.03, 0.00)	0.39	0.34
**I² = 0.36, Tau² = 0.00**		**1716**	**3312**			
**Fixed**				**−0.01 (−0.02, 0.00)**		
**Random**				**−0.01 (−0.02, 0.01)**		

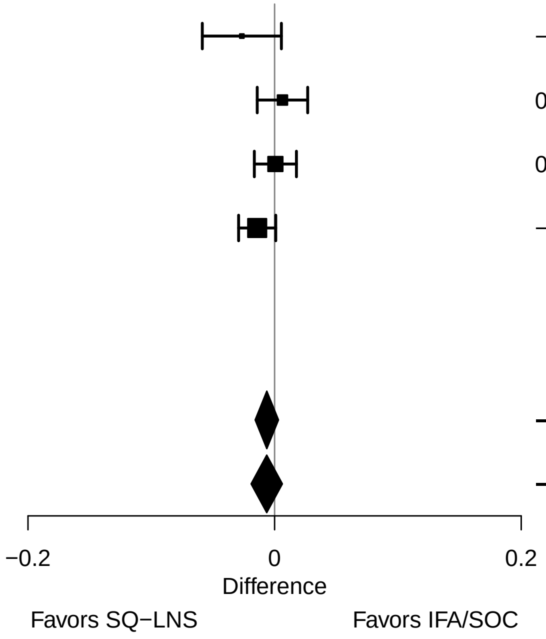

Supplemental figure 2AP: Mean difference in 6 mo head circumference-for-age z-score

Country	Trial	SQ−LNS N	IFA/SOC N	MD (95% CI)	Fixed W	Random W
Ghana	DYAD−G	282	274	0.07 (−0.09, 0.22)	0.16	0.16
Malawi	DYAD−M	350	363	−0.05 (−0.20, 0.10)	0.16	0.16
Bangladesh	RDNS	866	2463	0.01 (−0.07, 0.09)	0.57	0.57
Guatemala	WOMEN FIRST	219	211	0.06 (−0.12, 0.24)	0.11	0.11
**I² = 0.00, Tau² = 0.00**		**1717**	**3311**			
**Fixed**				**0.01 (−0.05, 0.07)**		
**Random**				**0.01 (−0.05, 0.07)**		

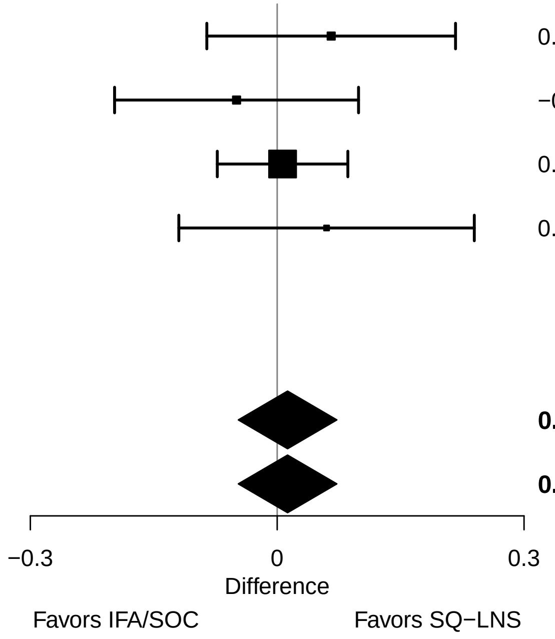

Supplemental figure 2AQ: 6 mo low HCZ prevalence ratio

Country	Trial	SQ−LNS N	IFA/SOC N	PR (95% CI)	Fixed W	Random W
Ghana	DYAD−G	282	274	0.80 (0.45, 1.44)	0.04	0.04
Malawi	DYAD−M	350	363	1.15 (0.63, 2.09)	0.04	0.04
Bangladesh	RDNS	866	2463	0.91 (0.80, 1.04)	0.86	0.86
Guatemala	WOMEN FIRST	219	211	0.75 (0.45, 1.26)	0.06	0.06
**I² = 0.00, Tau² = 0.00**		**1717**	**3311**			
**Fixed**				**0.90 (0.80, 1.02)**		
**Random**				**0.90 (0.80, 1.02)**		

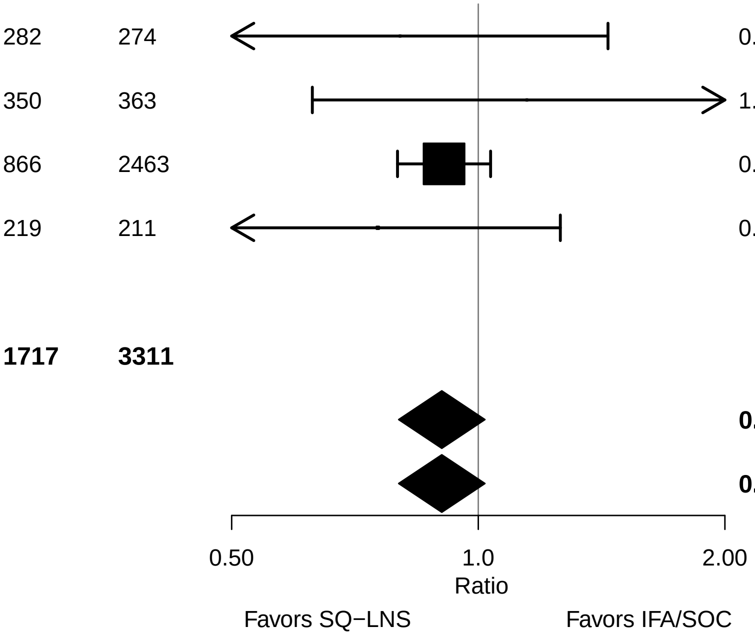

Supplemental figure 2AR: 6 mo low HCZ prevalence difference

Country	Trial	SQ−LNS N	IFA/SOC N	RD (95% CI)	Fixed W	Random W
Ghana	DYAD−G	282	274	−0.02 (−0.06, 0.03)	0.18	0.18
Malawi	DYAD−M	350	363	0.01 (−0.03, 0.04)	0.30	0.30
Bangladesh	RDNS	866	2463	−0.02 (−0.05, 0.01)	0.36	0.36
Guatemala	WOMEN FIRST	219	211	−0.03 (−0.07, 0.02)	0.16	0.16
**I² = 0.00, Tau² = 0.00**		**1717**	**3311**			
**Fixed**				**−0.01 (−0.03, 0.01)**		
**Random**				**−0.01 (−0.03, 0.01)**		

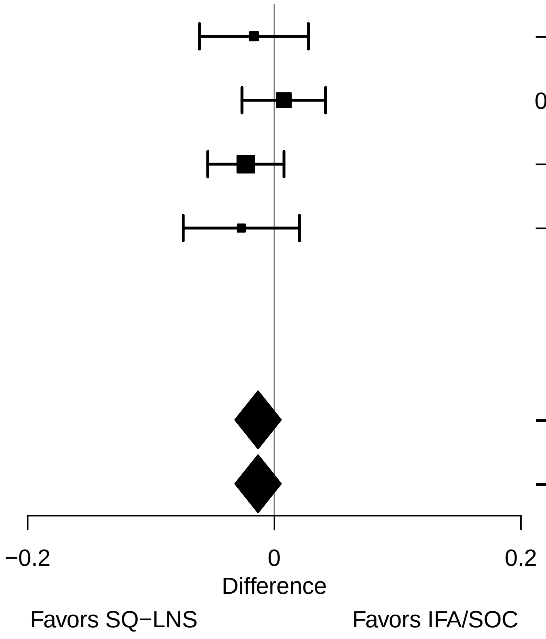

Supplemental figure 2AS: Mean difference in 6 mo MUAC-for-age z-score

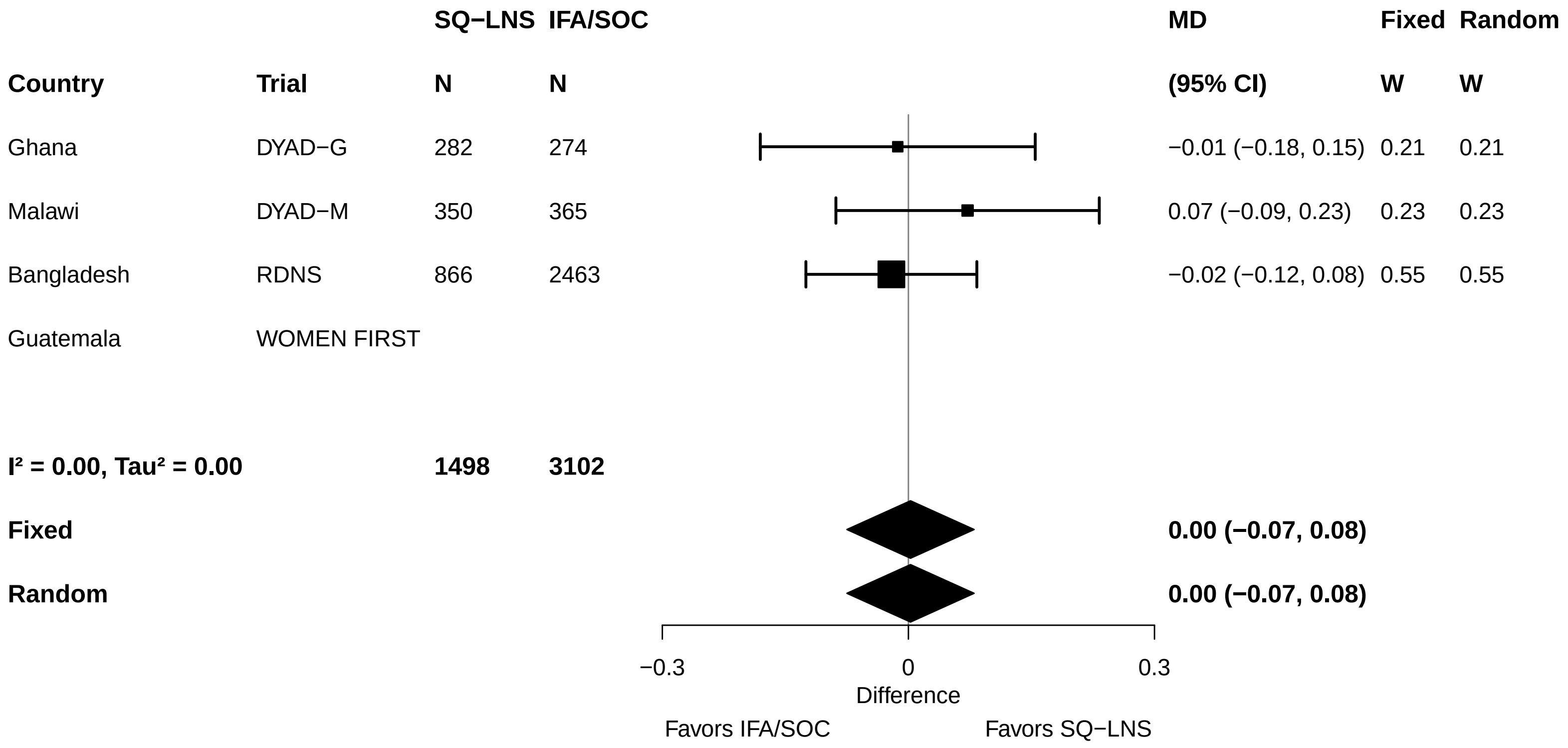

Supplemental figure 2AT: 6 mo low MUAC prevalence ratio

Country	Trial	SQ−LNS N	IFA/SOC N	PR (95% CI)	Fixed W	Random W
Ghana	DYAD−G	282	274	0.97 (0.56, 1.69)	0.21	0.21
Malawi	DYAD−M	350	365	0.74 (0.43, 1.30)	0.21	0.21
Bangladesh	RDNS	866	2463	1.13 (0.81, 1.58)	0.58	0.58
Guatemala	WOMEN FIRST					
**I² = 0.00, Tau² = 0.00**		**1498**	**3102**			
**Fixed**				**1.00 (0.78, 1.29)**		
**Random**				**1.00 (0.78, 1.29)**		

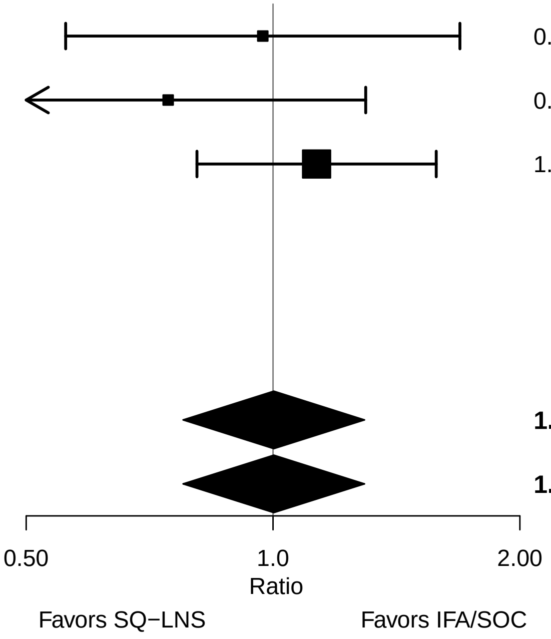

Supplemental figure 2AU: 6 mo low MUAC prevalence difference

Country	Trial	SQ−LNS N	IFA/SOC N	RD (95% CI)	Fixed W	Random W
Ghana	DYAD−G	282	274	0.00 (−0.05, 0.04)	0.15	0.15
Malawi	DYAD−M	350	365	−0.02 (−0.06, 0.02)	0.24	0.24
Bangladesh	RDNS	866	2463	0.01 (−0.01, 0.03)	0.61	0.61
Guatemala	WOMEN FIRST					
**I² = 0.00, Tau² = 0.00**		**1498**	**3102**			
**Fixed**				**0.00 (−0.02, 0.02)**		
**Random**				**0.00 (−0.02, 0.02)**		

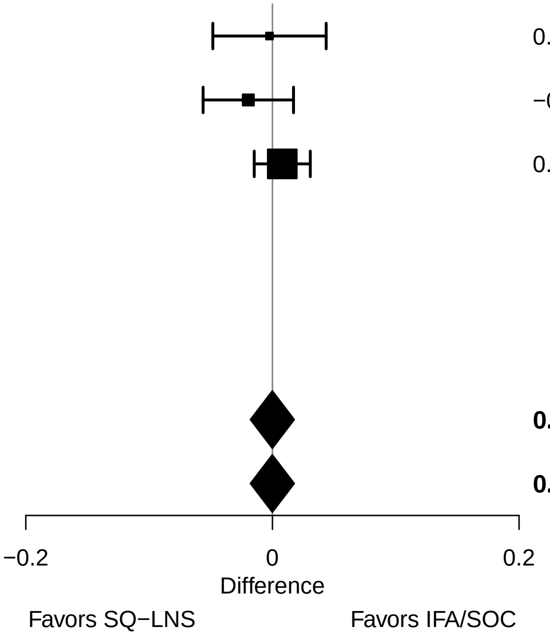

Supplemental figure 2AV: 6 mo acute malnutrition prevalence ratio

Country	Trial	SQ−LNS N	IFA/SOC N	PR (95% CI)	Fixed W	Random W
Ghana	DYAD−G	281	272	1.01 (0.58, 1.75)	0.17	0.17
Malawi	DYAD−M	350	364	0.83 (0.50, 1.39)	0.19	0.19
Bangladesh	RDNS	866	2462	1.05 (0.79, 1.39)	0.64	0.64
Guatemala	WOMEN FIRST					
**I² = 0.00, Tau² = 0.00**		**1497**	**3098**			
**Fixed**				**1.00 (0.79, 1.25)**		
**Random**				**1.00 (0.79, 1.25)**		

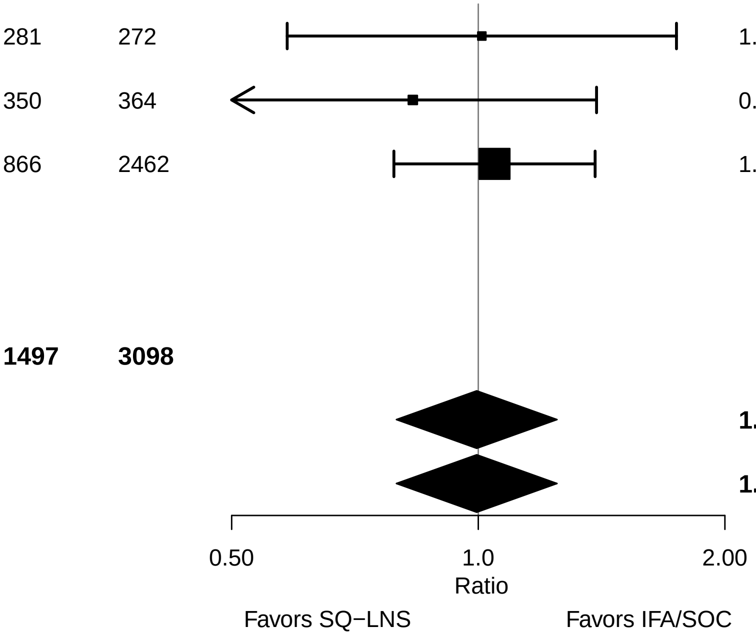

Supplemental figure 2AW: 6 mo acute malnutrition prevalence difference

Country	Trial	SQ−LNS N	IFA/SOC N	RD (95% CI)	Fixed W	Random W
Ghana	DYAD−G	281	272	0.00 (−0.05, 0.05)	0.16	0.16
Malawi	DYAD−M	350	364	−0.01 (−0.05, 0.02)	0.23	0.23
Bangladesh	RDNS	866	2462	0.00 (−0.02, 0.03)	0.61	0.61
Guatemala	WOMEN FIRST					
**I² = 0.00, Tau² = 0.00**		**1497**	**3098**			
**Fixed**				**0.00 (−0.02, 0.02)**		
**Random**				**0.00 (−0.02, 0.02)**		

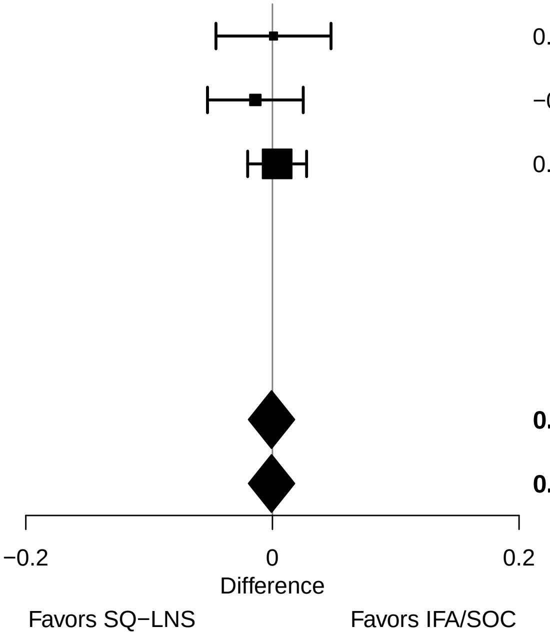

Supplemental figure 2AX: Cesarian-section relative risk

Country	Trial	SQ−LNS N	IFA/SOC N	RR (95% CI)	Fixed W	Random W
Ghana	DYAD−G	329	327	0.98 (0.71, 1.35)	0.18	0.25
Malawi	DYAD−M	421	405	1.92 (1.09, 3.39)	0.06	0.14
Bangladesh	RDNS	983	2763	1.08 (0.79, 1.48)	0.19	0.26
Guatemala	WOMEN FIRST	235	230	0.98 (0.82, 1.17)	0.57	0.35
**I² = 0.42, Tau² = 0.04**		**1968**	**3725**			
**Fixed**				**1.04 (0.90, 1.19)**		
**Random**				**1.10 (0.85, 1.43)**		

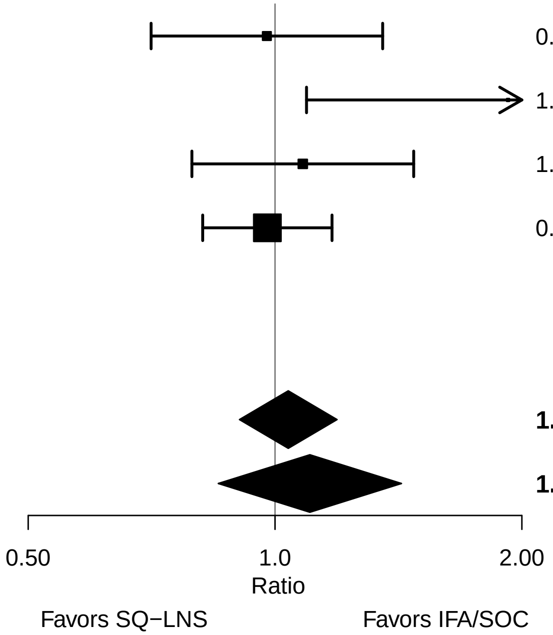

Supplemental figure 2AY: Cesarian-section risk difference

Country	Trial	SQ−LNS N	IFA/SOC N	RD (95% CI)	Fixed W	Random W
Ghana	DYAD−G	329	327	0.00 (−0.06, 0.06)	0.14	0.14
Malawi	DYAD−M	421	405	0.04 (0.01, 0.07)	0.47	0.47
Bangladesh	RDNS	983	2763	0.01 (−0.04, 0.06)	0.19	0.19
Guatemala	WOMEN FIRST	235	230	−0.01 (−0.05, 0.04)	0.21	0.21
**I² = 0.01, Tau² = 0.00**		**1968**	**3725**			
**Fixed**				**0.02 (0.00, 0.04)**		
**Random**				**0.02 (0.00, 0.04)**		

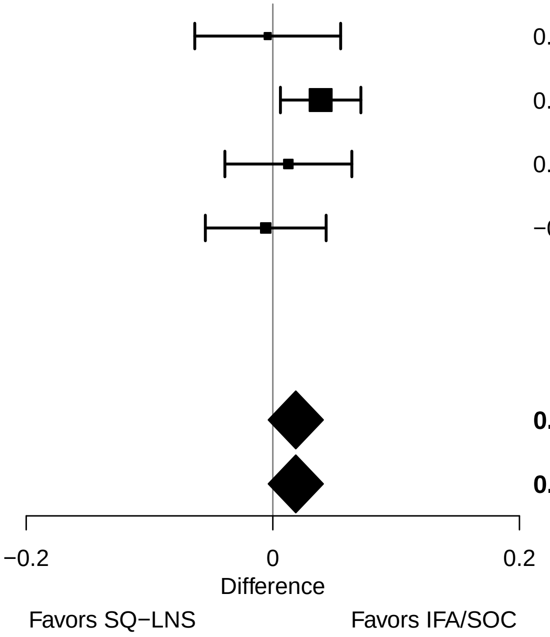

Supplemental figure 2AZ: Miscarriage relative risk

Country	Trial	SQ−LNS N	IFA/SOC N	RR (95% CI)	Fixed W	Random W
Ghana	DYAD−G	354	349	0.68 (0.30, 1.58)	0.07	0.07
Malawi	DYAD−M	431	433	4.02 (0.45, 35.81)	0.01	0.01
Bangladesh	RDNS	1044	2954	0.89 (0.69, 1.15)	0.72	0.72
Guatemala	WOMEN FIRST	267	270	0.81 (0.50, 1.31)	0.20	0.20
**I² = 0.00, Tau² = 0.00**		**2096**	**4006**			
**Fixed**				**0.87 (0.70, 1.08)**		
**Random**				**0.87 (0.70, 1.08)**		

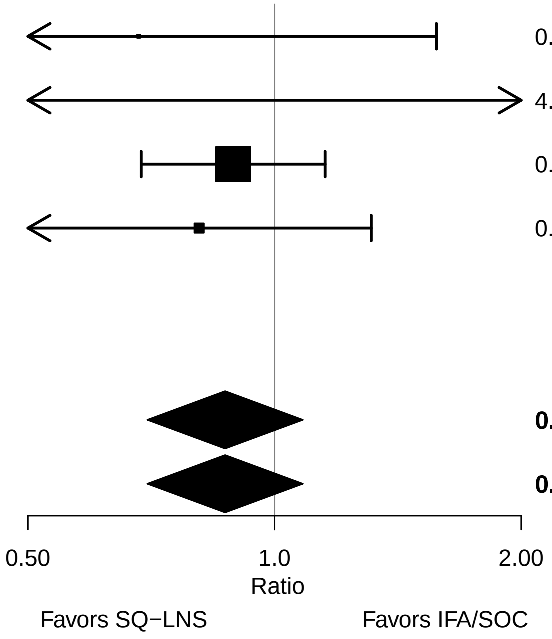

Supplemental figure 2BA: Miscarriage risk difference

Country	Trial	SQ−LNS N	IFA/SOC N	RD (95% CI)	Fixed W	Random W
Ghana	DYAD−G	354	349	−0.01 (−0.04, 0.01)	0.09	0.09
Malawi	DYAD−M	431	433	0.01 (0.00, 0.02)	0.60	0.60
Bangladesh	RDNS	1044	2954	−0.01 (−0.02, 0.01)	0.29	0.29
Guatemala	WOMEN FIRST	267	270	−0.03 (−0.09, 0.04)	0.01	0.01
**I² = 0.27, Tau² = 0.00**		**2096**	**4006**			
**Fixed**				**0.00 (−0.01, 0.01)**		
**Random**				**0.00 (−0.01, 0.01)**		

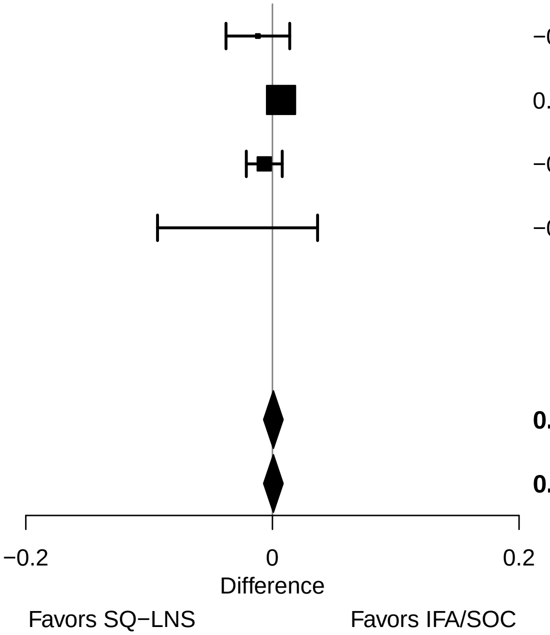

Supplemental figure 2BB: Stillbirth relative risk

Country	Trial	SQ−LNS N	IFA/SOC N	RR (95% CI)	Fixed W	Random W
Ghana	DYAD−G	354	349	0.44 (0.14, 1.41)	0.10	0.23
Malawi	DYAD−M	431	433	1.56 (0.68, 3.57)	0.20	0.29
Bangladesh	RDNS	1044	2954	1.35 (0.85, 2.15)	0.66	0.35
Guatemala	WOMEN FIRST	267	270	6.07 (0.86, 42.74)	0.04	0.13
**I² = 0.49, Tau² = 0.51**		**2096**	**4006**			
**Fixed**				**1.31 (0.90, 1.91)**		
**Random**				**[illegible] ([illegible], 3.18)**		

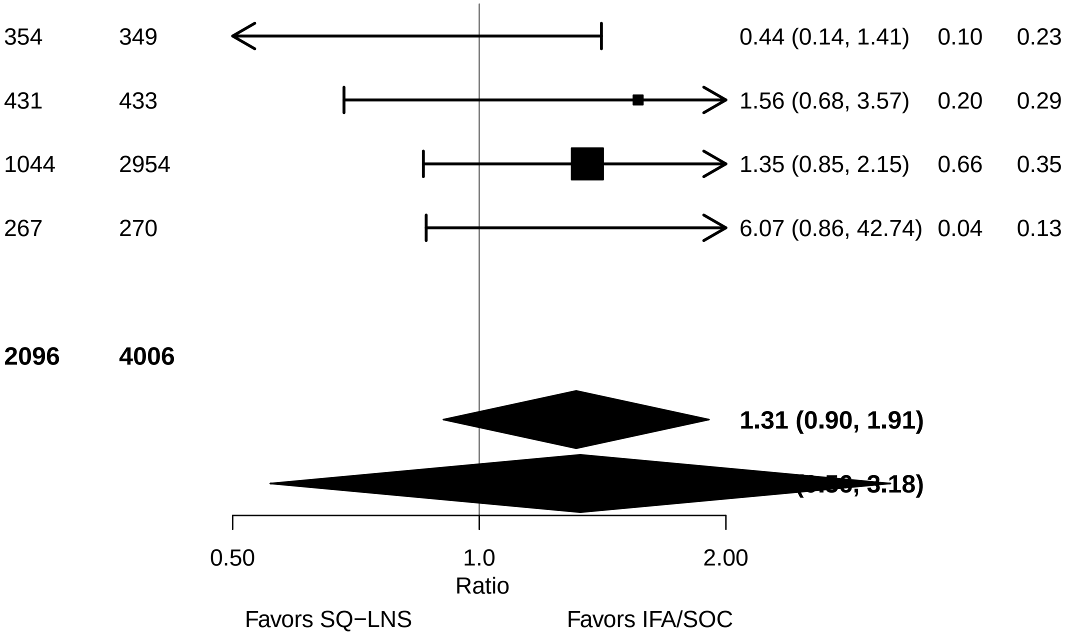

Supplemental figure 2BC: Stillbirth risk difference

Country	Trial	SQ−LNS N	IFA/SOC N	RD (95% CI)	Fixed W	Random W
Ghana	DYAD−G	354	349	−0.01 (−0.03, 0.01)	0.19	0.23
Malawi	DYAD−M	431	433	0.01 (−0.01, 0.03)	0.17	0.21
Bangladesh	RDNS	1044	2954	0.01 (−0.01, 0.02)	0.39	0.31
Guatemala	WOMEN FIRST	267	270	0.02 (0.00, 0.04)	0.24	0.25
**I² = 0.53, Tau² = 0.00**		**2096**	**4006**			
**Fixed**				**0.01 (0.00, 0.02)**		
**Random**				**0.01 (−0.01, 0.02)**		

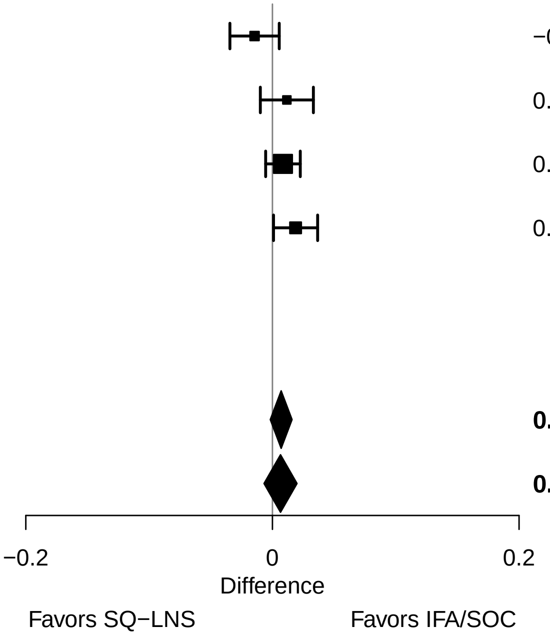

Supplemental figure 2BD: Miscarriage or stillbirth relative risk

Country	Trial	SQ−LNS N	IFA/SOC N	RR (95% CI)	Fixed W	Random W
Ghana	DYAD−G	354	349	0.58 (0.30, 1.14)	0.08	0.18
Malawi	DYAD−M	431	433	1.81 (0.84, 3.87)	0.06	0.15
Bangladesh	RDNS	1044	2954	1.01 (0.79, 1.29)	0.61	0.37
Guatemala	WOMEN FIRST	267	270	0.94 (0.64, 1.38)	0.25	0.30
**I² = 0.39, Tau² = 0.08**		**2096**	**4006**			
**Fixed**				**0.98 (0.81, 1.19)**		
**Random**				**0.98 (0.68, 1.42)**		

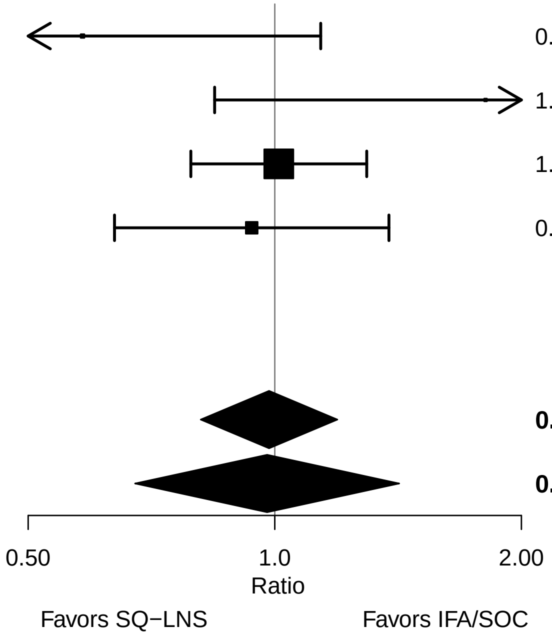

Supplemental figure 2BE: Miscarriage or stillbirth risk difference

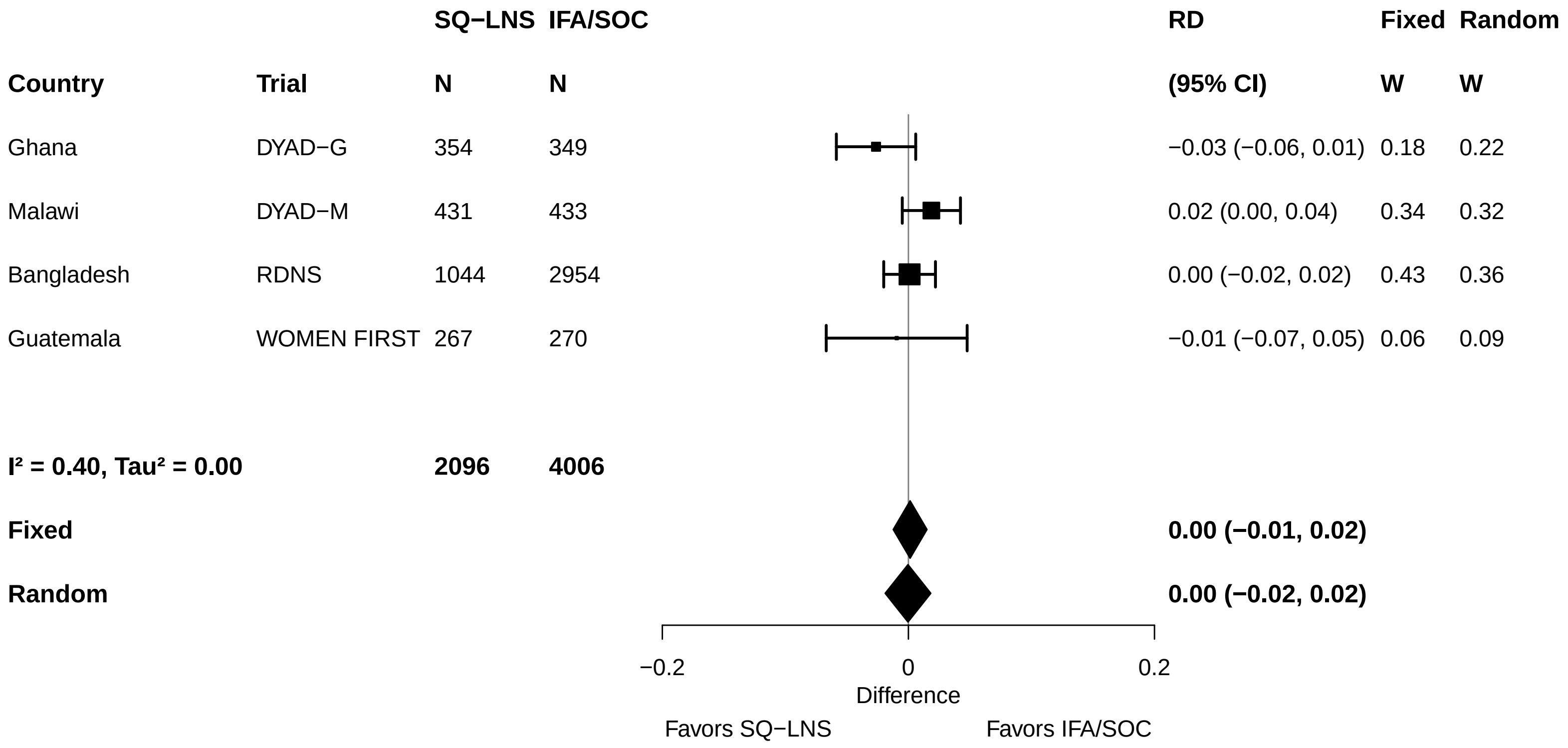

Supplemental figure 2BF: Early neonatal mortality relative risk

Country	Trial	SQ−LNS N	IFA/SOC N	RR (95% CI)	Fixed W	Random W
Ghana	DYAD−G	332	324	1.95 (0.49, 7.74)	0.12	0.13
Malawi	DYAD−M	399	412	0.52 (0.22, 1.19)	0.32	0.33
Bangladesh	RDNS	935	2651	0.79 (0.40, 1.52)	0.51	0.50
Guatemala	WOMEN FIRST	228	225	0.33 (0.03, 3.31)	0.04	0.05
**I² = 0.04, Tau² = 0.02**		**1894**	**3612**			
**Fixed**				**0.74 (0.46, 1.19)**		
**Random**				**0.74 (0.45, 1.22)**		

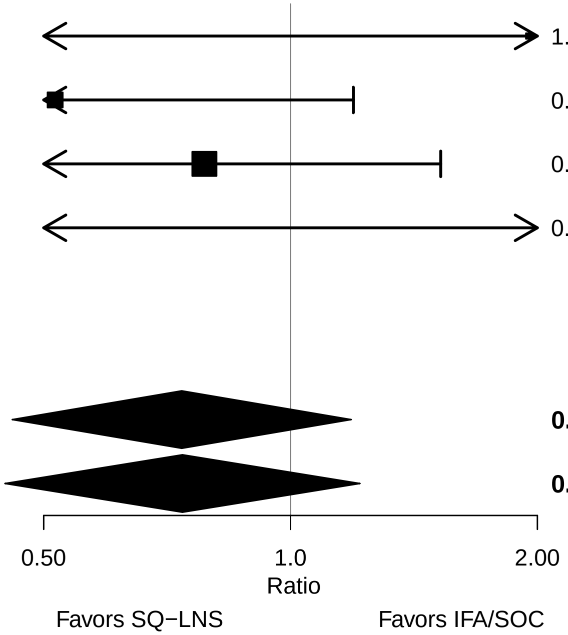

Supplemental figure 2BG: Early neonatal mortality risk difference

Country	Trial	SQ−LNS N	IFA/SOC N	RD (95% CI)	Fixed W	Random W
Ghana	DYAD−G	332	324	0.01 (−0.01, 0.03)	0.23	0.25
Malawi	DYAD−M	399	412	−0.02 (−0.04, 0.00)	0.13	0.18
Bangladesh	RDNS	935	2651	−0.01 (−0.02, 0.01)	0.40	0.33
Guatemala	WOMEN FIRST	228	225	−0.01 (−0.03, 0.01)	0.24	0.25
**I² = 0.22, Tau² = 0.00**		**1894**	**3612**			
**Fixed**				**0.00 (−0.01, 0.00)**		
**Random**				**−0.01 (−0.02, 0.01)**		

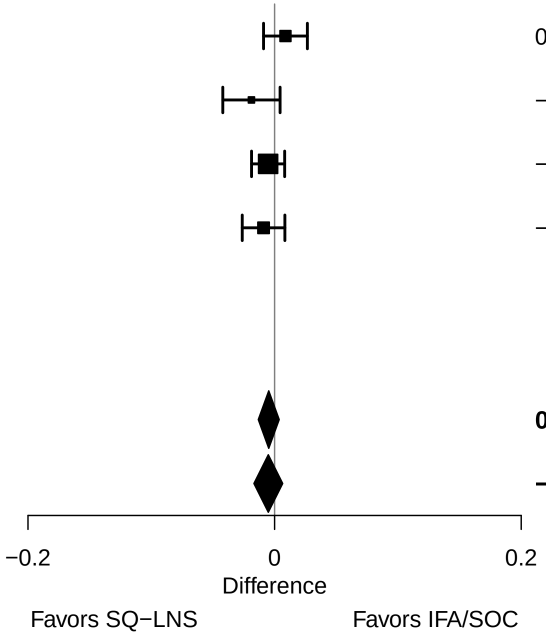

Supplemental figure 2BH: Miscarriage or stillbirth or early neonatal mortality relative risk

Country	Trial	SQ−LNS N	IFA/SOC N	RR (95% CI)	Fixed W	Random W
Ghana	DYAD−G	354	349	0.75 (0.42, 1.34)	0.10	0.10
Malawi	DYAD−M	431	433	1.00 (0.59, 1.70)	0.13	0.13
Bangladesh	RDNS	1044	2954	0.97 (0.75, 1.24)	0.56	0.56
Guatemala	WOMEN FIRST	267	270	0.90 (0.60, 1.34)	0.22	0.22
**I² = 0.00, Tau² = 0.00**		**2096**	**4006**			
**Fixed**				**0.93 (0.77, 1.12)**		
**Random**				**0.93 (0.77, 1.12)**		

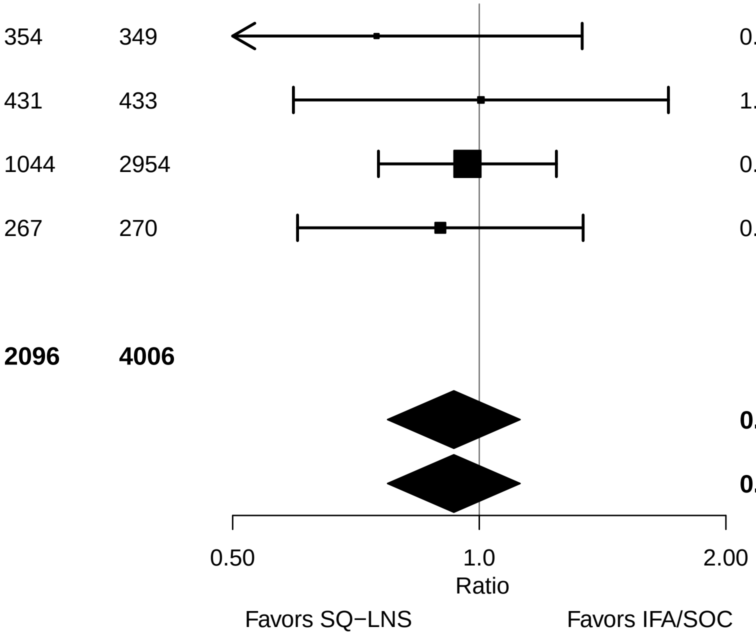

Supplemental figure 2BI: Miscarriage or stillbirth or early neonatal mortality risk difference

Country	Trial	SQ−LNS N	IFA/SOC N	RD (95% CI)	Fixed W	Random W
Ghana	DYAD−G	354	349	−0.02 (−0.05, 0.02)	0.22	0.22
Malawi	DYAD−M	431	433	0.00 (−0.03, 0.03)	0.28	0.28
Bangladesh	RDNS	1044	2954	0.00 (−0.03, 0.02)	0.43	0.43
Guatemala	WOMEN FIRST	267	270	−0.02 (−0.08, 0.05)	0.07	0.07
**I² = 0.00, Tau² = 0.00**		**2096**	**4006**			
**Fixed**				**−0.01 (−0.02, 0.01)**		
**Random**				**−0.01 (−0.02, 0.01)**		

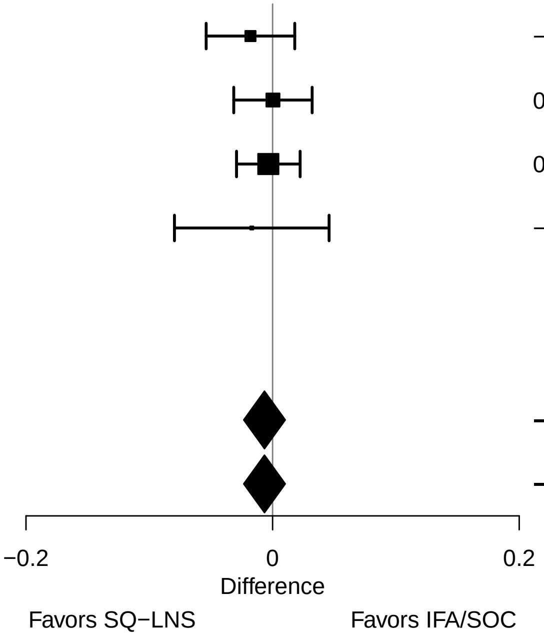

Supplemental figure 2BJ: Neonatal mortality relative risk

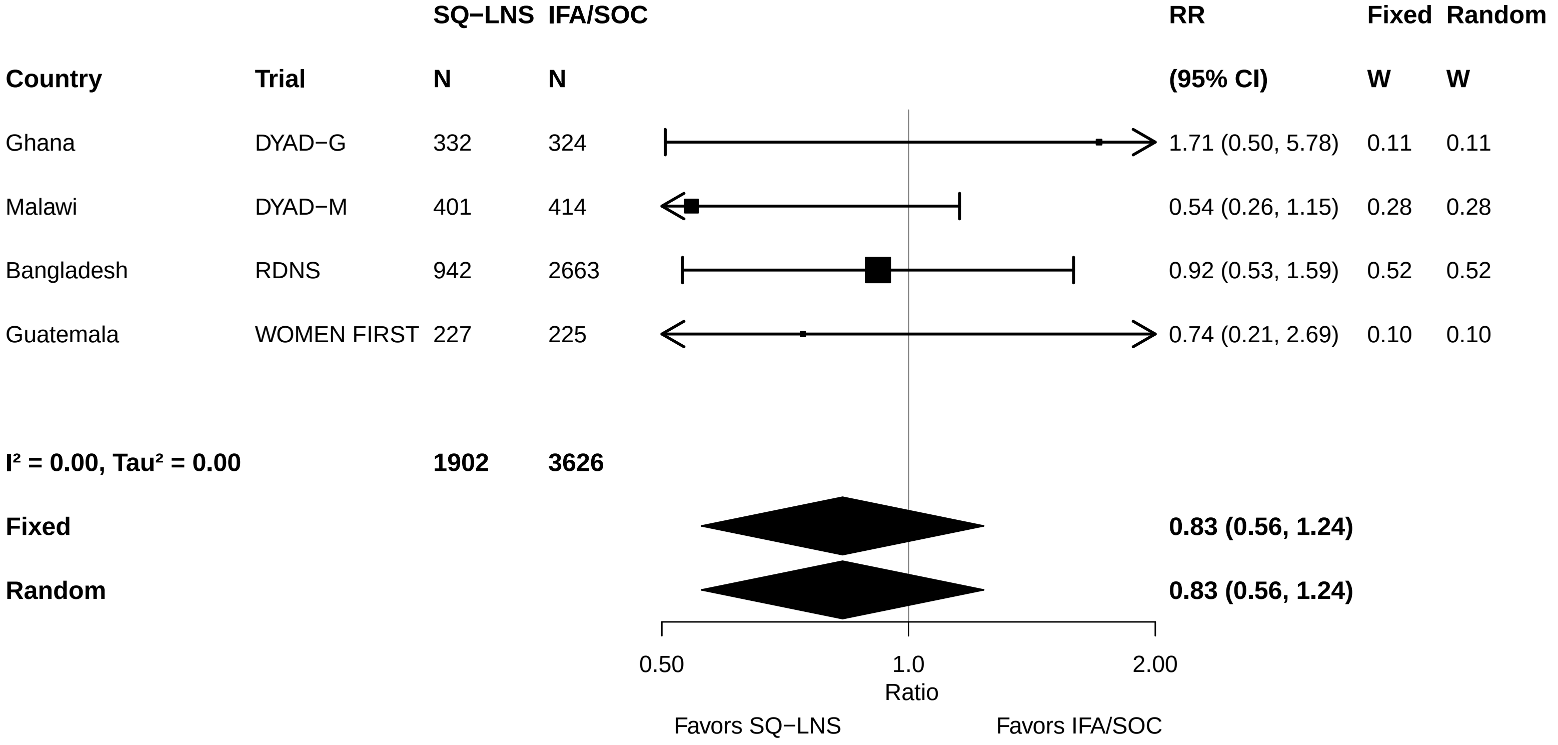

Supplemental figure 2BK: Neonatal mortality risk difference

Country	Trial	SQ−LNS N	IFA/SOC N	RD (95% CI)	Fixed W	Random W
Ghana	DYAD−G	332	324	0.01 (−0.01, 0.03)	0.23	0.23
Malawi	DYAD−M	401	414	−0.02 (−0.05, 0.00)	0.14	0.14
Bangladesh	RDNS	942	2663	0.00 (−0.02, 0.01)	0.41	0.41
Guatemala	WOMEN FIRST	227	225	0.00 (−0.02, 0.02)	0.22	0.22
**I² = 0.10, Tau² = 0.00**		**1902**	**3626**			
**Fixed**				**0.00 (−0.01, 0.01)**		
**Random**				**0.00 (−0.01, 0.01)**		

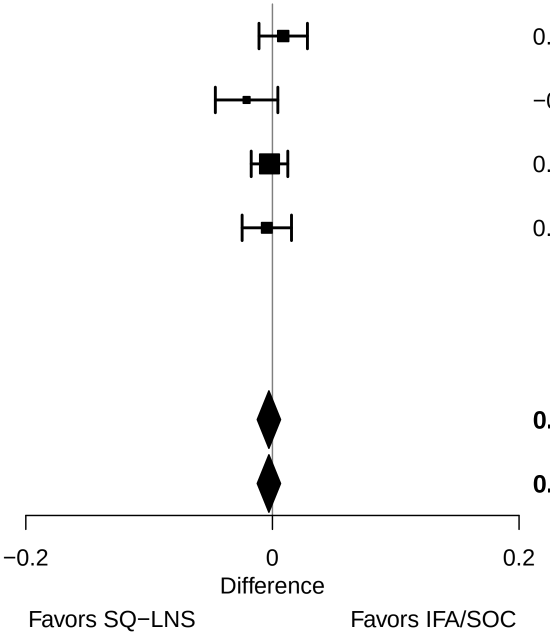

Supplemental figure 2BL: Mortality 0-6 mo relative risk

Country	Trial	SQ−LNS N	IFA/SOC N	RR (95% CI)	Fixed W	Random W
Ghana	DYAD−G	332	324	1.56 (0.52, 4.72)	0.08	0.08
Malawi	DYAD−M	406	416	0.73 (0.38, 1.40)	0.23	0.23
Bangladesh	RDNS	954	2689	1.01 (0.67, 1.53)	0.58	0.58
Guatemala	WOMEN FIRST	225	220	0.81 (0.32, 2.05)	0.11	0.11
**I² = 0.00, Tau² = 0.00**		**1917**	**3649**			
**Fixed**				**0.95 (0.69, 1.30)**		
**Random**				**0.95 (0.69, 1.30)**		

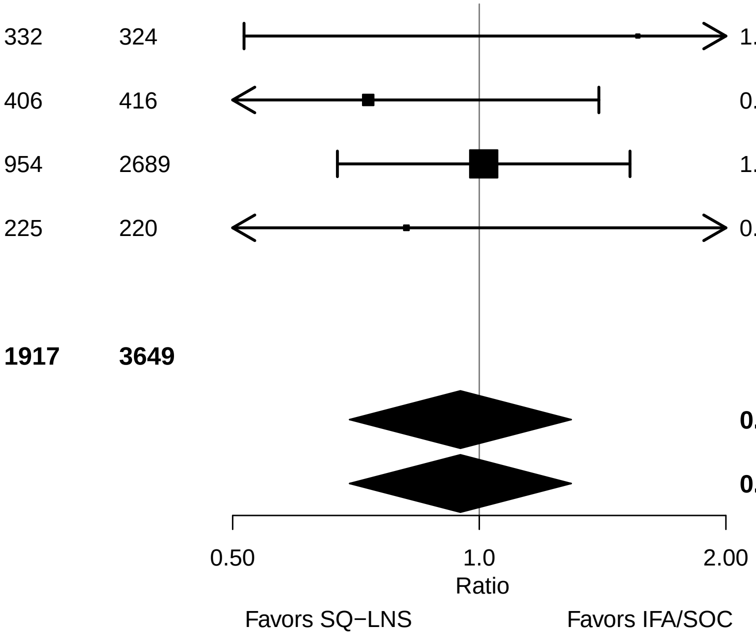

Supplemental figure 2BM: Mortality 0-6 mo risk difference

Country	Trial	SQ−LNS N	IFA/SOC N	RD (95% CI)	Fixed W	Random W
Ghana	DYAD−G	332	324	0.01 (−0.01, 0.03)	0.24	0.24
Malawi	DYAD−M	406	416	−0.01 (−0.04, 0.01)	0.14	0.14
Bangladesh	RDNS	954	2689	0.00 (−0.02, 0.02)	0.43	0.43
Guatemala	WOMEN FIRST	225	220	−0.01 (−0.03, 0.02)	0.19	0.19
**I² = 0.00, Tau² = 0.00**		**1917**	**3649**			
**Fixed**				**0.00 (−0.01, 0.01)**		
**Random**				**0.00 (−0.01, 0.01)**		

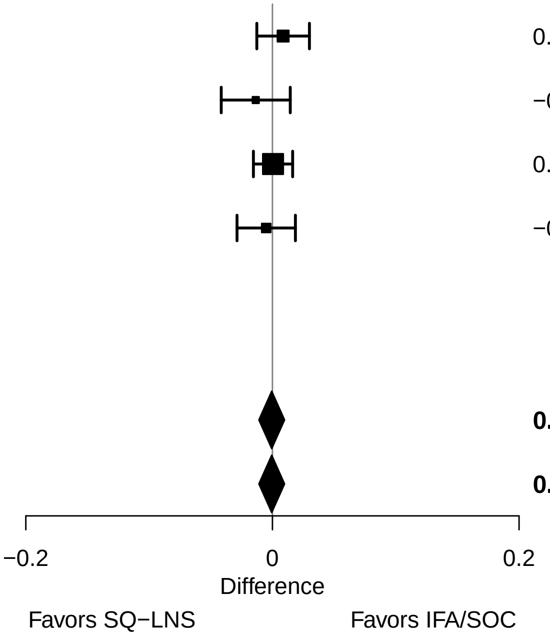

Supplement: Multimedia component 1 [file mmc1.zip › Maternal SQ-LNS Supplemental_2024-09-03/7_Maternal SQ-LNS Supplemental figure 2.pdf]
