# Supplementary material for: Effects of prenatal small-quantity lipid-based nutrient supplements on pregnancy, birth, and infant outcomes: a systematic review and meta-analysis of individual participant data from randomized controlled trials in low- and middle-income countries
Source: Am J Clin Nutr. 2024 Aug 16;120(4):814–35. doi: 10.1016/j.ajcnut.2024.08.008 (PMC11473441; doi:10.1016/j.ajcnut.2024.08.008)

Supplemental figure 3: Pooled plots for infant outcomes at birth and at 6 mo, stratified by potential effect modifiers, SQ-LNS vs IFA/SOC

Contents

Supplemental figure 3A: Sex **3**

3A1: Mean differences for birth outcomes . . . 3

3A2: Relative risks for birth outcomes . . . 4

3A3: Mean differences for 6 mo outcomes . . . 5

3A4: Prevalence ratios for 6 mo outcomes . . . 6

Supplemental figure 3B: Birth order **7**

3B1: Mean differences for birth outcomes . . . 7

3B2: Relative risks for birth outcomes . . . 8

3B3: Mean differences for 6 mo outcomes . . . 9

3B4: Prevalence ratios for 6 mo outcomes . . . 10

Supplemental figure 3C: Maternal height **11**

3C1: Mean differences for birth outcomes . . . 11

3C2: Relative risks for birth outcomes . . . 12

3C3: Mean differences for 6 mo outcomes . . . 13

3C4: Prevalence ratios for 6 mo outcomes . . . 14

Supplemental figure 3D: Maternal BMI **15**

3D1: Mean differences for birth outcomes . . . 15

3D2: Relative risks for birth outcomes . . . 16

3D3: Mean differences for 6 mo outcomes . . . 17

3D4: Prevalence ratios for 6 mo outcomes . . . 18

Supplemental figure 3E: Maternal age **19**

3E1: Mean differences for birth outcomes . . . 19

3E2: Relative risks for birth outcomes . . . 20

3E3: Mean differences for 6 mo outcomes . . . 21

3E4: Prevalence ratios for 6 mo outcomes . . . 22

Supplemental figure 3F: Maternal education **23**

3F1: Mean differences for birth outcomes . . . 23

3F2: Relative risks for birth outcomes . . . 24

3F3: Mean differences for 6 mo outcomes . . . 25

3F4: Prevalence ratios for 6 mo outcomes . . . 26

Supplemental figure 3G: Baseline anemia status **27**

3G1: Mean differences for birth outcomes . . . 27

3G2: Relative risks for birth outcomes . . . 28

3G3: Mean differences for 6 mo outcomes . . . 29

3G4: Prevalence ratios for 6 mo outcomes . . . 30

Supplemental figure 3H: Baseline inflammation status **31**

3H1: Mean differences for birth outcomes . . . 31

3H2: Relative risks for birth outcomes . . . 32

3H3: Mean differences for 6 mo outcomes . . . 33

3H4: Prevalence ratios for 6 mo outcomes . . . 34

Supplemental figure 3I: Baseline malaria status **35**

3I1: Mean differences for birth outcomes . . . 35

3I2: Relative risks for birth outcomes . . . 36

3I3: Mean differences for 6 mo outcomes . . . 37

3I4: Prevalence ratios for 6 mo outcomes . . . 38

Supplemental figure 3J: Gestational age at supplementation **39**

3J1: Mean differences for birth outcomes . . . 39

3J2: Relative risks for birth outcomes . . . 40

3J3: Mean differences for 6 mo outcomes . . . 41

3J4: Prevalence ratios for 6 mo outcomes . . . 42

Supplemental figure 3K: Compliance with supplementation **43**

3K1: Mean differences for birth outcomes . . . 43

3K2: Relative risks for birth outcomes . . . 44

3K3: Mean differences for 6 mo outcomes . . . 45

3K4: Prevalence ratios for 6 mo outcomes . . . 46

Supplemental figure 3L: Household socio-economic status **47**

3L1: Mean differences for birth outcomes . . . 47

3L2: Relative risks for birth outcomes . . . 48

3L3: Mean differences for 6 mo outcomes . . . 49

3L4: Prevalence ratios for 6 mo outcomes . . . 50

Supplemental figure 3M: Household food security **51**

3M1: Mean differences for birth outcomes 51

3M2: Relative risks for birth outcomes 52

3M3: Mean differences for 6 mo outcomes 53

3M4: Prevalence ratios for 6 mo outcomes 54

Supplemental figure 3N: Sanitation **55**

3N1: Mean differences for birth outcomes 55

3N2: Relative risks for birth outcomes 56

3N3: Mean differences for 6 mo outcomes 57

3N4: Prevalence ratios for 6 mo outcomes 58

These figures show pooled effects of SQ-LNS within study-level and individual-level characteristic subgroups along with the p-for-interaction. For definitions of effect modifiers, see Box 1 in the main paper. Individual study estimates were generated from log-binomial regression for dichotomous outcomes and simple linear regression for continuous outcomes with clustered observations using robust standard errors for cluster-randomized trials. Pooled sub-group estimates and statistical testing of the pooled interaction term were generated using inverse-variance weighting. For continuous outcomes the intervention effect is measured by the difference in mean of the SQ-LNS group minus IFA/SOC. For dichotomous outcomes analyzed via prevalence/risk ratios, the effect estimate is the prevalence/risk in the SQ-LNS group divided by the prevalence/risk in the IFA/SOC group. For dichotomous outcomes analyzed via prevalence/risk differences, the effect estimate is the prevalence/risk in the SQ-LNS group minus the prevalence/risk in the IFA/SOC group. The labels on the left y-axis correspond to the characteristic subgroups and their sample sizes. The values on the right indicate the pooled prevalence ratio and confidence interval within that subgroup.

LAZ, length-for-age z-score; WLZ, weight-for-length z-score; WAZ, weight for-age z-score; MUACZ, mid-upper arm circumference z-score; BMI, body mass index; HCZ, head circumference-for-age z-score; LGAZ, length-for-gestational-age z-score; HCGAZ, head circumference-for-gestational-age z-score; BMIZ, body mass index-for-age z-score; IFA/SOC, Iron and folic acid or standard of care; MD, mean difference; MMS, multiple micronutrient supplement; MUAC, mid-upper arm circumference; PR, prevalence ratio; PD, prevalence difference; RD, risk difference; RR, relative risk; SOC, standard of care; SQ-LNS, small-quantity lipid-based nutrient supplements; WGAZ, weight-for-gestational age z-score.

Supplemental figure 3A: Sex

3A1: Mean differences for birth outcomes

Outcome (p−for−interaction)	SQ−LNS N	IFA/SOC N	Mean difference (95% CI)
Birth weight (kg) (p=0.017)			
Male	901	1736	0.03 (−0.01, 0.06)
Female	914	1722	0.08 (0.05, 0.11)
Weight−for−age z score (p=0.014)			
Male	901	1736	0.06 (−0.02, 0.14)
Female	914	1722	0.19 (0.13, 0.26)
Weight−for−gestational age z−score (p=0.765)			
Male	425	423	0.13 (0.02, 0.25)
Female	441	428	0.11 (0.01, 0.21)
Birth length (cm) (p=0.133)			
Male	831	1677	0.12 (−0.05, 0.29)
Female	849	1657	0.30 (0.16, 0.45)
Length−for−age z−score (p=0.139)			
Male	831	1677	0.07 (−0.02, 0.16)
Female	849	1657	0.16 (0.09, 0.24)
Length−for−gestational age z−score (p=0.683)			
Male	355	365	0.14 (0.00, 0.27)
Female	377	363	0.09 (−0.02, 0.21)
BMI z−score (p=0.025)			
Male	829	1670	0.03 (−0.06, 0.12)
Female	847	1656	0.17 (0.09, 0.25)
Head circumference (cm) (p=0.101)			
Male	832	1678	0.06 (−0.05, 0.18)
Female	848	1658	0.18 (0.09, 0.28)
Head circumference−for−age z−score (p=0.073)			
Male	832	1678	0.05 (−0.04, 0.14)
Female	848	1658	0.16 (0.08, 0.24)
Head circumference−for−gestational age z score (p=0.391)			
Male	356	365	0.07 (−0.05, 0.20)
Female	376	364	0.14 (0.02, 0.26)
Mid−upper arm circumference (cm) (p=0.193)			
Male	730	1562	0.04 (−0.04, 0.13)
Female	731	1558	0.12 (0.04, 0.19)
Duration (wk) (p=0.003)			
Male	918	1748	−0.06 (−0.24, 0.13)
Female	922	1726	0.30 (0.15, 0.45)

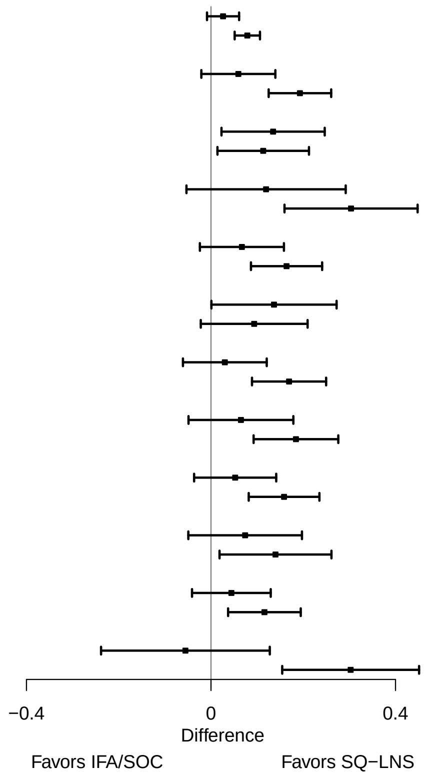

Supplemental figure 3A: Sex

3A2: Relative risks for birth outcomes

Outcome (p−for−interaction)	SQ−LNS N	IFA/SOC N	Relative risk (95% CI)
Low birth weight (p=0.016)			
Male	901	1736	1.01 (0.86, 1.18)
Female	914	1722	0.80 (0.71, 0.90)
Birth weight < 2 kg (p=0.018)			
Male	797	1620	1.14 (0.79, 1.66)
Female	795	1622	0.50 (0.30, 0.84)
Small−for−gestational age (p=0.283)			
Male	881	1707	0.99 (0.91, 1.06)
Female	892	1701	0.93 (0.87, 1.00)
Large−for−gestational age (p=0.589)			
Male	344	339	1.06 (0.49, 2.30)
Female	350	352	0.78 (0.35, 1.75)
Newborn stunting (p=0.332)			
Male	831	1677	0.88 (0.74, 1.06)
Female	849	1657	0.76 (0.64, 0.90)
Low LGAZ (p=0.722)			
Male	811	1648	0.94 (0.79, 1.13)
Female	827	1636	0.89 (0.72, 1.09)
Low BMIZ (p=0.017)			
Male	829	1670	1.02 (0.89, 1.16)
Female	847	1656	0.78 (0.66, 0.92)
Low HCZ (p=0.848)			
Male	832	1678	0.86 (0.68, 1.08)
Female	848	1658	0.83 (0.71, 0.96)
Low HCGAZ (p=0.859)			
Male	696	1519	0.89 (0.70, 1.14)
Female	689	1503	0.85 (0.69, 1.05)
Preterm birth (p=0.080)			
Male	918	1748	1.08 (0.86, 1.36)
Female	922	1726	0.82 (0.65, 1.03)

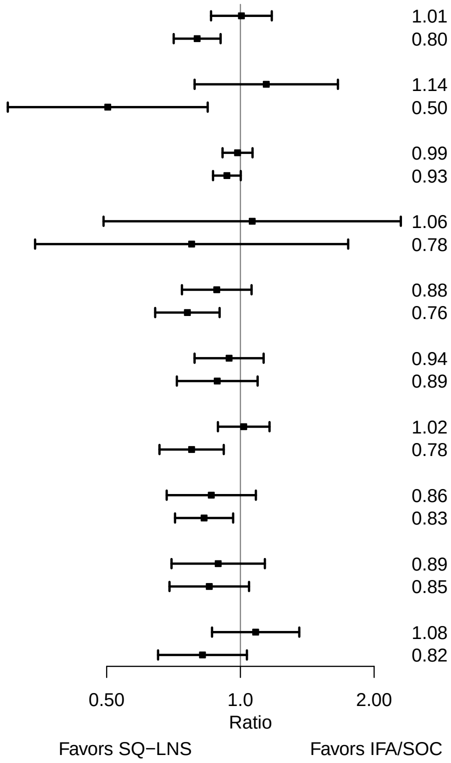

Supplemental figure 3A: Sex

3A3: Mean differences for 6 mo outcomes

Outcome (p−for−interaction)	SQ−LNS N	IFA/SOC N	Mean difference (95% CI)
Weight−for−age z score (p=0.005)			
Male	849	1661	−0.05 (−0.15, 0.06)
Female	867	1652	0.12 (0.03, 0.20)
Length−for−age z−score (p=0.008)			
Male	849	1660	−0.02 (−0.11, 0.08)
Female	868	1655	0.12 (0.04, 0.20)
Weight−for−length z−score (p=0.075)			
Male	849	1660	−0.06 (−0.15, 0.04)
Female	867	1652	0.05 (−0.04, 0.14)
Head circumference−for−age z−score (p=0.035)			
Male	849	1659	−0.05 (−0.13, 0.02)
Female	868	1652	0.06 (−0.02, 0.14)
MUAC−for−age z−score (p=0.025)			
Male	747	1550	−0.07 (−0.16, 0.03)
Female	751	1552	0.07 (−0.03, 0.16)

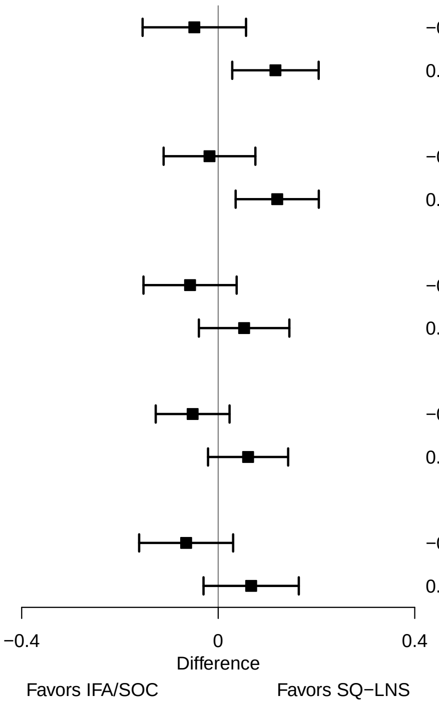

Supplemental figure 3A: Sex

3A4: Prevalence ratios for 6 mo outcomes

Outcome (p−for−interaction)	SQ−LNS N	IFA/SOC N	Prevalence ratio (95% CI)
Underweight (p=0.001)			
Male	849	1661	1.08 (0.88, 1.32)
Female	867	1652	0.74 (0.59, 0.93)
Stunted (p=0.530)			
Male	849	1660	0.93 (0.79, 1.09)
Female	868	1655	0.87 (0.73, 1.04)
Wasted (p=0.503)			
Male	747	1549	0.88 (0.55, 1.40)
Female	750	1549	1.08 (0.64, 1.82)
Low HCAZ (p=0.001)			
Male	849	1659	1.09 (0.94, 1.26)
Female	868	1652	0.70 (0.56, 0.87)
Low MUAC (p=0.972)			
Male	747	1550	1.19 (0.85, 1.65)
Female	751	1552	1.02 (0.71, 1.45)
Acute malnutrition (p=0.365)			
Male	747	1549	0.91 (0.66, 1.25)
Female	750	1549	1.09 (0.79, 1.49)

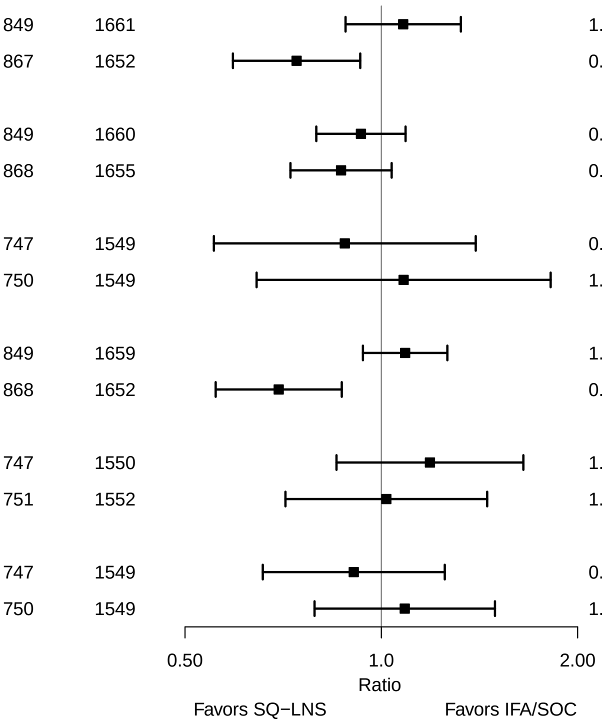

Supplemental figure 3B: Birth order

3B1: Mean differences for birth outcomes

Outcome (p−for−interaction)	SQ−LNS N	IFA/SOC N	Mean difference (95% CI)
Birth weight (kg) (p=0.983)			
Later born	1250	2281	0.05 (0.03, 0.08)
Firstborn	564	1171	0.05 (0.01, 0.10)
Weight−for−age z score (p=0.890)			
Later born	1250	2281	0.13 (0.07, 0.19)
Firstborn	564	1171	0.14 (0.03, 0.24)
Weight−for−gestational age z−score (p=0.129)			
Later born	677	663	0.10 (0.02, 0.19)
Firstborn	189	187	0.24 (0.06, 0.43)
Birth length (cm) (p=0.861)			
Later born	1150	2180	0.21 (0.08, 0.34)
Firstborn	529	1148	0.21 (−0.02, 0.43)
Length−for−age z−score (p=0.997)			
Later born	1150	2180	0.12 (0.06, 0.19)
Firstborn	529	1148	0.10 (−0.01, 0.22)
Length−for−gestational age z−score (p=0.156)			
Later born	578	562	0.10 (0.00, 0.19)
Firstborn	154	165	0.22 (0.01, 0.42)
BMI z−score (p=0.961)			
Later born	1148	2175	0.10 (0.03, 0.17)
Firstborn	527	1145	0.12 (0.01, 0.23)
Head circumference (cm) (p=0.582)			
Later born	1150	2182	0.13 (0.05, 0.22)
Firstborn	529	1148	0.08 (−0.06, 0.23)
Head circumference−for−age z−score (p=0.430)			
Later born	1150	2182	0.12 (0.05, 0.19)
Firstborn	529	1148	0.06 (−0.05, 0.17)
Head circumference−for−gestational age z score (p=0.986)			
Later born	578	564	0.08 (−0.02, 0.18)
Firstborn	154	164	0.08 (−0.12, 0.29)
Mid−upper arm circumference (cm) (p=0.760)			
Later born	945	1975	0.07 (0.01, 0.13)
Firstborn	515	1139	0.11 (0.00, 0.21)
Duration (wk) (p=0.406)			
Later born	1261	2288	0.15 (0.00, 0.29)
Firstborn	593	1199	0.06 (−0.15, 0.27)

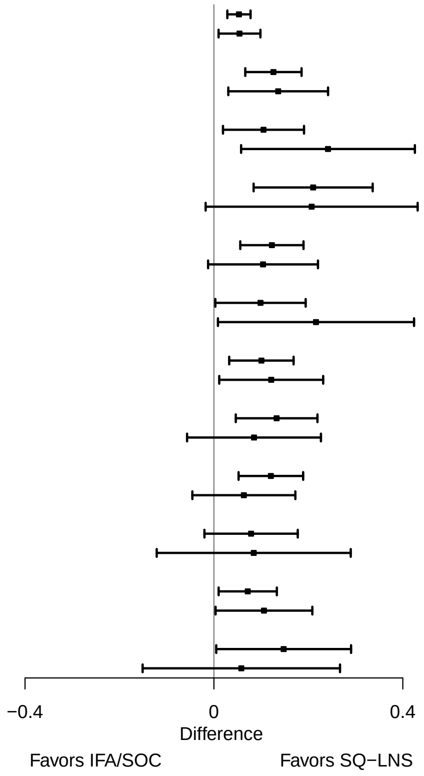

Supplemental figure 3B: Birth order

3B2: Relative risks for birth outcomes

Outcome (p−for−interaction)	SQ−LNS N	IFA/SOC N	Relative risk (95% CI)
Low birth weight (p=0.972)			
Later born	1250	2281	0.88 (0.77, 0.99)
Firstborn	564	1171	0.89 (0.76, 1.05)
Birth weight < 2 kg (p=0.602)			
Later born	1041	2074	0.84 (0.58, 1.23)
Firstborn	550	1162	0.72 (0.50, 1.04)
Small−for−gestational age (p=0.633)			
Later born	1210	2232	0.94 (0.87, 1.00)
Firstborn	562	1170	0.98 (0.90, 1.06)
Large−for−gestational age (p=0.316)			
Later born	1041	2074	0.88 (0.51, 1.53)
Firstborn	550	1162	1.85 (0.52, 6.58)
Newborn stunting (p=0.195)			
Later born	1150	2180	0.86 (0.74, 1.01)
Firstborn	529	1148	0.76 (0.64, 0.91)
Low LGAZ (p=0.809)			
Later born	1110	2131	0.90 (0.76, 1.06)
Firstborn	527	1147	0.88 (0.72, 1.09)
Low BMIZ (p=0.766)			
Later born	939	1968	0.91 (0.78, 1.06)
Firstborn	513	1136	0.88 (0.78, 1.00)
Low HCZ (p=0.069)			
Later born	1150	2182	0.92 (0.81, 1.05)
Firstborn	529	1148	0.74 (0.61, 0.91)
Low HCGAZ (p=0.142)			
Later born	729	1757	0.98 (0.79, 1.21)
Firstborn	474	1094	0.76 (0.61, 0.95)
Preterm birth (p=0.517)			
Later born	1261	2288	0.90 (0.73, 1.11)
Firstborn	593	1199	1.00 (0.80, 1.26)

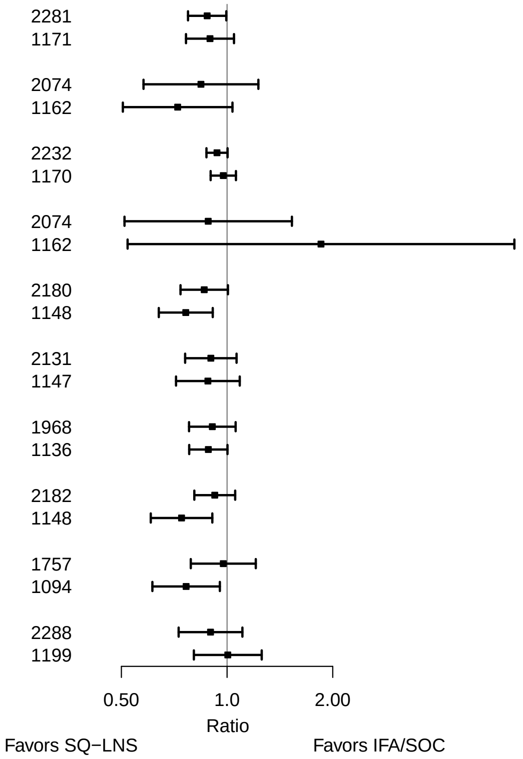

Supplemental figure 3B: Birth order

3B3: Mean differences for 6 mo outcomes

Outcome (p−for−interaction)	SQ−LNS N	IFA/SOC N	Mean difference (95% CI)
Weight−for−age z score (p=0.143)			
Later born	1176	2196	0.06 (−0.02, 0.14)
Firstborn	540	1113	0.00 (−0.13, 0.12)
Length−for−age z−score (p=0.134)			
Later born	1176	2199	0.08 (0.00, 0.15)
Firstborn	541	1112	−0.04 (−0.15, 0.06)
Weight−for−length z−score (p=0.594)			
Later born	1176	2196	0.00 (−0.08, 0.08)
Firstborn	540	1112	−0.02 (−0.13, 0.08)
Head circumference−for−age z−score (p=0.386)			
Later born	1176	2194	0.03 (−0.04, 0.09)
Firstborn	541	1113	−0.01 (−0.11, 0.09)
MUAC−for−age z−score (p=0.879)			
Later born	971	1995	−0.01 (−0.09, 0.08)
Firstborn	527	1103	0.01 (−0.12, 0.13)

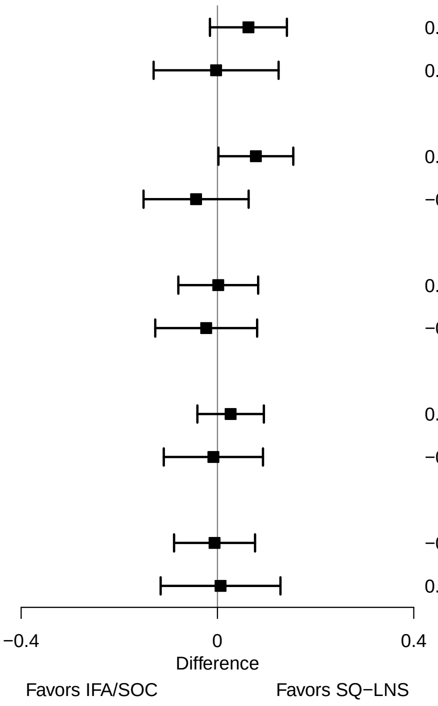

Supplemental figure 3B: Birth order

3B4: Prevalence ratios for 6 mo outcomes

Outcome (p−for−interaction)	SQ−LNS N	IFA/SOC N	Prevalence ratio (95% CI)
Underweight (p=0.026)			
Later born	1176	2196	0.79 (0.66, 0.93)
Firstborn	540	1113	1.11 (0.87, 1.42)
Stunted (p=0.074)			
Later born	1176	2199	0.84 (0.72, 0.97)
Firstborn	541	1112	1.04 (0.85, 1.28)
Wasted (p=0.766)			
Later born	691	1695	0.92 (0.61, 1.39)
Firstborn	456	1035	0.98 (0.56, 1.70)
Low HCAZ (p=0.202)			
Later born	1176	2194	0.84 (0.73, 0.97)
Firstborn	541	1113	0.99 (0.81, 1.21)
Low MUAC (p=0.117)			
Later born	971	1995	0.83 (0.60, 1.16)
Firstborn	527	1103	1.38 (0.92, 2.06)
Acute malnutrition (p=0.171)			
Later born	971	1992	0.89 (0.68, 1.17)
Firstborn	526	1102	1.27 (0.87, 1.85)

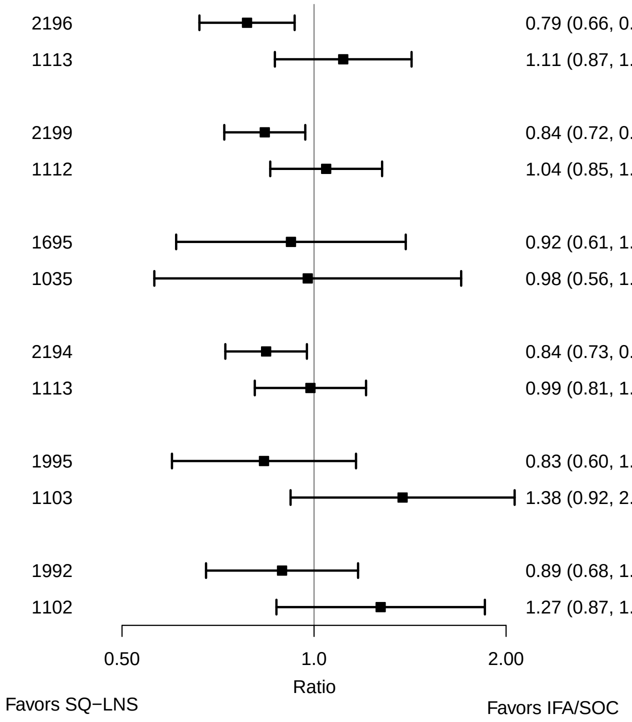

Supplemental figure 3C: Maternal height

3C1: Mean differences for birth outcomes

Outcome (p−for−interaction)	SQ−LNS N	IFA/SOC N	Mean difference (95% CI)
Birth weight (kg) (p=0.656)			
At least 150.1 cm	1154	1960	0.04 (0.01, 0.08)
Less than 150.1 cm	624	1415	0.04 (0.01, 0.08)
Weight−for−age z score (p=0.682)			
At least 150.1 cm	1154	1960	0.11 (0.03, 0.18)
Less than 150.1 cm	624	1415	0.11 (0.03, 0.20)
Weight−for−gestational age z−score (p=0.922)			
At least 150.1 cm	662	625	0.09 (−0.02, 0.19)
Less than 150.1 cm	198	218	0.15 (0.04, 0.27)
Birth length (cm) (p=0.767)			
At least 150.1 cm	1038	1856	0.17 (0.02, 0.33)
Less than 150.1 cm	605	1395	0.15 (−0.02, 0.32)
Length−for−age z−score (p=0.795)			
At least 150.1 cm	1038	1856	0.10 (0.02, 0.18)
Less than 150.1 cm	605	1395	0.10 (0.00, 0.19)
Length−for−gestational age z−score (p=0.999)			
At least 150.1 cm	546	521	0.09 (−0.03, 0.21)
Less than 150.1 cm	180	199	0.10 (−0.04, 0.23)
BMI z−score (p=0.591)			
At least 150.1 cm	1035	1851	0.09 (0.01, 0.17)
Less than 150.1 cm	604	1392	0.10 (0.01, 0.19)
Head circumference (cm) (p=0.804)			
At least 150.1 cm	1037	1857	0.12 (0.02, 0.22)
Less than 150.1 cm	606	1396	0.06 (−0.07, 0.19)
Head circumference−for−age z−score (p=0.800)			
At least 150.1 cm	1037	1857	0.10 (0.02, 0.18)
Less than 150.1 cm	606	1396	0.07 (−0.04, 0.17)
Head circumference−for−gestational age z score (p=0.741)			
At least 150.1 cm	545	522	0.12 (0.00, 0.25)
Less than 150.1 cm	181	199	0.04 (−0.12, 0.20)
Mid−upper arm circumference (cm) (p=0.263)			
At least 150.1 cm	993	1831	0.05 (−0.02, 0.13)
Less than 150.1 cm	432	1206	0.08 (−0.02, 0.18)
Duration (wk) (p=0.269)			
At least 150.1 cm	1209	2017	0.06 (−0.10, 0.22)
Less than 150.1 cm	609	1393	0.18 (−0.03, 0.39)

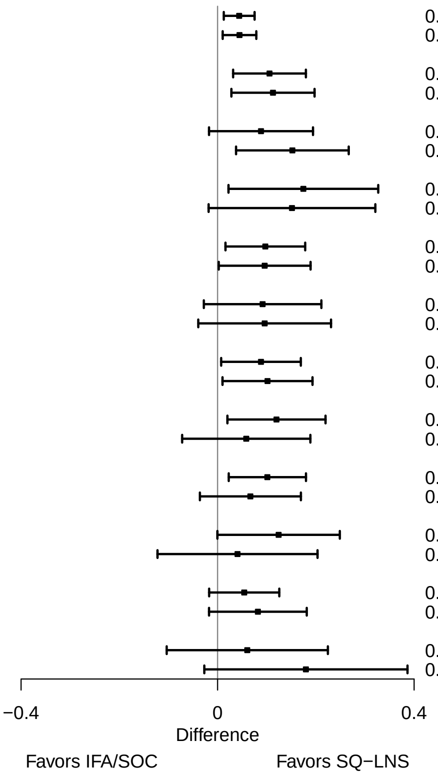

Supplemental figure 3C: Maternal height

3C2: Relative risks for birth outcomes

Outcome (p−for−interaction)	SQ−LNS N	IFA/SOC N	Relative risk (95% CI)
Low birth weight (p=0.365)			
At least 150.1 cm	863	1679	0.89 (0.77, 1.04)
Less than 150.1 cm	613	1398	0.94 (0.82, 1.08)
Birth weight < 2 kg (p=0.653)			
At least 150.1 cm	816	1653	0.92 (0.60, 1.42)
Less than 150.1 cm	438	1208	0.76 (0.50, 1.15)
Small−for−gestational age (p=0.829)			
At least 150.1 cm	1145	1953	0.95 (0.87, 1.04)
Less than 150.1 cm	591	1372	0.97 (0.91, 1.03)
Newborn stunting (p=0.755)			
At least 150.1 cm	747	1575	0.83 (0.64, 1.08)
Less than 150.1 cm	594	1378	0.85 (0.72, 1.00)
Low LGAZ (p=0.071)			
At least 150.1 cm	700	1549	0.79 (0.65, 0.96)
Less than 150.1 cm	419	1188	1.03 (0.85, 1.24)
Low BMIZ (p=0.935)			
At least 150.1 cm	697	1544	0.91 (0.80, 1.04)
Less than 150.1 cm	418	1185	0.92 (0.79, 1.07)
Low HCZ (p=0.605)			
At least 150.1 cm	699	1550	0.89 (0.76, 1.04)
Less than 150.1 cm	420	1189	0.83 (0.67, 1.03)
Preterm birth (p=0.252)			
At least 150.1 cm	1209	2017	1.03 (0.83, 1.27)
Less than 150.1 cm	609	1393	0.85 (0.65, 1.12)

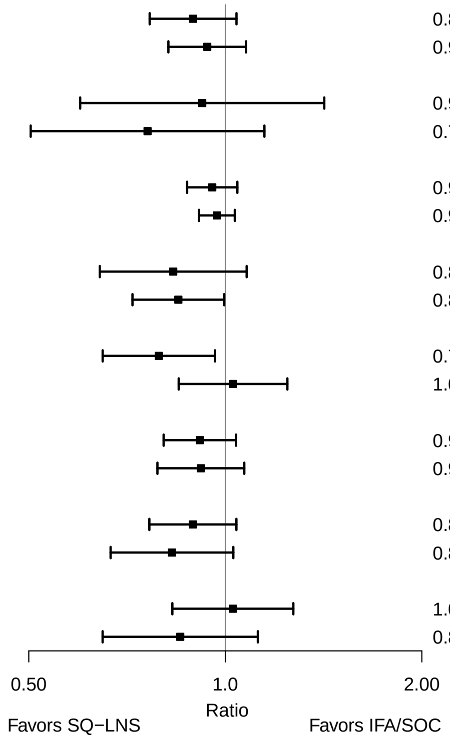

Supplemental figure 3C: Maternal height

3C3: Mean differences for 6 mo outcomes

Outcome (p−for−interaction)	SQ−LNS N	IFA/SOC N	Mean difference (95% CI)
Weight−for−age z score (p=0.513)			
At least 150.1 cm	1085	1882	0.05 (−0.05, 0.14)
Less than 150.1 cm	598	1359	0.01 (−0.10, 0.12)
Length−for−age z−score (p=0.490)			
At least 150.1 cm	1086	1884	0.03 (−0.06, 0.12)
Less than 150.1 cm	598	1359	0.08 (−0.01, 0.17)
Weight−for−length z−score (p=0.073)			
At least 150.1 cm	1085	1881	0.04 (−0.05, 0.13)
Less than 150.1 cm	598	1359	−0.06 (−0.15, 0.04)
Head circumference−for−age z−score (p=0.861)			
At least 150.1 cm	1086	1884	0.01 (−0.07, 0.08)
Less than 150.1 cm	598	1355	0.00 (−0.08, 0.09)
MUAC−for−age z−score (p=0.664)			
At least 150.1 cm	1039	1858	0.02 (−0.06, 0.10)
Less than 150.1 cm	427	1172	−0.05 (−0.17, 0.06)

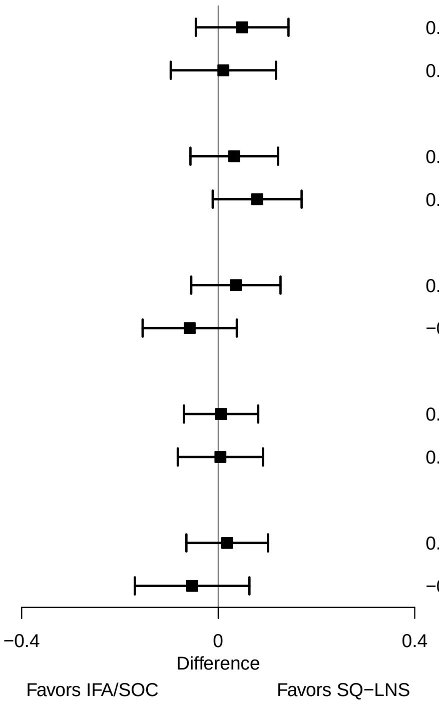

Supplemental figure 3C: Maternal height

3C4: Prevalence ratios for 6 mo outcomes

Outcome (p−for−interaction)	SQ−LNS N	IFA/SOC N	Prevalence ratio (95% CI)
Underweight (p=0.478)			
At least 150.1 cm	820	1633	0.91 (0.69, 1.21)
Less than 150.1 cm	587	1343	0.93 (0.77, 1.13)
Stunted (p=0.797)			
At least 150.1 cm	1086	1884	0.95 (0.79, 1.16)
Less than 150.1 cm	598	1359	0.89 (0.77, 1.01)
Low HCAZ (p=0.646)			
At least 150.1 cm	820	1633	0.95 (0.79, 1.14)
Less than 150.1 cm	587	1339	0.89 (0.76, 1.03)
Low MUAC (p=0.037)			
At least 150.1 cm	1039	1858	1.14 (0.85, 1.53)
Less than 150.1 cm	427	1172	0.88 (0.58, 1.33)
Acute malnutrition (p=0.079)			
At least 150.1 cm	1038	1854	1.09 (0.83, 1.44)
Less than 150.1 cm	427	1172	0.88 (0.64, 1.21)

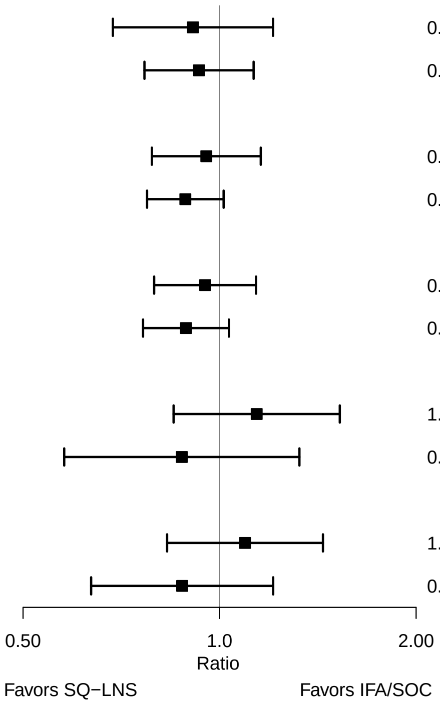

Supplemental figure 3D: Maternal BMI

3D1: Mean differences for birth outcomes

**Outcome (p−for−interaction)**	**SQ−LNS N**	**IFA/SOC N**	**Mean difference (95% CI)**
Birth weight (kg) (p=0.233)			
At least 20 kg/m²	1041	1806	0.04 (0.02, 0.07)
Less than 20 kg/m²	736	1567	0.06 (0.03, 0.09)
Weight−for−age z score (p=0.276)			
At least 20 kg/m²	1041	1806	0.11 (0.04, 0.18)
Less than 20 kg/m²	736	1567	0.14 (0.06, 0.22)
Weight−for−gestational age z−score (p=0.995)			
At least 20 kg/m²	613	588	0.15 (0.07, 0.23)
Less than 20 kg/m²	246	253	0.06 (−0.10, 0.23)
Birth length (cm) (p=0.186)			
At least 20 kg/m²	975	1749	0.16 (0.02, 0.30)
Less than 20 kg/m²	667	1501	0.27 (0.10, 0.45)
Length−for−age z−score (p=0.303)			
At least 20 kg/m²	975	1749	0.10 (0.02, 0.18)
Less than 20 kg/m²	667	1501	0.14 (0.05, 0.23)
Length−for−gestational age z−score (p=0.791)			
At least 20 kg/m²	548	532	0.13 (0.04, 0.22)
Less than 20 kg/m²	177	187	0.12 (−0.09, 0.33)
BMI z−score (p=0.239)			
At least 20 kg/m²	974	1748	0.09 (0.01, 0.16)
Less than 20 kg/m²	664	1494	0.13 (0.04, 0.22)
Head circumference (cm) (p=0.111)			
At least 20 kg/m²	973	1751	0.07 (−0.01, 0.16)
Less than 20 kg/m²	669	1501	0.17 (0.05, 0.28)
Head circumference−for−age z−score (p=0.185)			
At least 20 kg/m²	973	1751	0.08 (0.01, 0.15)
Less than 20 kg/m²	669	1501	0.13 (0.04, 0.22)
Head circumference−for−gestational age z score (p=0.884)			
At least 20 kg/m²	546	533	0.11 (0.01, 0.21)
Less than 20 kg/m²	179	187	0.10 (−0.11, 0.31)
Mid−upper arm circumference (cm) (p=0.081)			
At least 20 kg/m²	773	1548	0.03 (−0.04, 0.10)
Less than 20 kg/m²	651	1488	0.11 (0.03, 0.20)
Duration (wk) (p=0.840)			
At least 20 kg/m²	1062	1820	0.11 (−0.05, 0.27)
Less than 20 kg/m²	755	1588	0.12 (−0.08, 0.31)

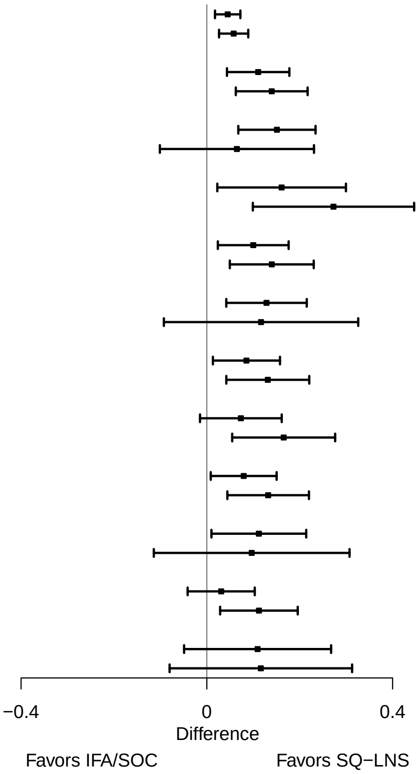

Supplemental figure 3D: Maternal BMI

3D2: Relative risks for birth outcomes

Outcome (p−for−interaction)	SQ−LNS N	IFA/SOC N	Relative risk (95% CI)
Low birth weight (p=0.248)			
At least 20 kg/m²	1041	1806	0.93 (0.80, 1.08)
Less than 20 kg/m²	736	1567	0.87 (0.76, 0.98)
Birth weight < 2 kg (p=0.220)			
At least 20 kg/m²	582	1357	1.08 (0.66, 1.78)
Less than 20 kg/m²	671	1502	0.69 (0.46, 1.02)
Small−for−gestational age (p=0.923)			
At least 20 kg/m²	1003	1759	0.95 (0.87, 1.03)
Less than 20 kg/m²	732	1564	0.96 (0.90, 1.03)
Large−for−gestational age (p=0.488)			
At least 20 kg/m²	582	1357	0.77 (0.31, 1.88)
Less than 20 kg/m²	671	1502	1.46 (0.52, 4.12)
Newborn stunting (p=0.342)			
At least 20 kg/m²	975	1749	0.89 (0.75, 1.06)
Less than 20 kg/m²	667	1501	0.80 (0.69, 0.93)
Low LGAZ (p=0.640)			
At least 20 kg/m²	937	1702	0.95 (0.78, 1.15)
Less than 20 kg/m²	663	1498	0.88 (0.75, 1.04)
Low BMIZ (p=0.683)			
At least 20 kg/m²	974	1748	0.89 (0.75, 1.05)
Less than 20 kg/m²	664	1494	0.89 (0.79, 1.00)
Low HCZ (p=0.049)			
At least 20 kg/m²	973	1751	0.97 (0.81, 1.17)
Less than 20 kg/m²	669	1501	0.77 (0.66, 0.90)
Low HCGAZ (p=0.157)			
At least 20 kg/m²	637	1411	0.96 (0.75, 1.24)
Less than 20 kg/m²	531	1363	0.81 (0.69, 0.95)
Preterm birth (p=0.687)			
At least 20 kg/m²	887	1653	0.92 (0.71, 1.19)
Less than 20 kg/m²	741	1578	0.98 (0.78, 1.24)

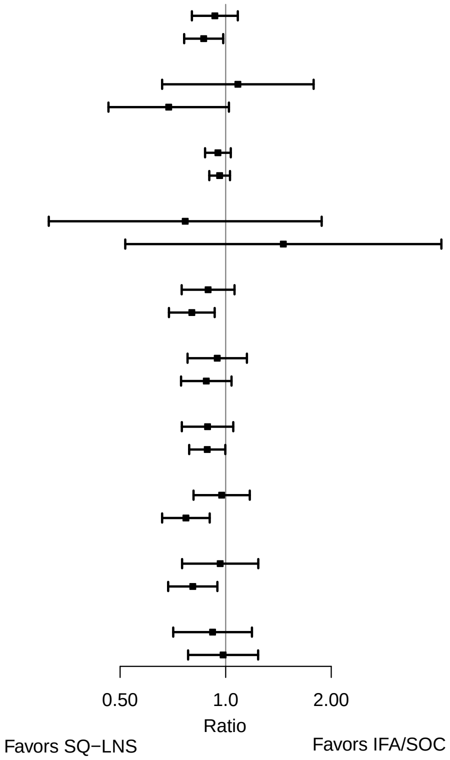

Supplemental figure 3D: Maternal BMI

3D3: Mean differences for 6 mo outcomes

Outcome (p−for−interaction)	SQ−LNS N	IFA/SOC N	Mean difference (95% CI)
Weight−for−age z score (p=0.402)			
At least 20 kg/m²	987	1730	0.05 (−0.04, 0.14)
Less than 20 kg/m²	695	1510	0.05 (−0.06, 0.16)
Length−for−age z−score (p=0.922)			
At least 20 kg/m²	987	1732	0.09 (0.00, 0.18)
Less than 20 kg/m²	696	1510	0.05 (−0.05, 0.14)
Weight−for−length z−score (p=0.115)			
At least 20 kg/m²	987	1730	−0.02 (−0.10, 0.06)
Less than 20 kg/m²	695	1509	0.03 (−0.07, 0.14)
Head circumference−for−age z−score (p=0.773)			
At least 20 kg/m²	987	1728	0.02 (−0.05, 0.09)
Less than 20 kg/m²	696	1510	0.02 (−0.07, 0.11)
MUAC−for−age z−score (p=0.529)			
At least 20 kg/m²	787	1530	−0.01 (−0.10, 0.08)
Less than 20 kg/m²	678	1499	0.02 (−0.08, 0.12)

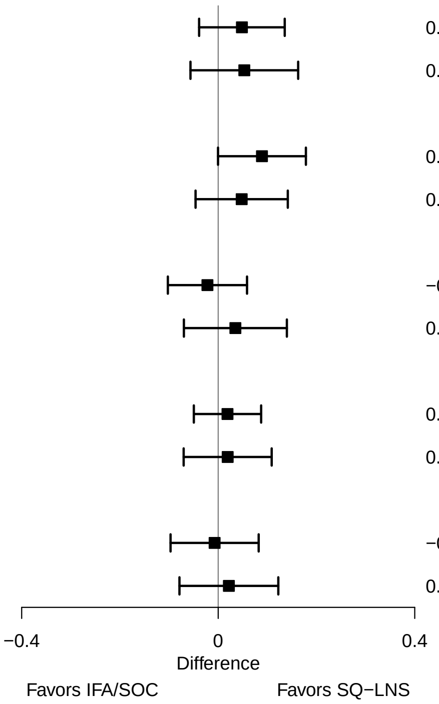

Supplemental figure 3D: Maternal BMI

3D4: Prevalence ratios for 6 mo outcomes

Outcome (p−for−interaction)	SQ−LNS N	IFA/SOC N	Prevalence ratio (95% CI)
Underweight (p=0.838)			
At least 20 kg/m²	987	1730	0.83 (0.67, 1.04)
Less than 20 kg/m²	695	1510	0.95 (0.79, 1.14)
Stunted (p=0.802)			
At least 20 kg/m²	987	1732	0.88 (0.75, 1.03)
Less than 20 kg/m²	696	1510	0.91 (0.78, 1.06)
Wasted (p=0.916)			
At least 20 kg/m²	787	1528	0.85 (0.48, 1.52)
Less than 20 kg/m²	677	1497	1.01 (0.71, 1.44)
Low HCAZ (p=0.595)			
At least 20 kg/m²	787	1529	0.93 (0.77, 1.13)
Less than 20 kg/m²	678	1498	0.88 (0.73, 1.06)
Low MUAC (p=0.665)			
At least 20 kg/m²	787	1530	0.93 (0.61, 1.43)
Less than 20 kg/m²	678	1499	1.06 (0.78, 1.45)
Acute malnutrition (p=0.605)			
At least 20 kg/m²	787	1528	0.94 (0.65, 1.38)
Less than 20 kg/m²	677	1497	1.06 (0.80, 1.39)

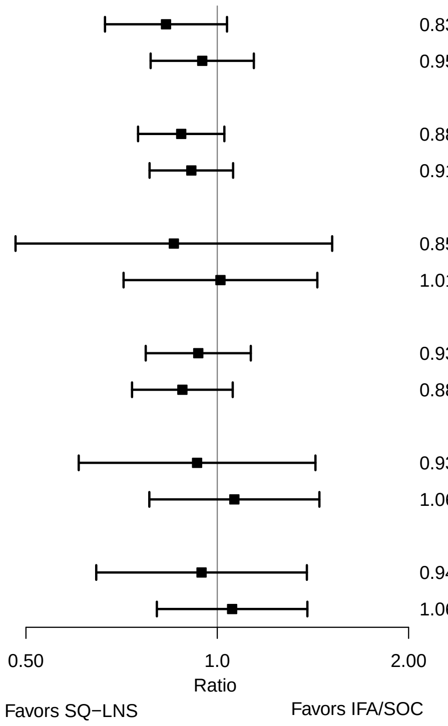

Supplemental figure 3E: Maternal age

3E1: Mean differences for birth outcomes

Outcome (p−for−interaction)	SQ−LNS N	IFA/SOC N	Mean difference (95% CI)
Birth weight (kg) (p=0.257)			
At least 25 y	723	1173	0.07 (0.03, 0.11)
Less than 25 y	1092	2285	0.04 (0.01, 0.07)
Weight−for−age z score (p=0.395)			
At least 25 y	723	1173	0.15 (0.07, 0.24)
Less than 25 y	1092	2285	0.10 (0.03, 0.17)
Weight−for−gestational age z−score (p=0.256)			
At least 25 y	451	455	0.22 (0.12, 0.33)
Less than 25 y	415	396	0.07 (−0.05, 0.19)
Birth length (cm) (p=0.522)			
At least 25 y	668	1113	0.26 (0.08, 0.45)
Less than 25 y	1012	2221	0.14 (0.00, 0.29)
Length−for−age z−score (p=0.699)			
At least 25 y	668	1113	0.13 (0.04, 0.23)
Less than 25 y	1012	2221	0.09 (0.01, 0.17)
Length−for−gestational age z−score (p=0.192)			
At least 25 y	397	395	0.18 (0.05, 0.32)
Less than 25 y	335	333	0.02 (−0.12, 0.17)
BMI z−score (p=0.245)			
At least 25 y	667	1111	0.14 (0.05, 0.24)
Less than 25 y	1009	2215	0.08 (0.00, 0.15)
Head circumference (cm) (p=0.290)			
At least 25 y	668	1115	0.19 (0.07, 0.32)
Less than 25 y	1012	2221	0.06 (−0.03, 0.16)
Head circumference−for−age z−score (p=0.395)			
At least 25 y	668	1115	0.15 (0.06, 0.25)
Less than 25 y	1012	2221	0.07 (−0.01, 0.15)
Head circumference−for−gestational age z score (p=0.862)			
At least 25 y	397	397	0.14 (0.00, 0.27)
Less than 25 y	335	332	0.10 (−0.05, 0.24)
Mid−upper arm circumference (cm) (p=0.621)			
At least 25 y	582	1012	0.10 (0.01, 0.18)
Less than 25 y	879	2108	0.07 (−0.01, 0.14)
Duration (wk) (p=0.020)			
At least 25 y	744	1189	−0.06 (−0.27, 0.15)
Less than 25 y	1111	2304	0.23 (0.06, 0.39)

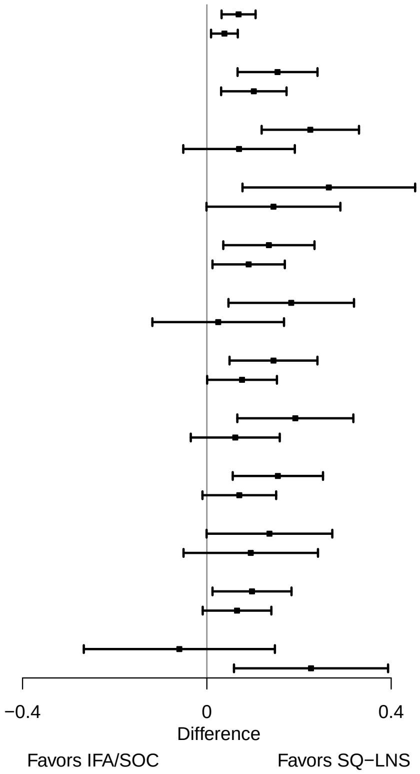

Supplemental figure 3E: Maternal age

3E2: Relative risks for birth outcomes

Outcome (p−for−interaction)	SQ−LNS N	IFA/SOC N	Relative risk (95% CI)
Low birth weight (p=0.481)			
At least 25 y	723	1173	0.85 (0.70, 1.03)
Less than 25 y	1092	2285	0.92 (0.82, 1.03)
Birth weight < 2 kg (p=0.022)			
At least 25 y	633	1070	1.26 (0.77, 2.08)
Less than 25 y	959	2172	0.64 (0.47, 0.86)
Small−for−gestational age (p=0.052)			
At least 25 y	705	1151	0.87 (0.77, 0.97)
Less than 25 y	1068	2257	1.00 (0.94, 1.06)
Large−for−gestational age (p=0.279)			
At least 25 y	633	1070	0.83 (0.45, 1.54)
Less than 25 y	959	2172	1.43 (0.59, 3.47)
Newborn stunting (p=0.343)			
At least 25 y	668	1113	0.91 (0.72, 1.16)
Less than 25 y	1012	2221	0.80 (0.70, 0.92)
Low LGAZ (p=0.956)			
At least 25 y	650	1091	0.94 (0.74, 1.18)
Less than 25 y	988	2193	0.91 (0.77, 1.06)
Low BMIZ (p=0.462)			
At least 25 y	667	1111	0.83 (0.65, 1.05)
Less than 25 y	1009	2215	0.93 (0.84, 1.03)
Low HCZ (p=0.920)			
At least 25 y	668	1115	0.84 (0.66, 1.06)
Less than 25 y	1012	2221	0.85 (0.71, 1.01)
Low HCGAZ (p=0.395)			
At least 25 y	510	956	0.97 (0.71, 1.32)
Less than 25 y	875	2066	0.84 (0.71, 1.00)
Preterm birth (p=0.236)			
At least 25 y	744	1189	1.07 (0.82, 1.39)
Less than 25 y	1111	2304	0.87 (0.71, 1.06)

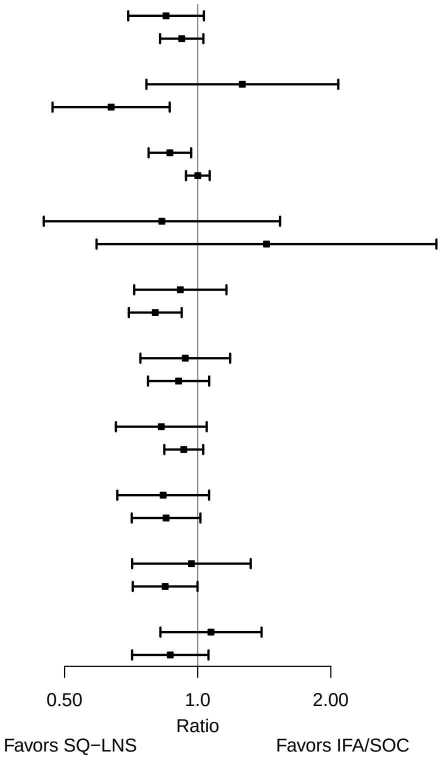

Supplemental figure 3E: Maternal age

3E3: Mean differences for 6 mo outcomes

Outcome (p−for−interaction)	SQ−LNS N	IFA/SOC N	Mean difference (95% CI)
Weight−for−age z score (p=0.924)			
At least 25 y	671	1128	0.04 (−0.08, 0.15)
Less than 25 y	1045	2185	0.04 (−0.05, 0.13)
Length−for−age z−score (p=0.267)			
At least 25 y	672	1129	0.11 (0.00, 0.21)
Less than 25 y	1045	2186	0.03 (−0.06, 0.11)
Weight−for−length z−score (p=0.111)			
At least 25 y	671	1128	−0.04 (−0.15, 0.07)
Less than 25 y	1045	2184	0.02 (−0.05, 0.10)
Head circumference−for−age z−score (p=0.698)			
At least 25 y	672	1127	0.03 (−0.07, 0.12)
Less than 25 y	1045	2184	0.00 (−0.07, 0.08)
MUAC−for−age z−score (p=0.109)			
At least 25 y	582	1025	−0.07 (−0.18, 0.04)
Less than 25 y	916	2077	0.03 (−0.05, 0.12)

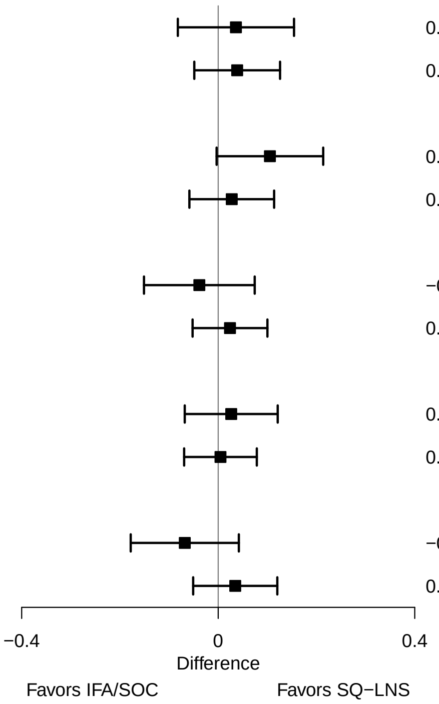

Supplemental figure 3E: Maternal age

3E4: Prevalence ratios for 6 mo outcomes

**Outcome (p−for−interaction)**	**SQ−LNS N**	**IFA/SOC N**	**Prevalence ratio (95% CI)**
Underweight (p=0.830)			
At least 25 y	671	1128	0.85 (0.66, 1.10)
Less than 25 y	1045	2185	0.96 (0.81, 1.14)
Stunted (p=0.953)			
At least 25 y	672	1129	0.92 (0.75, 1.14)
Less than 25 y	1045	2186	0.90 (0.77, 1.05)
Wasted (p=0.519)			
At least 25 y	581	1024	0.81 (0.50, 1.32)
Less than 25 y	916	2074	1.09 (0.74, 1.62)
Low HCAZ (p=0.341)			
At least 25 y	672	1127	0.81 (0.66, 1.00)
Less than 25 y	1045	2184	0.95 (0.80, 1.11)
Low MUAC (p=0.289)			
At least 25 y	582	1025	0.88 (0.59, 1.32)
Less than 25 y	916	2077	1.14 (0.87, 1.51)
Acute malnutrition (p=0.384)			
At least 25 y	581	1024	0.92 (0.66, 1.28)
Less than 25 y	916	2074	1.08 (0.84, 1.37)

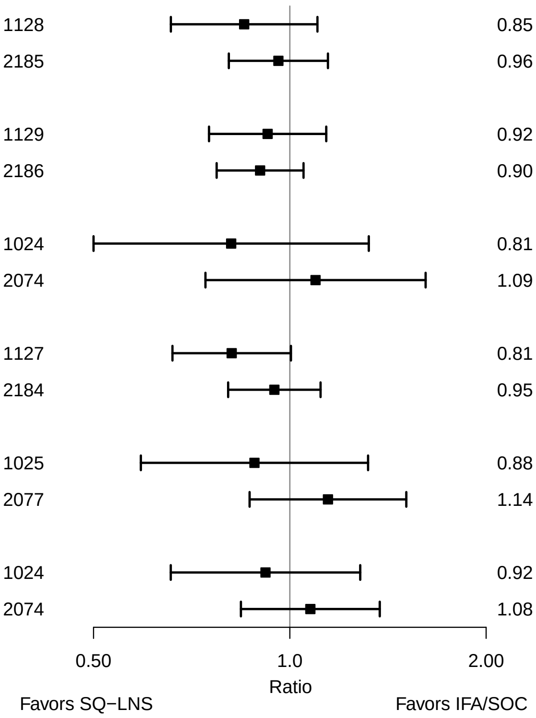

Supplemental figure 3F: Maternal education

3F1: Mean differences for birth outcomes

**Outcome (p−for−interaction)**	**SQ−LNS N**	**IFA/SOC N**	**Mean difference (95% CI)**
Birth weight (kg) (p=0.929)			
Primary or greater	1212	2328	0.05 (0.02, 0.07)
Incomplete or no formal	600	1126	0.05 (0.00, 0.10)
Weight−for−age z score (p=0.981)			
Primary or greater	1212	2328	0.12 (0.05, 0.18)
Incomplete or no formal	600	1126	0.11 (0.00, 0.22)
Weight−for−gestational age z−score (p=0.773)			
Primary or greater	468	446	0.17 (0.07, 0.27)
Incomplete or no formal	395	401	0.04 (−0.09, 0.17)
Birth length (cm) (p=0.690)			
Primary or greater	1194	2310	0.19 (0.07, 0.32)
Incomplete or no formal	484	1022	0.17 (−0.08, 0.42)
Length−for−age z−score (p=0.665)			
Primary or greater	1194	2310	0.11 (0.04, 0.18)
Incomplete or no formal	484	1022	0.10 (−0.03, 0.23)
Length−for−gestational age z−score (p=0.369)			
Primary or greater	450	429	0.15 (0.06, 0.24)
Incomplete or no formal	280	297	0.04 (−0.13, 0.20)
BMI z−score (p=0.638)			
Primary or greater	1193	2310	0.11 (0.04, 0.18)
Incomplete or no formal	481	1014	0.05 (−0.08, 0.18)
Head circumference (cm) (p=0.797)			
Primary or greater	1194	2311	0.09 (0.00, 0.17)
Incomplete or no formal	484	1023	0.12 (−0.04, 0.28)
Head circumference−for−age z−score (p=0.678)			
Primary or greater	1194	2311	0.08 (0.01, 0.15)
Incomplete or no formal	484	1023	0.12 (−0.01, 0.24)
Head circumference−for−gestational age z score (p=0.726)			
Primary or greater	450	429	0.11 (0.00, 0.23)
Incomplete or no formal	280	298	0.10 (−0.06, 0.25)
Mid−upper arm circumference (cm) (p=0.967)			
Primary or greater	985	2117	0.07 (0.00, 0.14)
Incomplete or no formal	474	1001	0.08 (−0.02, 0.18)
Duration (wk) (p=0.741)			
Primary or greater	1205	2320	0.08 (−0.06, 0.22)
Incomplete or no formal	645	1168	0.16 (−0.06, 0.39)

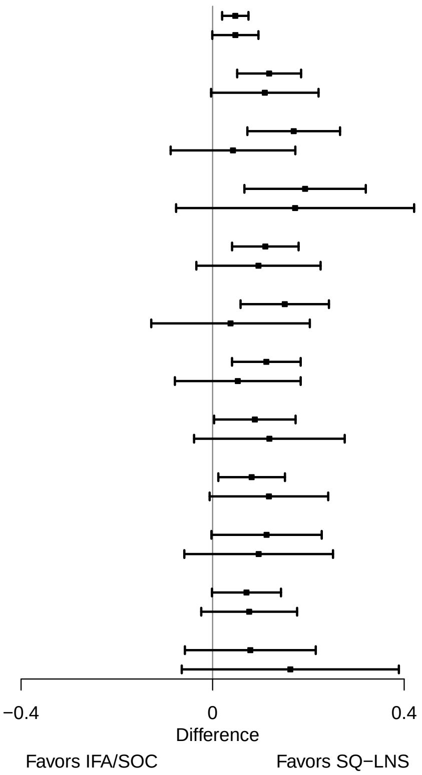

Supplemental figure 3F: Maternal education

3F2: Relative risks for birth outcomes

Outcome (p−for−interaction)	SQ−LNS N	IFA/SOC N	Relative risk (95% CI)
Low birth weight (p=0.216)			
Primary or greater	1212	2328	0.86 (0.76, 0.98)
Incomplete or no formal	600	1126	1.01 (0.85, 1.20)
Birth weight < 2 kg (p=0.607)			
Primary or greater	758	1892	0.78 (0.55, 1.10)
Incomplete or no formal	524	1041	0.91 (0.61, 1.36)
Small−for−gestational age (p=0.466)			
Primary or greater	1172	2286	0.97 (0.91, 1.03)
Incomplete or no formal	598	1118	0.94 (0.85, 1.03)
Large−for−gestational age (p=0.539)			
Primary or greater	1003	2134	0.92 (0.45, 1.88)
Incomplete or no formal	586	1104	1.06 (0.51, 2.17)
Newborn stunting (p=0.285)			
Primary or greater	1194	2310	0.80 (0.68, 0.93)
Incomplete or no formal	484	1022	0.94 (0.78, 1.13)
Low LGAZ (p=0.627)			
Primary or greater	985	2116	0.88 (0.76, 1.03)
Incomplete or no formal	470	1000	0.95 (0.76, 1.19)
Low BMIZ (p=0.089)			
Primary or greater	1193	2310	0.85 (0.76, 0.95)
Incomplete or no formal	481	1014	1.06 (0.88, 1.29)
Low HCZ (p=0.935)			
Primary or greater	1194	2311	0.86 (0.73, 1.01)
Incomplete or no formal	484	1023	0.85 (0.68, 1.07)
Low HCGAZ (p=0.372)			
Primary or greater	943	2079	0.84 (0.68, 1.03)
Incomplete or no formal	261	777	0.99 (0.75, 1.32)
Preterm birth (p=0.671)			
Primary or greater	1027	2158	1.00 (0.81, 1.23)
Incomplete or no formal	633	1153	0.90 (0.72, 1.12)

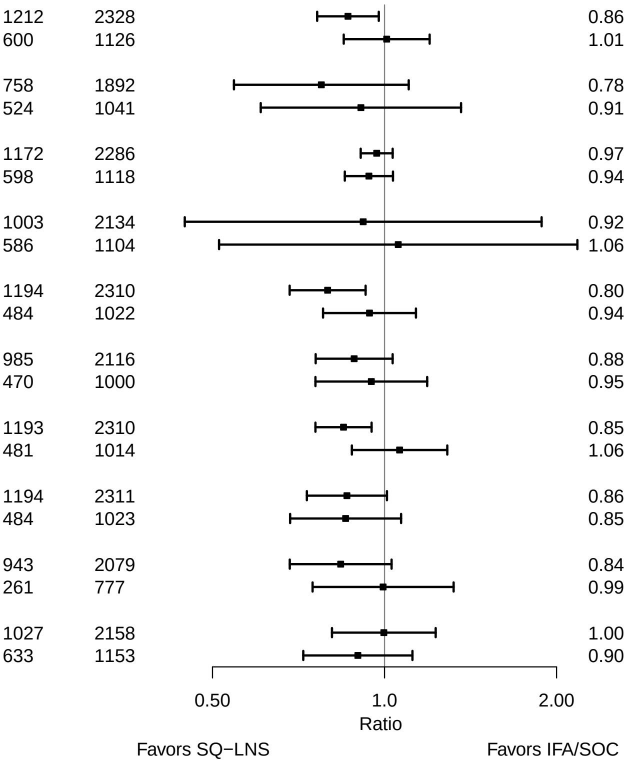

Supplemental figure 3F: Maternal education

3F3: Mean differences for 6 mo outcomes

Outcome (p−for−interaction)	SQ−LNS N	IFA/SOC N	Mean difference (95% CI)
Weight−for−age z score (p=0.559)			
Primary or greater	1162	2235	0.06 (−0.02, 0.14)
Incomplete or no formal	553	1077	−0.01 (−0.14, 0.12)
Length−for−age z−score (p=0.631)			
Primary or greater	1163	2236	0.05 (−0.03, 0.13)
Incomplete or no formal	553	1078	0.05 (−0.07, 0.17)
Weight−for−length z−score (p=0.085)			
Primary or greater	1162	2234	0.03 (−0.05, 0.10)
Incomplete or no formal	553	1077	−0.08 (−0.21, 0.05)
Head circumference−for−age z−score (p=0.637)			
Primary or greater	1163	2234	0.03 (−0.04, 0.09)
Incomplete or no formal	553	1076	−0.03 (−0.14, 0.08)
MUAC−for−age z−score (p=0.572)			
Primary or greater	957	2042	−0.01 (−0.10, 0.07)
Incomplete or no formal	540	1059	0.00 (−0.12, 0.12)

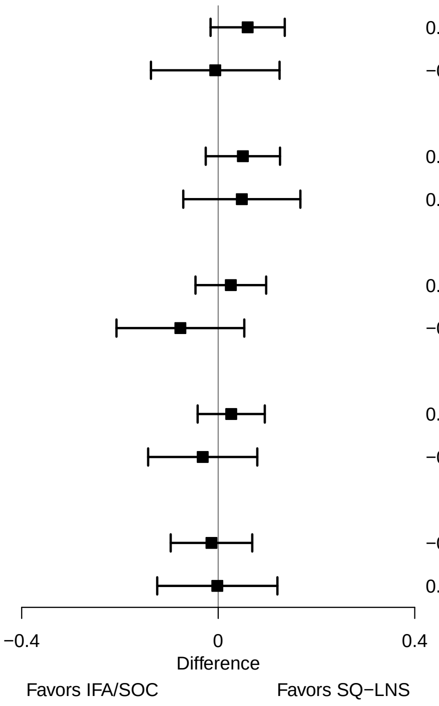

Supplemental figure 3F: Maternal education

3F4: Prevalence ratios for 6 mo outcomes

Outcome (p−for−interaction)	SQ−LNS N	IFA/SOC N	Prevalence ratio (95% CI)
Underweight (p=0.218)			
Primary or greater	1162	2235	0.80 (0.67, 0.94)
Incomplete or no formal	553	1077	1.09 (0.86, 1.39)
Stunted (p=0.845)			
Primary or greater	1163	2236	0.92 (0.79, 1.07)
Incomplete or no formal	553	1078	0.91 (0.77, 1.08)
Wasted (p=0.901)			
Primary or greater	956	2039	0.93 (0.64, 1.36)
Incomplete or no formal	540	1058	1.07 (0.66, 1.73)
Low HCAZ (p=0.897)			
Primary or greater	1163	2234	0.90 (0.79, 1.03)
Incomplete or no formal	553	1076	0.94 (0.75, 1.18)
Low MUAC (p=0.187)			
Primary or greater	907	1988	1.00 (0.75, 1.35)
Incomplete or no formal	241	749	1.45 (0.89, 2.36)
Acute malnutrition (p=0.175)			
Primary or greater	956	2039	0.95 (0.73, 1.25)
Incomplete or no formal	540	1058	1.12 (0.79, 1.59)

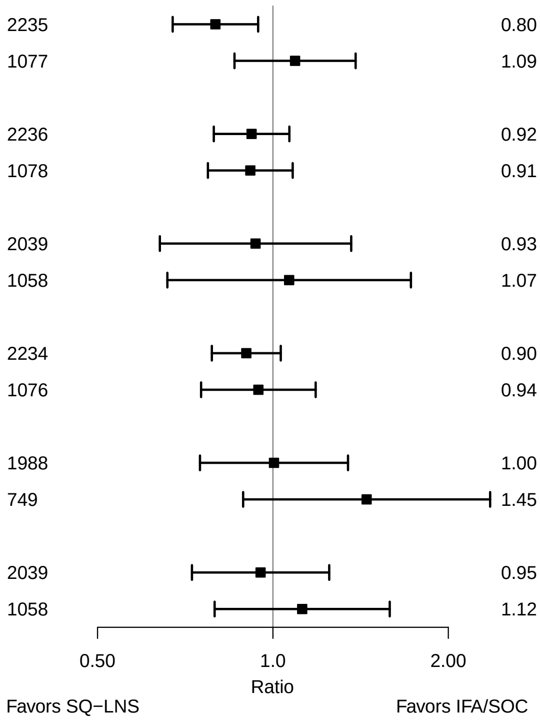

Supplemental figure 3G: Baseline anemia status

3G1: Mean differences for birth outcomes

**Outcome (p−for−interaction)**	**SQ−LNS N**	**IFA/SOC N**	**Mean difference (95% CI)**
Birth weight (kg) (p=0.633)			
Anemia	435	429	0.04 (−0.02, 0.10)
No anemia	796	704	0.05 (0.01, 0.09)
Weight−for−age z score (p=0.654)			
Anemia	435	429	0.10 (−0.04, 0.23)
No anemia	796	704	0.13 (0.03, 0.22)
Weight−for−gestational age z−score (p=0.653)			
Anemia	277	287	0.11 (−0.05, 0.27)
No anemia	408	398	0.07 (−0.07, 0.20)
Birth length (cm) (p=0.936)			
Anemia	374	362	0.20 (−0.11, 0.51)
No anemia	723	648	0.15 (−0.06, 0.37)
Length−for−age z−score (p=0.814)			
Anemia	374	362	0.12 (−0.05, 0.28)
No anemia	723	648	0.08 (−0.03, 0.19)
Length−for−gestational age z−score (p=0.606)			
Anemia	216	220	0.14 (−0.06, 0.34)
No anemia	335	342	0.07 (−0.08, 0.23)
BMI z−score (p=0.975)			
Anemia	371	358	0.14 (−0.01, 0.28)
No anemia	722	644	0.12 (0.01, 0.23)
Head circumference (cm) (p=0.877)			
Anemia	374	364	0.17 (−0.04, 0.38)
No anemia	722	647	0.09 (−0.05, 0.23)
Head circumference−for−age z−score (p=0.749)			
Anemia	374	364	0.15 (−0.01, 0.32)
No anemia	722	647	0.07 (−0.04, 0.18)
Head circumference−for−gestational age z score (p=0.605)			
Anemia	216	222	0.09 (−0.11, 0.29)
No anemia	335	341	0.15 (−0.01, 0.31)
Mid−upper arm circumference (cm) (p=0.881)			
Anemia	375	364	0.11 (−0.02, 0.25)
No anemia	725	647	0.07 (−0.01, 0.16)
Duration (wk) (p=0.173)			
Anemia	471	466	−0.07 (−0.38, 0.24)
No anemia	833	741	0.19 (−0.03, 0.42)

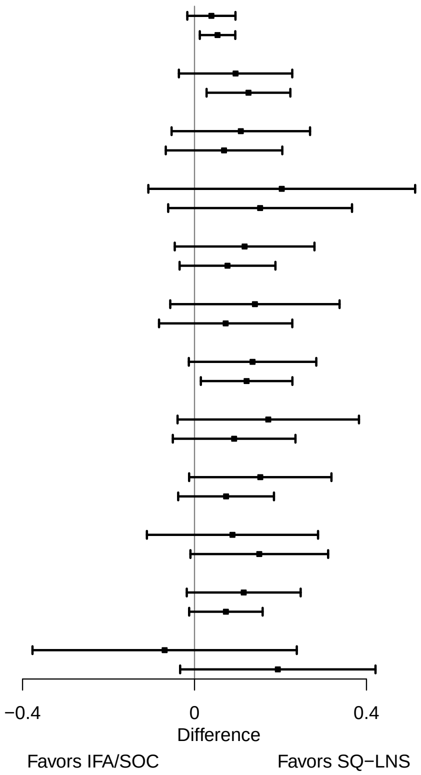

Supplemental figure 3G: Baseline anemia status

3G2: Relative risks for birth outcomes

Outcome (p−for−interaction)	SQ−LNS N	IFA/SOC N	Relative risk (95% CI)
Low birth weight (p=0.785)			
Anemia	435	429	0.89 (0.71, 1.11)
No anemia	796	704	0.84 (0.70, 1.01)
Birth weight < 2 kg (p=0.744)			
Anemia	435	429	1.06 (0.55, 2.05)
No anemia	796	704	0.84 (0.50, 1.42)
Small−for−gestational age (p=0.359)			
Anemia	435	429	0.88 (0.73, 1.07)
No anemia	796	704	0.98 (0.87, 1.12)
Large−for−gestational age (p=0.750)			
Anemia	282	289	0.82 (0.33, 2.06)
No anemia	412	402	0.98 (0.48, 1.98)
Newborn stunting (p=0.406)			
Anemia	374	362	0.96 (0.72, 1.29)
No anemia	723	648	0.83 (0.64, 1.07)
Low LGAZ (p=0.773)			
Anemia	374	362	0.94 (0.66, 1.36)
No anemia	723	648	0.87 (0.67, 1.13)
Low BMIZ (p=0.602)			
Anemia	371	358	0.85 (0.63, 1.15)
No anemia	722	644	0.77 (0.64, 0.92)
Low HCZ (p=0.436)			
Anemia	374	364	0.76 (0.56, 1.05)
No anemia	722	647	0.92 (0.72, 1.17)
Preterm birth (p=0.141)			
Anemia	471	466	1.20 (0.86, 1.66)
No anemia	833	741	0.90 (0.67, 1.21)

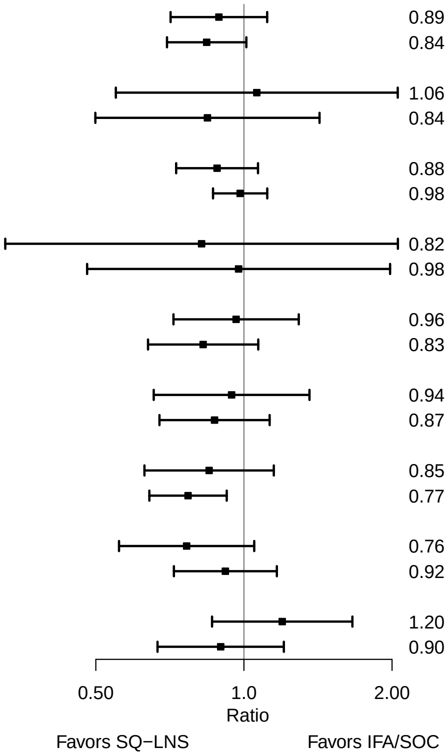

Supplemental figure 3G: Baseline anemia status

3G3: Mean differences for 6 mo outcomes

Outcome (p−for−interaction)	SQ−LNS N	IFA/SOC N	Mean difference (95% CI)
Weight−for−age z score (p=0.763)			
Anemia	405	413	0.04 (−0.11, 0.19)
No anemia	749	654	0.06 (−0.07, 0.19)
Length−for−age z−score (p=0.960)			
Anemia	406	413	0.05 (−0.10, 0.19)
No anemia	749	657	0.05 (−0.07, 0.18)
Weight−for−length z−score (p=0.850)			
Anemia	405	413	0.02 (−0.12, 0.16)
No anemia	749	654	0.03 (−0.08, 0.15)
Head circumference−for−age z−score (p=0.340)			
Anemia	406	412	−0.04 (−0.16, 0.09)
No anemia	749	656	0.06 (−0.04, 0.15)
MUAC−for−age z−score (p=0.939)			
Anemia	406	413	0.04 (−0.11, 0.18)
No anemia	749	657	0.04 (−0.06, 0.15)

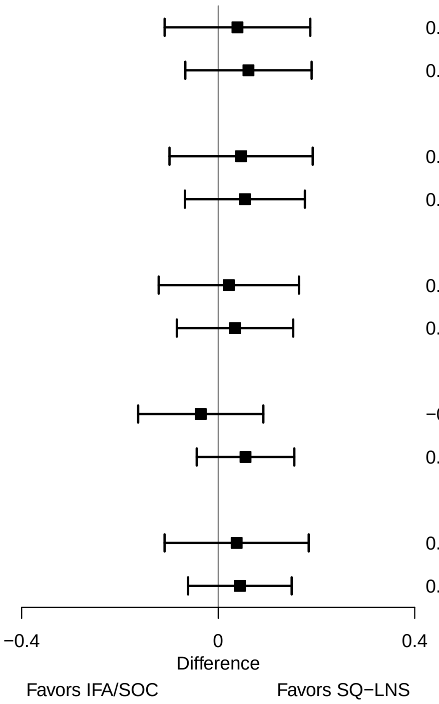

Supplemental figure 3G: Baseline anemia status

3G4: Prevalence ratios for 6 mo outcomes

Outcome (p−for−interaction)	SQ−LNS N	IFA/SOC N	Prevalence ratio (95% CI)
Underweight (p=0.292)			
Anemia	405	413	1.03 (0.69, 1.53)
No anemia	749	654	0.79 (0.59, 1.07)
Stunted (p=0.477)			
Anemia	406	413	1.00 (0.76, 1.31)
No anemia	749	657	0.86 (0.69, 1.08)
Wasted (p=0.215)			
Anemia	405	413	1.18 (0.52, 2.72)
No anemia	749	654	0.62 (0.36, 1.06)
Low HCAZ (p=0.454)			
Anemia	406	412	1.01 (0.71, 1.45)
No anemia	749	656	0.85 (0.67, 1.08)
Low MUAC (p=0.196)			
Anemia	406	413	1.15 (0.69, 1.91)
No anemia	749	657	0.76 (0.51, 1.12)
Acute malnutrition (p=0.060)			
Anemia	405	413	1.30 (0.80, 2.13)
No anemia	749	654	0.72 (0.51, 1.02)

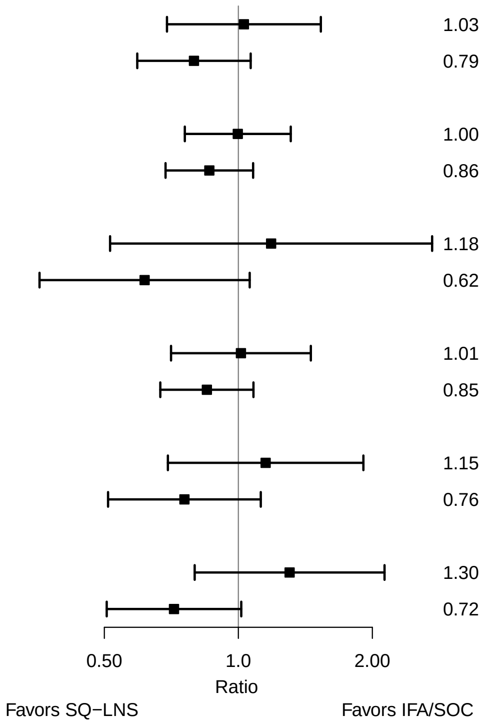

Supplemental figure 3H: Baseline inflammation status

3H1: Mean differences for birth outcomes

Outcome (p−for−interaction)	SQ−LNS N	IFA/SOC N	Mean difference (95% CI)
Birth weight (kg) (p=0.118)			
Not inflamed	816	772	0.03 (0.00, 0.07)
Inflamed	401	343	0.09 (0.03, 0.16)
Weight−for−age z score (p=0.091)			
Not inflamed	816	772	0.08 (−0.01, 0.17)
Inflamed	401	343	0.23 (0.08, 0.37)
Weight−for−gestational age z−score (p=0.327)			
Not inflamed	377	387	0.04 (−0.09, 0.18)
Inflamed	294	284	0.14 (−0.03, 0.30)
Birth length (cm) (p=0.308)			
Not inflamed	749	704	0.12 (−0.08, 0.31)
Inflamed	337	292	0.39 (0.06, 0.72)
Length−for−age z−score (p=0.280)			
Not inflamed	749	704	0.06 (−0.04, 0.16)
Inflamed	337	292	0.21 (0.03, 0.38)
Length−for−gestational age z−score (p=0.717)			
Not inflamed	310	319	0.07 (−0.09, 0.23)
Inflamed	230	233	0.12 (−0.07, 0.31)
BMI z−score (p=0.242)			
Not inflamed	746	702	0.09 (−0.01, 0.20)
Inflamed	336	286	0.20 (0.02, 0.37)
Head circumference (cm) (p=0.778)			
Not inflamed	747	705	0.13 (0.00, 0.27)
Inflamed	338	292	0.22 (0.00, 0.45)
Head circumference−for−age z−score (p=0.607)			
Not inflamed	747	705	0.11 (0.00, 0.21)
Inflamed	338	292	0.20 (0.01, 0.38)
Head circumference−for−gestational age z score (p=0.844)			
Not inflamed	309	320	0.15 (−0.01, 0.32)
Inflamed	231	233	0.12 (−0.07, 0.32)
Mid−upper arm circumference (cm) (p=0.189)			
Not inflamed	749	705	0.06 (−0.03, 0.14)
Inflamed	339	292	0.19 (0.04, 0.33)
Duration (wk) (p=0.090)			
Not inflamed	858	805	0.02 (−0.18, 0.22)
Inflamed	430	382	0.39 (0.06, 0.72)

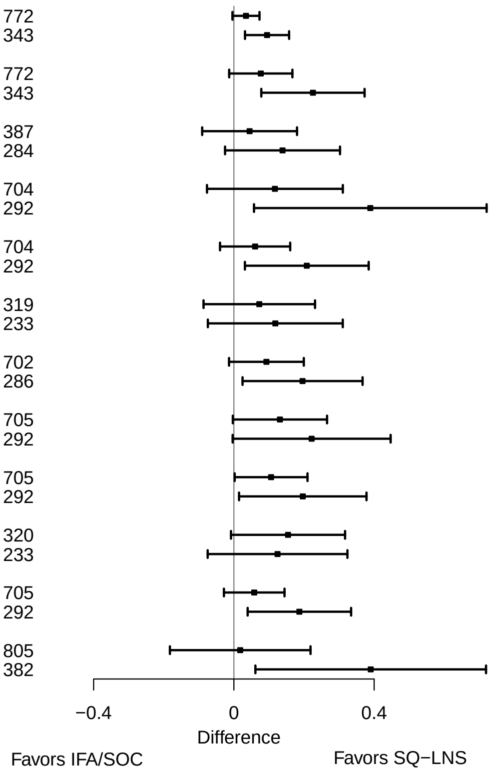

Supplemental figure 3H: Baseline inflammation status

3H2: Relative risks for birth outcomes

Outcome (p−for−interaction)	SQ−LNS N	IFA/SOC N	Relative risk (95% CI)
Low birth weight (p=0.140)			
Not inflamed	816	772	0.89 (0.76, 1.05)
Inflamed	401	343	0.67 (0.49, 0.92)
Birth weight < 2 kg (p=0.808)			
Not inflamed	816	772	0.91 (0.55, 1.50)
Inflamed	401	343	0.85 (0.36, 2.01)
Small−for−gestational age (p=0.963)			
Not inflamed	816	772	0.95 (0.85, 1.07)
Inflamed	401	343	0.94 (0.77, 1.15)
Large−for−gestational age (p=0.176)			
Not inflamed	384	390	0.65 (0.30, 1.41)
Inflamed	296	286	1.43 (0.62, 3.27)
Newborn stunting (p=0.189)			
Not inflamed	749	704	0.91 (0.73, 1.12)
Inflamed	337	292	0.69 (0.49, 0.97)
Low LGAZ (p=0.808)			
Not inflamed	749	704	0.90 (0.71, 1.14)
Inflamed	337	292	0.87 (0.59, 1.29)
Low BMIZ (p=0.324)			
Not inflamed	746	702	0.82 (0.69, 0.97)
Inflamed	336	286	0.67 (0.46, 0.98)
Low HCZ (p=0.095)			
Not inflamed	747	705	0.91 (0.74, 1.11)
Inflamed	338	292	0.60 (0.41, 0.89)
Low HCGAZ (p=0.700)			
Not inflamed	747	705	0.79 (0.59, 1.07)
Inflamed	338	292	0.91 (0.52, 1.59)
Preterm birth (p=0.284)			
Not inflamed	858	805	1.09 (0.82, 1.46)
Inflamed	430	382	0.80 (0.55, 1.15)

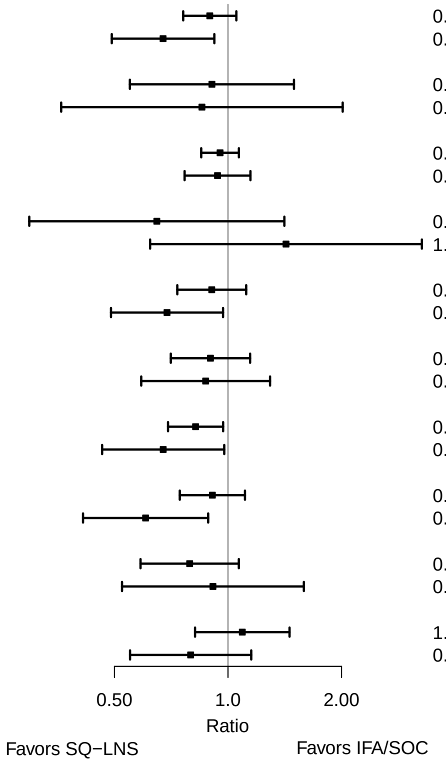

Supplemental figure 3H: Baseline inflammation status

3H3: Mean differences for 6 mo outcomes

Outcome (p−for−interaction)	SQ−LNS N	IFA/SOC N	Mean difference (95% CI)
Weight−for−age z score (p=0.341)			
Not inflamed	769	733	0.01 (−0.11, 0.14)
Inflamed	373	318	0.12 (−0.04, 0.29)
Length−for−age z−score (p=0.372)			
Not inflamed	770	735	0.03 (−0.08, 0.15)
Inflamed	373	319	0.13 (−0.03, 0.29)
Weight−for−length z−score (p=0.453)			
Not inflamed	769	733	0.00 (−0.12, 0.12)
Inflamed	373	318	0.07 (−0.10, 0.23)
Head circumference−for−age z−score (p=0.060)			
Not inflamed	770	734	−0.02 (−0.11, 0.08)
Inflamed	373	318	0.13 (−0.02, 0.28)
MUAC−for−age z−score (p=0.536)			
Not inflamed	770	735	0.01 (−0.10, 0.13)
Inflamed	373	319	0.08 (−0.07, 0.23)

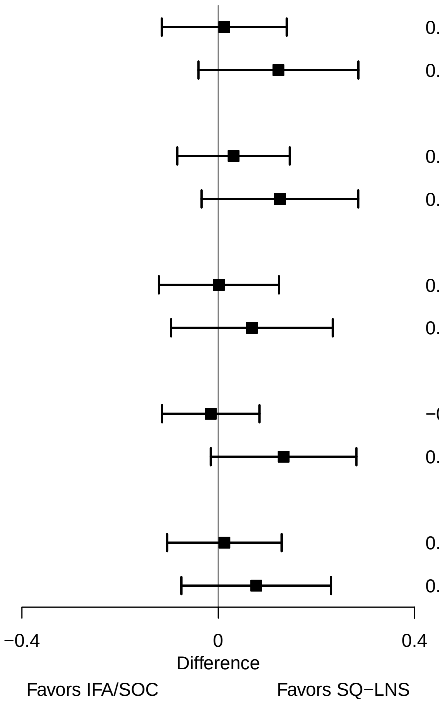

Supplemental figure 3H: Baseline inflammation status

3H4: Prevalence ratios for 6 mo outcomes

Outcome (p−for−interaction)	SQ−LNS N	IFA/SOC N	Prevalence ratio (95% CI)
Underweight (p=0.708)			
Not inflamed	769	733	0.85 (0.62, 1.15)
Inflamed	373	318	0.79 (0.49, 1.27)
Stunted (p=0.245)			
Not inflamed	770	735	0.93 (0.75, 1.15)
Inflamed	373	319	0.78 (0.58, 1.05)
Wasted (p=0.569)			
Not inflamed	769	733	0.79 (0.44, 1.42)
Inflamed	373	318	0.70 (0.29, 1.69)
Low HCAZ (p=0.184)			
Not inflamed	770	734	0.94 (0.73, 1.21)
Inflamed	373	318	0.72 (0.50, 1.02)
Low MUAC (p=0.753)			
Not inflamed	770	735	0.89 (0.61, 1.30)
Inflamed	373	319	0.93 (0.48, 1.79)
Acute malnutrition (p=0.851)			
Not inflamed	769	733	0.90 (0.63, 1.29)
Inflamed	373	318	0.86 (0.48, 1.52)

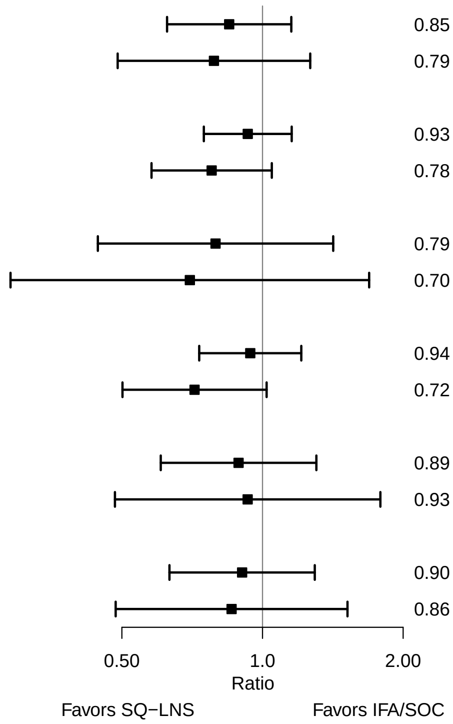

Supplemental figure 3I: Baseline malaria status

3I1: Mean differences for birth outcomes

Outcome (p−for−interaction)	SQ−LNS N	IFA/SOC N	Mean difference (95% CI)
Birth weight (kg) (p=0.053)			
No malaria	574	582	0.05 (−0.01, 0.10)
Malaria	118	109	0.16 (0.06, 0.27)
Weight−for−age z score (p=0.024)			
No malaria	574	582	0.09 (−0.02, 0.21)
Malaria	118	109	0.41 (0.16, 0.66)
Weight−for−gestational age z−score (p=0.268)			
No malaria	567	576	0.07 (−0.04, 0.19)
Malaria	116	109	0.22 (−0.01, 0.45)
Birth length (cm) (p=0.989)			
No malaria	470	485	0.28 (0.03, 0.53)
Malaria	89	83	0.27 (−0.35, 0.90)
Length−for−age z−score (p=0.794)			
No malaria	470	485	0.14 (0.01, 0.27)
Malaria	89	83	0.19 (−0.15, 0.52)
Length−for−gestational age z−score (p=0.715)			
No malaria	463	479	0.11 (−0.02, 0.24)
Malaria	87	83	0.08 (−0.22, 0.38)
BMI z−score (p=0.235)			
No malaria	467	481	0.10 (−0.04, 0.24)
Malaria	88	79	0.32 (0.02, 0.62)
Head circumference (cm) (p=0.977)			
No malaria	471	486	0.21 (0.03, 0.39)
Malaria	88	83	0.17 (−0.26, 0.59)
Head circumference−for−age z−score (p=0.703)			
No malaria	471	486	0.16 (0.02, 0.30)
Malaria	88	83	0.19 (−0.16, 0.55)
Head circumference−for−gestational age z score (p=0.969)			
No malaria	464	480	0.13 (0.00, 0.27)
Malaria	86	83	0.10 (−0.23, 0.42)
Mid−upper arm circumference (cm) (p=0.269)			
No malaria	473	486	0.11 (0.00, 0.23)
Malaria	89	83	0.27 (0.00, 0.54)
Duration (wk) (p=0.761)			
No malaria	629	638	0.14 (−0.12, 0.40)
Malaria	136	127	0.18 (−0.45, 0.82)

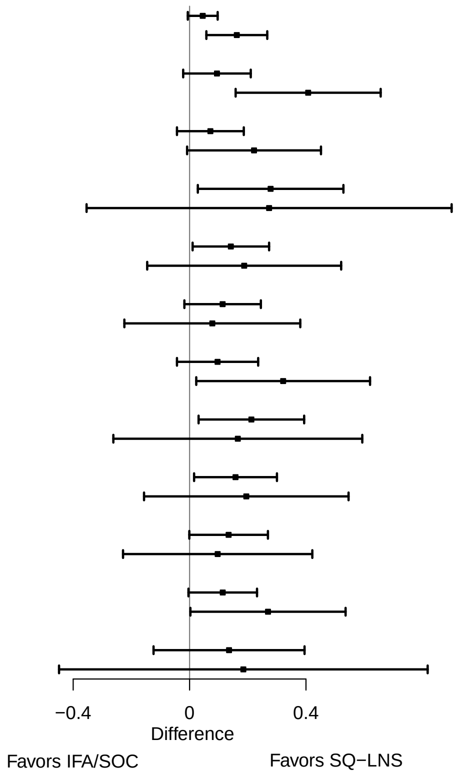

Supplemental figure 3I: Baseline malaria status

3I2: Relative risks for birth outcomes

Outcome (p−for−interaction)	SQ−LNS N	IFA/SOC N	Relative risk (95% CI)
Low birth weight (p=0.097)			
No malaria	574	582	0.88 (0.64, 1.21)
Malaria	118	109	0.53 (0.28, 0.98)
Small−for−gestational age (p=0.016)			
No malaria	574	582	1.01 (0.81, 1.26)
Malaria	118	109	0.60 (0.41, 0.89)
Newborn stunting (p=0.673)			
No malaria	470	485	0.88 (0.61, 1.28)
Malaria	89	83	0.96 (0.56, 1.64)
Low LGAZ (p=0.780)			
No malaria	470	485	0.96 (0.64, 1.44)
Malaria	89	83	0.98 (0.55, 1.76)
Low HCZ (p=0.254)			
No malaria	471	486	0.93 (0.57, 1.52)
Malaria	88	83	0.40 (0.10, 1.61)
Preterm birth (p=0.271)			
No malaria	629	638	0.98 (0.71, 1.36)
Malaria	136	127	0.61 (0.30, 1.25)

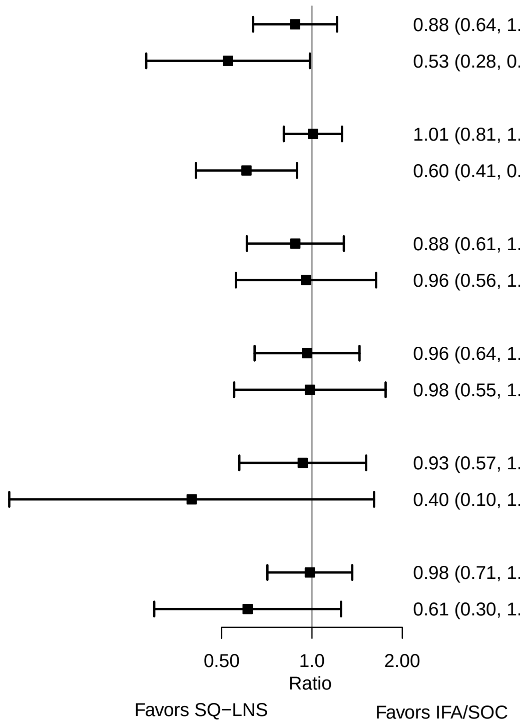

Supplemental figure 3I: Baseline malaria status

3I3: Mean differences for 6 mo outcomes

Outcome (p−for−interaction)	SQ−LNS N	IFA/SOC N	Mean difference (95% CI)
Weight−for−age z score (p=0.092)			
No malaria	523	534	−0.01 (−0.14, 0.13)
Malaria	106	102	0.27 (−0.02, 0.57)
Length−for−age z−score (p=0.133)			
No malaria	524	537	0.02 (−0.11, 0.15)
Malaria	106	102	0.24 (−0.04, 0.53)
Weight−for−length z−score (p=0.379)			
No malaria	523	534	−0.02 (−0.16, 0.12)
Malaria	106	102	0.16 (−0.15, 0.47)
Head circumference−for−age z−score (p=0.210)			
No malaria	524	535	−0.01 (−0.13, 0.10)
Malaria	106	102	0.14 (−0.13, 0.40)
MUAC−for−age z−score (p=0.540)			
No malaria	524	537	0.02 (−0.11, 0.15)
Malaria	106	102	0.13 (−0.13, 0.40)

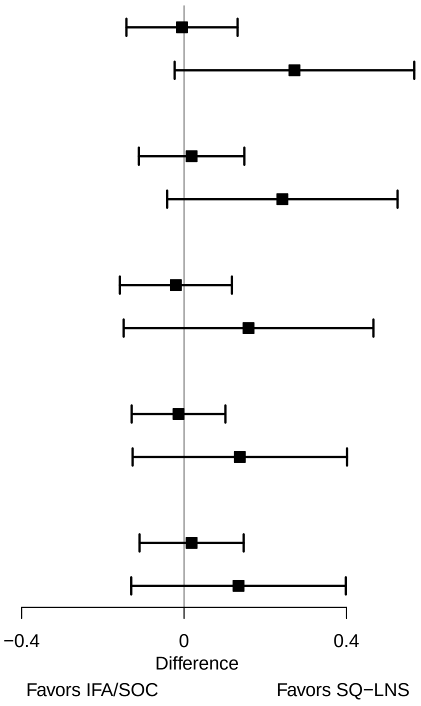

Supplemental figure 3I: Baseline malaria status

3I4: Prevalence ratios for 6 mo outcomes

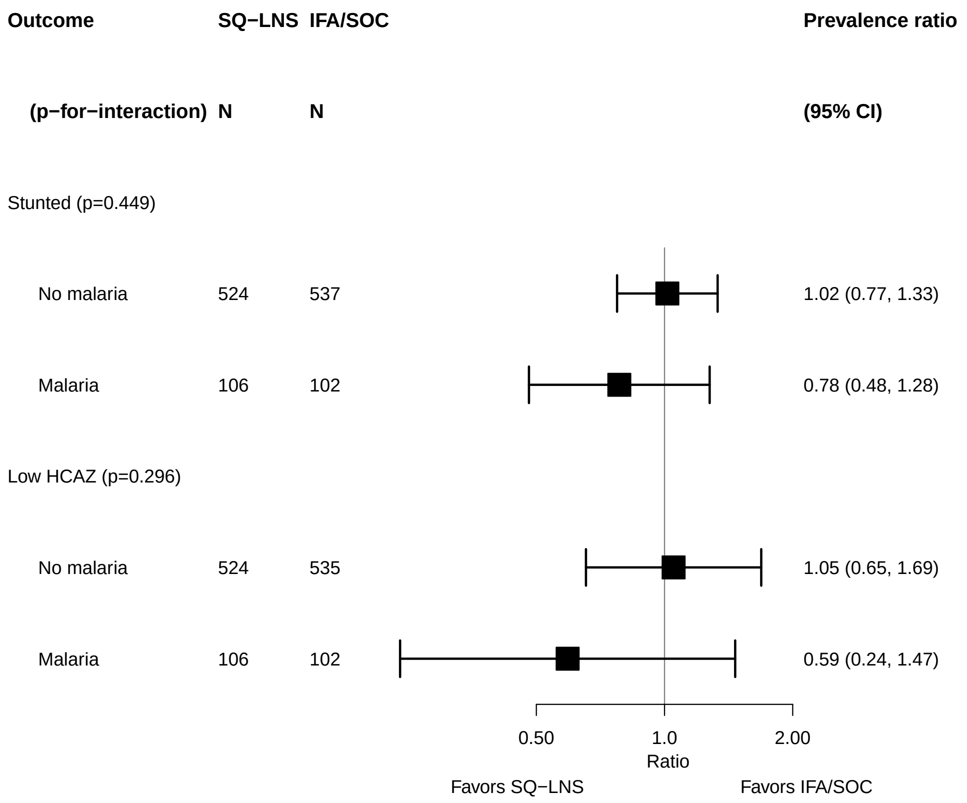

Supplemental figure 3J: Gestational age at supplementation

3J1: Mean differences for birth outcomes

Outcome (p−for−interaction)	SQ−LNS N	IFA/SOC N	Mean difference (95% CI)
Birth weight (kg) (p=0.283)			
< 14 wks	851	1847	0.04 (0.01, 0.07)
>= 14 wks	949	1586	0.06 (0.03, 0.10)
Weight−for−age z score (p=0.316)			
< 14 wks	851	1847	0.10 (0.03, 0.17)
>= 14 wks	949	1586	0.15 (0.07, 0.23)
Weight−for−gestational age z−score (p=0.900)			
< 14 wks	115	117	0.13 (−0.12, 0.37)
>= 14 wks	570	568	0.08 (−0.03, 0.20)
Birth length (cm) (p=0.226)			
< 14 wks	836	1840	0.13 (0.00, 0.27)
>= 14 wks	829	1469	0.30 (0.11, 0.49)
Length−for−age z−score (p=0.182)			
< 14 wks	836	1840	0.08 (0.00, 0.15)
>= 14 wks	829	1469	0.16 (0.06, 0.26)
Length−for−gestational age z−score (p=0.787)			
< 14 wks	100	111	0.13 (−0.15, 0.41)
>= 14 wks	451	451	0.09 (−0.05, 0.22)
BMI z−score (p=0.620)			
< 14 wks	836	1838	0.09 (0.01, 0.18)
>= 14 wks	825	1463	0.12 (0.03, 0.21)
Head circumference (cm) (p=0.213)			
< 14 wks	835	1841	0.06 (−0.04, 0.15)
>= 14 wks	830	1470	0.20 (0.07, 0.33)
Head circumference−for−age z−score (p=0.192)			
< 14 wks	835	1841	0.06 (−0.02, 0.13)
>= 14 wks	830	1470	0.17 (0.06, 0.27)
Head circumference−for−gestational age z score (p=0.773)			
< 14 wks	99	111	0.09 (−0.20, 0.39)
>= 14 wks	452	452	0.13 (−0.01, 0.27)
Mid−upper arm circumference (cm) (p=0.262)			
< 14 wks	655	1673	0.04 (−0.04, 0.11)
>= 14 wks	806	1447	0.13 (0.04, 0.21)
Duration (wk) (p=0.308)			
< 14 wks	685	1689	0.07 (−0.10, 0.24)
>= 14 wks	980	1627	0.21 (0.01, 0.41)

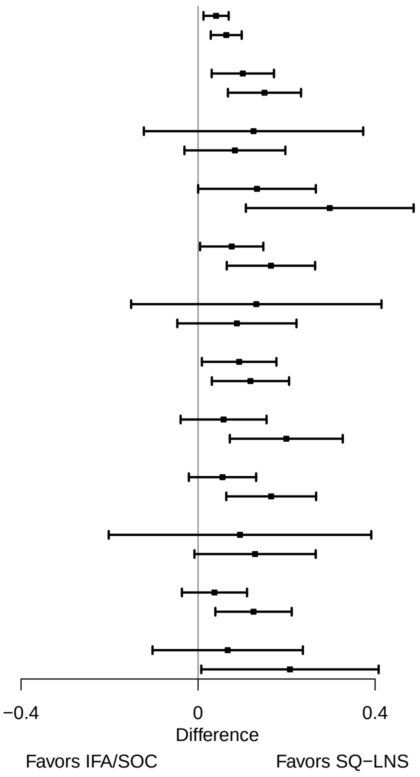

Supplemental figure 3J: Gestational age at supplementation

3J2: Relative risks for birth outcomes

Outcome (p−for−interaction)	SQ−LNS N	IFA/SOC N	Relative risk (95% CI)
Low birth weight (p=0.802)			
< 14 wks	851	1847	0.90 (0.80, 1.00)
>= 14 wks	949	1586	0.89 (0.76, 1.05)
Birth weight < 2 kg (p=0.860)			
< 14 wks	602	1606	0.77 (0.55, 1.09)
>= 14 wks	683	1331	0.86 (0.55, 1.36)
Small−for−gestational age (p=0.993)			
< 14 wks	670	1679	0.97 (0.90, 1.05)
>= 14 wks	922	1563	0.96 (0.88, 1.04)
Large−for−gestational age (p=0.060)			
< 14 wks	618	1632	0.57 (0.17, 1.93)
>= 14 wks	587	1224	1.60 (0.70, 3.69)
Newborn stunting (p=0.485)			
< 14 wks	836	1840	0.86 (0.75, 0.97)
>= 14 wks	829	1469	0.81 (0.65, 1.01)
Low LGAZ (p=0.811)			
< 14 wks	655	1672	0.90 (0.78, 1.04)
>= 14 wks	802	1446	0.90 (0.74, 1.10)
Low BMIZ (p=0.806)			
< 14 wks	836	1838	0.89 (0.78, 1.02)
>= 14 wks	825	1463	0.91 (0.80, 1.03)
Low HCZ (p=0.923)			
< 14 wks	835	1841	0.86 (0.72, 1.04)
>= 14 wks	830	1470	0.83 (0.67, 1.05)
Low HCGAZ (p=0.505)			
< 14 wks	618	1632	0.83 (0.65, 1.07)
>= 14 wks	586	1224	0.94 (0.73, 1.20)
Preterm birth (p=0.442)			
< 14 wks	685	1689	0.88 (0.70, 1.11)
>= 14 wks	980	1627	0.97 (0.77, 1.23)

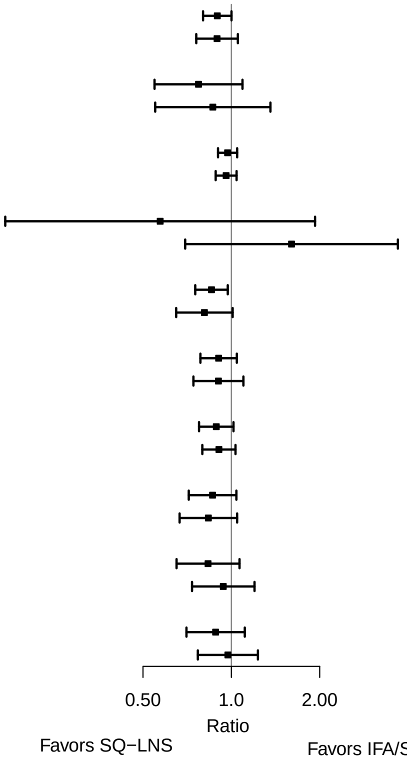

Supplemental figure 3J: Gestational age at supplementation

3J3: Mean differences for 6 mo outcomes

Outcome (p−for−interaction)	SQ−LNS N	IFA/SOC N	Mean difference (95% CI)
Weight−for−age z score (p=0.756)			
< 14 wks	830	1790	0.04 (−0.04, 0.13)
>= 14 wks	867	1492	0.03 (−0.08, 0.14)
Length−for−age z−score (p=0.780)			
< 14 wks	830	1790	0.07 (−0.02, 0.16)
>= 14 wks	868	1494	0.05 (−0.05, 0.15)
Weight−for−length z−score (p=0.712)			
< 14 wks	830	1790	−0.01 (−0.10, 0.07)
>= 14 wks	867	1491	0.00 (−0.11, 0.11)
Head circumference−for−age z−score (p=0.513)			
< 14 wks	830	1787	−0.01 (−0.09, 0.07)
>= 14 wks	868	1493	0.02 (−0.07, 0.11)
MUAC−for−age z−score (p=0.939)			
< 14 wks	653	1625	−0.02 (−0.11, 0.08)
>= 14 wks	840	1471	0.01 (−0.09, 0.11)

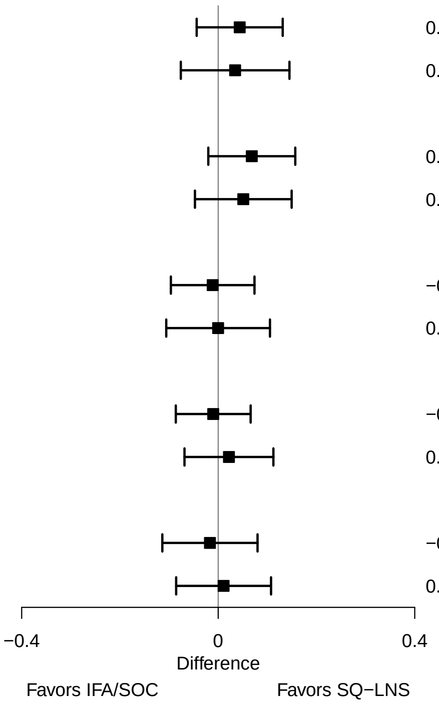

Supplemental figure 3J: Gestational age at supplementation

3J4: Prevalence ratios for 6 mo outcomes

Outcome (p−for−interaction)	SQ−LNS N	IFA/SOC N	Prevalence ratio (95% CI)
Underweight (p=0.983)			
< 14 wks	830	1790	0.90 (0.76, 1.07)
>= 14 wks	867	1492	0.96 (0.72, 1.26)
Stunted (p=0.131)			
< 14 wks	830	1790	0.83 (0.72, 0.96)
>= 14 wks	868	1494	1.02 (0.84, 1.25)
Wasted (p=0.731)			
< 14 wks	605	1580	1.03 (0.68, 1.55)
>= 14 wks	537	1148	0.75 (0.44, 1.29)
Low HCAZ (p=0.911)			
< 14 wks	782	1743	0.89 (0.75, 1.05)
>= 14 wks	566	1174	0.90 (0.73, 1.12)
Low MUAC (p=0.010)			
< 14 wks	653	1625	1.36 (0.97, 1.91)
>= 14 wks	840	1471	0.76 (0.55, 1.07)
Acute malnutrition (p=0.094)			
< 14 wks	653	1625	1.20 (0.88, 1.63)
>= 14 wks	839	1467	0.83 (0.61, 1.13)

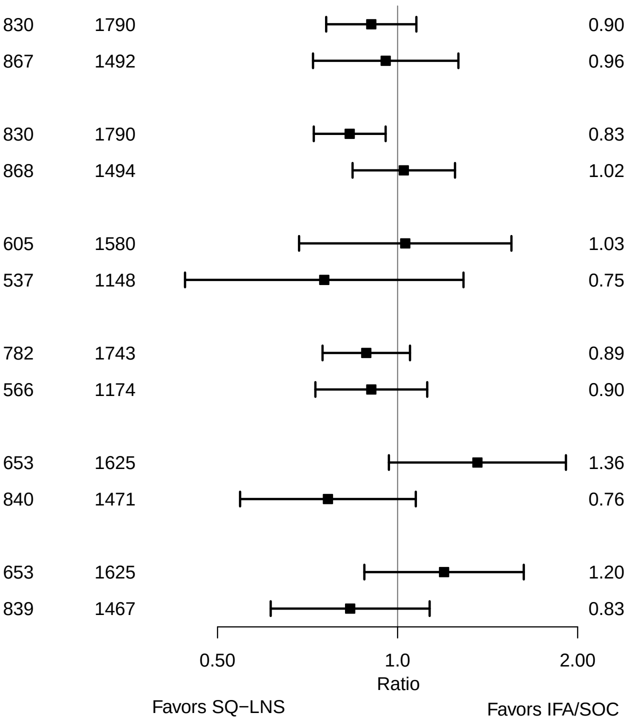

Supplemental figure 3K: Compliance with supplementation

3K1: Mean differences for birth outcomes

Outcome (p−for−interaction)	SQ−LNS N	IFA/SOC N	Mean difference (95% CI)
Birth weight (kg) (p=0.105)			
At least 4/7 d	1184	2961	0.07 (0.04, 0.10)
Less than 4/7 d	400	249	0.00 (−0.07, 0.07)
Weight−for−age z score (p=0.123)			
At least 4/7 d	1184	2961	0.16 (0.08, 0.23)
Less than 4/7 d	400	249	0.01 (−0.17, 0.19)
Weight−for−gestational age z−score (p=0.949)			
At least 4/7 d	602	626	0.09 (−0.02, 0.20)
Less than 4/7 d	80	56	0.13 (−0.23, 0.48)
Birth length (cm) (p=0.113)			
At least 4/7 d	1063	2847	0.27 (0.13, 0.42)
Less than 4/7 d	389	241	−0.04 (−0.38, 0.30)
Length−for−age z−score (p=0.115)			
At least 4/7 d	1063	2847	0.15 (0.07, 0.23)
Less than 4/7 d	389	241	−0.02 (−0.20, 0.16)
Length−for−gestational age z−score (p=0.296)			
At least 4/7 d	482	513	0.12 (−0.01, 0.25)
Less than 4/7 d	69	48	−0.10 (−0.47, 0.28)
BMI z−score (p=0.590)			
At least 4/7 d	1059	2840	0.12 (0.04, 0.20)
Less than 4/7 d	389	240	0.07 (−0.11, 0.25)
Head circumference (cm) (p=0.534)			
At least 4/7 d	1063	2849	0.17 (0.07, 0.28)
Less than 4/7 d	389	241	0.06 (−0.17, 0.30)
Head circumference−for−age z−score (p=0.466)			
At least 4/7 d	1063	2849	0.15 (0.06, 0.23)
Less than 4/7 d	389	241	0.05 (−0.13, 0.23)
Head circumference−for−gestational age z score (p=0.860)			
At least 4/7 d	482	514	0.13 (0.00, 0.26)
Less than 4/7 d	69	48	0.17 (−0.28, 0.62)
Mid−upper arm circumference (cm) (p=0.508)			
At least 4/7 d	1067	2849	0.11 (0.03, 0.18)
Less than 4/7 d	389	241	0.04 (−0.10, 0.19)
Duration (wk) (p=0.903)			
At least 4/7 d	1235	3019	0.12 (−0.05, 0.30)
Less than 4/7 d	421	264	0.12 (−0.22, 0.46)

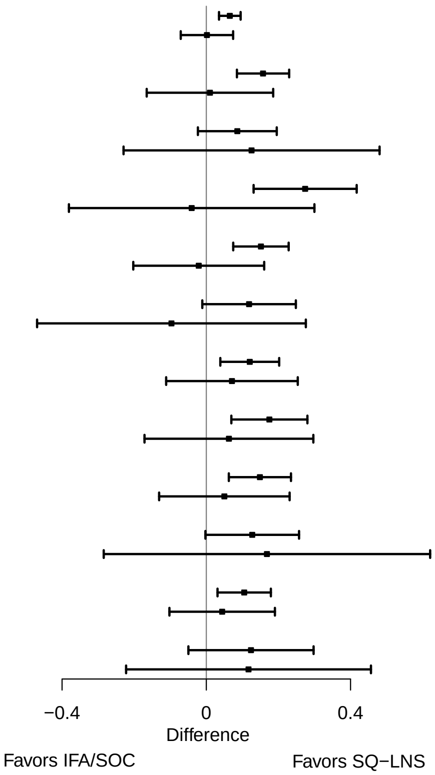

Supplemental figure 3K: Compliance with supplementation

3K2: Relative risks for birth outcomes

**Outcome (p−for−interaction)**	**SQ−LNS N**	**IFA/SOC N**	**Relative risk (95% CI)**
Low birth weight (p=0.364)			
At least 4/7 d	1184	2961	0.84 (0.74, 0.96)
Less than 4/7 d	400	249	0.96 (0.75, 1.23)
Small−for−gestational age (p=0.540)			
At least 4/7 d	1184	2961	0.94 (0.87, 1.01)
Less than 4/7 d	400	249	1.00 (0.85, 1.18)
Newborn stunting (p=0.081)			
At least 4/7 d	1063	2847	0.78 (0.66, 0.92)
Less than 4/7 d	389	241	1.08 (0.79, 1.48)
Low LGAZ (p=0.743)			
At least 4/7 d	1063	2847	0.85 (0.72, 1.01)
Less than 4/7 d	389	241	0.90 (0.63, 1.27)
Low BMIZ (p=0.565)			
At least 4/7 d	1059	2840	0.86 (0.76, 0.97)
Less than 4/7 d	389	240	0.91 (0.73, 1.15)
Low HCZ (p=0.909)			
At least 4/7 d	1063	2849	0.82 (0.69, 0.99)
Less than 4/7 d	389	241	0.84 (0.65, 1.11)
Low HCGAZ (p=0.613)			
At least 4/7 d	821	2601	0.84 (0.67, 1.07)
Less than 4/7 d	378	226	0.76 (0.54, 1.06)
Preterm birth (p=0.845)			
At least 4/7 d	1235	3019	0.93 (0.75, 1.15)
Less than 4/7 d	421	264	0.98 (0.67, 1.43)

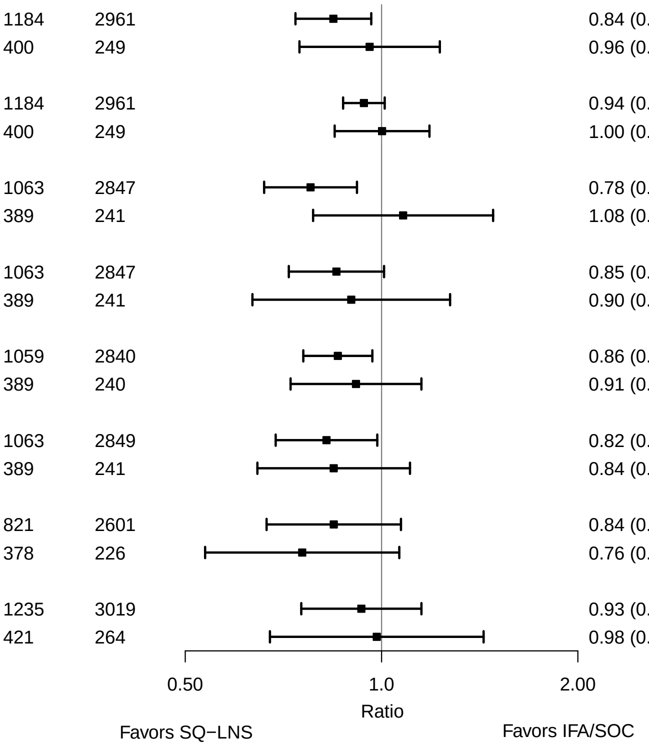

Supplemental figure 3K: Compliance with supplementation

3K3: Mean differences for 6 mo outcomes

Outcome (p−for−interaction)	SQ−LNS N	IFA/SOC N	Mean difference (95% CI)
Weight−for−age z score (p=0.937)			
At least 4/7 d	1111	2830	0.01 (−0.07, 0.09)
Less than 4/7 d	378	244	0.01 (−0.19, 0.21)
Length−for−age z−score (p=0.147)			
At least 4/7 d	1112	2833	0.07 (−0.01, 0.15)
Less than 4/7 d	378	243	−0.05 (−0.22, 0.11)
Weight−for−length z−score (p=0.180)			
At least 4/7 d	1111	2830	−0.06 (−0.13, 0.02)
Less than 4/7 d	378	243	0.04 (−0.14, 0.23)
Head circumference−for−age z−score (p=0.291)			
At least 4/7 d	1112	2831	0.03 (−0.04, 0.10)
Less than 4/7 d	378	244	−0.05 (−0.19, 0.10)
MUAC−for−age z−score (p=0.916)			
At least 4/7 d	1112	2833	0.00 (−0.08, 0.07)
Less than 4/7 d	378	244	−0.02 (−0.20, 0.16)

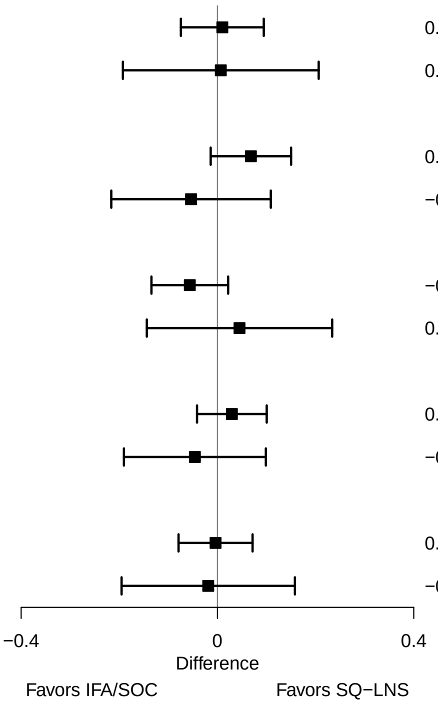

Supplemental figure 3K: Compliance with supplementation

3K4: Prevalence ratios for 6 mo outcomes

Outcome (p−for−interaction)	SQ−LNS N	IFA/SOC N	Prevalence ratio (95% CI)
Underweight (p=0.635)			
At least 4/7 d	1111	2830	0.99 (0.83, 1.19)
Less than 4/7 d	378	244	0.89 (0.57, 1.40)
Stunted (p=0.190)			
At least 4/7 d	1112	2833	0.90 (0.76, 1.06)
Less than 4/7 d	378	243	1.10 (0.80, 1.51)
Low HCAZ (p=0.370)			
At least 4/7 d	1112	2831	0.86 (0.74, 0.99)
Less than 4/7 d	378	244	0.99 (0.73, 1.34)
Low MUAC (p=0.512)			
At least 4/7 d	1112	2833	1.04 (0.80, 1.36)
Less than 4/7 d	378	244	0.92 (0.49, 1.76)
Acute malnutrition (p=0.166)			
At least 4/7 d	1111	2830	1.07 (0.85, 1.33)
Less than 4/7 d	378	243	0.73 (0.41, 1.32)

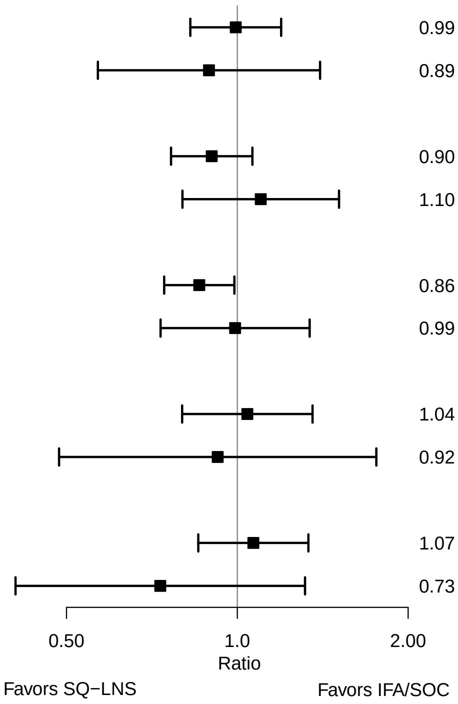

Supplemental figure 3L: Household socio-economic status

3L1: Mean differences for birth outcomes

Outcome (p−for−interaction)	SQ−LNS N	IFA/SOC N	Mean difference (95% CI)
Birth weight (kg) (p=0.909)			
At least median	874	1681	0.05 (0.02, 0.08)
Less than median	914	1739	0.05 (0.01, 0.09)
Weight−for−age z score (p=0.933)			
At least median	874	1681	0.12 (0.04, 0.20)
Less than median	914	1739	0.12 (0.03, 0.21)
Weight−for−gestational age z−score (p=0.280)			
At least median	383	389	0.18 (0.05, 0.30)
Less than median	463	431	0.05 (−0.08, 0.17)
Birth length (cm) (p=0.829)			
At least median	816	1626	0.19 (0.03, 0.36)
Less than median	838	1673	0.19 (0.00, 0.38)
Length−for−age z−score (p=0.951)			
At least median	816	1626	0.11 (0.02, 0.19)
Less than median	838	1673	0.12 (0.01, 0.22)
Length−for−gestational age z−score (p=0.347)			
At least median	326	335	0.18 (0.04, 0.31)
Less than median	387	365	0.06 (−0.07, 0.19)
BMI z−score (p=0.952)			
At least median	814	1625	0.09 (0.00, 0.17)
Less than median	836	1666	0.10 (0.00, 0.19)
Head circumference (cm) (p=0.565)			
At least median	814	1628	0.12 (0.02, 0.22)
Less than median	840	1673	0.11 (−0.03, 0.24)
Head circumference−for−age z−score (p=0.706)			
At least median	814	1628	0.12 (0.04, 0.19)
Less than median	840	1673	0.11 (0.00, 0.21)
Head circumference−for−gestational age z score (p=0.143)			
At least median	324	336	0.21 (0.08, 0.35)
Less than median	389	365	0.01 (−0.13, 0.15)
Mid−upper arm circumference (cm) (p=0.560)			
At least median	717	1540	0.06 (−0.02, 0.14)
Less than median	742	1576	0.10 (0.01, 0.18)
Duration (wk) (p=0.345)			
At least median	885	1708	0.16 (0.01, 0.31)
Less than median	940	1746	0.07 (−0.10, 0.24)

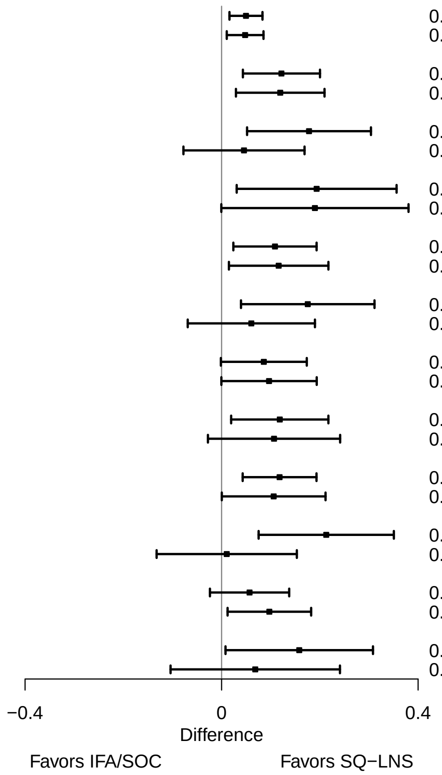

Supplemental figure 3L: Household socio-economic status

3L2: Relative risks for birth outcomes

Outcome (p−for−interaction)	SQ−LNS N	IFA/SOC N	Relative risk (95% CI)
Low birth weight (p=0.895)			
At least median	874	1681	0.92 (0.79, 1.07)
Less than median	914	1739	0.90 (0.78, 1.03)
Birth weight < 2 kg (p=0.161)			
At least median	774	1593	0.98 (0.68, 1.42)
Less than median	815	1642	0.64 (0.42, 0.98)
Small−for−gestational age (p=0.012)			
At least median	854	1666	1.04 (0.96, 1.12)
Less than median	899	1711	0.90 (0.83, 0.97)
Large−for−gestational age (p=0.669)			
At least median	774	1593	0.89 (0.44, 1.78)
Less than median	815	1642	1.10 (0.53, 2.29)
Newborn stunting (p=0.789)			
At least median	816	1626	0.85 (0.70, 1.02)
Less than median	838	1673	0.85 (0.71, 1.03)
Low LGAZ (p=0.763)			
At least median	796	1611	0.88 (0.73, 1.06)
Less than median	823	1645	0.93 (0.77, 1.12)
Low BMIZ (p=0.882)			
At least median	814	1625	0.91 (0.78, 1.06)
Less than median	836	1666	0.90 (0.77, 1.04)
Low HCZ (p=0.641)			
At least median	814	1628	0.89 (0.76, 1.04)
Less than median	840	1673	0.83 (0.69, 1.01)
Low HCGAZ (p=0.018)			
At least median	548	1348	1.08 (0.89, 1.32)
Less than median	513	1345	0.74 (0.59, 0.93)
Preterm birth (p=0.759)			
At least median	885	1708	0.96 (0.76, 1.23)
Less than median	940	1746	0.92 (0.74, 1.14)

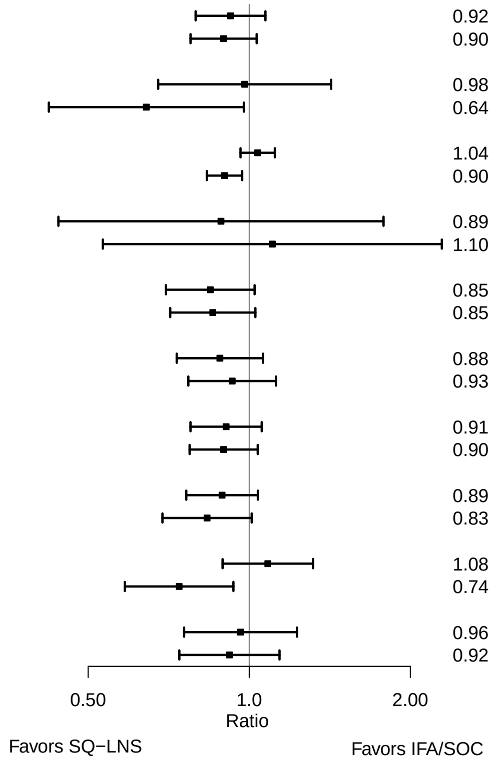

Supplemental figure 3L: Household socio-economic status

3L3: Mean differences for 6 mo outcomes

Outcome (p−for−interaction)	SQ−LNS N	IFA/SOC N	Mean difference (95% CI)
Weight−for−age z score (p=0.706)			
At least median	821	1616	0.03 (−0.07, 0.13)
Less than median	874	1671	0.04 (−0.06, 0.14)
Length−for−age z−score (p=0.332)			
At least median	822	1616	0.01 (−0.08, 0.10)
Less than median	874	1673	0.07 (−0.01, 0.16)
Weight−for−length z−score (p=0.497)			
At least median	821	1615	0.04 (−0.04, 0.12)
Less than median	874	1671	−0.02 (−0.11, 0.08)
Head circumference−for−age z−score (p=0.637)			
At least median	822	1614	0.02 (−0.06, 0.09)
Less than median	874	1671	−0.02 (−0.10, 0.07)
MUAC−for−age z−score (p=0.318)			
At least median	720	1524	−0.04 (−0.13, 0.05)
Less than median	775	1575	0.02 (−0.07, 0.11)

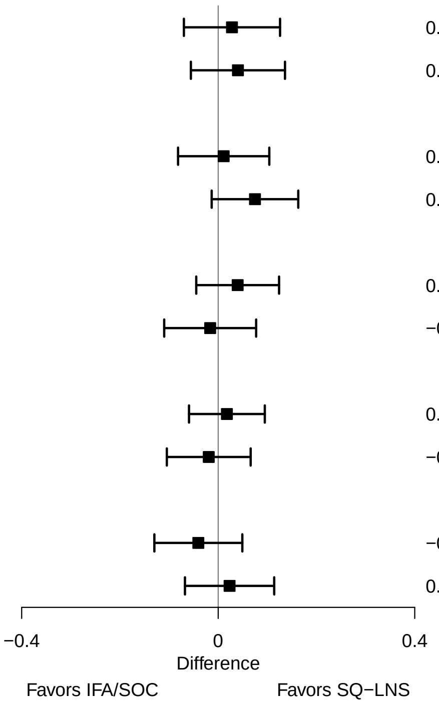

Supplemental figure 3L: Household socio-economic status

3L4: Prevalence ratios for 6 mo outcomes

Outcome (p−for−interaction)	SQ−LNS N	IFA/SOC N	Prevalence ratio (95% CI)
Underweight (p=0.498)			
At least median	821	1616	0.96 (0.73, 1.26)
Less than median	874	1671	0.89 (0.73, 1.09)
Stunted (p=0.664)			
At least median	822	1616	0.94 (0.79, 1.13)
Less than median	874	1673	0.90 (0.77, 1.04)
Wasted (p=0.992)			
At least median	719	1522	0.91 (0.56, 1.49)
Less than median	775	1573	0.98 (0.67, 1.44)
Low HCAZ (p=0.757)			
At least median	822	1614	0.88 (0.74, 1.06)
Less than median	874	1671	0.92 (0.76, 1.10)
Low MUAC (p=0.725)			
At least median	720	1524	1.07 (0.74, 1.54)
Less than median	775	1575	1.00 (0.75, 1.35)
Acute malnutrition (p=0.811)			
At least median	719	1522	0.97 (0.69, 1.36)
Less than median	775	1573	1.03 (0.80, 1.34)

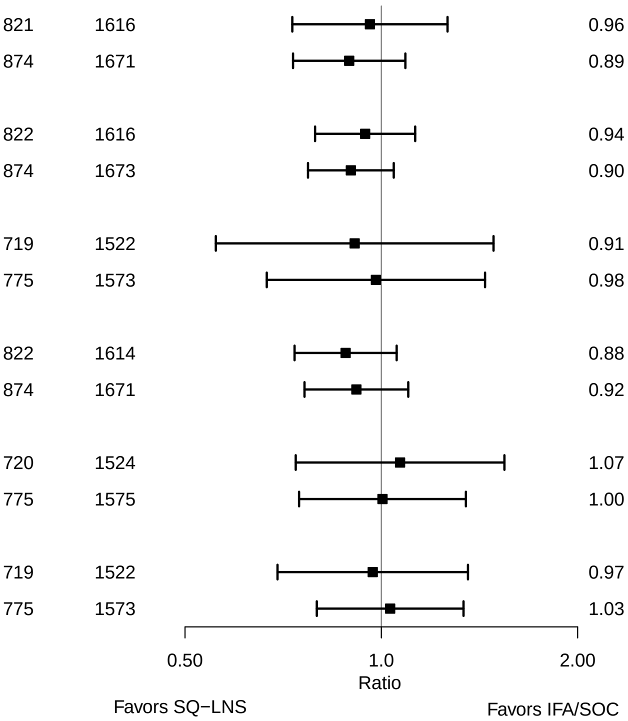

Supplemental figure 3M: Household food security

3M1: Mean differences for birth outcomes

Outcome (p−for−interaction)	SQ−LNS N	IFA/SOC N	Mean difference (95% CI)
Birth weight (kg) (p=0.103)			
Mild to secure	945	1884	0.03 (0.00, 0.06)
Moderate to severe	643	1352	0.07 (0.03, 0.11)
Weight−for−age z score (p=0.135)			
Mild to secure	945	1884	0.08 (0.01, 0.15)
Moderate to severe	643	1352	0.16 (0.06, 0.25)
Weight−for−gestational age z−score (p=0.526)			
Mild to secure	347	326	0.14 (−0.01, 0.29)
Moderate to severe	334	353	0.03 (−0.12, 0.18)
Birth length (cm) (p=0.024)			
Mild to secure	897	1844	0.09 (−0.05, 0.23)
Moderate to severe	556	1271	0.34 (0.15, 0.52)
Length−for−age z−score (p=0.047)			
Mild to secure	897	1844	0.06 (−0.02, 0.13)
Moderate to severe	556	1271	0.17 (0.08, 0.27)
Length−for−gestational age z−score (p=0.700)			
Mild to secure	300	287	0.12 (−0.05, 0.28)
Moderate to severe	247	272	0.07 (−0.12, 0.25)
BMI z−score (p=0.249)			
Mild to secure	897	1841	0.07 (−0.01, 0.15)
Moderate to severe	552	1266	0.13 (0.02, 0.24)
Head circumference (cm) (p=0.053)			
Mild to secure	898	1844	0.04 (−0.05, 0.13)
Moderate to severe	555	1273	0.21 (0.07, 0.35)
Head circumference−for−age z−score (p=0.144)			
Mild to secure	898	1844	0.05 (−0.03, 0.12)
Moderate to severe	555	1273	0.16 (0.05, 0.28)
Head circumference−for−gestational age z score (p=0.556)			
Mild to secure	301	286	0.18 (0.00, 0.35)
Moderate to severe	246	274	0.06 (−0.12, 0.24)
Mid−upper arm circumference (cm) (p=0.110)			
Mild to secure	900	1844	0.04 (−0.04, 0.12)
Moderate to severe	557	1273	0.12 (0.04, 0.20)
Duration (wk) (p=0.351)			
Mild to secure	981	1919	0.06 (−0.08, 0.21)
Moderate to severe	678	1389	0.21 (−0.03, 0.46)

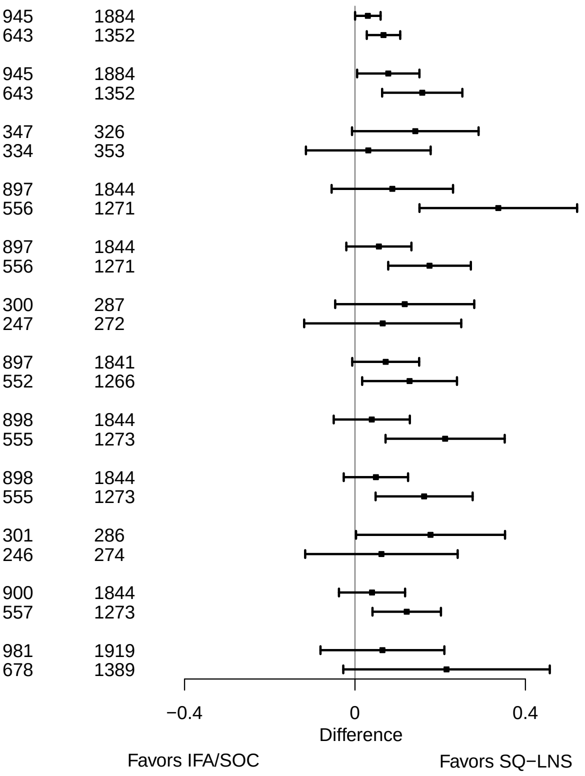

Supplemental figure 3M: Household food security

3M2: Relative risks for birth outcomes

Outcome (p−for−interaction)	SQ−LNS N	IFA/SOC N	Relative risk (95% CI)
Low birth weight (p=0.176)			
Mild to secure	945	1884	0.96 (0.84, 1.10)
Moderate to severe	643	1352	0.84 (0.71, 0.99)
Birth weight < 2 kg (p=0.189)			
Mild to secure	945	1884	0.90 (0.66, 1.23)
Moderate to severe	643	1352	0.63 (0.41, 0.99)
Small−for−gestational age (p=0.554)			
Mild to secure	945	1884	0.98 (0.92, 1.05)
Moderate to severe	643	1352	0.95 (0.86, 1.05)
Large−for−gestational age (p=0.279)			
Mild to secure	354	331	0.70 (0.30, 1.65)
Moderate to severe	336	354	1.10 (0.51, 2.36)
Newborn stunting (p=0.052)			
Mild to secure	897	1844	0.93 (0.80, 1.08)
Moderate to severe	556	1271	0.75 (0.61, 0.91)
Low LGAZ (p=0.514)			
Mild to secure	897	1844	0.88 (0.73, 1.05)
Moderate to severe	556	1271	0.96 (0.80, 1.17)
Low BMIZ (p=0.406)			
Mild to secure	897	1841	0.93 (0.81, 1.06)
Moderate to severe	552	1266	0.87 (0.75, 1.00)
Low HCZ (p=0.959)			
Mild to secure	898	1844	0.86 (0.72, 1.03)
Moderate to severe	555	1273	0.85 (0.70, 1.03)
Low HCGAZ (p=0.442)			
Mild to secure	811	1762	0.84 (0.70, 1.00)
Moderate to severe	392	1093	0.95 (0.74, 1.22)
Preterm birth (p=0.310)			
Mild to secure	981	1919	1.03 (0.86, 1.22)
Moderate to severe	678	1389	0.86 (0.66, 1.13)

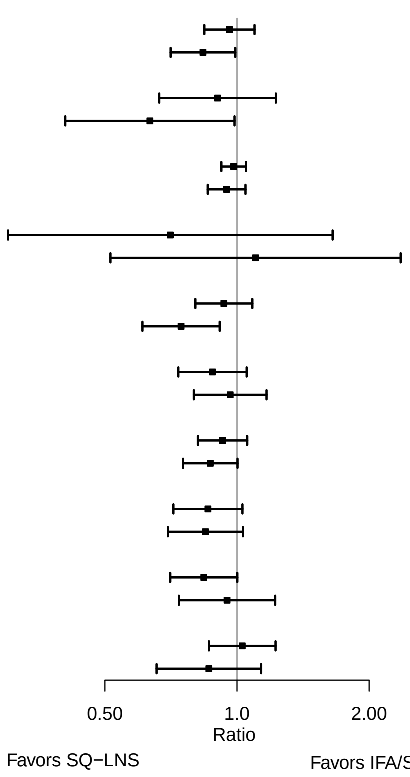

Supplemental figure 3M: Household food security

3M3: Mean differences for 6 mo outcomes

Outcome (p−for−interaction)	SQ−LNS N	IFA/SOC N	Mean difference (95% CI)
Weight−for−age z score (p=0.104)			
Mild to secure	891	1798	−0.03 (−0.14, 0.08)
Moderate to severe	603	1298	0.07 (−0.04, 0.18)
Length−for−age z−score (p=0.044)			
Mild to secure	891	1799	0.00 (−0.10, 0.09)
Moderate to severe	604	1299	0.10 (0.01, 0.19)
Weight−for−length z−score (p=0.657)			
Mild to secure	891	1797	−0.03 (−0.13, 0.07)
Moderate to severe	603	1298	−0.01 (−0.12, 0.11)
Head circumference−for−age z−score (p=0.378)			
Mild to secure	891	1800	−0.01 (−0.09, 0.07)
Moderate to severe	604	1297	0.02 (−0.07, 0.11)
MUAC−for−age z−score (p=0.215)			
Mild to secure	891	1800	−0.05 (−0.15, 0.05)
Moderate to severe	604	1299	0.04 (−0.06, 0.14)

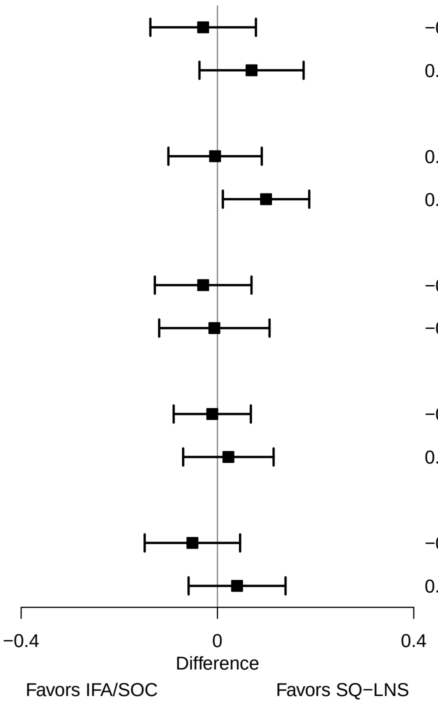

Supplemental figure 3M: Household food security

3M4: Prevalence ratios for 6 mo outcomes

**Outcome (p−for−interaction)**	**SQ−LNS N**	**IFA/SOC N**	**Prevalence ratio (95% CI)**
Underweight (p=0.053)			
Mild to secure	891	1798	1.10 (0.86, 1.40)
Moderate to severe	603	1298	0.84 (0.66, 1.07)
Stunted (p=0.173)			
Mild to secure	891	1799	1.03 (0.84, 1.25)
Moderate to severe	604	1299	0.85 (0.72, 1.00)
Wasted (p=0.965)			
Mild to secure	891	1797	0.92 (0.59, 1.42)
Moderate to severe	603	1298	1.04 (0.64, 1.68)
Low HCAZ (p=0.250)			
Mild to secure	891	1800	0.97 (0.82, 1.14)
Moderate to severe	604	1297	0.85 (0.71, 1.02)
Low MUAC (p=0.552)			
Mild to secure	891	1800	1.14 (0.82, 1.58)
Moderate to severe	604	1299	0.95 (0.68, 1.32)
Acute malnutrition (p=0.980)			
Mild to secure	891	1797	1.03 (0.77, 1.38)
Moderate to severe	603	1298	0.98 (0.71, 1.35)

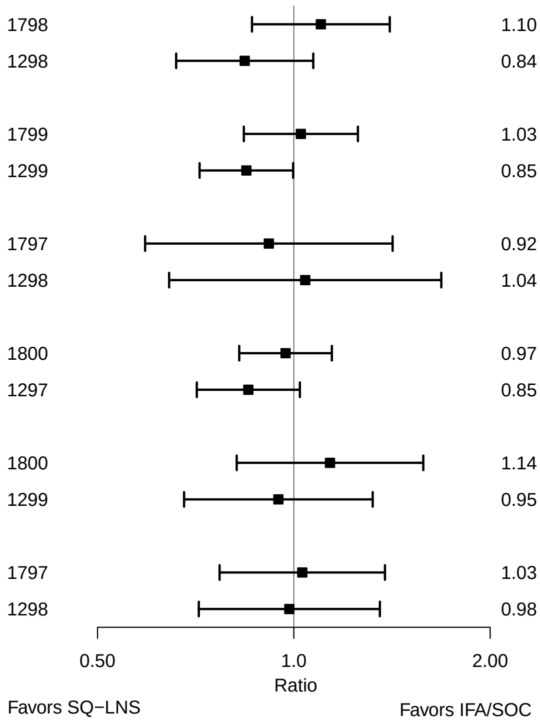

Supplemental figure 3N: Sanitation

3N1: Mean differences for birth outcomes

Outcome (p−for−interaction)	SQ−LNS N	IFA/SOC N	Mean difference (95% CI)
Birth weight (kg) (p=0.194)			
Improved	1074	2212	0.07 (0.04, 0.10)
Unimproved	738	1238	0.03 (0.00, 0.06)
Weight−for−age z score (p=0.141)			
Improved	1074	2212	0.17 (0.10, 0.24)
Unimproved	738	1238	0.07 (−0.01, 0.15)
Weight−for−gestational age z−score (p=0.009)			
Improved	113	111	0.36 (0.21, 0.52)
Unimproved	454	437	0.00 (−0.11, 0.12)
Birth length (cm) (p=0.978)			
Improved	1060	2199	0.19 (0.06, 0.32)
Unimproved	618	1130	0.21 (0.02, 0.40)
Length−for−age z−score (p=0.720)			
Improved	1060	2199	0.11 (0.04, 0.18)
Unimproved	618	1130	0.11 (0.01, 0.21)
Length−for−gestational age z−score (p=0.278)			
Improved	100	99	0.25 (0.05, 0.44)
Unimproved	334	329	0.07 (−0.08, 0.21)
BMI z−score (p=0.073)			
Improved	1060	2199	0.17 (0.09, 0.25)
Unimproved	614	1122	0.01 (−0.09, 0.11)
Head circumference (cm) (p=0.323)			
Improved	1059	2200	0.09 (0.00, 0.18)
Unimproved	619	1131	0.13 (0.01, 0.25)
Head circumference−for−age z−score (p=0.511)			
Improved	1059	2200	0.09 (0.01, 0.16)
Unimproved	619	1131	0.10 (0.01, 0.20)
Head circumference−for−gestational age z score (p=0.985)			
Improved	99	99	0.08 (−0.15, 0.31)
Unimproved	335	330	0.06 (−0.08, 0.20)
Mid−upper arm circumference (cm) (p=0.873)			
Improved	960	2105	0.08 (0.01, 0.15)
Unimproved	499	1010	0.06 (−0.02, 0.14)
Duration (wk) (p=0.963)			
Improved	1090	2227	0.11 (−0.04, 0.26)
Unimproved	760	1257	0.15 (−0.05, 0.34)

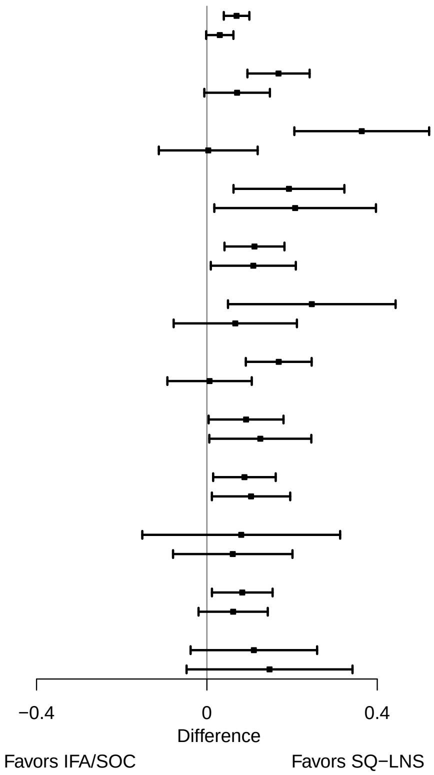

Supplemental figure 3N: Sanitation

3N2: Relative risks for birth outcomes

**Outcome (p−for−interaction)**	**SQ−LNS N**	**IFA/SOC N**	**Relative risk (95% CI)**
Low birth weight (p=0.324)			
Improved	1074	2212	0.86 (0.75, 0.98)
Unimproved	738	1238	0.95 (0.83, 1.09)
Birth weight < 2 kg (p=0.023)			
Improved	676	1824	0.91 (0.66, 1.24)
Unimproved	607	1106	0.61 (0.42, 0.87)
Small−for−gestational age (p=0.121)			
Improved	1056	2194	0.94 (0.88, 1.00)
Unimproved	714	1206	1.02 (0.94, 1.12)
Newborn stunting (p=0.252)			
Improved	1043	2177	0.88 (0.76, 1.02)
Unimproved	382	890	0.76 (0.62, 0.93)
Low LGAZ (p=0.740)			
Improved	1042	2181	0.90 (0.77, 1.04)
Unimproved	594	1098	0.93 (0.77, 1.14)
Low BMIZ (p=0.130)			
Improved	763	1906	0.86 (0.77, 0.97)
Unimproved	605	1111	1.01 (0.86, 1.17)
Low HCZ (p=0.678)			
Improved	746	1885	0.87 (0.74, 1.03)
Unimproved	373	879	0.87 (0.70, 1.09)
Low HCGAZ (p=0.528)			
Improved	728	1867	0.91 (0.77, 1.09)
Unimproved	349	847	0.86 (0.69, 1.06)
Preterm birth (p=0.727)			
Improved	770	1912	0.98 (0.78, 1.24)
Unimproved	750	1245	0.88 (0.70, 1.11)

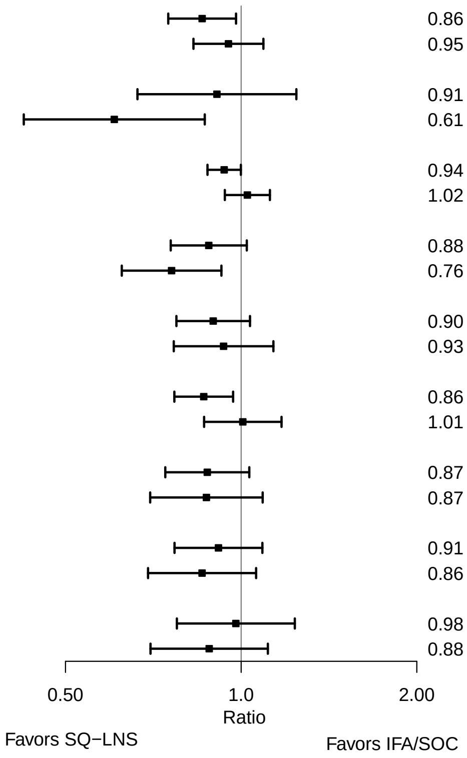

Supplemental figure 3N: Sanitation

3N3: Mean differences for 6 mo outcomes

Outcome (p−for−interaction)	SQ−LNS N	IFA/SOC N	Mean difference (95% CI)
Weight−for−age z score (p=0.097)			
Improved	1018	2125	0.10 (0.01, 0.19)
Unimproved	696	1183	−0.02 (−0.13, 0.09)
Length−for−age z−score (p=0.601)			
Improved	1019	2126	0.07 (−0.01, 0.15)
Unimproved	696	1184	0.03 (−0.07, 0.14)
Weight−for−length z−score (p=0.044)			
Improved	1018	2124	0.04 (−0.05, 0.13)
Unimproved	696	1183	−0.07 (−0.17, 0.03)
Head circumference−for−age z−score (p=0.252)			
Improved	1019	2126	0.04 (−0.03, 0.11)
Unimproved	696	1180	−0.03 (−0.12, 0.06)
MUAC−for−age z−score (p=0.138)			
Improved	919	2029	0.01 (−0.08, 0.09)
Unimproved	577	1068	−0.03 (−0.14, 0.08)

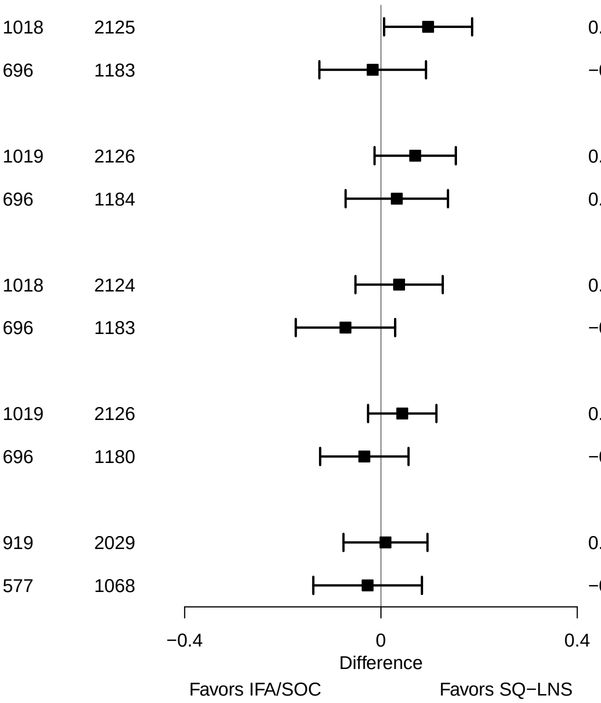

Supplemental figure 3N: Sanitation

3N4: Prevalence ratios for 6 mo outcomes

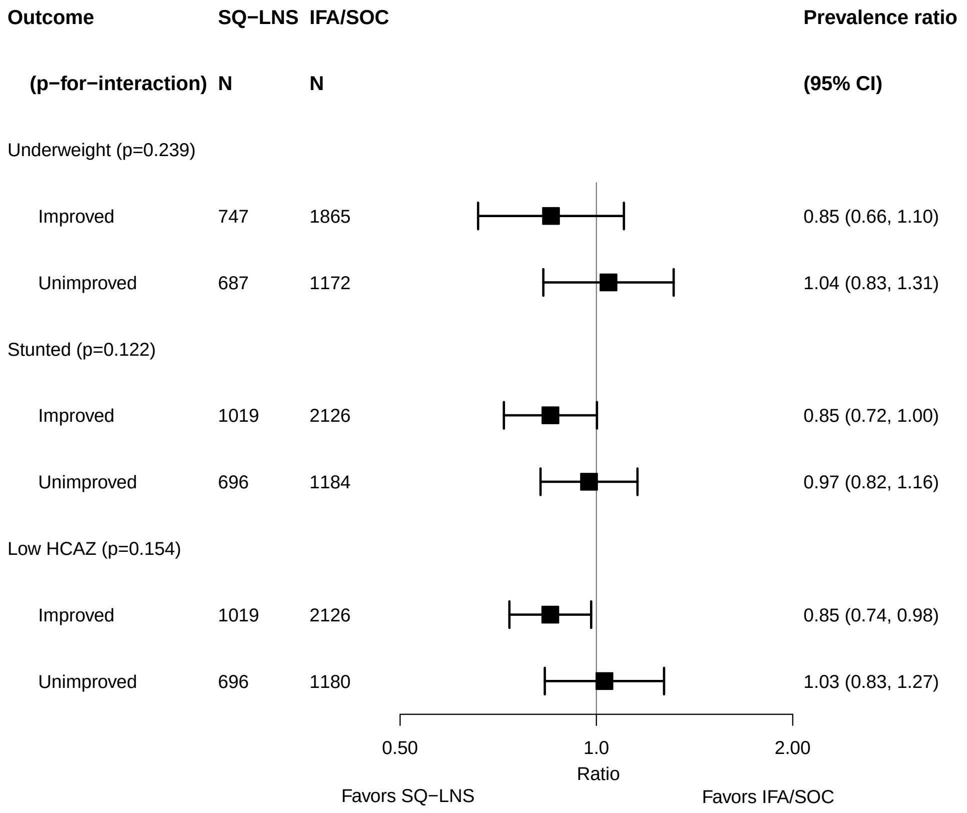

Supplement: Multimedia component 1 [file mmc1.zip › Maternal SQ-LNS Supplemental_2024-09-03/8_Maternal SQ-LNS Supplemental figure 3.pdf]
