# Supplementary material for: Effects of prenatal small-quantity lipid-based nutrient supplements on pregnancy, birth, and infant outcomes: a systematic review and meta-analysis of individual participant data from randomized controlled trials in low- and middle-income countries
Source: Am J Clin Nutr. 2024 Aug 16;120(4):814–35. doi: 10.1016/j.ajcnut.2024.08.008 (PMC11473441; doi:10.1016/j.ajcnut.2024.08.008)

Supplemental figure 4: Pooled plots for adverse outcomes stratified by potential effect modifiers, SQ-LNS vs IFA/SOC

Contents

Supplemental figure 4A: Sex 2

Supplemental figure 4B: Birth order 3

Supplemental figure 4C: Maternal height 4

Supplemental figure 4D: Maternal BMI 5

Supplemental figure 4E: Maternal age 6

Supplemental figure 4F: Maternal education 7

Supplemental figure 4G: Baseline anemia status 8

Supplemental figure 4H: Baseline inflammation status 9

Supplemental figure 4I: Baseline malaria status 10

Supplemental figure 4J: Gestational age at supplementation 11

Supplemental figure 4K: Compliance with supplementation 12

Supplemental figure 4L: Household socio-economic status 13

Supplemental figure 4M: Household food security 14

Supplemental figure 4N: Sanitation 15

These figures show pooled effects of SQ-LNS within study-level and individual-level characteristic subgroups along with the p-for-interaction. For definitions of effect modifiers, see Box 1 in the main paper. Individual study estimates were generated from log-binomial regression for dichotomous outcomes and simple linear regression for continuous outcomes with clustered observations using robust standard errors for cluster-randomized trials. Pooled sub-group estimates and statistical testing of the pooled interaction term were generated using inverse-variance weighting. For dichotomous outcomes analyzed via prevalence/risk ratios, the effect estimate is the prevalence/risk in the SQ-LNS group divided by the prevalence/risk in the IFA/SOC group. For dichotomous outcomes analyzed via prevalence/risk differences, the effect estimate is the prevalence/risk in the SQ-LNS group minus the prevalence/risk in the IFA/SOC group. The labels on the left y-axis correspond to the characteristic subgroups and their sample sizes. The values on the right indicate the pooled prevalence ratio and confidence interval within that subgroup.

LAZ, length-for-age z-score; WLZ, weight-for-length z-score; WAZ, weight for-age z-score; MUACZ, mid-upper arm circumference z-score; BMI, body mass index; HCZ, head circumference-for-age z-score; LGAZ, length-for-gestational-age z-score; HCGAZ, head circumference-for-gestational-age z-score; BMIZ, body mass index-for-age z-score; IFA/SOC, Iron and folic acid or standard of care; MD, mean difference; MMS, multiple micronutrient supplement; MUAC, mid-upper arm circumference; PR, prevalence ratio; PD, prevalence difference; RD, risk difference; RR, relative risk; SOC, standard of care; SQ-LNS, small-quantity lipid-based nutrient supplements; WGAZ, weight-for-gestational age z-score.

Supplemental figure 4A: Sex

Outcome (p−for−interaction)	SQ−LNS N	IFA/SOC N	Relative risk (95% CI)
C−section (p=0.037)			
Male	977	1874	1.01 (0.83, 1.22)
Female	984	1840	1.11 (0.88, 1.39)
Neonatal mortality (p=0.306)			
Male	662	1544	0.65 (0.33, 1.26)
Female	672	1516	1.08 (0.60, 1.93)
Miscarriage or stillbirth or early neonatal mortality (p=0.405)			
Male	980	1883	1.03 (0.71, 1.50)
Female	989	1853	1.27 (0.85, 1.91)
Mortality 0−6 mo (p=0.579)			
Male	778	1670	0.86 (0.52, 1.41)
Female	798	1637	1.04 (0.65, 1.65)

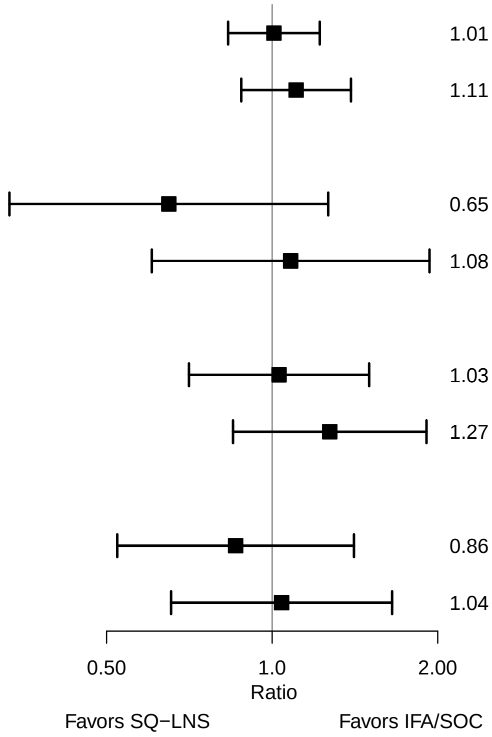

Supplemental figure 4B: Birth order

**Outcome (p−for−interaction)**	**SQ−LNS N**	**IFA/SOC N**	**Relative risk (95% CI)**
C−section (p=0.199)			
Later born	1337	2418	1.01 (0.87, 1.17)
Firstborn	630	1301	0.96 (0.75, 1.24)
Neonatal mortality (p=0.024)			
Later born	1079	2138	1.08 (0.69, 1.70)
Firstborn	594	1257	0.54 (0.27, 1.06)
Miscarriage or stillbirth or early neonatal mortality (p=0.657)			
Later born	1421	2619	0.91 (0.73, 1.13)
Firstborn	670	1381	0.97 (0.73, 1.30)
Mortality 0−6 mo (p=0.037)			
Later born	1092	2156	1.22 (0.86, 1.73)
Firstborn	598	1267	0.60 (0.32, 1.15)

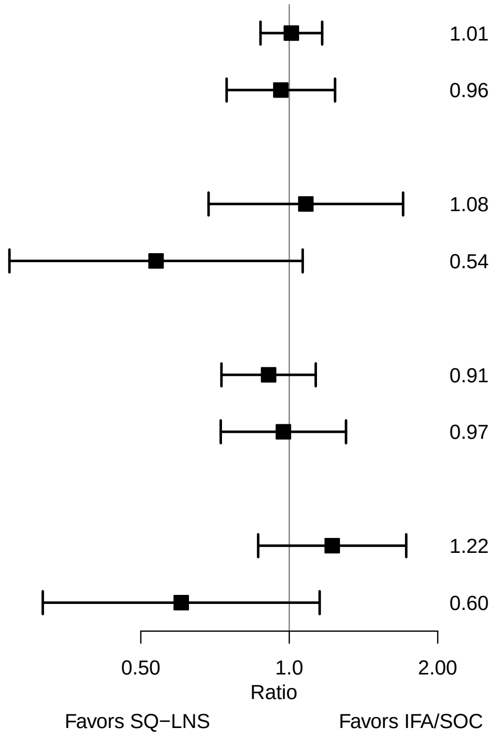

Supplemental figure 4C: Maternal height

Outcome (p−for−interaction)	SQ−LNS N	IFA/SOC N	Relative risk (95% CI)
C−section (p=0.288)			
At least 150.1 cm	1244	2099	1.06 (0.87, 1.29)
Less than 150.1 cm	679	1529	1.00 (0.83, 1.22)
Neonatal mortality (p=0.315)			
At least 150.1 cm	900	1766	0.61 (0.36, 1.05)
Less than 150.1 cm	630	1449	0.94 (0.47, 1.86)
Miscarriage or stillbirth or early neonatal mortality (p=0.086)			
At least 150.1 cm	1308	2238	0.80 (0.63, 1.03)
Less than 150.1 cm	735	1649	1.07 (0.81, 1.41)
Mortality 0−6 mo (p=0.523)			
At least 150.1 cm	908	1771	0.80 (0.51, 1.24)
Less than 150.1 cm	637	1466	0.99 (0.60, 1.61)

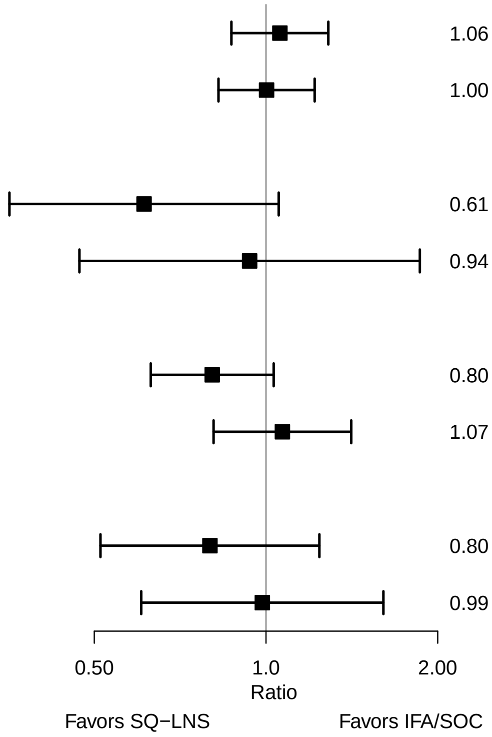

Supplemental figure 4D: Maternal BMI

Outcome (p−for−interaction)	SQ−LNS N	IFA/SOC N	Relative risk (95% CI)
C−section (p=0.987)			
At least 20 kg/m²	1123	1937	1.04 (0.91, 1.19)
Less than 20 kg/m²	799	1689	1.24 (0.86, 1.78)
Neonatal mortality (p=0.472)			
At least 20 kg/m²	611	1419	0.79 (0.42, 1.48)
Less than 20 kg/m²	693	1569	0.70 (0.40, 1.23)
Miscarriage or stillbirth or early neonatal mortality (p=0.879)			
At least 20 kg/m²	910	1804	0.94 (0.72, 1.23)
Less than 20 kg/m²	783	1739	0.98 (0.72, 1.34)
Mortality 0−6 mo (p=0.329)			
At least 20 kg/m²	620	1429	1.04 (0.66, 1.64)
Less than 20 kg/m²	701	1586	0.82 (0.52, 1.28)

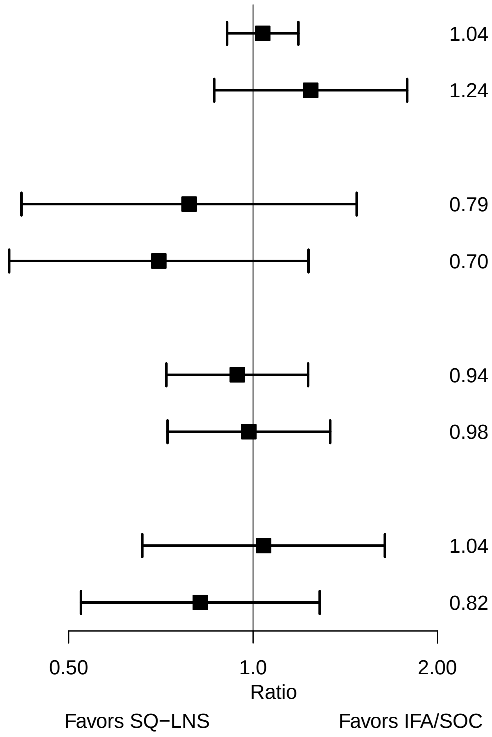

Supplemental figure 4E: Maternal age

**Outcome (p−for−interaction)**	**SQ−LNS N**	**IFA/SOC N**	**Relative risk (95% CI)**
C−section (p=0.994)			
At least 25 y	777	1262	1.02 (0.84, 1.24)
Less than 25 y	1191	2463	1.08 (0.88, 1.31)
Neonatal mortality (p=0.424)			
At least 25 y	546	1035	0.61 (0.33, 1.13)
Less than 25 y	1024	2267	0.97 (0.57, 1.65)
Miscarriage or stillbirth or early neonatal mortality (p=0.505)			
At least 25 y	827	1373	0.90 (0.71, 1.15)
Less than 25 y	1269	2633	1.00 (0.79, 1.26)
Mortality 0−6 mo (p=0.918)			
At least 25 y	555	1039	0.86 (0.54, 1.40)
Less than 25 y	1030	2286	0.96 (0.63, 1.46)

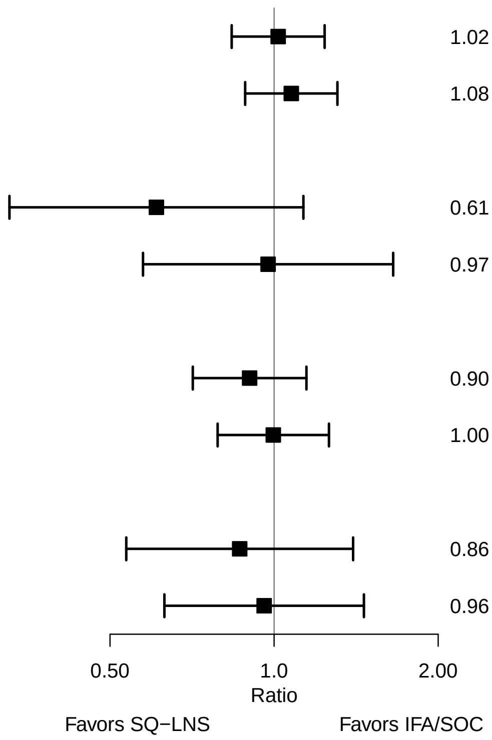

Supplemental figure 4F: Maternal education

Outcome (p−for−interaction)	SQ−LNS N	IFA/SOC N	Relative risk (95% CI)
C−section (p=0.919)			
Primary or greater	1301	2503	1.01 (0.87, 1.16)
Incomplete or no formal	663	1218	1.17 (0.86, 1.59)
Neonatal mortality (p=0.871)			
Primary or greater	1056	2232	1.06 (0.56, 1.98)
Incomplete or no formal	616	1165	0.80 (0.48, 1.33)
Miscarriage or stillbirth or early neonatal mortality (p=0.936)			
Primary or greater	1393	2687	0.93 (0.74, 1.16)
Incomplete or no formal	699	1314	0.95 (0.70, 1.30)
Mortality 0−6 mo (p=0.079)			
Primary or greater	1059	2247	0.92 (0.54, 1.57)
Incomplete or no formal	630	1178	1.36 (0.98, 1.88)

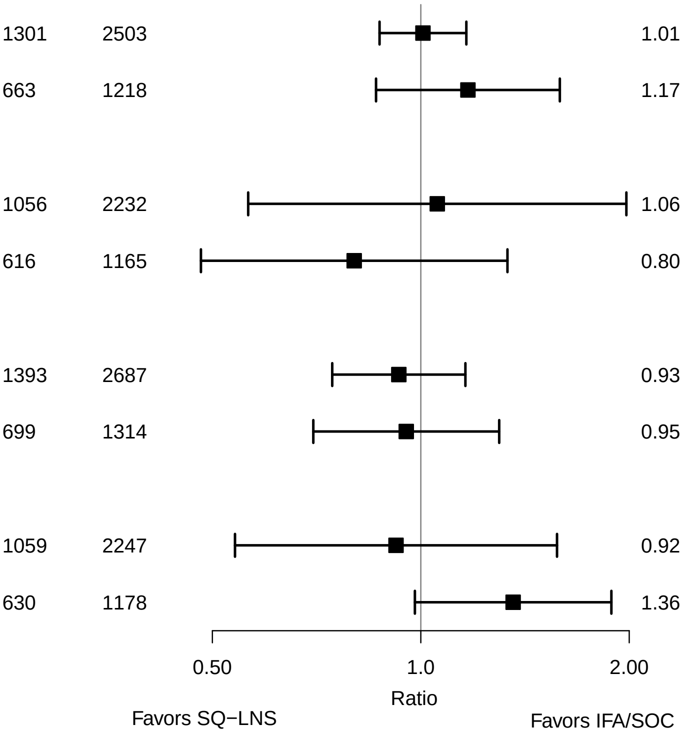

Supplemental figure 4G: Baseline anemia status

**Outcome** (p−for−interaction)	**SQ−LNS** N	**IFA/SOC** N	**Relative risk** (95% CI)
C−section (p=0.571)			
Anemia	474	461	0.99 (0.68, 1.42)
No anemia	867	746	1.12 (0.86, 1.46)
Neonatal mortality (p=0.084)			
Anemia	458	459	1.24 (0.57, 2.67)
No anemia	836	741	0.64 (0.34, 1.20)
Miscarriage or stillbirth or early neonatal mortality (p=0.540)			
Anemia	492	490	1.05 (0.67, 1.67)
No anemia	912	797	0.93 (0.68, 1.26)
Mortality 0−6 mo (p=0.438)			
Anemia	460	462	1.15 (0.58, 2.31)
No anemia	846	742	0.94 (0.55, 1.61)

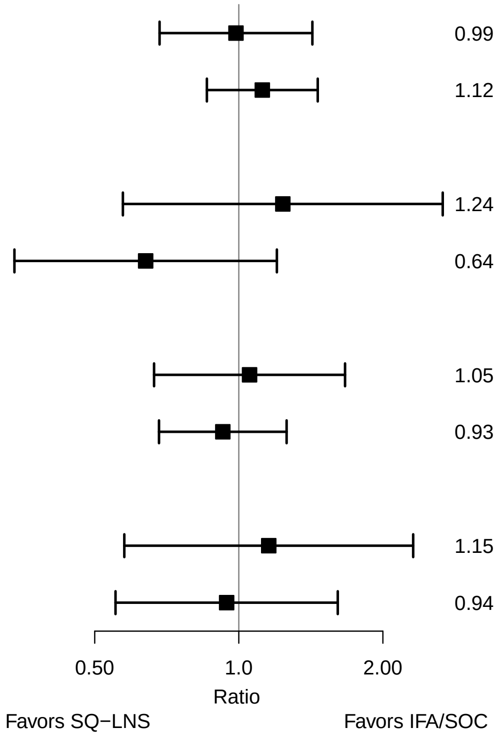

Supplemental figure 4H: Baseline inflammation status

Outcome (p−for−interaction)	SQ−LNS N	IFA/SOC N	Relative risk (95% CI)
C−section (p=0.921)			
Not inflamed	892	817	1.04 (0.78, 1.37)
Inflamed	433	371	1.06 (0.75, 1.51)
Neonatal mortality (p=0.317)			
Not inflamed	856	809	0.91 (0.48, 1.72)
Inflamed	423	372	0.50 (0.20, 1.23)
Miscarriage or stillbirth or early neonatal mortality (p=0.399)			
Not inflamed	930	867	1.03 (0.73, 1.44)
Inflamed	457	400	0.73 (0.44, 1.21)
Mortality 0−6 mo (p=0.111)			
Not inflamed	866	811	1.24 (0.70, 2.19)
Inflamed	425	374	0.53 (0.24, 1.17)

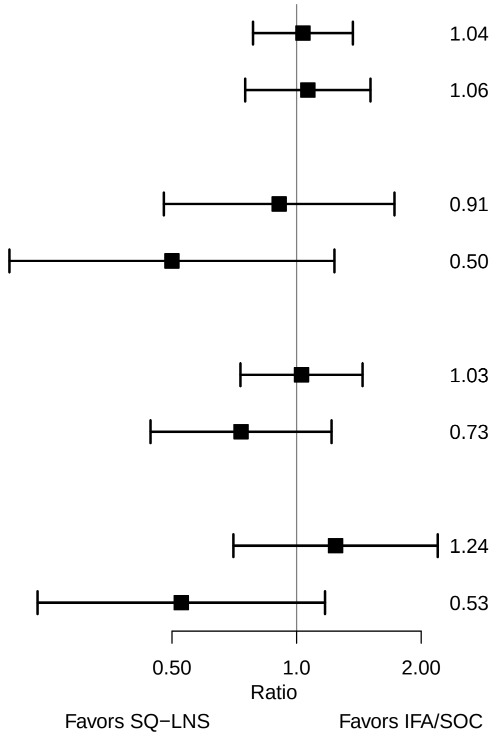

Supplemental figure 4I: Baseline malaria status

**Outcome (p−for−interaction)**	**SQ−LNS N**	**IFA/SOC N**	**Relative risk (95% CI)**
C−section (p=0.018)			
No malaria	616	614	1.24 (0.92, 1.67)
Malaria	132	118	0.53 (0.20, 1.41)
Miscarriage or stillbirth or early neonatal mortality (p=0.620)			
No malaria	648	655	0.83 (0.54, 1.27)
Malaria	135	127	1.18 (0.44, 3.16)

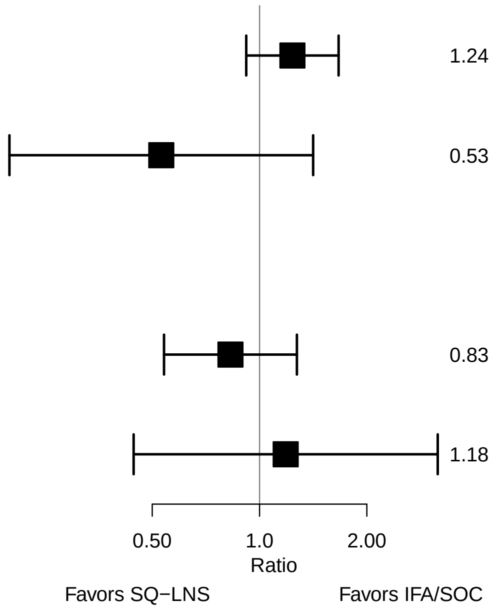

Supplemental figure 4J: Gestational age at supplementation

Outcome (p−for−interaction)	SQ−LNS N	IFA/SOC N	Relative risk (95% CI)
C−section (p=0.796)			
< 14 wks	914	1971	1.02 (0.87, 1.20)
>= 14 wks	1031	1715	1.10 (0.87, 1.39)
Neonatal mortality (p=0.877)			
< 14 wks	621	1662	0.82 (0.44, 1.51)
>= 14 wks	714	1404	0.71 (0.41, 1.23)
Miscarriage or stillbirth or early neonatal mortality (p=0.178)			
< 14 wks	962	2109	1.07 (0.81, 1.41)
>= 14 wks	1070	1799	0.83 (0.62, 1.11)
Mortality 0−6 mo (p=0.712)			
< 14 wks	627	1675	0.91 (0.51, 1.64)
>= 14 wks	725	1418	0.94 (0.64, 1.40)

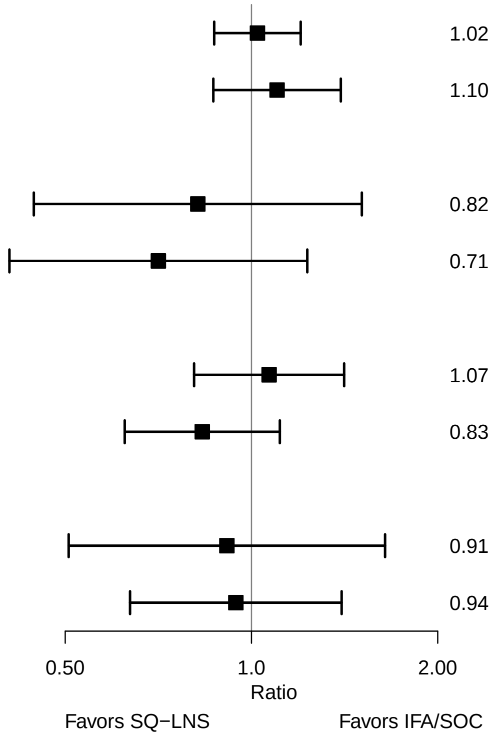

Supplemental figure 4K: Compliance with supplementation

Outcome (p−for−interaction)	SQ−LNS N	IFA/SOC N	Relative risk (95% CI)
C−section (p=0.247)			
At least 4/7 d	1269	3170	1.05 (0.84, 1.31)
Less than 4/7 d	450	286	1.26 (0.85, 1.86)
Miscarriage or stillbirth or early neonatal mortality (p=0.787)			
At least 4/7 d	890	2807	0.89 (0.58, 1.36)
Less than 4/7 d	434	268	1.07 (0.52, 2.18)

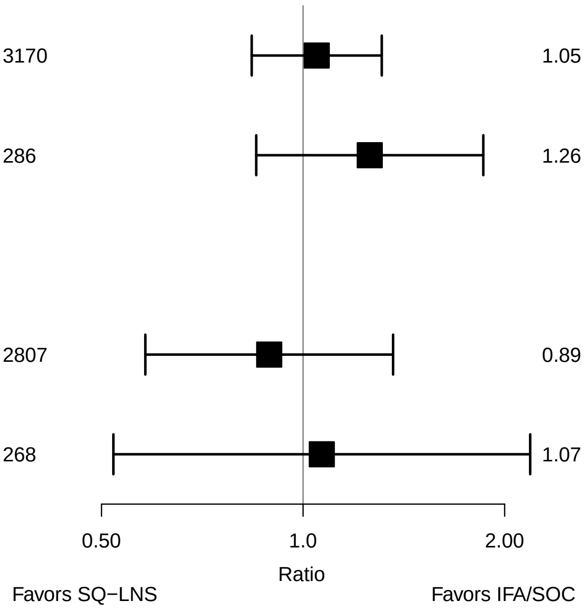

Supplemental figure 4L: Household socio-economic status

**Outcome (p−for−interaction)**	**SQ−LNS N**	**IFA/SOC N**	**Relative risk (95% CI)**
C−section (p=0.700)			
At least median	932	1812	1.06 (0.87, 1.29)
Less than median	998	1867	1.15 (0.89, 1.48)
Neonatal mortality (p=0.082)			
At least median	812	1687	0.60 (0.32, 1.12)
Less than median	860	1708	1.03 (0.65, 1.65)
Miscarriage or stillbirth or early neonatal mortality (p=0.131)			
At least median	869	1828	0.82 (0.61, 1.10)
Less than median	950	1895	1.08 (0.84, 1.39)
Mortality 0−6 mo (p=0.127)			
At least median	816	1693	0.72 (0.41, 1.28)
Less than median	873	1729	1.15 (0.81, 1.64)

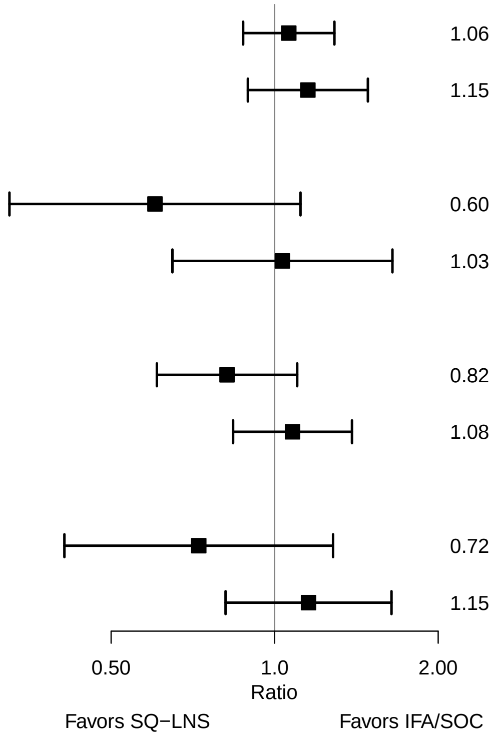

Supplemental figure 4M: Household food security

Outcome (p−for−interaction)	SQ−LNS N	IFA/SOC N	Relative risk (95% CI)
C−section (p=0.838)			
Mild to secure	1031	2035	1.14 (0.90, 1.45)
Moderate to severe	697	1453	1.08 (0.78, 1.51)
Neonatal mortality (p=0.895)			
Mild to secure	997	1982	0.95 (0.58, 1.54)
Moderate to severe	675	1413	0.78 (0.43, 1.41)
Miscarriage or stillbirth or early neonatal mortality (p=0.318)			
Mild to secure	1076	2174	0.87 (0.66, 1.14)
Moderate to severe	743	1551	1.08 (0.80, 1.45)
Mortality 0−6 mo (p=0.849)			
Mild to secure	1005	1992	1.06 (0.73, 1.52)
Moderate to severe	684	1431	0.92 (0.57, 1.49)

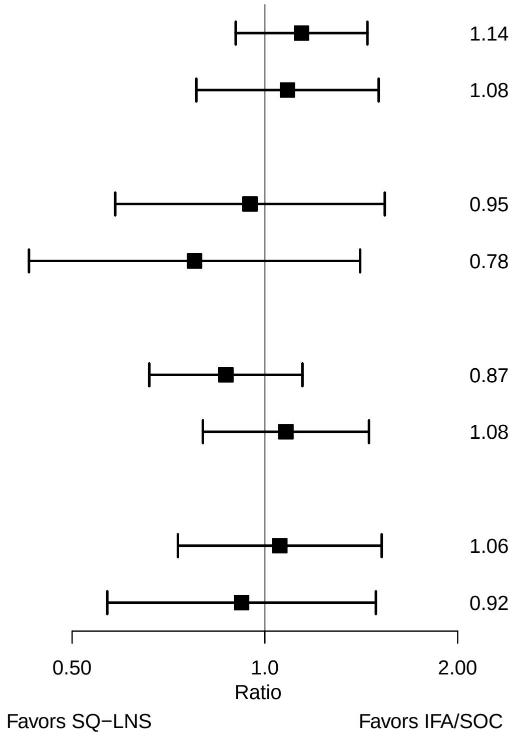

Supplemental figure 4N: Sanitation

Outcome (p−for−interaction)	SQ−LNS N	IFA/SOC N	Relative risk (95% CI)
C−section (p=0.483)			
Improved	1166	2397	1.00 (0.82, 1.22)
Unimproved	798	1320	1.19 (0.93, 1.53)
Miscarriage or stillbirth or early neonatal mortality (p=0.251)			
Improved	865	2197	0.84 (0.64, 1.10)
Unimproved	445	1024	1.00 (0.74, 1.35)
Mortality 0−6 mo (p=0.919)			
Improved	786	1992	1.03 (0.67, 1.58)
Unimproved	392	914	0.99 (0.50, 1.98)

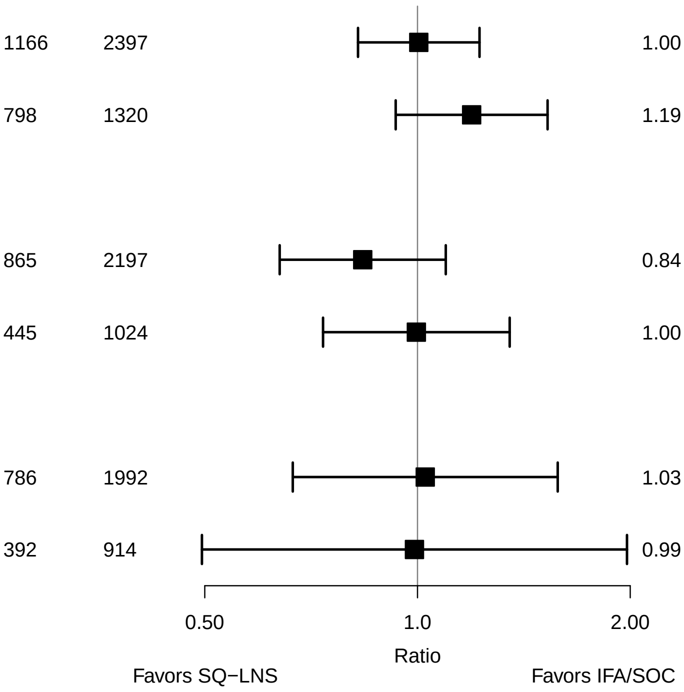

Supplement: Multimedia component 1 [file mmc1.zip › Maternal SQ-LNS Supplemental_2024-09-03/9_Maternal SQ-LNS Supplemental figure 4.pdf]
