# Supplementary figures and images for: Effects of prenatal small-quantity lipid-based nutrient supplements on pregnancy, birth, and infant outcomes: a systematic review and meta-analysis of individual participant data from randomized controlled trials in low- and middle-income countries
Source: Am J Clin Nutr. 2024 Aug 16;120(4):814–35. doi: 10.1016/j.ajcnut.2024.08.008 (PMC11473441; doi:10.1016/j.ajcnut.2024.08.008)

Supplemental Figure 1: Summary risk of bias as a percentage of all included studies

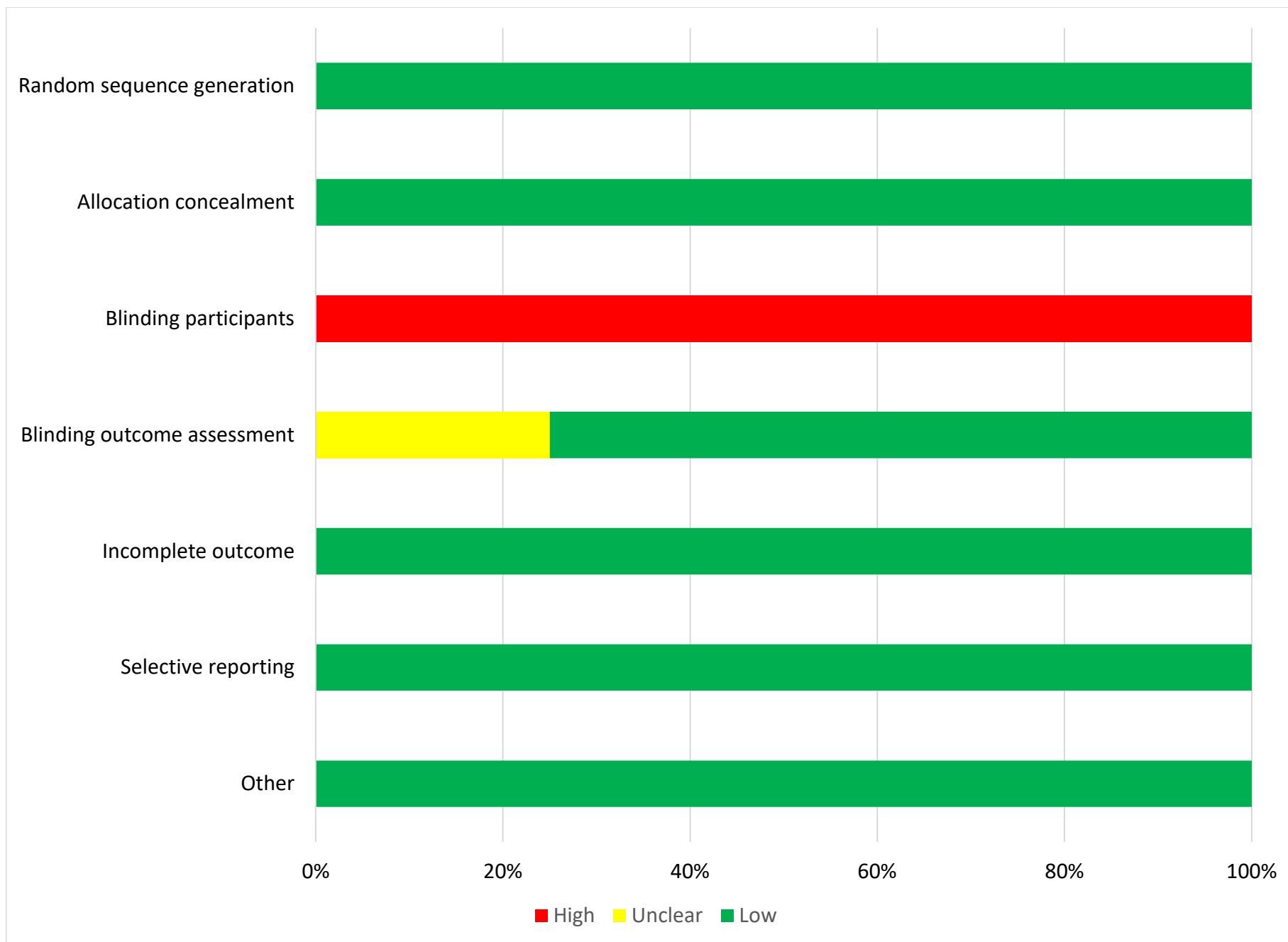

Supplement: Multimedia component 1 [file mmc1.zip › Maternal SQ-LNS Supplemental_2024-09-03/6_Maternal SQ-LNS Supplemental figure 1.pdf]
